# Mapping knowledge of the stem cell in traumatic brain injury: a bibliometric and visualized analysis

**DOI:** 10.3389/fneur.2024.1301277

**Published:** 2024-03-08

**Authors:** Tingzhen Deng, Ruiwen Ding, Yatao Wang, Yueyang Chen, Hongtao Sun, Maohua Zheng

**Affiliations:** ^1^The First School of Clinical Medicine, Lanzhou University, Lanzhou, China; ^2^Department of Neurosurgery, The First Hospital of Lanzhou University, Lanzhou, China; ^3^Tianjin Key Laboratory of Neurotrauma Repair, Institute of Neurotrauma Repair, Characteristic Medical Center of Chinese People’s Armed Police Force, Tianjin, China

**Keywords:** traumatic brain injury, TBI, stem cell, bibliometric analysis, VOSviewer, CiteSpace

## Abstract

**Background:**

Traumatic brain injury (TBI) is a brain function injury caused by external mechanical injury. Primary and secondary injuries cause neurological deficits that mature brain tissue cannot repair itself. Stem cells can self-renewal and differentiate, the research of stem cells in the pathogenesis and treatment of TBI has made significant progress in recent years. However, numerous articles must be summarized to analyze hot spots and predict trends. This study aims to provide a panorama of knowledge and research hotspots through bibliometrics.

**Method:**

We searched in the Web of Science Core Collection (WoSCC) database to identify articles pertaining to TBI and stem cells published between 2000 and 2022. Visualization knowledge maps, including co-authorship, co-citation, and co-occurrence analysis were generated by VOSviewer, CiteSpace, and the R package “bibliometrix.”

**Results:**

We retrieved a total of 459 articles from 45 countries. The United States and China contributed the majority of publications. The number of publications related to TBI and stem cells is increasing yearly. Tianjin Medical University was the most prolific institution, and Professor Charles S. Cox, Jr. from the University of Texas Health Science Center at Houston was the most influential author. The *Journal of Neurotrauma* has published the most research articles on TBI and stem cells. Based on the burst references, “immunomodulation,” “TBI,” and “cellular therapy” have been regarded as research hotspots in the field. The keywords co-occurrence analysis revealed that “exosomes,” “neuroinflammation,” and “microglia” were essential research directions in the future.

**Conclusion:**

Research on TBI and stem cells has shown a rapid growth trend in recent years. Existing studies mainly focus on the activation mechanism of endogenous neural stem cells and how to make exogenous stem cell therapy more effective. The combination with bioengineering technology is the trend in this field. Topics related to exosomes and immune regulation may be the future focus of TBI and stem cell research.

## 1 Introduction

Traumatic brain injury (TBI) is an intracranial injury caused by external mechanical damage, it is a significant health and socioeconomic problem around the world, with 50–60 million people suffering from TBI annually, and costing the global economy around $400 billion each year (1). The pathogenesis is a complex and dynamic process, with primary and secondary injuries that lead to impermanent or permanent neurological deficits (2, 3). The primary deficit is directly related to the external impact of the brain, and the secondary injury consists of a chemical, molecular, and inflammatory cascade responsible for further cerebral damage (4). Unlike other organs, mature brain tissue cannot self-repair after damage (5, 6). About half of the TBI patients could not return to their previous work after 1 year, and ~ 28% never returned to work in any form (7). Numerous monitoring, drug treatments, and operations of TBI exist to reduce neurological damage, unfortunately, there is no effective clinical treatment to improve neural repair and functional recovery of patients (8–11).

Stem cells possess the capacity for differentiation and self-renewal, enabling them to differentiate into specialized cell types necessary for tissue repair (12). Stem cell therapy exerts beneficial effects on neurodegeneration and functional recovery through diverse mechanisms, including the secretion of chemokines and growth factors, the promotion of neurogenesis and angiogenesis, and the regulation of neuroinflammation (13–15). In recent decades, researchers have extensively investigated exogenous stem/progenitor cells for promoting recovery from TBI, spinal cord injury (SCI), Alzheimer’s disease, and stroke (14, 16–20). A growing body of evidence supports the use of different types of stem cells to treat TBI, both mesenchymal stem cells (MSCs), neural stem cells (adult or embryonic), and multipotent adult progenitor cells (MAPCs) have all shown efficacy in preclinical models of TBI (21). Furthermore, scholars observed the activation and proliferation of endogenous neural stem cells after TBI (22, 23). Despite unresolved issues in recent years, it is evident that new research offers promising prospects for treating TBI (24).

Given the severity of TBI and the unique characteristics of stem cells, an increasing number of scholars have directed their attention toward exploring TBI and stem cells (25), resulting in a substantial body of literature on this subject. However, while meta-analyses and reviews can provide credible, evidence-based medical findings, these studies often lack comprehensive coverage as they focus on specific aspects. Moreover, they may have integrity and quantitative analysis limitations, which hinder a holistic understanding for researchers in this field (26). In order to overcome these limitations and gain insights into the research landscape, future trends, and dynamic evolution within this domain, we aim to employ bibliometric methodologies in our present study to investigate the current status and frontiers of stem cell applications in traumatic brain injury. Bibliometrics is a quantitative approach for synthesizing multidimensional information within a specific field, utilizing visualization and network technologies to facilitate researchers in rapidly comprehending the research landscape, predicting future research trends, and exploring the dynamic evolution within that particular domain (27, 28). With the development of information technologies, researchers have extensively applied bibliometric tools such as CiteSpace (29), VoSviewer (30), and R package “bibliometrix” (31) in neurology-related fields, including stroke (32), spinal trauma (33), neuro-oncology (34), and seizures (35).

## 2 Methods

### 2.1 Data source

The Web of Science Core Collection (WoSCC, Clarivate Analytics, Philadelphia, PA, USA) has been extensively employed in bibliometric studies due to its highly comprehensive and authoritative database platform for accessing global academic information. It provides basic information such as title, country/region, institution, author, and author keywords and includes references. Therefore, it is considered the optimal database for bibliometric research. Journal information, including impact factor (IF) and quartile in category (Q1-Q4), was obtained from the 2022 Journal Citation Reports (Clarivate Analytics, Philadelphia, PA, USA).

### 2.2 Search strategy and data extraction

**Figure 1:**
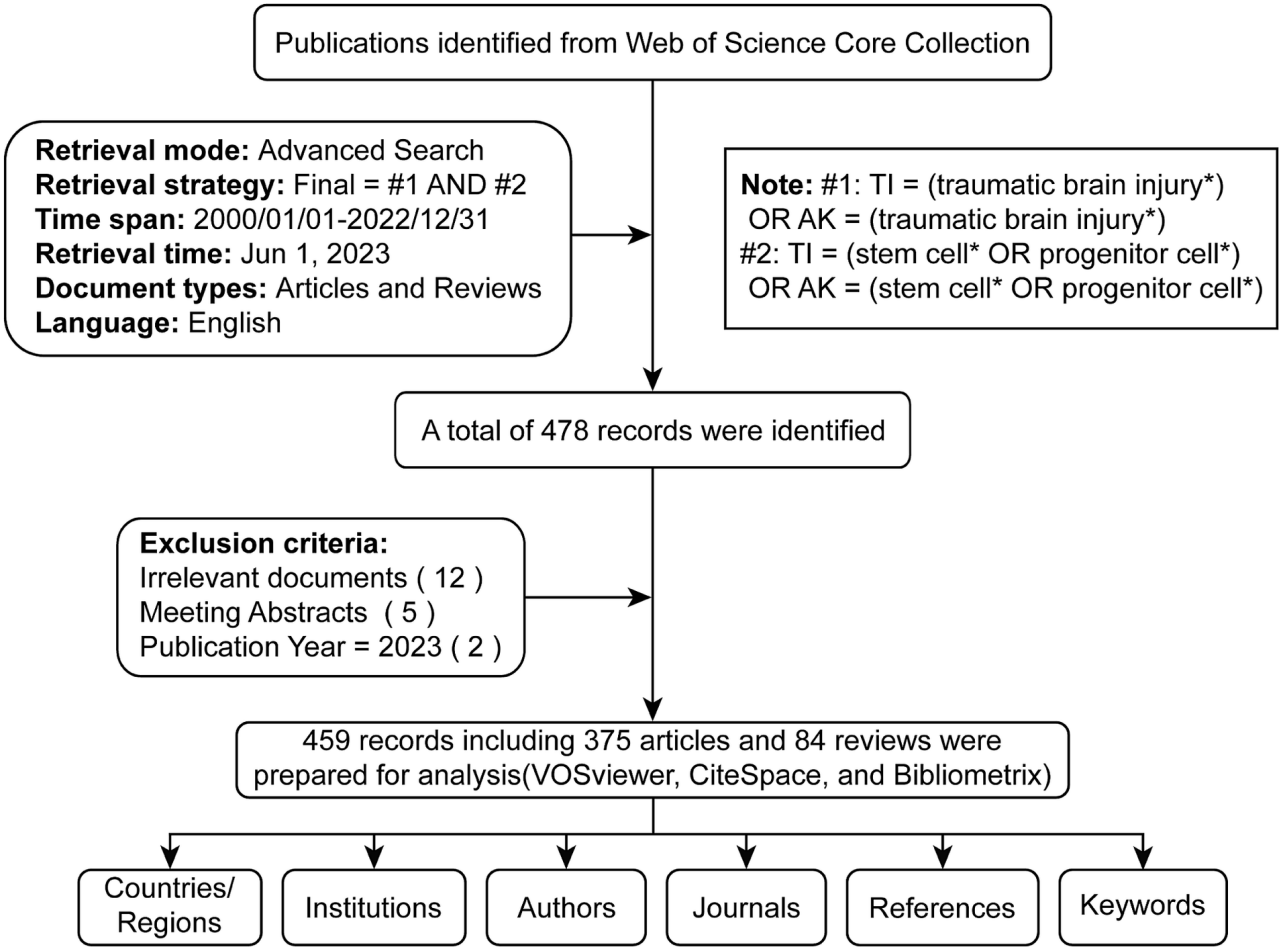
Publications screening flowchart.

Two authors (TD and RD) conducted a comprehensive online search to avoid bias due to potential daily updates in the running database. Based on the title (TI) and author keyword (AK), we identified potentially relevant publications using the following search formula: #1: TI = (traumatic brain injury*) OR AK = (traumatic brain injury*); #2: TI = (stem cell* OR progenitor cell*) OR AK = (stem cell* OR progenitor cell*); Final dataset: [#1 AND #2]. Only English-language literature classified as “articles” or “reviews” from January 1st, 2000, to December 31st, 2022, was included. A total of 478 articles were initially acquired as potential candidates. Subsequently, to check the relevance of the literature, two independent researchers (YW and YC) manually examined the paper titles, abstracts, and full text to exclude any irrelevant content related to the study topic (including disease type, research purposes, animal models, interventions, cell types, outcome indexes). Any divergent viewpoints were resolved through discussions with a third investigator (TD or RD). Eighteen invalid records were excluded, including irrelevant literature, meeting abstracts, and advance access. We obtained 459 valid documents (Supplementary Data 1), exported in TXT format as ‘full record and cited references’ for further analysis (Figure 1).

### 2.3 Validation

The search query was validated using three criteria. The first criterion was the top 100 cited documents, which were reviewed by checking the titles and abstracts and consulting two external colleagues in the field of health sciences in case of doubt. The reviewers had to judge the presence of false-positive results. The absence of false-positive results was used as an indicator of validity. The second validity criterion was the relevancy of the top 20 journals to TBI and stem cells. In the final search query, the top 20 active journals were in neurology, molecular, and biology. The third criterion was to investigate two of the most active authors obtained by the search strategy. In the present study, Cox Charles S. Jr. and Zhang Jianning were among the top active authors, with 21 and 15 publications, respectively. To confirm that the research strategy was comprehensive and retrieved all possible data, the research activity of the two researchers was investigated and counted using author search methodology in WoSCC. The result of this approach showed that the two researchers have research output similar to that produced by the search strategy, which indicates the high validity of the search strategy (36).

### 2.4 Statistical analysis

We conducted statistical analysis and curve fitting procedures using Microsoft Office Excel 2021 (Microsoft, Redmond, Washington, USA) software. Excel was also used to calculate the annual number of publications and citations. Various functions, including exponential, linear, logarithmic, and polynomial, were employed for curve-fitting purposes. We chose the best-fit model based on correlation coefficient value (R^2^). Applying a specific formula determined the growth rate of publications over time: Growth rate = [(number of publications in the last year ÷ number of publications in the first year)^1/(last year − first year) ^− 1] × 100%.

### 2.5 Bibliometric analysis and visualization

VOSviewer (version 1.6.19) is a bibliometric software published by Professor van Eck and Waltman that was used to explore collaboration networks (30, 37). Different nodes indicated different items, such as authors, countries, institutions, journals, and keywords, with the node size reflecting the corresponding number of publications, citations, or occurrences. The links between nodes represented the co-authorship, co-citation, or co-occurrence associations between nodes. The color of the nodes and lines indicated different clusters or corresponding average appearing year (AAY). Line thickness between nodes reflects the level of collaboration or co-citation among them (38).

CiteSpace (version 6.1.R4) was published by Professor Chen Chaomei for bibliometric analysis and visualization (29). In our study, we used Citespace to perform a cooperation analysis of institutions, analyze the co-citation relationship of authors, conduct a dual-map overlay of scientific journals, perform a co-citation analysis of references, identify the top 20 references and 15 keywords with the most robust citation bursts. In the network maps, the nodes represent various items such as institutions, authors, and references. The node size and color rings indicate the number of these items and different years, respectively. The lines between the nodes reflect the co-citation relationships of items. CiteSpace parameters included were as follows: time span (2001–2020), years per slice (1), selection criteria (g-index: *k* = 25), and pruning (minimum spanning tree, pruning sliced networkshttps://www.bibliometrix.org).

For thematic evolution analysis and construction of a global distribution network, the R package “bibliometrix” (version 3.2.1)^1^ was employed (31).

## 3 Results

### 3.1 Publication volume and trends

**Figure 2:**
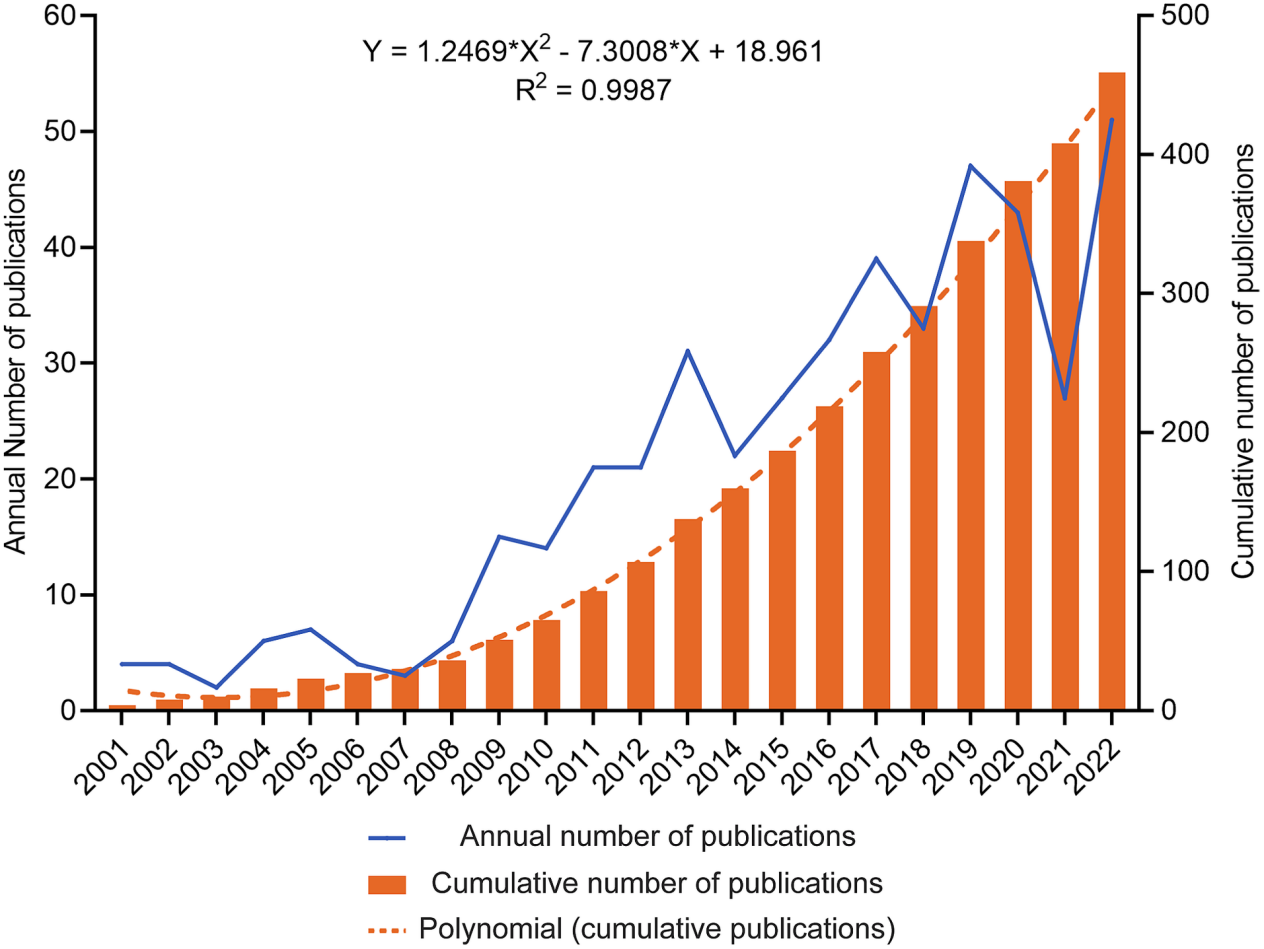
Trends in the annual publication of TBI and stem cells were analyzed. The blue line depicts the developmental trend of the yearly publication count, while the orange columns represent the cumulative number of publications. An orange dashed line depicts a fitting curve for the cumulative number of publications.

From 2001 to 2022, 459 TBI and stem cell articles were published, including 375 articles (81.7%) and 84 reviews (18.3%). Figure 2 shows the gradual growth trend of annual publications, from 4 papers in 2000 to 51 in 2022. The average growth rate of the number of publications is 12.9%, the index function *Y* = 1.2469*X^2^–7.3008*X + 18.961 (Y is the annual cumulative publications, X is the year minus 2000, *R*^2^ = 0.9987) is derived to evaluate further the changing trend of the cumulative number of publications. According to the fitting curve, the number of papers published in 2030 is expected to be close to 100, and the cumulative number of publications will exceed 1,000.

### 3.2 Country/region and institutional analysis

**Figure 3:**
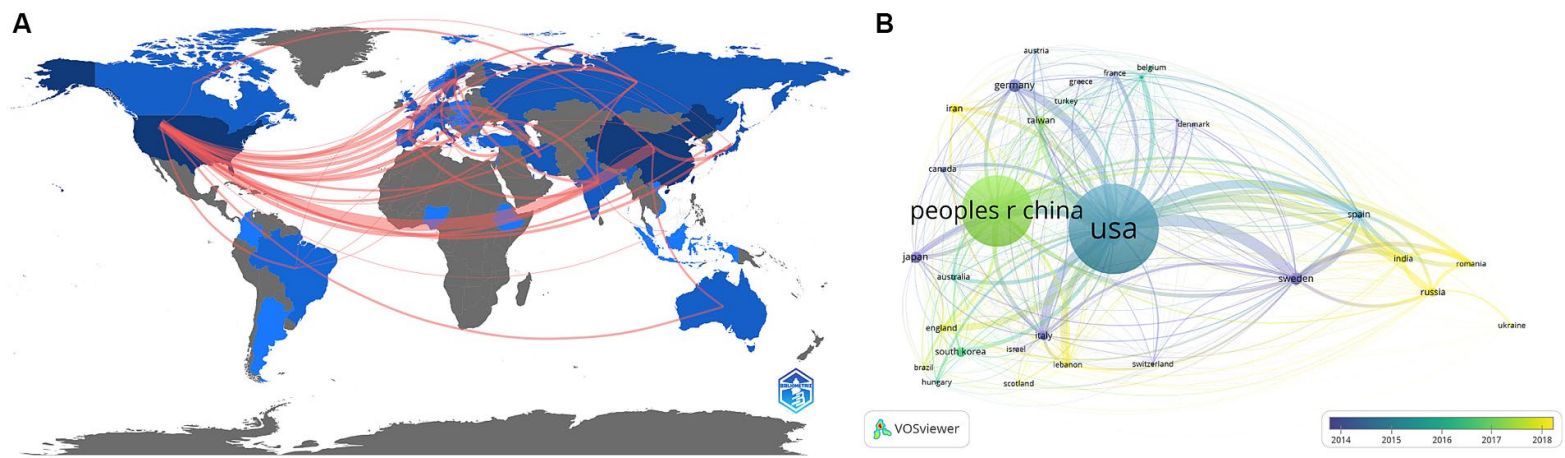
**(A)** A geographical distribution map illustrating the global research contributions in the field. The intensity of the blue color indicates the number of publications published in each country, while gray represents countries with no publications. **(B)** A co-authorship network among countries/regions over time is visualized using VOSviewer. Each node represents a country/region, and the connecting lines between nodes indicate their collaborative relationships. The thickness of the line reflects the strength of cooperation. At the same time, each node’s color corresponds to its position on the timeline and indicates when it engaged in cooperative activities with other nodes.

Forty-five countries/regions contributed to the literature. Figure 3A shows a world map depicting countries’ collaboration and contribution. According to the color gradient and the top 10 countries/regions in Table 1 (Part A), researchers from North America, Eastern Asia, and Europe published most articles. Specifically, The United States has the largest number of publications (*n* = 172, 37.5%), followed by China (*n* = 158, 34.4%). Subsequently, a co-authorship network among countries/regions over time is visualized using VOSviewer (Figure 3B), we also observed close cooperation between many countries/regions, especially between China and the United States, Canada, Japan, England, and Taiwan. Different colors marked nodes representing countries/regions based on the average appearing year (AAY). According to the color gradient in the lower right corner, The United States was given the bluish color, indicating that most researchers in the country were relatively early players. In contrast, China and many countries labeled in green are relatively new participants in TBI and stem cell research.

**Table 1:** The top 10 countries and institutions with the most publications in the field on TBI and stem cells.

Rank	Part A			Part B	
	Country/Region	Count	Percent	Institutions	Count
1	United States	172	37.5%	Tianjin Med Univ (China)	22
2	China	158	34.4%	Univ S Florida (United States)	17
3	Japan	13	2.8%	Univ Miami (United States)	13
4	Korea	13	2.8%	Univ Texas Houston (United States)	13
5	Germany	12	2.6%	henry ford hosp (United States)	11
6	Sweden	12	2.6%	Zhengzhou Univ (China)	10
7	Russia	11	2.4%	Uppsala Univ (Sweden)	10
8	Iran	8	1.7%	Virginia Commonwealth Univ (United States)	10
9	Canada	5	1.1%	Sichuan Univ (China)	10
10	Italy	5	1.1%	Univ Calif San Francisco (United States)	10

**Figure 4:**
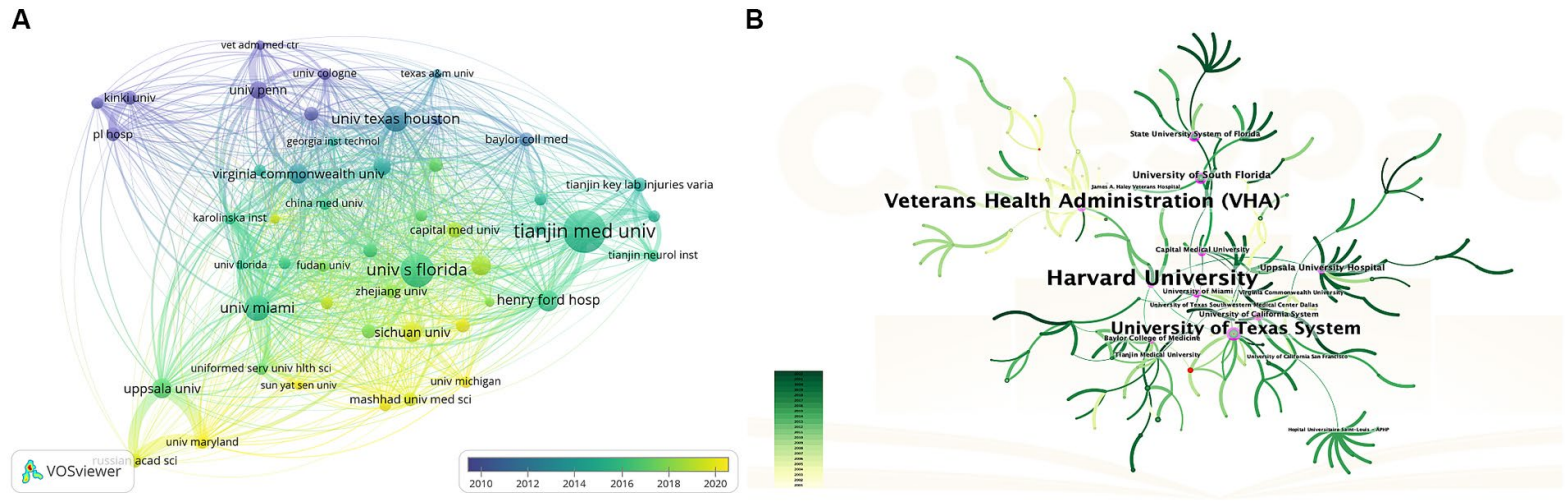
**(A)** The overlay visualization map of Institution co-authorship analysis conducted by VOSviewer. **(B)** Network visualization map of institutional collaborations generated by CiteSpace. A node represents an institution, each line represents the strength of the cooperation relationship between two institutions, Generally, nodes with a BC value of more than 0.1 occupy pivotal positions connecting many nodes and are identified as hubs of nodes displayed in purple rings.

Regarding the analysis of organizations, 637 institutions contributed to this area. Table 1 (Part B) lists the top 10 institutions with the most publications, they are located in the United States (*n* = 6), China (*n* = 3), and Sweden (*n* = 1), these institutions collectively published 125 articles, accounting for 27.2% of all articles. Specifically, Tianjin Medical University emerged as the most prolific institution (22, 4.8%), followed by the University of South Florida (17, 3.70%), University of Miami (13, 2.8%), University of Texas Houston (13, 2.8%), and Henry Ford Hospital (11, 2.4%). We analyzed institutional cooperation networks based on co-authorship using VOSviewer (Figure 4A), 49 institutions with more than five publications were identified. According to the color gradient in the lower right corner, institutions such as Sichuan University, Mashhad University of Medical Sciences, University of Michigan, etc., were given a yellow color with the later AAY values. Conversely, the University of Texas Houston, the University of Pennsylvania, and Kinki University were given a bluish color with the prior AAY values. However, co-citation analysis revealed that despite the high publication volume of the institutions mentioned above, their collaboration with other institutions was relatively limited. CiteSpace analyzed institutional cooperation based on citation relationships (Figure 4B). Harvard University has the highest centrality with a betweenness centrality (BC) value of 0.32, followed closely by Veterans Health Administration (BC = 0.30), indicating their significant influence in this field (institutions with a purple outer circle represent having a larger BC). The top 10 institutions based on betweenness centrality are listed in Supplementary Table 1.

### 3.3 Most prolific and cited journals

**Figure 5:**
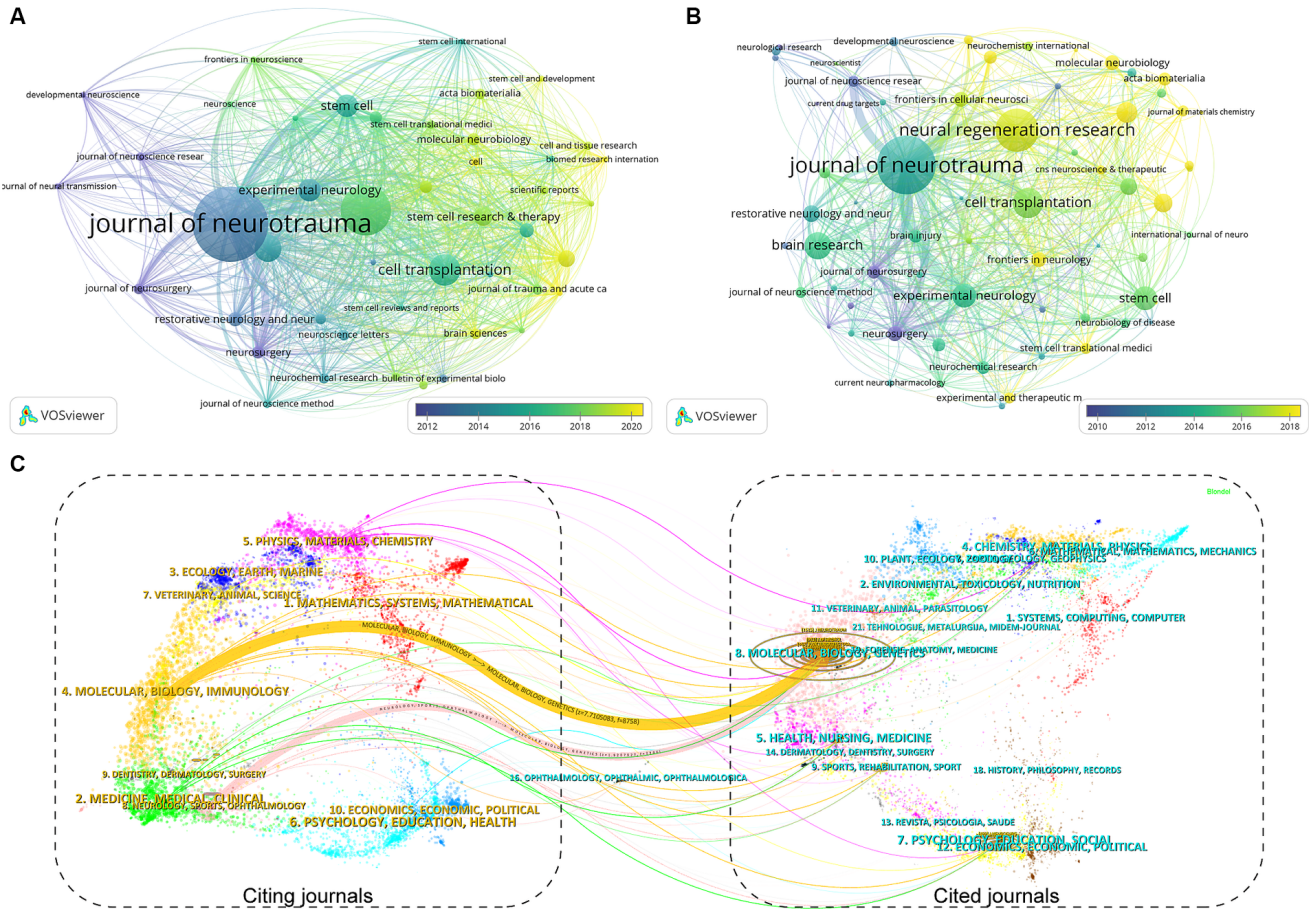
The visualization of journal coupling map **(A)** and citation map **(B)** generated by Vosviewer. **(C)** The dual-map overlay of academic journals in the field of TBI and stem cell research generated by CiteSpace.

One hundred eighty-seven journals published articles related to stem cells and TBI. We summarized the journal information of the top 15 most prolific and cited (Table 2). The *Journal of Neurotrauma* published the most papers (*n* = 36, 7.8%), followed by *Neural Regeneration Research* (*n* = 24, 5.2%), *Cell Transplantation* (*n* = 15, 3.3%), and *Brain Research* (*n* = 13, 2.8%). Among the top 15 journals, *Acta Biomaterialia* (IF = 10.633) is the journal with the highest impact factor, followed by the *Journal of Neuroinflammation* (IF = 9.587) and *Stem Cell Research & Therapy* (IF = 8.079). From Table 2, more than half of the journals belong to Q1. Six publishers were from the USA, three from the UK, and the others from Switzerland, the Netherlands, and India. Subsequently, we screened 46 journals with more than three publications and created the journal coupling map using VOSviewer. The *Journal of Neurotrauma* (IF = 4.869) has active coupling relationships with *Neural Regeneration Research* (IF = 6.058), *Journal of Neurosurgery* (IF = 5.526), and *Stem Cells* (IF = 5.845), etc. (Figure 5A), according to the nodes that represent journals marked by different colors, we can also find the AAY of each periodical.

**Table 2:** The top 15 most prolific journals and cited journals.

Rank	Journal	Articles (percent)	IF	JCR	Country	Cited Journal	Citation	IF	JCR
1	Journal of Neurotrauma	36 (7.84)	4.869	Q2	United States	Journal of Neurotrauma	1,665	4.869	Q2
2	Neural Regeneration Research	24 (5.23)	6.058	Q2	China	Journal of Neuroinflammation	630	9.587	Q1
3	Cell Transplantation	15 (3.27)	4.139	Q3	United States	Neurosurgery	601	5.315	Q1
4	Brain Research	13 (2.83)	3.61	Q4	Netherlands	Journal of Neuroscience Research	600	4.433	Q2
5	Experimental Neurology	11 (2.40)	5.62	Q1	United States	Experimental Neurology	515	5.62	Q1
6	Stem Cells	11 (2.40)	5.845	Q1	United States	Journal of Neurosurgery	496	5.526	Q1
7	Stem Cell Research & Therapy	9 (1.96)	8.079	Q1	England	Brain Research	437	3.61	Q4
8	International Journal of Molecular Sciences	8 (1.74)	6.208	Q3	Switzerland	Stem Cells	427	5.845	Q1
9	Journal of Neuroinflammation	7 (1.53)	9.587	Q1	England	Cell Transplantation	385	4.139	Q1
10	Restorative Neurology and Neuroscience	7 (1.53)	2.976	Q2	Netherlands	Neural Regeneration Research	368	4.433	Q3
11	Frontiers in Cellular Neuroscience	6 (1.31)	6.147	Q1	Switzerland	Frontiers in Cellular Neuroscience	324	6.147	Q1
12	Frontiers in Neurology	6 (1.31)	4.086	Q2	Switzerland	Stem Cell Research & Therapy	316	8.079	Q1
13	Molecular Neurobiology	6 (1.31)	5.682	Q2	United States	Journal of Neuroscience	260	6.709	Q1
14	Neurosurgery	6 (1.31)	5.315	Q1	United States	Neurochemistry International	238	4.297	Q2
15	Acta Biomaterialia	5 (1.09)	10.633	Q1	England	Biomaterials	219	15.304	Q1

As for the analysis of cited journals, two-thirds of the journals belong to Q1, five journals have been cited more than 500 times, and the *Journal of Neurotrauma* (*n* = 1,665) was the most cited, followed by the *Journal of Neuroinflammation* (*n* = 630), *Neurosurgery* (*n* = 601), and *Journal of Neuroscience Research* (*n* = 600). Furthermore, the journal with the highest impact factor is *Biomaterials* (IF = 15.304), followed by the *Journal of Neuroinflammation* (IF = 9.587) and *Stem Cell Research & Therapy* (IF = 8.079). The network visualization map of the journal citation analysis was created by filtering journals with less than 50 citations (Figure 5B). *Journal of Neurotrauma* has close citation relationships with *Experimental Neurology*, *Cell transplantation*, *Neurol regeneration research, and Neurosurgery*.

In the WoSCC database, each article was categorized with one or more subject categories. The dual-map overlay of journals represented the disciplinary distribution of journals related to stem cell and TBI research. As depicted in Figure 5C, the citing trajectories were constructed within the dual-map overlay module, where the left side represents clusters of citing journals and the right side represents clusters of cited journals. The yellow and pink lines indicate that literature published in molecular/biology/immunology and neurology/sports/ophthalmology journals often cite literature published in molecular/biology/genetics journals.

### 3.4 Analysis of the influential authors

More than 2,500 authors participated in research on stem cells and TBI. Charles S. Cox, Jr. from The University of Texas Health Science Center at Houston was the author with the most published literature (*n* = 21), followed by Zhang Jianning (*n* = 15), and Zhang Sai (*n* = 8), Zhang Jianning and Zhang Sai came from the same university (Tianjin Medical University) (Table 3).

**Table 3:** The top 10 authors and co-cited authors involved in research on TBI and stem cell.

Rank	Highly published authors	Count	Highly cited authors	Citations	Co-cited authors	Citations
1	Cox Charles S. Jr.	21	Cox Charles S. Jr.	1,190	Asim Mahmood	297
2	Zhang Jianning	15	Mcintosh Tk	674	Sharma Hari Shanker	151
3	Zhang Sai	8	Jimenez Fernando	655	Xiong, Ye	139
4	Jimenez Fernando	8	Harting Matthew T.	575	Lu, Dunyue	115
5	Xue Hasen	8	dash pramod k.	569	Harting Matthew T.	100
6	Mcintosh Tk	8	Xue Hasen	490	Sun, Dong	99
7	Walker Peter a.	7	Walker Peter a.	448	Walker, Peter a.	98
8	Harting Matthew t.	7	shah shinil k.	439	borlongan cesar v.	79
9	borlongan cesar v.	7	pati shibani	407	Riess, Peter	75
10	shah shinil k.	7	Zhang Jianning	274	Cox Charles s. jr.	75

**Figure 6:**
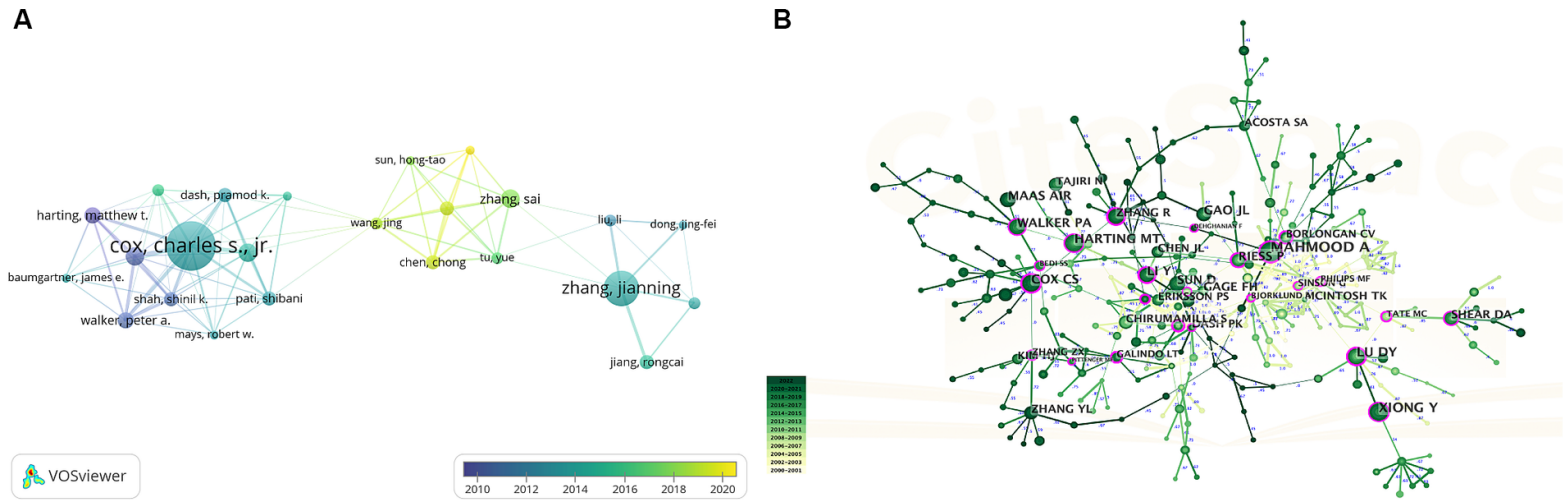
**(A)** Overlay visualization map of author co-authorship analysis generated by VOSviewer. **(B)** Visualization map of author co-citation analysis by using CiteSpace software.

In Figure 6A, we generated an overlay visualization map of author co-authorship analysis by VOSviewer software. Several researcher clusters have been produced, and each cluster is radiated by a core author such as Cox Charles s. jr., Zhang Jianning, and Zhang Sai. There are only a few connections between different clusters, which indicates that cooperation and communication have not been well developed in this area. In addition, we can also know the AAY of each author based on the color in the lower right corner, we can find that clusters centered around Zhang Sai seem to be relatively young researchers in this field.

The co-citation relationship refers to two authors/works of literature appearing together in the reference list of a third document, the author co-citation analysis is often used to reveal the key authors in a co-citation network of a particular field. Generally, frequently cited authors are thought to have a more significant influence than those less cited. Authors who are co-cited are likely to focus on similar research areas. As shown in Table 3 five authors were co-cited more than 100 times. Asim Mahmood is the most co-cited author (*n* = 297), followed by Sharma Hari Shanker (*n* = 151) and Xiong Ye (*n* = 139). As we can see in Figure 6B, highly co-cited authors occupied key locations connecting many nodes, the hubs of nodes marked with purple rings.

### 3.5 Cited references and co-cited references

Although there remains some controversy on the value of citation rates (39, 40), the number of citations remains a crucial indicator of scholarly impact (41), as highly cited papers typically reflect a high degree of academic attention and represent the research hotspots in a field. Table 4 lists the top 10 most cited papers, over half were cited at least 200 times. These studies were published from 2001 to 2017, and the majority of articles were published in neurosurgery journals such as the *Journal of Neuroscience Research*, *Journal of Neuroinflammation*, *Journal of Neurotrauma*, and *Neurosurgery*. Nine of them are original articles, and one is a systematic review. Specifically, the most cited paper in this area is the article “Enhanced Neurogenesis in the Rodent Hippocampus Following Traumatic Brain Injury” published by P.K. Dash (42), cited 344 times. The second and third most cited papers were published by Run Zhang et al. (43) and Steven G. Kernie et al. (44), which were animal model studies about the immunomodulatory effect of MSC transplantation and proliferation of neuron and astrocytic after TBI. In summary, the predominant topics covered by these 10 publications include the pharmacologic studies of stem cell or cell-derived exosomes after TBI (43, 45–49), molecular mechanism research underlying TBI-associated neurogenesis (42, 44, 50), and review regarding stem cell therapy for the immune response after TBI (51).

**Table 4:** Top 10 cited references of publications in TBI and stem cells.

Rank	Title	Journal	First author	Publication year	Citations
1	Enhanced neurogenesis in the rodent hippocampus following traumatic brain injury	Journal of Neuroscience Research	P.K. Dash	2001	344
2	Anti-inflammatory and immunomodulatory mechanisms of mesenchymal stem cell transplantation in experimental traumatic brain injury	Journal of Neuroinflammation	Run Zhang	2013	263
3	Brain Remodeling Due to Neuronal and Astrocytic Proliferation After Controlled Cortical Injury in Mice	Journal of Neuroscience Research	Steven G. Kernie	2001	248
4	Traumatic Brain Injury Induced Cell Proliferation in the Adult Mammalian Central Nervous System	Journal of Neurotrauma	S. Chirumamilla	2002	238
5	Systemic administration of cell-free exosomes generated by human bone marrow derived mesenchymal stem cells cultured under 2D and 3D conditions improves functional recovery in rats after traumatic brain injury	Neurochemistry Internationa	Yanlu Zhang	2017	220
6	Treatment of Traumatic Brain Injury in Female Rats with Intravenous Administration of Bone Marrow Stromal Cells	Neurosurgery	Asim Mahmood	2001	203
7	Intravenous mesenchymal stem cell therapy for traumatic brain injury	Journal of Neurosurgery	Matthew T. Harting	2009	199
8	Hypoxic preconditioning enhances the therapeutic potential of the secretome from cultured human mesenchymal stem cells in experimental traumatic brain injury	Clinical Science	Ching-Ping Chang	2013	194
9	Transplanted Neural Stem Cells Survive, Differentiate, and Improve Neurological Motor Function after Experimental Traumatic Brain Injury	Neurosurgery	Peter Riess	2002	190
10	New perspectives on central and peripheral immune responses to acute traumatic brain injury	Journal of Neuroinflammation	Mahasweta Das	2012	170

**Figure 7:**
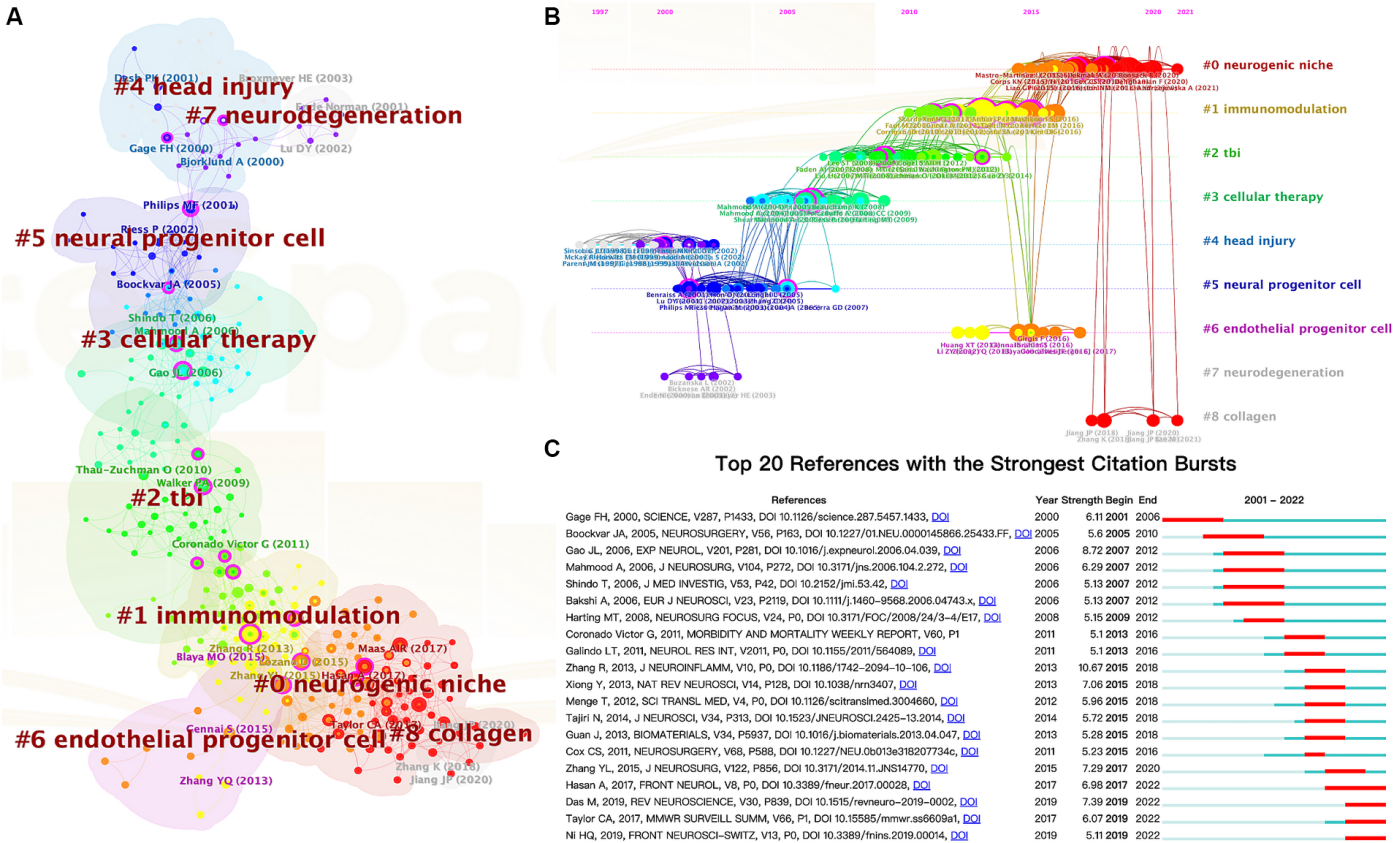
The cluster view map **(A)** and timeline view map **(B)** of reference co-citation analysis were generated by CiteSpace. **(C)** Top 20 references with the most robust citation bursts.

In addition, co-citation analysis of reference is a practical approach for evaluating research hotspots and tracking advancements in the field. The literature network of co-citation was divided into 9 clusters using CiteSpace (Figure 7A), with each cluster focusing on similar research topics. To assess the significance of the cluster structure, we evaluated two parameters: modularity value (*Q*-value) and mean silhouette value (*S*-value). Our findings indicate that the *Q*-value (0.7127) exceeds 0.3, suggesting a reasonable network, while the *S*-value (0.9042) exceeds 0.7, indicating high homogeneity within clusters (52). The largest cluster identified was labeled as “#0 neurogenic niche,” followed by “#1immunomodulation,” “#2 TBI,” and “#3 cellular therapy.”

Additionally, we employed a timeline view of co-cited references to conduct a visual analysis by integrating clustering and time-slicing techniques (Figure 7B). The arrangement of cluster labels is based on their appearance order after clustering, providing insights into topic distribution, trends, and correlation over time. Each horizontal line represents a collection of clustered references to which they belong. The closest clusters on the timeline were “#0 neurogenic niche,” “#1 immunomodulation,” “#6 endothelial progenitor cells,” and “#8 collagen.”

Moreover, CiteSpace can identify burst detection for highly cited references, a widely employed method for discerning actively researched hotspots or topics over time. Figure 7C shows the top 20 references with significant citation bursts highlighted in red, corresponding to specific time intervals denoted by blue lines. Notably, “Das M, 2019, REV NEUROSCIENCE, V30, P839, DOI 10.1515/revenue-2019-0002” (2019-2022, strength = 7.39), “Taylor CA, 2017, MMWR SURVEILL SUMM, V66, P1, DOI 10.15585/mmwr. ss6609a1” (2019-2022, strength = 6.07), and “Ni HQ, 2019, FRONT NEUROSCI-SWITZ, V13, P0, DOI 10.3389/fnins.2019.00014” (2019-2022, strength = 5.11) represent recently published highly influential literature.

### 3.6 Analysis of keywords

**Figure 8:**
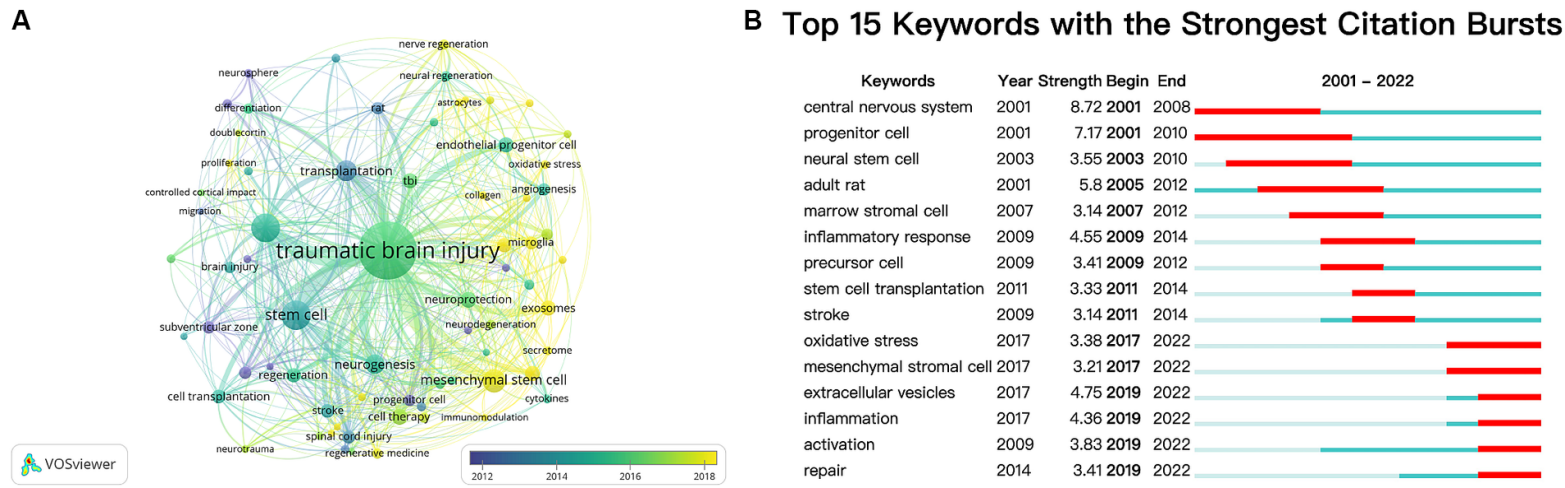
**(A)** Overlay visualization map of keywords analysis based on the VOSviewer. The node size is proportional to the sum of occurrence times. The color of each node implies the average appearing year according to the color gradient in the lower right corner. Bluish represents the keywords that appeared relatively earlier, and yellow reflects the recent occurrence. **(B)** Top 15 keywords with the strongest citation bursts.

Besides references, keywords can also represent a specific topic’s core themes and primary content (53). After aggregating keywords with the same connotation, we analyzed the keywords that appeared more than five times in all literature using VOSviewer (Figure 8A). A total of 70 keywords were identified, and the top five most frequently occurring keywords were traumatic brain injury (349 times), stem cell (86 times), neural stem cell (84 times), mesenchymal stem cell (52 times), and transplantation (43 times).

Meanwhile, we employed CiteSpace’s burst detection algorithm to identify keyword bursts. Figure 8B presents the top 15 keywords with the most vigorous bursts. The most prominent keyword was “central nervous system” (strength 8.72), followed by “progenitor cell” (strength 7.17). After 2019, “extracellular vesicles,” “inflammation,” “activation,” and “repair” were keywords with citation bursts.

## 4 Discussion

### 4.1 General information

For this study, we conducted a comprehensive search of articles on TBI and stem cells in the Web of Science databases from 2000 to 2022. Our analysis included 459 English papers affiliated with 637 institutions across 45 countries/regions. While there was some variation in publication numbers over the years, an overall upward trend was observed, reaching its peak in 2022, with 51 publications accounting for approximately 11.11% of the total corpus. These findings indicate a growing research interest in investigating the relationship between TBI and stem cells (54).

China and the United States have emerged as significant contributors to these publications, collectively accounting for over 70% of the total publications. The distribution of institutions mirrors this pattern, with six out of the top 10 institutions based in the United States and three in China. Despite the significant contributions made by researchers from Asian countries to paper publications, collaborative networks have yet to be established among research institutions in these regions (Figure 4). It is imperative to remove academic barriers and enhance cooperation and communication between diverse research institutions or groups.

The *Journal of Neurotrauma* (*n* = 36, 7.8%) ranked first in total publications, focusing on the traumatic injury of the central and peripheral nervous system and encompassing fundamental biology and clinical trials. It was followed by *Neural Regeneration Research* (*n* = 24, 5.2%) and *Cell Transplantation* (*n* = 15, 3.3%), indicating their interest in TBI and stem cell research articles. These findings will assist future scholars in selecting appropriate journals for submitting their contributions. Notably, the top 10 journals published only 141 papers, accounting for merely 30.72% of all papers, this suggests further potential for the impact of papers. Furthermore, most of these influential journals are located in Western Europe and North America; China is represented by only one journal, with no representation from Japan or Korea. This situation highlights the need for Asian countries to enhance the development of international journals and augment their academic influence further, particularly in China, where the number of individuals affected by TBI surpasses that of most nations, resulting in a substantial burden on society and families (55). It is commendable that the Chinese government has invested considerable resources in the construction of international journals, with multiple incentive measures in recent years (56).

Charles S. Cox Jr. has published 21 papers and been cited 1,190 times, establishing himself as one of the most prolific scholars with noteworthy achievements. Through a comprehensive analysis of publication frequency, citation impact, and co-citation patterns, we identified that Charles S. Cox Jr., Walker Peter A., and Harting Matthew T. were the scholars who appeared in all three indicators simultaneously, suggesting that they are accomplished authors in this field. Notably, three researchers are all from the University of Texas Health Science Center Houston, this team is known for its essential contribution to the research field (57), particularly concerning cell therapy safety for individuals affected by TBI or SCI (58). They would make excellent potential collaborators for researchers.

Furthermore, it is worth highlighting the lack of close cooperation among scholars in this domain. Out of 78 researchers who have published more than four papers, only approximately 30% could establish co-authorship networks (Figure 6A), indicating that collaboration was primarily confined to specific teams. Scholars from various institutions should strive for technological innovation and breakthroughs within research activities by strengthening cooperation, including personnel exchange and study, research progress communication, and sharing platforms and data.

### 4.2 Development of stem cell research in TBI

The study of TBI and stem cells continuously evolves, with discoveries and insights emerging regularly. We have created a knowledge map of the research in this field through citation and co-citation analysis. In general, research can be categorized into two main areas. The first area focuses on the endogenous stem cells activated by TBI. This research aims to investigate the mechanisms underlying their activation and enhance their efficiency in differentiating into mature neurons. The second area involves using exogenous stem cells for treating TBI, which stems from the overall advancements in cell therapy. Investigating diverse cell types, identifying specific components that facilitate recovery, and employing novel techniques to enhance cell retention and prognosis constitute the primary focus of studies. Ongoing studies continue to shed new light on the potential of both endogenous and exogenous stem cells in improving outcomes for TBI patients.

#### 4.2.1 Endogenous stem cells

In 2001, P.K. Ash demonstrated that TBI induces a significant upregulation in neurogenesis within the dentate gyrus region, with peak production observed between days 3 and 7 post-injury, returning to baseline levels by day 14 (42), as a result of this groundbreaking study, Ash has received the highest number of citations in the field. In parallel, Steven G. Kernie discovered that neural proliferation plays a crucial role in remodeling after TBI and proposed a mechanism for explaining how functional recovery can be sustained over an extended period following such injuries (44). One year later, Chirumamilla, S. observed a significant rise in the overall number of proliferating cells within both the subventricular zone (SVZ) and hippocampus just 48 h after TBI; however, differentiation had not yet commenced among these proliferating cells within SVZ. Additionally, notable growth was explicitly seen in immature astrocytes and activated microglia but not neurons within the hippocampus region (50). These three articles are cornerstones in the research field of endogenous nerve regeneration after TBI, based on these articles’ findings, scholars aim to promote outcomes of TBI. Given the inherent limitations of innate recovery capacity, it is necessary to enhance this endogenous process through exogenous means, diverse categories of growth factors and pharmaceutical agents can potentially augment neurogenesis (59). Brain-derived neurotrophic factor (BDNF), bFGF, and EGF can enhance TBI-induced cell proliferation in the hippocampus and the SVZ (60–62). Neurotrophic factors have been widely investigated for their role in promoting NSC survival, proliferation, and differentiation, these findings suggest that neurotrophic factors hold promise as potential therapeutic agents for enhancing endogenous NSC regeneration after TBI.

Additionally, neuroinflammation plays a crucial role in the pathophysiology of TBI, and emerging evidence suggests its modulation of endogenous repair mechanisms (63). Suppression of inflammation through progranulin administration protects hippocampal neurogenesis (64), while hyperbaric oxygen therapy may enhance outcomes of TBI in rats by inhibiting inflammation and gliosis (65). The extracellular matrix (ECM) also regulates the behavior of NSC, where chondroitin sulfate proteoglycans (CSPGs) facilitate endogenous NSC repair following injury through ECM manipulation (66). Furthermore, electrical stimulation promoted anti-inflammatory phenotypes of microglia and increased the population of NSCs, thereby regulating neuroinflammation and enhancing neuroregeneration (67). These findings present a novel opportunity to facilitate endogenous NSC regeneration. Future research should prioritize elucidating the underlying mechanisms governing endogenous NSC behavior and identifying innovative targets for therapeutic intervention.

#### 4.2.2 Exogenous stem cells

While endogenous NSC regeneration holds promise, transplantation strategies involving exogenous NSCs have also been explored. Exogenous stem cells exert their therapeutic effects through various mechanisms. Transplanted stem cells not only can directly differentiate into neuronal and glial cell types, thereby replacing damaged cells within the injured brain (49), but also secrete trophic factors, such as growth factors and cytokines, which promote endogenous repair mechanisms, enhance neuroplasticity, and reduce inflammation (68).

In 2001, Asim Mahmood and colleagues experimented with using marrow stromal cells to treat TBI, they injected cells through the tail vein of rats, resulting in a significant reduction in motor and neurological deficits; the transplanted cells exhibited a preference for implanting themselves into the damaged brain tissue and expressed markers indicative of neurons (NeuN) and astrocytes (GFAP) (46). One year later, Peter Riess and colleagues utilized stereotactic injection to transplant murine neural stem cells (NSCs) into mice with CCI-induced brain injuries; the study demonstrated that the transplanted NSCs were capable of surviving within the injured brain, differentiating into either neurons or glial cells, and subsequent a reduction in motor dysfunction caused by TBI. These pioneering studies signify the inception of exogenous stem cell therapy for TBI. Inspired researchers were dedicated to further stem cell research as a potential therapeutic strategy for various diseases (69, 70). In numerous preclinical studies and early clinical trials, intravenous infusion is a cell delivery method (71). However, Charles S. Cox Jr. and colleagues observed that the majority of MSCs localized primarily in the lungs within 48 h after infusion, only 0.0005% reached the cerebral parenchyma and remained there over time (47), MSCs were largely undetectable in brain tissue after 2 weeks, they described this phenomenon as the “pulmonary first-pass effect,” which may impede therapeutic efficacy significantly. Further optimization of strategies for stem cell transplantation is imperative to ensure successful clinical translation. Considerations encompass the timing and delivery routes of transplantation, optimal cell dosage, and immunological compatibility.

Scholars have conducted additional studies to gain deeper insights into the beneficial effects of cell therapy for traumatic brain injury (TBI). In 2012, Mahasweta Das et al. published a review focusing on various effector cells, cytokines, and signaling pathways involved in the pathophysiology of TBI (51); they also discussed the immunoreaction observed after stem cell transplantation. One year later, Run Zhang and Yi Liu discovered that MSCs can regulate inflammation-related immune cells and cytokines during brain inflammatory responses caused by TBI (43). During this period, researchers have discovered a paracrine mechanism mediated by stem cell releasing factor that plays a crucial role in repairing brain injuries following stem cell mobilization (72). Ching-Ping Chang and colleagues found that MSCs secrete bioactive factors such as HGF and VEGF, which stimulate neurogenesis and improve the prognosis in rat models of TBI (48). However, MSC therapy has a few drawbacks, including tumor formation, which can be avoided using MSC-derived exosomes (73). Yanlu Zhang demonstrated the efficacy of MSC-generated exosomes in enhancing functional recovery by stimulating angiogenesis and neurogenesis (74). Exosomes derived from bone marrow mesenchymal stem cells also potentially mitigate early inflammatory response after TBI (75). Combining exosome therapy with hydrogels for traumatic brain injury repair by promoting angiogenesis and neurogenesis (76), this combination therapy approach holds promise for optimizing exosome delivery and creating a conducive microenvironment for tissue repair in TBI.

Collectively, these highly cited and co-cited articles provide valuable insights into the current understanding of neuroregeneration, the exploration and advancement of cell therapy, and the therapeutic potential of stem cell-derived exosomes. Investigating the pathophysiological changes occurring in stem cells following TBI can enhance our comprehension of factors influencing nerve regeneration. Compared to conventional drugs or surgery, both stem cells and their derived exosomes offer significant advantages for treatment. Therefore, studying strategies involving stem cells for TBI treatment holds immense practical value in promoting nerve regeneration.

### 4.3 Research hotspots and keywords

To investigate and elucidate the hotspots of TBI and stem cell research further, we did a citation clustering analysis using CiteSpace. As depicted in Figures 7A,B, initial studies focused on understanding the pathophysiology, mechanism underlying neuroregeneration, and experimental stem cell therapy after TBI, including labels “#4 head injury,” “#7 neurodegeneration,” “#5 neural progenitor cell,” and “#3 cellular therapy.” However, current research is centered around exploring immunomodulatory aspects, microenvironments associated with stem cells, and enhancing therapeutic efficacy through combination with biomaterials, such as “#1 immunomodulation,” “#0 neurogenic niche,” and “#8 collagen.” Additionally, VOSviewer was employed to generate a visualization map (Figure 8A), which effectively integrates frequently occurring keywords with their corresponding average appearing year (AAY). The AAY for keywords such as “exosomes,” “neuroinflammation,” and “microglia” indicates that these topics have recently gained attention and hold the potential to become prominent areas of research. Furthermore, utilizing CiteSpace’s burst keyword analysis (Figure 8B), we identified emerging hotspots in the field, revealing an upsurge in citations related to “extracellular vesicles” and “inflammation” during the period from 2019 to 2022. As we all know, exosomes are a specific type of extracellular vesicle (77), while microglia play a crucial role in neuroinflammation (78, 79). Therefore, both methods highlight “exosomes” and “neuroinflammation” as candidates with significant potential to emerge as research hotspots.

In recent decades, significant progress has been made in the research of TBI and the potential application of stem cells in the therapeutic intervention (80). Stem cell-based therapy has emerged as a promising approach for addressing injuries and disorders associated with the central nervous system. Investigations into the utilization of stem/progenitor cells for the treatment of brain injury (47, 73), spinal cord injury (81, 82), and stroke (83, 84) have yielded positive outcomes in facilitating rehabilitation.

Researchers have been investigating the impact of neurodegeneration after traumatic brain injury (TBI), which can result in enduring cognitive impairments (85) and chronic neurological deficits, including Alzheimer’s and Parkinson’s disease (86). Stem cell therapy is currently receiving attention due to its potential to delay or halt the progression of neurodegenerative disorders following TBI (87). Moreover, stem cells may contribute to immunomodulation by mitigating inflammation (4) and promoting neural repair in response to the inflammatory cascade triggered after TBI (88, 89). The stem cell niche, also known as the microenvironment surrounding stem cells, plays a crucial role in regulating cell fate within specific anatomical locations where stem cells reside (90). The term ‘niche’ denotes the stem-cell microenvironment *in vivo* or *in vitro*. By modulating the neurogenic niche, stem cells can promote neurogenesis and enhance the brain’s regenerative capacity following injury (91). Researchers have explored the potential of collagen-based scaffolds to create a conducive environment for the survival and integration of stem cells within damaged brain tissues (92, 93). This approach holds promise for augmenting the efficacy of TBI treatment using stem cell therapy (94). In summary, the research on TBI and stem cells is rapidly advancing, demonstrating significant progress in applying neural stem cells, immunomodulation techniques, regulating the neurogenic niche, and collagen-based scaffolds. These emerging areas hold immense potential for improving the prognosis of patients with brain injuries and neurodegenerative disorders.

Bibliometric research serves as a method for elucidating the structure and dynamics of scientific knowledge, facilitating the visualization of intricate relationships among knowledge clusters (95). Consequently, comprehending these intricate knowledge connections enables researchers to gain valuable insights into domain-specific trends in knowledge. Our studies suggest that immune regulation and inflammatory responses after TBI and exploring strategies to enhance exosome or biomaterial combinations could significantly contribute to future studies in this field.

### 4.4 Limitations

This study provides an overview of current research, analyzes hot areas of concern, and predicts future trends. However, certain limitations should be acknowledged. Firstly, the literature searches were limited to the WoSCC database, excluding non-English language publications and other databases, which may introduce selection bias. However, as mentioned in previous bibliometric studies, WoSCC is widely used as a reliable database due to its extensive data coverage. Additionally, slight output variations may occur when software applies different parameter settings since no standardized setting is available. Lastly, due to their low citation counts, recent studies published in high-quality journals might not have been included or fully considered during citation and co-citation analyses.

## 5 Conclusion

This study represents the first comprehensive bibliometric analysis of literature on TBI and stem cells from 2000 to 2022. The involvement of stem cells in TBI has gradually gained attention among researchers, as evidenced by an increasing number of annual publications and citations. The United States and China have become leading contributors in this field, however, cooperation and exchanges between countries and institutions still need strengthening. Notably, *The Journal of Neurotrauma* has significant influence within this domain, along with Tianjin Medical University and Charles S. Cox Jr., influential organizations and authors, respectively. According to the burst references, “neurogenic niche” and “immunomodulation” have been identified as research hotspots within this field; further investigation is warranted to explore the potential of “exosomes” and “neuroinflammation.” The research on TBI and stem cells has entered a new stage. Based on the neurogenesis mechanism of endogenous NSC and the treatment of exogenous stem cells, the development of therapeutic strategies for nerve function recovery is expected. Particularly, addressing the urgent issue of combining bioengineering technology with advancements in immune regulation after TBI will pave the way for future directions in post-TBI stem cell therapy.

## Data availability statement

The original contributions presented in the study are included in the article/Supplementary material, further inquiries can be directed to the corresponding authors.

## Author contributions

TD: Conceptualization, Data curation, Formal analysis, Methodology, Software, Visualization, Writing – original draft. RD: Data curation, Formal analysis, Software, Writing – review & editing. YW: Software, Validation, Writing – review & editing. YC: Validation, Visualization, Writing – review & editing. HS: Conceptualization, Methodology, Writing – review & editing. MZ: Conceptualization, Methodology, Project administration, Supervision, Writing – review & editing.

## Footnotes

^1^ https://www.bibliometrix.org

## Supplementary material

SUPPLEMENTARY TABLE S1 The top 10 institutions and betweenness centrality.

SUPPLEMENTARY DATA S1 Raw file with bibliographic information for all 459 articles.
