# Supplementary material for: Mapping knowledge of the stem cell in traumatic brain injury: a bibliometric and visualized analysis
Source: Front Neurol. 2024 Mar 8;15:1301277. doi: 10.3389/fneur.2024.1301277 (PMC10957745; doi:10.3389/fneur.2024.1301277)
Supplement: SUPPLEMENTARY TABLE S1 — The top 10 institutions and betweenness centrality. [file Table_1.docx]

Supplementary Table 1 The top 10 institutions and betweenness centrality

| Rank | Institution | betweenness centrality (BC) |
| --- | --- | --- |
| 1 | Harvard University | 0.32 |
| 2 | Veterans Health Administration (VHA) | 0.30 |
| 3 | University of Texas System | 0.26 |
| 4 | University of South Florida | 0.15 |
| 5 | Uppsala University Hospital | 0.13 |
| 6 | State University System of Florida | 0.11 |
| 7 | University of California System | 0.11 |
| 8 | University of Miami | 0.11 |
| 9 | Baylor College of Medicine | 0.11 |
| 10 | Tianjin Medical University | 0.10 |
