# Supplementary material for: Mapping knowledge of the stem cell in traumatic brain injury: a bibliometric and visualized analysis
Source: Front Neurol. 2024 Mar 8;15:1301277. doi: 10.3389/fneur.2024.1301277 (PMC10957745; doi:10.3389/fneur.2024.1301277)
Supplement: SUPPLEMENTARY DATA S1 — Raw file with bibliographic information for all 459 articles. [file Data_Sheet_1.docx]

Supplementary Data 1. Raw file with bibliographic information for all 459 articles

FN Clarivate Analytics Web of Science

VR 1.0

PT J

AU Dixon, KJ

Theus, MH

Nelersa, CM

Mier, J

Travieso, LG

Yu, TS

Kernie, SG

Liebl, DJ

AF Dixon, Kirsty J.

Theus, Michelle H.

Nelersa, Claudiu M.

Mier, Jose

Travieso, Lissette G.

Yu, Tzong-Shiue

Kernie, Steven G.

Liebl, Daniel J.

TI Endogenous Neural Stem/Progenitor Cells Stabilize the Cortical

Microenvironment after Traumatic Brain Injury

SO JOURNAL OF NEUROTRAUMA

LA English

DT Article

DE gliosis; neural stem; progenitor cell ablation; neurogenesis; neuronal

survival; traumatic brain injury

ID ENDOTHELIAL GROWTH-FACTOR; STEM-CELLS; INTERMEDIATE-FILAMENT; NEURONAL

PRECURSORS; REACTIVE ASTROCYTES; ADULT BRAIN; NEUROGENESIS; RAT;

PROLIFERATION; EXPRESSION

AB Although a myriad of pathological responses contribute to traumatic brain injury (TBI), cerebral dysfunction has been closely linked to cell death mechanisms. A number of therapeutic strategies have been studied in an attempt to minimize or ameliorate tissue damage; however, few studies have evaluated the inherent protective capacity of the brain. Endogenous neural stem/progenitor cells (NSPCs) reside in distinct brain regions and have been shown to respond to tissue damage by migrating to regions of injury. Until now, it remained unknown whether these cells have the capacity to promote endogenous repair. We ablated NSPCs in the subventricular zone to examine their contribution to the injury microenvironment after controlled cortical impact (CCI) injury. Studies were performed in transgenic mice expressing the herpes simplex virus thymidine kinase gene under the control of the nestin promoter exposed to CCI injury. Two weeks after CCI injury, mice deficient in NSPCs had reduced neuronal survival in the perilesional cortex and fewer Iba-1-positive and glial fibrillary acidic protein-positive glial cells but increased glial hypertrophy at the injury site. These findings suggest that the presence of NSPCs play a supportive role in the cortex to promote neuronal survival and glial cell expansion after TBI injury, which corresponds with improvements in motor function. We conclude that enhancing this endogenous response may have acute protective roles after TBI.

C1 [Dixon, Kirsty J.; Nelersa, Claudiu M.; Mier, Jose; Travieso, Lissette G.; Liebl, Daniel J.] Univ Miami, Miami Project Cure Paralysis, Miami, FL 33136 USA.

[Dixon, Kirsty J.; Nelersa, Claudiu M.; Mier, Jose; Travieso, Lissette G.; Liebl, Daniel J.] Univ Miami, Dept Neurol Surg, Miami, FL 33136 USA.

[Theus, Michelle H.] Virginia Polytech Inst & State Univ, Virginia Maryland Reg Coll Vet Med, Dept Biomed Sci & Pathobiol, Blacksburg, VA 24061 USA.

[Yu, Tzong-Shiue; Kernie, Steven G.] Columbia Univ, Dept Pathol & Cell Biol, New York, NY USA.

C3 University of Miami; University of Miami; Virginia Polytechnic Institute

& State University; Columbia University

RP Liebl, DJ (通讯作者)，Univ Miami, Miller Sch Med, Miami Project Cure Paralysis, Dept Neurol Surg, 1095 NW 14th Terrace,R-48, Miami, FL 33136 USA.

EM dliebl@med.miami.edu

RI Kernie, Steven/HZM-4287-2023; Kernie, Steven/AAT-9912-2020

OI Yu, Tzong-Shiue/0000-0003-4277-6213; Kernie, Steven/0000-0003-1371-0549

FU NINDS NIH HHS [NS049545, R01 NS048192, NS064699, NS30291, NS007459]

Funding Source: Medline

CR Ahlemeyer B, 2013, J NEUROSCI METH, V212, P156, DOI 10.1016/j.jneumeth.2012.09.016

Gomez-Climent MA, 2011, CEREB CORTEX, V21, P1028, DOI 10.1093/cercor/bhq177

Arias-Carrion O, 2006, J NEUROSCI RES, V84, P1425, DOI 10.1002/jnr.21068

Arvidsson A, 2002, NAT MED, V8, P963, DOI 10.1038/nm747

Benner EJ, 2013, NATURE, V497, P369, DOI 10.1038/nature12069

Bernal GM, 2011, AGING CELL, V10, P466, DOI 10.1111/j.1474-9726.2011.00694.x

Blaiss CA, 2011, J NEUROSCI, V31, P4906, DOI 10.1523/JNEUROSCI.5265-10.2011

Budinich CS, 2012, NEURAL PLAST, V2012, DOI 10.1155/2012/378307

Butti E, 2012, BRAIN, V135, P3320, DOI 10.1093/brain/aws194

Chen XH, 2003, J NEUROTRAUM, V20, P623, DOI 10.1089/089771503322144545

Chiu CC, 2005, CANCER DETECT PREV, V29, P286, DOI 10.1016/j.cdp.2005.02.001

Cromwell HC, 1996, J NEUROSCI, V16, P3444

Dang MT, 2006, P NATL ACAD SCI USA, V103, P15254, DOI 10.1073/pnas.0601758103

Doyon J, 1996, EUR J NEUROSCI, V8, P637, DOI 10.1111/j.1460-9568.1996.tb01249.x

Ehninger D, 2011, CELL TISSUE RES, V345, P69, DOI 10.1007/s00441-011-1200-z

Ekmark-Lewen S, 2010, RESTOR NEUROL NEUROS, V28, P311, DOI 10.3233/RNN-2010-0529

Eliasson C, 1999, J BIOL CHEM, V274, P23996, DOI 10.1074/jbc.274.34.23996

Faiz M, 2008, MOL CELL NEUROSCI, V38, P170, DOI 10.1016/j.mcn.2008.02.002

Galindo LT, 2011, NEUROL RES INT, V2011, DOI 10.1155/2011/564089

Ghirnikar RS, 1998, NEUROCHEM RES, V23, P329, DOI 10.1023/A:1022453332560

Giunti D, 2012, STEM CELLS, V30, P2044, DOI 10.1002/stem.1174

Guerra-Crespo M, 2009, NEUROSCIENCE, V160, P470, DOI 10.1016/j.neuroscience.2009.02.029

Heymach Jr. J. V., 1995, NATURE, P374

HEYMACH JV, 1995, NATURE, V374, P405, DOI 10.1038/374405a0

Homsi S, 2010, J NEUROTRAUM, V27, P911, DOI 10.1089/neu.2009.1223

Jin KL, 2010, P NATL ACAD SCI USA, V107, P7993, DOI 10.1073/pnas.1000154107

Krum JM, 1998, EXP NEUROL, V154, P57, DOI 10.1006/exnr.1998.6930

Lai B, 2008, NEUROSCI LETT, V442, P305, DOI 10.1016/j.neulet.2008.07.032

Lee ST, 2008, BRAIN, V131, P616, DOI 10.1093/brain/awm306

Li B, 2010, BRAIN RES, V1327, P91, DOI 10.1016/j.brainres.2010.02.030

Liu YP, 2009, ACTA NEUROPATHOL, V117, P469, DOI 10.1007/s00401-009-0516-1

Lossinsky AS, 2004, HISTOL HISTOPATHOL, V19, P535, DOI 10.14670/HH-19.535

Lotocki G, 2009, J NEUROTRAUM, V26, P1123, DOI 10.1089/neu.2008.0802

Luo Chun, 2002, Chin J Traumatol, V5, P24

Ma YH, 2011, BRAIN RES BULL, V86, P441, DOI 10.1016/j.brainresbull.2011.07.007

Mizuno Y, 2006, J NEUROL SCI, V246, P131, DOI 10.1016/j.jns.2006.02.019

MorgantiKossman MC, 1997, MOL PSYCHIATR, V2, P133, DOI 10.1038/sj.mp.4000227

Nakano K, 2000, BRAIN DEV-JPN, V22, pS5

Neves SS, 2006, BBA-BIOMEMBRANES, V1758, P1703, DOI 10.1016/j.bbamem.2006.08.021

Nishijima K, 2007, AM J PATHOL, V171, P53, DOI 10.2353/ajpath.2007.061237

O'Connor CA, 2006, ACTA NEUROCHIR SUPPL, V96, P121

Petridis AK, 2011, J CLIN NEUROSCI, V18, P265, DOI 10.1016/j.jocn.2010.06.021

Proia P, 2008, INT J MOL MED, V21, P63

Raghupathi R, 2004, BRAIN PATHOL, V14, P215, DOI 10.1111/j.1750-3639.2004.tb00056.x

Rani SB, 2006, INDIAN J MED RES, V124, P269

Ricard J, 2006, MOL CELL NEUROSCI, V31, P713, DOI 10.1016/j.mcn.2006.01.002

Salhia B, 2000, BRAIN RES, V883, P87, DOI 10.1016/S0006-8993(00)02825-0

Snapyan M, 2009, J NEUROSCI, V29, P4172, DOI 10.1523/JNEUROSCI.4956-08.2009

Sun CR, 2013, J NEUROSCI, V33, P17314, DOI 10.1523/JNEUROSCI.2129-13.2013

Sun F, 2012, PLOS ONE, V7, DOI 10.1371/journal.pone.0046326

Suzuki R, 2006, ACTA NEUROCHIR SUPPL, V96, P398

Takahashi K, 2005, J EXP MED, V201, P647, DOI 10.1084/jem.20041611

Taupin Philippe, 2008, Int J Med Sci, V5, P127

Teramoto T, 2003, J CLIN INVEST, V111, P1125, DOI 10.1172/JCI200317170

Theus M. H, 2012, CURR PROTOC STEM CEL

Thored P, 2007, STROKE, V38, P3032, DOI 10.1161/STROKEAHA.107.488445

Thored P, 2006, STEM CELLS, V24, P739, DOI 10.1634/stemcells.2005-0281

Wang XL, 2012, PLOS ONE, V7, DOI [10.1371/journal.pone.0040711, 10.1371/journal.pone.0036663]

Wei SJ, 1998, EXP CELL RES, V241, P66, DOI 10.1006/excr.1998.4005

Yu TS, 2008, J NEUROSCI, V28, P12901, DOI 10.1523/JNEUROSCI.4629-08.2008

NR 60

TC 29

Z9 31

U1 1

U2 10

PU MARY ANN LIEBERT, INC

PI NEW ROCHELLE

PA 140 HUGUENOT STREET, 3RD FL, NEW ROCHELLE, NY 10801 USA

SN 0897-7151

EI 1557-9042

J9 J NEUROTRAUM

JI J. Neurotrauma

PD JUN 1

PY 2015

VL 32

IS 11

BP 753

EP 764

DI 10.1089/neu.2014.3390

PG 12

WC Critical Care Medicine; Clinical Neurology; Neurosciences

WE Science Citation Index Expanded (SCI-EXPANDED)

SC General & Internal Medicine; Neurosciences & Neurology

GA CV0YU

UT WOS:000363980700001

PM 25290253

OA Green Published

DA 2023-06-10

ER

PT J

AU Greer, K

Chen, J

Brickler, T

Gourdie, R

Theus, MH

AF Greer, Kisha

Chen, Jiang

Brickler, Thomas

Gourdie, Robert

Theus, Michelle H.

TI Modulation of gap junction-associated Cx43 in neural stem/progenitor

cells following traumatic brain injury

SO BRAIN RESEARCH BULLETIN

LA English

DT Article

DE Cx43; Traumatic brain injury; Neural stem progenitor cell; Gap junction;

Apoptosis

ID ENHANCED HIPPOCAMPAL NEUROGENESIS; COGNITIVE IMPAIRMENT; CONNEXIN

EXPRESSION; SUBVENTRICULAR ZONE; ADULT NEUROGENESIS; PROGENITOR CELLS;

RADIAL GLIA; PHOSPHORYLATION; HEMICHANNELS; ACTIVATION

AB Restoration of learning and memory deficits following traumatic brain injury (TBI) is attributed, in part, to enhanced neural stem/progenitor cell (NSPCs) function. Recent findings suggest gap junction (GJ)-associated connexin 43 (Cx43) plays a key role in the cell cycle regulation and function of NSPCs and is modulated following TBI. Here, we demonstrate that Cx43 is up-regulated in the dentate gyrus following TBI and is expressed on vimentin-positive cells in the subgranular zone. To test the role of Cx43 on NSPCs, we exposed primary cultures to the alpha-connexin Carboxyl Terminal (alpha CT1) peptide which selectively modulates GJ-associated Cx43. Treatment with alpha CT1 substantially reduced proliferation and increased caspase 3/7 expression on NSPCs in a dose-dependent manner. alpha CT1 exposure also reduced overall expression of Cx43 and phospho (p)-Serine368. These findings demonstrate that Cx43 positively regulates adult NPSCs; the modulation of which may influence changes in the dentate gyrus following TBI.

C1 [Greer, Kisha; Chen, Jiang; Brickler, Thomas; Theus, Michelle H.] Virginia Maryland Reg Coll Vet Med, Dept Biomed Sci & Pathobiol, 215 Duck Pond Dr, Blacksburg, VA 24061 USA.

[Gourdie, Robert] Virgnia Tech Carillion Res Inst, Coll Med, 2 Riverside Circle, Roanoke, VA 24016 USA.

RP Theus, MH (通讯作者)，Virginia Tech, Virginia Maryland Reg Coll Vet Med, Dept Biomed Sci & Pathobiol, Duck Pond Dr 0442, Blacksburg, VA 24061 USA.

EM mtheus@vt.edu

FU VT's Institute of Critical Technology and Science; Virginia Maryland

College of Veterinary Medicine; [R01 NS096281]; [NS096281]; [R15

NS081623]

FX The authors would like to thank VT-IMSD Programs for student support

(K.M.G.). This work was supported by R01 NS096281 (MHT), diversity

supplement NS096281 (MHT, KG), R15 NS081623 (MHT) and VT's Institute of

Critical Technology and Science. We recognize the Virginia Maryland

College of Veterinary Medicine for student and financial support. The

content is solely the responsibility of the authors and does not

necessarily represent the official views of the NIH or any other funding

agency.

CR Arciniegas David B., 2002, Curr Treat Options Neurol, V4, P43, DOI 10.1007/s11940-002-0004-6

Baumann G, 2013, EXP BIOL MED, V238, P830, DOI 10.1177/1535370213494558

Bittman K, 1997, J NEUROSCI, V17, P7037

Blaiss CA, 2011, J NEUROSCI, V31, P4906, DOI 10.1523/JNEUROSCI.5265-10.2011

Brickler T, 2016, MEDIAT INFLAMM, V2016, DOI 10.1155/2016/6373506

Cameron SJ, 2003, J BIOL CHEM, V278, P18682, DOI 10.1074/jbc.M213283200

Cheng AW, 2004, DEV BIOL, V272, P203, DOI 10.1016/j.ydbio.2004.04.031

Cina C, 2009, J NEUROSCI, V29, P2009, DOI 10.1523/JNEUROSCI.5025-08.2009

Contreras JE, 2004, BRAIN RES REV, V47, P290, DOI 10.1016/j.brainresrev.2004.08.002

DeSantis CE, 2016, CA-CANCER J CLIN, V66, P31, DOI 10.3322/caac.21320

Dixon KJ, 2015, J NEUROTRAUM, V32, P753, DOI 10.1089/neu.2014.3390

Elias LAB, 2007, NATURE, V448, P901, DOI 10.1038/nature06063

Eriksson PS, 1998, NAT MED, V4, P1313, DOI 10.1038/3305

Eungdamrong NJ, 2004, BIOL CELL, V96, P355, DOI 10.1016/j.biolcel.2004.03.004

Evans WH, 2006, BIOCHEM J, V397, P1, DOI 10.1042/BJ20060175

Frantseva MV, 2002, J NEUROSCI, V22, P644, DOI 10.1523/JNEUROSCI.22-03-00644.2002

Freitas AS, 2012, DEV NEUROBIOL, V72, P1482, DOI 10.1002/dneu.22005

Ghatnekar GS, 2009, REGEN MED, V4, P205, DOI 10.2217/17460751.4.2.205

Goncalves JT, 2016, CELL, V167, P897, DOI 10.1016/j.cell.2016.10.021

Grek CL, 2015, BMC CANCER, V15, DOI 10.1186/s12885-015-1229-6

HAMM RJ, 1993, BEHAV BRAIN RES, V59, P169, DOI 10.1016/0166-4328(93)90164-L

Han XD, 2011, J NEUROTRAUM, V28, P995, DOI 10.1089/neu.2010.1563

Hoang QV, 2010, MOL VIS, V16, P1343

Hunter AW, 2005, MOL BIOL CELL, V16, P5686, DOI 10.1091/mbc.E05-08-0737

Johnson Victoria E, 2015, Handb Clin Neurol, V127, P115, DOI 10.1016/B978-0-444-52892-6.00008-8

Kennedy KL, 2003, MOL REPROD DEV, V64, P61, DOI 10.1002/mrd.10219

Kernie SG, 2010, NEUROBIOL DIS, V37, P267, DOI 10.1016/j.nbd.2009.11.002

Kleindienst A, 2005, J NEUROTRAUM, V22, P645, DOI 10.1089/neu.2005.22.645

Kunze A, 2009, P NATL ACAD SCI USA, V106, P11336, DOI 10.1073/pnas.0813160106

LAIRD DW, 1991, BIOCHEM J, V273, P67, DOI 10.1042/bj2730067

Langlois JA, 2006, J HEAD TRAUMA REHAB, V21, P375, DOI 10.1097/00001199-200609000-00001

Leithe E, 2004, J CELL SCI, V117, P1211, DOI 10.1242/jcs.00951

Lemcke H, 2013, CELL SIGNAL, V25, P2676, DOI 10.1016/j.cellsig.2013.08.030

Leuner B, 2010, ANNU REV PSYCHOL, V61, P111, DOI 10.1146/annurev.psych.093008.100359

Li Shang-Xun, 2011, Fa Yi Xue Za Zhi, V27, P286

Liebmann M, 2013, NEUROSCI LETT, V545, P40, DOI 10.1016/j.neulet.2013.04.013

MALDONADO PE, 1988, J MEMBRANE BIOL, V106, P203, DOI 10.1007/BF01872158

Naus CC, 2016, SEMIN CELL DEV BIOL, V50, P59, DOI 10.1016/j.semcdb.2015.12.008

O'Connor WT, 2011, PHARMACOL THERAPEUT, V130, P106, DOI 10.1016/j.pharmthera.2011.01.001

Ohsumi A, 2010, J NEUROTRAUM, V27, P1255, DOI 10.1089/neu.2009.1234

Plotkin LI, 2002, J BIOL CHEM, V277, P8648, DOI 10.1074/jbc.M108625200

Rapoport MJ, 2005, J NEUROPSYCH CLIN N, V17, P61, DOI 10.1176/appi.neuropsych.17.1.61

Rola R, 2006, EXP NEUROL, V202, P189, DOI 10.1016/j.expneurol.2006.05.034

Saez JC, 2005, BBA-BIOMEMBRANES, V1711, P215, DOI 10.1016/j.bbamem.2005.01.014

Santopietro John, 2015, N C Med J, V76, P96

Schneider CA, 2012, NAT METHODS, V9, P671, DOI 10.1038/nmeth.2089

Seri B, 2004, J COMP NEUROL, V478, P359, DOI 10.1002/cne.20288

Siopi E, 2012, NEUROSCI LETT, V511, P110, DOI 10.1016/j.neulet.2012.01.051

Smyth JW, 2013, CELL REP, V5, P611, DOI 10.1016/j.celrep.2013.10.009

Soder BL, 2009, PLAST RECONSTR SURG, V123, P1440, DOI 10.1097/PRS.0b013e3181a0741d

Solan JL, 2005, BBA-BIOMEMBRANES, V1711, P154, DOI 10.1016/j.bbamem.2004.09.013

Solan JL, 2009, BIOCHEM J, V419, P261, DOI 10.1042/BJ20082319

Spitz G, 2013, J HEAD TRAUMA REHAB, V28, P116, DOI 10.1097/HTR.0b013e3182452f4f

Sun D, 2014, NEURAL REGEN RES, V9, P688, DOI 10.4103/1673-5374.131567

Sun D, 2009, EXP NEUROL, V216, P56, DOI 10.1016/j.expneurol.2008.11.011

Sun LQ, 2015, BEHAV BRAIN RES, V291, P315, DOI 10.1016/j.bbr.2015.05.049

Sun LQ, 2014, NEUROL SCI, V35, P677, DOI 10.1007/s10072-013-1575-6

Sun Y, 2012, INT J ONCOL, V41, P941, DOI 10.3892/ijo.2012.1524

Sutor B, 2005, BBA-BIOMEMBRANES, V1719, P59, DOI 10.1016/j.bbamem.2005.09.005

Sutor Bernd, 2002, Results Probl Cell Differ, V39, P53

Swanson T.M., 2017, PUBLIC HLTH REP

Theus MH, 2014, CELL DEATH DIS, V5, DOI 10.1038/cddis.2014.165

Theus M. H., 2012, CURR PROTOC STEM CEL, V2

Theus MH, 2010, STEM CELLS, V28, P1231, DOI 10.1002/stem.449

Ueki T, 2001, NEUROSCI LETT, V313, P53, DOI 10.1016/S0304-3940(01)02249-2

Vikhamar G, 1998, CELL ADHES COMMUN, V5, P451, DOI 10.3109/15419069809005603

Whiting M.D., 2006, ANIMAL MODELCOGNIT

Wu HT, 2008, J NEUROTRAUM, V25, P130, DOI 10.1089/neu.2007.0369

Yu TS, 2008, J NEUROSCI, V28, P12901, DOI 10.1523/JNEUROSCI.4629-08.2008

Zheng WM, 2013, J NEUROTRAUM, V30, P1872, DOI 10.1089/neu.2010.1579

Zhou JZ, 2014, FEBS LETT, V588, P1186, DOI 10.1016/j.febslet.2014.01.001

NR 71

TC 13

Z9 15

U1 0

U2 2

PU PERGAMON-ELSEVIER SCIENCE LTD

PI OXFORD

PA THE BOULEVARD, LANGFORD LANE, KIDLINGTON, OXFORD OX5 1GB, ENGLAND

SN 0361-9230

EI 1873-2747

J9 BRAIN RES BULL

JI Brain Res. Bull.

PD SEP

PY 2017

VL 134

BP 38

EP 46

DI 10.1016/j.brainresbull.2017.06.016

PG 9

WC Neurosciences

WE Science Citation Index Expanded (SCI-EXPANDED)

SC Neurosciences & Neurology

GA FI3HA

UT WOS:000411847400006

PM 28648814

OA Green Accepted, hybrid, Green Published

DA 2023-06-10

ER

PT J

AU Dwivedi, A

Dwivedi, SS

Tariq, MR

Qiu, XM

Hong, SZ

Xin, Y

AF Dwivedi, Atul

Dwivedi, Shweta Shukla

Tariq, Muhammad Raheel

Qiu, Xiaoming

Hong, Suzhen

Xin, Yu

TI Traumatic Brain Injury and Stem Cell Therapy

SO JOURNAL OF RESEARCH IN MEDICAL AND DENTAL SCIENCE

LA English

DT Article

DE Traumatic brain injury; Mesenchymal stem cells; Stem cell therapy;

Neural stem cells; Endothelial progenitor cells

ID TRANSPLANTATION; SEQUELAE; OUTCOMES

AB Traumatic brain injury is a major health issue globally. Recently, there is no effective treatment, which can improve functional recovery of the TBI patients. Recent studies conclude that stem cell therapy has got tremendous regenerative potential, which can cure CNS injuries. Experimental Success in stem cell therapy also raise a hope in researchers to investigate more. The therapeutic aspects of stem cell therapy are based on the potential to differentiate in to tissue specific cells and integrate in to host tissue to regenerate the lost cells or injured cells. This commentary reflects the role of variety of stem cells in treating TBI .

C1 [Dwivedi, Atul; Hong, Suzhen; Xin, Yu] Hubei Polytech Univ HBPU Huangshi, Dept Clin & Basic Sci, Med Coll, Huangshi, Hubei, Peoples R China.

[Dwivedi, Shweta Shukla] Dept Dent Surg, Jabalpur, Madhya Pradesh, India.

[Tariq, Muhammad Raheel] North Sichuan Med Coll, Dept Internal Med, Affiliated Hosp, Nanchong, Sichuan, Peoples R China.

[Qiu, Xiaoming] Hubei Polytech Univ HBPU, Dept Radiol, Huangshi Cent Hosp, Edong Hlth Care Grp, Huangshi, Hubei, Peoples R China.

C3 North Sichuan Medical University

RP Dwivedi, SS (通讯作者)，Dept Dent Surg, Jabalpur, Madhya Pradesh, India.

EM shweta0761@gmail.com

CR Baethmann A, 1998, Curr Opin Anaesthesiol, V11, P193, DOI 10.1097/00001503-199804000-00013

Clervius H, 2019, NEURAL REGEN RES, V14, P1699, DOI 10.4103/1673-5374.255620

Cox CS, 2018, PEDIATR RES, V83, P325, DOI 10.1038/pr.2017.253

Gill M, 2001, CIRC RES, V88, P167

Harting MT, 2009, J SURG RES, V153, P188, DOI 10.1016/j.jss.2008.03.037

KRAUS JF, 1987, PEDIATRICS, V79, P501

Liu L, 2011, CRIT CARE MED, V39, P1760, DOI 10.1097/CCM.0b013e3182186cee

LOIS C, 1993, P NATL ACAD SCI USA, V90, P2074, DOI 10.1073/pnas.90.5.2074

Maas AIR, 2017, LANCET NEUROL, V16, P987, DOI 10.1016/S1474-4422(17)30371-X

McIntosh TK, 1996, LAB INVEST, V74, P315

Nortje J, 2004, CURR OPIN NEUROL, V17, P711, DOI 10.1097/00019052-200412000-00011

Park KI, 2002, NAT BIOTECHNOL, V20, P1111, DOI 10.1038/nbt751

Rouhl RPW, 2008, STROKE, V39, P2158, DOI 10.1161/STROKEAHA.107.507251

Sinson G, 1996, J NEUROSURG, V84, P655, DOI 10.3171/jns.1996.84.4.0655

Steiner LA, 2006, BRIT J ANAESTH, V97, P26, DOI 10.1093/bja/ael110

Wang S, 2013, BRAIN RES, V1532, P76, DOI 10.1016/j.brainres.2013.08.001

Xiong Y, 2008, BRAIN RES, V1230, P247, DOI 10.1016/j.brainres.2008.06.127

NR 17

TC 2

Z9 2

U1 1

U2 8

PU AMBER PUBLICATION

PI GUJARAT

PA 402, PARIJAT APT, WALKESHWARI NAGAR, JAMNAGAR, GUJARAT, 361 008, INDIA

SN 2347-2545

EI 2347-2367

J9 J RES MED DENT SCI

JI J. Res. Med. Dent. Sci.

PY 2020

VL 8

IS 3

BP 94

EP 96

PG 3

WC Medicine, Research & Experimental

WE Emerging Sources Citation Index (ESCI)

SC Research & Experimental Medicine

GA MJ5HK

UT WOS:000548121100015

DA 2023-06-10

ER

PT J

AU Baumann, G

Travieso, L

Liebl, DJ

Theus, MH

AF Baumann, Gisela

Travieso, Lissette

Liebl, Daniel J.

Theus, Michelle H.

TI Pronounced hypoxia in the subventricular zone following traumatic brain

injury and the neural stem/progenitor cell response

SO EXPERIMENTAL BIOLOGY AND MEDICINE

LA English

DT Article

DE Hypoxia; Eph; ephrin; subventricular zone; AKT; Hif1-alpha; neural

stem/progenitor cell; traumatic brain injury

ID ADULT MAMMALIAN BRAIN; CENTRAL-NERVOUS-SYSTEM; STEM-CELLS; COMMISSURAL

AXONS; PROGENITOR CELLS; OLFACTORY-BULB; PROLIFERATION; NEUROGENESIS;

DIFFERENTIATION; SURVIVAL

AB Traumatic brain injury (TBI) elicits identifiable changes within the adult subventricular zone (SVZ). Previously, we demonstrated that EphB3/ephrinB3 interaction inhibits neural stem/progenitor cell (NSPC) proliferation and downregulating this pathway following TBI plays a pivotal role in the expansion of the SVZ neurogenic compartment. It remains unclear, however, what early initiating factors may precede these changes. Using hypoxyprobe-1 (HPb) to identify regions of low oxygen tension or hypoxia (<1%), we found HPb uptake throughout the cortex (CTX), corpus callosum (CC) and SVZ within the first 24 h following controlled cortical impact (CCI) injury. At this early time point, HPb co-localized with EphB3 in the SVZ. NSPC specific markers also co-localized with HPb staining throughout the lateral wall of the ventricle. To determine the cell autonomous effects of hypoxia on EphB3/ephrinB3 signaling in NSPCs, we used an in vitro model of hypoxia to mimic 1% oxygen in the presence and absence of soluble aggregated ephrinB3 (eB3). As expected, hypoxia stimulated the uptake of 5-bromo-2'-deoxyuridine (BrdU) and reduced cell death. Coincident with these proliferative changes, both Hif1-alpha and phospho (p)-AKT were increased while EphB3 expression was decreased. Stimulation of EphB3 attenuated hypoxia-induced proliferation and prevented phosphorylation of AKT. Hif1-alpha accumulation, on the other hand, was not affected by EphB3/ephrinB3 signaling. These findings indicate that this pathway limits the NSPC response to hypoxic stimuli. These studies also suggest that early transient changes in oxygen tension following localized cortical injury may initiate a growth-promoting response in the SVZ.

C1 [Baumann, Gisela; Travieso, Lissette; Liebl, Daniel J.] Univ Miami, Miami Project Cure Paralysis, Miami, FL 33136 USA.

[Baumann, Gisela; Travieso, Lissette; Liebl, Daniel J.] Univ Miami, Dept Neurol Surg, Miami, FL 33136 USA.

[Theus, Michelle H.] Virginia Polytech Inst & State Univ, Virginia Maryland Reg Coll Vet Med, Dept Biomed Sci & Pathobiol, Blacksburg, VA 24061 USA.

C3 University of Miami; University of Miami; Virginia Polytechnic Institute

& State University

RP Theus, MH (通讯作者)，Virginia Polytech Inst & State Univ, Virginia Maryland Reg Coll Vet Med, Dept Biomed Sci & Pathobiol, Blacksburg, VA 24061 USA.

EM mtheus@vt.edu

FU NIH/NINDS [NS049545, NS30291, NS007459, NS064699]; Virginia Tech's Open

Access Subvention Fund; Miami Project to Cure Paralysis

FX This work was supported by NIH/NINDS NS049545 (DJL), NS30291 (DJL),

NS007459 (MHT), NS064699 (MHT), Virginia Tech's Open Access Subvention

Fund and the Miami Project to Cure Paralysis.

CR ALTMAN J, 1965, J COMP NEUROL, V124, P319, DOI 10.1002/cne.901240303

Alvarez-Buylla A, 2002, BRAIN RES BULL, V57, P751, DOI 10.1016/S0361-9230(01)00770-5

Bessis A, 2007, GLIA, V55, P233, DOI 10.1002/glia.20459

Blits-Huizinga CT, 2004, IUBMB LIFE, V56, P257, DOI 10.1080/15216540412331270076

Chakraborty D, 2012, CELL CYCLE, V11, P2427, DOI 10.4161/cc.20542

Chen BS, 2012, ENDOCRINOLOGY, V153, P4946, DOI 10.1210/en.2012-1472

Chirumamilla S, 2002, J NEUROTRAUM, V19, P693, DOI 10.1089/08977150260139084

Conover JC, 2000, NAT NEUROSCI, V3, P1091, DOI 10.1038/80606

Cowan CA, 2000, NEURON, V26, P417, DOI 10.1016/S0896-6273(00)81174-5

Culver JC, 2013, PLOS ONE, V8, DOI 10.1371/journal.pone.0053546

Doetsch F, 1999, P NATL ACAD SCI USA, V96, P11619, DOI 10.1073/pnas.96.20.11619

Doetsch F, 2002, J NEUROSCI, V22, P2255, DOI 10.1523/JNEUROSCI.22-06-02255.2002

Doetsch F, 1999, CELL, V97, P703, DOI 10.1016/S0092-8674(00)80783-7

Doetsch F, 1997, J NEUROSCI, V17, P5046

Doetsch F, 1996, P NATL ACAD SCI USA, V93, P14895, DOI 10.1073/pnas.93.25.14895

Flanagan JG, 1998, ANNU REV NEUROSCI, V21, P309, DOI 10.1146/annurev.neuro.21.1.309

Gritti A, 2002, J NEUROSCI, V22, P437, DOI 10.1523/JNEUROSCI.22-02-00437.2002

Henkemeyer M, 1996, CELL, V86, P35, DOI 10.1016/S0092-8674(00)80075-6

Jin KL, 2003, AGING CELL, V2, P175, DOI 10.1046/j.1474-9728.2003.00046.x

Katakowski M, 2005, NEUROSCI LETT, V385, P204, DOI 10.1016/j.neulet.2005.05.060

Keith B, 2007, CELL, V129, P465, DOI 10.1016/j.cell.2007.04.019

Leone DP, 2005, J CELL SCI, V118, P2589, DOI 10.1242/jcs.02396

Lu DY, 2003, J NEUROSURG, V99, P351, DOI 10.3171/jns.2003.99.2.0351

Mignone JL, 2004, J COMP NEUROL, V469, P311, DOI 10.1002/cne.10964

Milosevic J, 2005, J NEUROCHEM, V92, P718, DOI 10.1111/j.1471-4159.2004.02893.x

Morrison SJ, 2000, J NEUROSCI, V20, P7370

Mottet D, 2003, J BIOL CHEM, V278, P31277, DOI 10.1074/jbc.M300763200

Myer DJ, 2006, BRAIN, V129, P2761, DOI 10.1093/brain/awl165

Nelersa CM, 2012, J BIOMOL SCREEN, V17, P785, DOI 10.1177/1087057112440880

Ogasawara MA, 2009, ANTIOXID REDOX SIGN, V11, P1107, DOI [10.1089/ars.2008.2308, 10.1089/ARS.2008.2308]

Ohnuma S, 2003, NEURON, V40, P199, DOI 10.1016/S0896-6273(03)00632-9

Orioli D, 1996, EMBO J, V15, P6035, DOI 10.1002/j.1460-2075.1996.tb00992.x

Ostrakhovitch EA, 2013, ARCH BIOCHEM BIOPHYS, V534, P44, DOI 10.1016/j.abb.2012.08.002

Panchision DM, 2009, J CELL PHYSIOL, V220, P562, DOI 10.1002/jcp.21812

Pasquale EB, 2008, CELL, V133, P38, DOI 10.1016/j.cell.2008.03.011

Pore N, 2006, MOL CANCER RES, V4, P471, DOI 10.1158/1541-7786.MCR-05-0234

Ramaswamy S, 2005, BRAIN RES, V1053, P38, DOI 10.1016/j.brainres.2005.06.042

REYNOLDS BA, 1992, SCIENCE, V255, P1707, DOI 10.1126/science.1553558

Ricard J, 2006, MOL CELL NEUROSCI, V31, P713, DOI 10.1016/j.mcn.2006.01.002

Rice AC, 2003, EXP NEUROL, V183, P406, DOI 10.1016/S0014-4886(03)00241-3

Roy S, 2012, EUR J HAEMATOL, V88, P396, DOI 10.1111/j.1600-0609.2012.01759.x

Scheffler B, 2005, P NATL ACAD SCI USA, V102, P9353, DOI 10.1073/pnas.0503965102

Skold MK, 2005, J NEUROTRAUM, V22, P353, DOI 10.1089/neu.2005.22.353

Studer L, 2000, J NEUROSCI, V20, P7377

Theus MH, 2010, STEM CELLS, V28, P1231, DOI 10.1002/stem.449

Theus MH, 2008, EXP NEUROL, V210, P656, DOI 10.1016/j.expneurol.2007.12.020

Urrea C, 2007, RESTOR NEUROL NEUROS, V25, P65

Vieira HLA, 2011, PROG NEUROBIOL, V93, P444, DOI 10.1016/j.pneurobio.2011.01.007

Wang TW, 2005, DEVELOPMENT, V132, P2721, DOI 10.1242/dev.01867

Xing SH, 2008, BRAIN RES, V1230, P237, DOI 10.1016/j.brainres.2008.06.097

Yokoyama N, 2001, NEURON, V29, P85, DOI 10.1016/S0896-6273(01)00182-9

NR 51

TC 13

Z9 13

U1 0

U2 5

PU ROYAL SOC MEDICINE PRESS LTD

PI LONDON

PA 1 WIMPOLE STREET, LONDON W1G 0AE, ENGLAND

SN 1535-3702

J9 EXP BIOL MED

JI Exp. Biol. Med.

PD JUL

PY 2013

VL 238

IS 7

BP 830

EP 841

DI 10.1177/1535370213494558

PG 12

WC Medicine, Research & Experimental

WE Science Citation Index Expanded (SCI-EXPANDED)

SC Research & Experimental Medicine

GA 203ZY

UT WOS:000323335200013

PM 23828590

OA Green Accepted, Green Published

DA 2023-06-10

ER

PT J

AU Anderson, J

Patel, M

Forenzo, D

Ai, X

Cai, C

Wade, Q

Risman, R

Cai, L

AF Anderson, Jeremy

Patel, Misaal

Forenzo, Dylan

Ai, Xin

Cai, Catherine

Wade, Quinn

Risman, Rebecca

Cai, Li

TI A novel mouse model for the study of endogenous neural stem and

progenitor cells after traumatic brain injury

SO EXPERIMENTAL NEUROLOGY

LA English

DT Article

DE Traumatic brain injury; Closed head injury; Neural stem/progenitor cell;

Notch1CR2-GFP; Mouse model; GABAergic neuron

ID CLOSED-HEAD INJURY; ADULT SUBVENTRICULAR ZONE; GENE-EXPRESSION; NOTCH;

PROLIFERATION; NEUROGENESIS; ACTIVATION; MECHANISMS; ASTROCYTES;

REGENERATION

AB Traumatic brain injury (TBI) is a leading cause of death and disability in the US. Neural stem/progenitor cells (NSPCs) persist in the adult brain and represent a potential cell source for tissue regeneration and wound healing after injury. The Notch signaling pathway is critical for embryonic development and adult brain injury response. However, the specific role of Notch signaling in the injured brain is not well characterized. Our previous study has established a Notch1CR2-GFP reporter mouse line in which the Notch1CR2 enhancer directs GFP expression in NSPCs and their progeny. In this study, we performed closed head injury (CHI) in the Notch1CR2-GFP mice to study the response of injury-activated NSPCs. We show that CHI induces neuroinflammation, cell death, and the expression of typical TBI markers (e.g., ApoE, Illb, and Tau), validating the animal model. In addition, CHI induces cell proliferation in GFP + cells expressing NSPC markers, e.g., Notch1 and Nestin. A significant higher percentage of GFP + astrocytes and GABAergic neurons was observed in the injured brain, with no significant change in oligodendrocyte lineage between the CHI and sham animal groups. Since injury is known to activate astrogliosis, our results suggest that injury-induced GFP + NSPCs preferentially differentiate into GABAergic neurons. Our study establishes that Notch1CR2-GFP transgenic mouse is a useful tool for the study of NSPC behavior in vivo after TBI. Unveiling the potential of NSPCs response to TBI (e.g., proliferation and differentiation) will identify new therapeutic strategy for the treatment of brain trauma.

C1 [Anderson, Jeremy; Patel, Misaal; Forenzo, Dylan; Ai, Xin; Cai, Catherine; Wade, Quinn; Risman, Rebecca; Cai, Li] Rutgers State Univ, Dept Biomed Engn, 599 Taylor Rd, Piscataway, NJ 08854 USA.

C3 Rutgers State University New Brunswick

RP Cai, L (通讯作者)，Rutgers State Univ, Dept Biomed Engn, 599 Taylor Rd, Piscataway, NJ 08854 USA.

EM lcai@rutgers.edu

OI Anderson, Jeremy/0000-0002-6032-6534; Cai, Li/0000-0003-3344-337X

FU New Jersey Commission on Spinal Cord Research [08-3074-SCR-E0,

10-3091-SCR-E-0, 15IRG006]; Busch Biomedical Award [659218]; NIH

Biotechnology Training Program [NIH] [T32 GM00839]; U.S. Department of

Education GAANN Precision and Personalized Medicine Pre-Doctoral

Training Fellowship [P200A150131]

FX The authors would like to thank members of the Cai lab for helpful

discussion and the Maribel Vasquez lab for imaging analysis. This work

was supported by the grants from the New Jersey Commission on Spinal

Cord Research [08-3074-SCR-E0; 10-3091-SCR-E-0; 15IRG006] and Busch

Biomedical Award [659218]. J.A. and M.N.P. are fellows of the NIH

Biotechnology Training Program [NIH T32 GM00839]. M.P. is also a

recipient of the U.S. Department of Education GAANN Precision and

Personalized Medicine Pre-Doctoral Training Fellowship (P200A150131).

CR Ables JL, 2011, NAT REV NEUROSCI, V12, P269, DOI 10.1038/nrn3024

Ables JL, 2010, J NEUROSCI, V30, P10484, DOI 10.1523/JNEUROSCI.4721-09.2010

[Anonymous], [No title captured]

Arnold K, 2011, CELL STEM CELL, V9, P317, DOI 10.1016/j.stem.2011.09.001

Artegiani B, 2017, CELL REP, V21, P3271, DOI 10.1016/j.celrep.2017.11.050

Barraud P, 2005, EUR J NEUROSCI, V22, P1555, DOI 10.1111/j.1460-9568.2005.04352.x

Benner EJ, 2013, NATURE, V497, P369, DOI 10.1038/nature12069

Braun SMG, 2014, DEVELOPMENT, V141, P1983, DOI 10.1242/dev.104596

Campolo M, 2018, METHODS MOL BIOL, V1727, P385, DOI 10.1007/978-1-4939-7571-6_30

Carlen M, 2009, NAT NEUROSCI, V12, P259, DOI 10.1038/nn.2268

Chang EH, 2016, FRONT NEUROSCI-SWITZ, V10, DOI 10.3389/fnins.2016.00332

Chapouton P, 2010, J NEUROSCI, V30, P7961, DOI 10.1523/JNEUROSCI.6170-09.2010

Chen CM, 2017, J DATA INFO SCI, V2, P1, DOI 10.1515/jdis-2017-0006

Chen Y, 1996, J NEUROTRAUM, V13, P557, DOI 10.1089/neu.1996.13.557

Chiu CC, 2016, J NEUROSCI METH, V272, P38, DOI 10.1016/j.jneumeth.2016.06.018

Chojnacki A, 2003, J NEUROSCI, V23, P1730

Dixon KJ, 2015, J NEUROTRAUM, V32, P753, DOI 10.1089/neu.2014.3390

Dulken BW, 2017, CELL REP, V18, P777, DOI 10.1016/j.celrep.2016.12.060

Evanson NK, 2018, PLOS ONE, V13, DOI 10.1371/journal.pone.0197346

Flierl MA, 2009, NAT PROTOC, V4, P1328, DOI 10.1038/nprot.2009.148

Gao Q, 2018, STEM CELLS DEV, V27, P479, DOI 10.1089/scd.2017.0193

Gao X, 2009, EXP NEUROL, V219, P516, DOI 10.1016/j.expneurol.2009.07.007

Gardner AJ, 2017, PHYS MED REH CLIN N, V28, P413, DOI 10.1016/j.pmr.2016.12.014

Giachino C, 2014, FRONT NEUROSCI-SWITZ, V8, DOI 10.3389/fnins.2014.00032

Givogri MI, 2006, DEV NEUROSCI-BASEL, V28, P81, DOI 10.1159/000090755

Hasan A, 2017, FRONT NEUROL, V8, DOI 10.3389/fneur.2017.00028

Hsieh CL, 2013, EUR J IMMUNOL, V43, P2010, DOI 10.1002/eji.201243084

Imayoshi I, 2011, MOL NEUROBIOL, V44, P7, DOI 10.1007/s12035-011-8186-0

Khalin I, 2016, NEURAL REGEN RES, V11, P630, DOI 10.4103/1673-5374.180749

Kim HJ, 2018, JCI INSIGHT, V3, DOI 10.1172/jci.insight.97105

Kishimoto N, 2012, DIS MODEL MECH, V5, P200, DOI 10.1242/dmm.007336

Kumar A, 2016, J NEUROTRAUM, V33, P1732, DOI 10.1089/neu.2015.4268

LeComte MD, 2016, CYTOKINE, V80, P64, DOI 10.1016/j.cyto.2015.08.259

LeComte MD, 2015, P NATL ACAD SCI USA, V112, P8726, DOI 10.1073/pnas.1501029112

Leinhase I, 2006, BMC NEUROSCI, V7, DOI 10.1186/1471-2202-7-55

Li Y, 2016, SCI REP-UK, V6, DOI 10.1038/srep38665

Lim DA, 2016, CSH PERSPECT BIOL, V8, DOI 10.1101/cshperspect.a018820

Livak KJ, 2001, METHODS, V25, P402, DOI 10.1006/meth.2001.1262

Louvi A, 2006, NAT REV NEUROSCI, V7, P93, DOI 10.1038/nrn1847

Ludwig PE, 2018, NEURAL REGEN RES, V13, P7, DOI 10.4103/1673-5374.224361

Ma MW, 2017, OXID MED CELL LONGEV, V2017, DOI 10.1155/2017/6057609

Ma XT, 2019, MOL NEUROBIOL, V56, P5332, DOI 10.1007/s12035-018-1454-5

Makara JK, 2003, MOL CELL NEUROSCI, V23, P521, DOI 10.1016/S1044-7431(03)00080-0

Encinas JM, 2017, ADV DRUG DELIVER REV, V120, P118, DOI 10.1016/j.addr.2017.07.016

Mignone JL, 2004, J COMP NEUROL, V469, P311, DOI 10.1002/cne.10964

Ming GL, 2011, NEURON, V70, P687, DOI 10.1016/j.neuron.2011.05.001

Newell EA, 2018, ENEURO, V5, DOI 10.1523/ENEURO.0385-17.2018

Oya S, 2009, NEUROSCIENCE, V158, P683, DOI 10.1016/j.neuroscience.2008.10.043

Patel K, 2016, BRAIN RES, V1640, P104, DOI 10.1016/j.brainres.2016.01.055

Phipps HW, 2016, METHODS MOL BIOL, V1462, P61, DOI 10.1007/978-1-4939-3816-2_5

Pluta R, 2018, PHARMACOL REP, V70, P881, DOI 10.1016/j.pharep.2018.03.004

Puhakka N, 2017, PLOS ONE, V12, DOI 10.1371/journal.pone.0172521

Ray SK, 2002, HISTOL HISTOPATHOL, V17, P1137, DOI 10.14670/HH-17.1137

Reis C, 2017, STEM CELLS INT, V2017, DOI 10.1155/2017/6392592

Schwab JM, 2002, J NEUROSURG, V96, P892, DOI 10.3171/jns.2002.96.5.0892

Shimada IS, 2011, STROKE, V42, P3231, DOI 10.1161/STROKEAHA.111.623280

Sibbe M, 2012, EUR J NEUROSCI, V36, P3643, DOI 10.1111/j.1460-9568.2012.08279.x

Stoica BA, 2010, NEUROTHERAPEUTICS, V7, P3, DOI 10.1016/j.nurt.2009.10.023

Sun MJ, 2018, FRONT NEUROL, V9, DOI 10.3389/fneur.2018.01089

Tanigaki K, 2001, NEURON, V29, P45, DOI 10.1016/S0896-6273(01)00179-9

Tatsumi K, 2010, J CHEM NEUROANAT, V39, P15, DOI 10.1016/j.jchemneu.2009.09.003

Tu M, 2017, MED SCI MONITOR, V23, P5480, DOI 10.12659/MSM.907160

Turtzo LC, 2014, J NEUROINFLAMM, V11, DOI 10.1186/1742-2094-11-82

Tzatzalos E, 2012, DEV BIOL, V372, P217, DOI 10.1016/j.ydbio.2012.09.015

Van KC, 2016, METHODS MOL BIOL, V1462, P231, DOI 10.1007/978-1-4939-3816-2_14

Wang XM, 2009, J CEREBR BLOOD F MET, V29, P1644, DOI 10.1038/jcbfm.2009.83

Wang XT, 2016, ENEURO, V3, DOI 10.1523/ENEURO.0162-16.2016

Wang Y, 2012, EXP BIOL MED, V237, P1424, DOI 10.1258/ebm.2012.012123

Weston NM, 2018, CURR NEUROL NEUROSCI, V18, DOI 10.1007/s11910-018-0812-z

Woo SM, 2009, BMC NEUROSCI, V10, DOI 10.1186/1471-2202-10-97

Yan YH, 2018, BRAIN BEHAV IMMUN, V67, P118, DOI 10.1016/j.bbi.2017.08.011

Yoon K, 2005, NAT NEUROSCI, V8, P709, DOI 10.1038/nn1475

Zhang YM, 2015, NEUROSCI LETT, V603, P12, DOI 10.1016/j.neulet.2015.07.009

Zhao CM, 2008, CELL, V132, P645, DOI 10.1016/j.cell.2008.01.033

Zhou ZD, 2010, IUBMB LIFE, V62, P618, DOI 10.1002/iub.362

NR 75

TC 5

Z9 5

U1 0

U2 9

PU ACADEMIC PRESS INC ELSEVIER SCIENCE

PI SAN DIEGO

PA 525 B ST, STE 1900, SAN DIEGO, CA 92101-4495 USA

SN 0014-4886

EI 1090-2430

J9 EXP NEUROL

JI Exp. Neurol.

PD MAR

PY 2020

VL 325

AR 113119

DI 10.1016/j.expneurol.2019.113119

PG 12

WC Neurosciences

WE Science Citation Index Expanded (SCI-EXPANDED)

SC Neurosciences & Neurology

GA KH0CW

UT WOS:000510317000018

PM 31751572

DA 2023-06-10

ER

PT J

AU Walker, PA

Shah, SK

Jimenez, F

Gerber, MH

Xue, HS

Cutrone, R

Hamilton, JA

Mays, RW

Deans, R

Pati, S

Dash, PK

Cox, CS

AF Walker, Peter A.

Shah, Shinil K.

Jimenez, Fernando

Gerber, Michael H.

Xue, Hasen

Cutrone, Rochelle

Hamilton, Jason A.

Mays, Robert W.

Deans, Robert

Pati, Shibani

Dash, Pramod K.

Cox, Charles S., Jr.

TI Intravenous multipotent adult progenitor cell therapy for traumatic

brain injury: Preserving the blood brain barrier via an interaction with

splenocytes

SO EXPERIMENTAL NEUROLOGY

LA English

DT Article

DE Multipotent adult progenitor cells; Traumatic brain injury; Stem cells;

Splenocytes; Blood brain barrier

ID MESENCHYMAL STEM-CELLS; BONE-MARROW; EXPERIMENTAL STROKE;

TRANSPLANTATION

AB Recent investigation has shown an interaction between transplanted progenitor cells and resident splenocytes leading to the modulation of the immunologic response in neurological injury. We hypothesize that the intravenous injection of multipotent adult progenitor cells (MAPC) confers neurovascular protection after traumatic brain injury through an interaction with resident splenocytes, subsequently leading to preservation of the blood brain barrier.

Four groups of rats underwent controlled cortical impact injury (3 groups) or sham injury (1 group). MAPC were injected via the tail vein at two doses (2*10(6) MAPC/kg or 10*10(6) MAPC/kg) 2 and 24 h after injury. Blood brain barrier permeability was assessed by measuring Evans blue dye extravasation (n = 6/group). Additionally, splenic mass was measured (n = 12/group) followed by splenocyte characterization (n = 9/group) including: cell cycle analysis (n = 6/group), apoptosis index (n = 6/group), cell proliferation (n = 6/group), and inflammatory cytokine measurements (n = 6/group). Vascular architecture was determined by immunohistochemistry (n = 3/group).

Traumatic brain injury results in a decrease in splenic mass and increased blood brain barrier permeability. Intravenous infusion of MAPC preserved splenic mass and returned blood brain barrier permeability towards control sham injured levels. Splenocyte characterization indicated an increase in the number and proliferative rate of CD4+ T cells as well as an increase in IL-4 and IL-10 production in stimulated splenocytes isolated from the MAPC treatment groups. Immunohistochemistry demonstrated stabilization of the vascular architecture in the pen-lesion area.

Traumatic brain injury causes a reduction in splenic mass that correlates with an increase in circulating immune cells leading to increased blood brain barrier permeability. The intravenous injection of MAPC preserves splenic mass and the integrity of the blood brain barrier. Furthermore, the co-localization of transplanted MAPC and resident CD4+ splenocytes is associated with a global increase in IL-4 and IL-10 production and stabilization of the cerebral microvasculature tight junction proteins. (C) 2010 Elsevier Inc. All rights reserved.

C1 [Walker, Peter A.; Shah, Shinil K.; Jimenez, Fernando; Xue, Hasen; Cox, Charles S., Jr.] Univ Texas Med Sch Houston, Dept Pediat Surg, Houston, TX 77030 USA.

[Walker, Peter A.; Shah, Shinil K.; Gerber, Michael H.; Xue, Hasen; Pati, Shibani; Cox, Charles S., Jr.] Univ Texas Med Sch Houston, Dept Surg, Houston, TX 77030 USA.

[Gerber, Michael H.; Dash, Pramod K.] Univ Texas Med Sch Houston, Dept Neurobiol & Anat, Houston, TX 77030 USA.

[Shah, Shinil K.; Cox, Charles S., Jr.] Texas A&M Univ, Michael E DeBakey Inst Comparat Cardiovasc Sci &, College Stn, TX USA.

[Cutrone, Rochelle; Hamilton, Jason A.; Mays, Robert W.; Deans, Robert] Athersys Inc, Dept Regenerat Med, Cleveland, OH USA.

C3 University of Texas System; University of Texas Health Science Center

Houston; University of Texas System; University of Texas Health Science

Center Houston; University of Texas System; University of Texas Health

Science Center Houston; Texas A&M University System; Texas A&M

University College Station

RP Cox, CS (通讯作者)，Univ Texas Med Sch Houston, Dept Pediat Surg, 6431 Fannin St,MSB 5-236, Houston, TX 77030 USA.

EM Charles.s.cox@uth.tmc.edu

RI Dash, Pramod Kumar/F-5832-2011

OI Dash, Pramod Kumar/0000-0001-6746-1002

FU NIH [T32 GM 08 79201, M01 RR 02558]; Texas Higher Education Coordinating

Board; Children's Memorial Hermann Hospital Foundation; Texas Emerging

Technology Fund; Athersys, Inc.

FX This work was supported by grants from NIH T32 GM 08 79201; M01 RR

02558; Texas Higher Education Coordinating Board; Children's Memorial

Hermann Hospital Foundation; Texas Emerging Technology Fund; Athersys,

Inc.

CR Ajmo CT, 2009, EXP NEUROL, V218, P47, DOI 10.1016/j.expneurol.2009.03.044

Boozer Sherry, 2009, J Stem Cells, V4, P17, DOI jsc.2009.4.1.17

Castro RF, 2002, SCIENCE, V297, P1299, DOI 10.1126/science.297.5585.1299

Cotton BA, 2007, J TRAUMA, V62, P26, DOI 10.1097/TA.0b013e31802d02d0

Deng J, 2006, STEM CELLS, V24, P1054, DOI 10.1634/stemcells.2005-0370

English D, 2006, EXP NEUROL, V199, P10, DOI 10.1016/j.expneurol.2006.03.005

Faul M, 2007, J TRAUMA, V63, P1271, DOI 10.1097/TA.0b013e3181493080

Fischer UM, 2009, STEM CELLS DEV, V18, P683, DOI 10.1089/scd.2008.0253

Harting MT, 2008, SURGERY, V144, P803, DOI 10.1016/j.surg.2008.05.017

Harting MT, 2008, BRAIN, V131, DOI 10.1093/brain/awn142

Harting MT, 2009, J NEUROSURG, V110, P1189, DOI 10.3171/2008.9.JNS08158

Jiang YH, 2002, EXP HEMATOL, V30, P896, DOI 10.1016/S0301-472X(02)00869-X

Liao WB, 2009, TRANSPLANTATION, V87, P350, DOI 10.1097/TP.0b013e318195742e

Lighthall JW, 1988, J NEUROTRAUM, V5, P1, DOI 10.1089/neu.1988.5.1

Lim JH, 2007, J VET SCI, V8, P275, DOI 10.4142/jvs.2007.8.3.275

Offner H, 2006, J CEREBR BLOOD F MET, V26, P654, DOI 10.1038/sj.jcbfm.9600217

Offner H, 2006, J IMMUNOL, V176, P6523, DOI 10.4049/jimmunol.176.11.6523

PATI S, STEM CELLS IN PRESS

Qu CS, 2008, BRAIN RES, V1208, P234, DOI 10.1016/j.brainres.2008.02.042

Ragnarsson KT, 1999, JAMA-J AM MED ASSOC, V282, P974

Schrepfer S, 2007, TRANSPL P, V39, P573, DOI 10.1016/j.transproceed.2006.12.019

Thurman DJ, 1999, J HEAD TRAUMA REHAB, V14, P602, DOI 10.1097/00001199-199912000-00009

Vendrame M, 2006, EXP NEUROL, V199, P191, DOI 10.1016/j.expneurol.2006.03.017

Walker PA, 2009, DIS MODEL MECH, V2, P23, DOI 10.1242/dmm.001198

Zhao LR, 2002, EXP NEUROL, V174, P11, DOI 10.1006/exnr.2001.7853

NR 25

TC 101

Z9 105

U1 0

U2 7

PU ACADEMIC PRESS INC ELSEVIER SCIENCE

PI SAN DIEGO

PA 525 B ST, STE 1900, SAN DIEGO, CA 92101-4495 USA

SN 0014-4886

EI 1090-2430

J9 EXP NEUROL

JI Exp. Neurol.

PD OCT

PY 2010

VL 225

IS 2

BP 341

EP 352

DI 10.1016/j.expneurol.2010.07.005

PG 12

WC Neurosciences

WE Science Citation Index Expanded (SCI-EXPANDED)

SC Neurosciences & Neurology

GA 654SV

UT WOS:000282191500014

PM 20637752

OA Green Accepted

DA 2023-06-10

ER

PT J

AU Hetz, RA

Bedi, SS

Olson, S

Olsen, A

Cox, CS

AF Hetz, Robert A.

Bedi, Supinder S.

Olson, Scott

Olsen, Alex

Cox, Charles S., Jr.

TI Progenitor Cells: Therapeutic Targets after Traumatic Brain Injury

SO TRANSLATIONAL STROKE RESEARCH

LA English

DT Review

DE Traumatic brain injury; TBI; Stem cells; Progenitor cells; Bone marrow

derived mononuclear cells; MAPC; MSC; Microglia; Inflammatory reflex;

Spleen

ID MOUSE SPINAL-CORD; INFLAMMATORY RESPONSE; ISCHEMIA-REPERFUSION;

TIME-COURSE; ACTIVATION; MICROGLIA; IDENTIFICATION; RECEPTOR; DAMAGE;

NERVE

AB Traumatic brain injuries and their associated treatments carry high cost in both financial impact and morbidity to human life. Recent studies and trials present promising results in reducing secondary injury in the days and weeks following the primary insult. A number of studies, both pre-clinical and clinical, have found that different populations of stem/progenitor cells result in a reduction of inflammation, maintenance of the blood brain barrier, and an overall improved prognosis. The mechanism of action of these cellular therapies appears to rely upon the ability of the cells to influence microglia/macrophage phenotype and alter the state of the inflammatory response. The spleen has become an area of intense interest as an arena where therapeutic cells interact with reactive macrophages to cause system-level changes in immune activity. Additionally, the spleen enacts anti-inflammatory responses originating in the CNS, delivered through vagal activity with a recently described mechanism culminating in acetylcholine release. This review provides a summary of recent findings as to the mechanisms of action observed in current cellular therapies.

C1 [Cox, Charles S., Jr.] Brown Fdn Inst Mol Med, Dept Stem Cell Res & Regenerat Med, Senator Lloyd & BA Bentsen Ctr Stroke Res, Houston, TX USA.

[Hetz, Robert A.; Bedi, Supinder S.; Olson, Scott; Olsen, Alex; Cox, Charles S., Jr.] Univ Texas Houston, Med Sch Houston, Dept Pediat Surg, Houston, TX 77030 USA.

[Cox, Charles S., Jr.] Texas A&M Univ, Michael E DeBakey Inst Comparat Cardiovasc Sci &, College Stn, TX USA.

C3 University of Texas System; University of Texas Health Science Center

Houston; Texas A&M University System; Texas A&M University College

Station

RP Cox, CS (通讯作者)，Brown Fdn Inst Mol Med, Dept Stem Cell Res & Regenerat Med, Senator Lloyd & BA Bentsen Ctr Stroke Res, Houston, TX USA.

EM charles.s.cox@uth.tmc.edu

OI Olson, Scott/0000-0001-8032-3755

FU Brown Foundation

FX This work is partially funded by the Brown Foundation.; Dr. Cox has

stock and royalty interests in EMIT Corp and sponsored research

agreements with Athersys Inc. and Celgene Inc.

CR Ajmo CT, 2009, EXP NEUROL, V218, P47, DOI 10.1016/j.expneurol.2009.03.044

Bareyre F, 1997, J NEUROTRAUM, V14, P839, DOI 10.1089/neu.1997.14.839

Beck KD, 2010, BRAIN, V133, P433, DOI 10.1093/brain/awp322

BELLINGER D L, 1989, Brain Behavior and Immunity, V3, P291, DOI 10.1016/0889-1591(89)90029-9

BELLINGER DL, 1993, BRAIN BEHAV IMMUN, V7, P191, DOI 10.1006/brbi.1993.1021

BERTHOUD HR, 1993, J AUTONOM NERV SYST, V42, P153, DOI 10.1016/0165-1838(93)90046-W

Borovikova LV, 2000, NATURE, V405, P458, DOI 10.1038/35013070

BULLOCK R, 1991, J NEUROL NEUROSUR PS, V54, P427, DOI 10.1136/jnnp.54.5.427

Chirumamilla S, 2002, J NEUROTRAUM, V19, P693, DOI 10.1089/08977150260139084

Coronado Victor G., 2011, Morbidity and Mortality Weekly Report, V60, P1

Coyne TM, 2006, STEM CELLS, V24, P2483, DOI 10.1634/stemcells.2006-0174

Cusimano M, 2012, BRAIN, V135, P447, DOI 10.1093/brain/awr339

David S, 2011, NAT REV NEUROSCI, V12, P388, DOI 10.1038/nrn3053

FISHMAN RA, 1975, NEW ENGL J MED, V293, P706, DOI 10.1056/NEJM197510022931407

Gaetz M, 2004, CLIN NEUROPHYSIOL, V115, P4, DOI 10.1016/S1388-2457(03)00258-X

Gordon S, 2003, NAT REV IMMUNOL, V3, P23, DOI 10.1038/nri978

Graeber MB, 2010, SCIENCE, V330, P783, DOI 10.1126/science.1190929

Guarini S, 2003, CIRCULATION, V107, P1189, DOI 10.1161/01.CIR.0000050627.90734.ED

Harting MT, 2008, SURGERY, V144, P803, DOI 10.1016/j.surg.2008.05.017

Huang XY, 2009, J NEUROIMMUNOL, V211, P3, DOI 10.1016/j.jneuroim.2009.04.006

Jiang HC, 2007, SURGERY, V141, P32, DOI 10.1016/j.surg.2006.03.024

Kigerl KA, 2009, J NEUROSCI, V29, P13435, DOI 10.1523/JNEUROSCI.3257-09.2009

Kin NW, 2006, J LEUKOCYTE BIOL, V79, P1093, DOI 10.1189/jlb.1105625

Lee ST, 2008, BRAIN, V131, P616, DOI 10.1093/brain/awm306

Lenzlinger PM, 2001, J NEUROTRAUM, V18, P479, DOI 10.1089/089771501300227288

Lenzlinger PM, 2001, MOL NEUROBIOL, V24, P169

Li M, 2011, J TRAUMA, V71, P141, DOI 10.1097/TA.0b013e3181f30fc9

Loane DJ, 2010, NEUROTHERAPEUTICS, V7, P366, DOI 10.1016/j.nurt.2010.07.002

Lu D, 2001, NEUROREPORT, V12, P559, DOI 10.1097/00001756-200103050-00025

Mebius RE, 2005, NAT REV IMMUNOL, V5, P606, DOI 10.1038/nri1669

Nakajima K, 2001, J BIOCHEM-TOKYO, V130, P169, DOI 10.1093/oxfordjournals.jbchem.a002969

Nimmerjahn A, 2005, SCIENCE, V308, P1314, DOI 10.1126/science.1110647

Okuaki Y, 1996, LIVER, V16, P188

Papadopoulos V, 1998, P SOC EXP BIOL MED, V217, P130

Pineau I, 2010, BRAIN BEHAV IMMUN, V24, P540, DOI 10.1016/j.bbi.2009.11.007

Pineau I, 2007, J COMP NEUROL, V500, P267, DOI 10.1002/cne.21149

Ramlackhansingh AF, 2011, ANN NEUROL, V70, P374, DOI 10.1002/ana.22455

Rao VLR, 2000, EXP NEUROL, V161, P102, DOI 10.1006/exnr.1999.7269

Rosas-Ballina M, 2009, J INTERN MED, V265, P663, DOI 10.1111/j.1365-2796.2009.02098.x

Rosas-Ballina M, 2011, SCIENCE, V334, P98, DOI 10.1126/science.1209985

Savas MC, 2003, J PEDIATR SURG, V38, P1465, DOI 10.1016/S0022-3468(03)00497-4

Smith HS, 2010, PAIN PHYSICIAN, V13, P295

Stocchetti N, 2007, J NEUROTRAUM, V24, P1339, DOI 10.1089/neu.2007.0300

Thiel A, 2010, J NUCL MED, V51, P1404, DOI 10.2967/jnumed.110.076612

Tracey KJ, 2002, NATURE, V420, P853, DOI 10.1038/nature01321

Vendrame M, 2006, EXP NEUROL, V199, P191, DOI 10.1016/j.expneurol.2006.03.017

Walker PA, 2010, EXP NEUROL, V225, P341, DOI 10.1016/j.expneurol.2010.07.005

Wang H, 2003, NATURE, V421, P384, DOI 10.1038/nature01339

ZHUANG J, 1993, J TRAUMA, V35, P415, DOI 10.1097/00005373-199309000-00014

NR 50

TC 5

Z9 5

U1 0

U2 9

PU SPRINGER

PI NEW YORK

PA ONE NEW YORK PLAZA, SUITE 4600, NEW YORK, NY, UNITED STATES

SN 1868-4483

EI 1868-601X

J9 TRANSL STROKE RES

JI Transl. Stroke Res.

PD SEP

PY 2012

VL 3

IS 3

SI SI

BP 318

EP 323

DI 10.1007/s12975-012-0192-7

PG 6

WC Clinical Neurology; Neurosciences

WE Science Citation Index Expanded (SCI-EXPANDED)

SC Neurosciences & Neurology

GA 992FK

UT WOS:000307760800004

PM 24323807

DA 2023-06-10

ER

PT J

AU Walker, PA

Bedi, SS

Shah, SK

Jimenez, F

Xue, H

Hamilton, JA

Smith, P

Thomas, CP

Mays, RW

Pati, S

Cox, CS

AF Walker, Peter A.

Bedi, Supinder S.

Shah, Shinil K.

Jimenez, Fernando

Xue, Hasen

Hamilton, Jason A.

Smith, Philippa

Thomas, Chelsea P.

Mays, Robert W.

Pati, Shibani

Cox, Charles S., Jr.

TI Intravenous multipotent adult progenitor cell therapy after traumatic

brain injury: modulation of the resident microglia population

SO JOURNAL OF NEUROINFLAMMATION

LA English

DT Article

DE Multipotent adult progenitor cells; Traumatic brain injury; Stem cells;

Splenocytes; Blood brain barrier; Microglia

ID CENTRAL-NERVOUS-SYSTEM; INFLAMMATORY RESPONSE; SPINAL-CORD

AB Introduction: We have demonstrated previously that the intravenous delivery of multipotent adult progenitor cells (MAPC) after traumatic brain injury affords neuroprotection via interaction with splenocytes, leading to an increase in systemic anti-inflammatory cytokines. We hypothesize that the observed modulation of the systemic inflammatory milieu is related to T regulatory cells and a subsequent increase in the locoregional neuroprotective M2 macrophage population.

Methods: C57B6 mice were injected with intravenous MAPC 2 and 24 hours after controlled cortical impact injury. Animals were euthanized 24, 48, 72, and 120 hours after injury. In vivo, the proportion of CD4(+)/CD25(+)/FOXP3(+) T-regulatory cells were measured in the splenocyte population and plasma. In addition, the brain CD86(+) M1 and CD206(+) M2 macrophage populations were quantified. A series of in vitro co-cultures were completed to investigate the need for direct MAPC:splenocyte contact as well as the effect of MAPC therapy on M1 and M2 macrophage subtype apoptosis and proliferation.

Results: Significant increases in the splenocyte and plasma T regulatory cell populations were observed with MAPC therapy at 24 and 48 hours, respectively. In addition, MAPC therapy was associated with an increase in the brain M2/M1 macrophage ratio at 24, 48 and 120 hours after cortical injury. In vitro cultures of activated microglia with supernatant derived from MAPC:splenocyte co-cultures also demonstrated an increase in the M2/M1 ratio. The observed changes were secondary to an increase in M1 macrophage apoptosis.

Conclusions: The data show that the intravenous delivery of MAPC after cortical injury results in increases in T regulatory cells in splenocytes and plasma with a concordant increase in the locoregional M2/M1 macrophage ratio. Direct contact between the MAPC and splenocytes is required to modulate activated microglia, adding further evidence to the central role of the spleen in MAPC-mediated neuroprotection.

C1 [Walker, Peter A.; Shah, Shinil K.; Xue, Hasen; Pati, Shibani; Cox, Charles S., Jr.] Univ Texas Houston, Sch Med, Dept Surg, Houston, TX 77030 USA.

[Walker, Peter A.; Bedi, Supinder S.; Shah, Shinil K.; Jimenez, Fernando; Xue, Hasen; Smith, Philippa; Thomas, Chelsea P.; Cox, Charles S., Jr.] Univ Texas Houston, Sch Med, Houston, TX 77030 USA.

[Shah, Shinil K.; Cox, Charles S., Jr.] Texas A&M Univ, Michael E DeBakey Inst Comparat Cardiovasc Sci &, College Stn, TX USA.

[Hamilton, Jason A.; Mays, Robert W.] Athersys Inc, Dept Regenerat Med, Cleveland, OH 44115 USA.

C3 University of Texas System; University of Texas Health Science Center

Houston; University of Texas System; University of Texas Health Science

Center Houston; Texas A&M University System; Texas A&M University

College Station

RP Cox, CS (通讯作者)，Univ Texas Houston, Sch Med, Dept Surg, 6431 Fannin St,MSB 5-236, Houston, TX 77030 USA.

EM Charles.s.cox@uth.tmc.edu

FU Athersys, Inc.; Cord Blood Registry, Inc.; BD Biosciences, Inc.; NIH

[T32 GM 08 79201]; Texas Higher Education Coordinating Board; Children's

Memorial Hermann Hospital Foundation; Texas Emerging Technology Fund; BD

Biosciences; [M01 RR 02558]

FX There are no known conflicts between the authors and the information

presented in this paper. Charles S. Cox Jr. MD has sponsored research

agreements with Athersys, Inc. and Cord Blood Registry, Inc. Peter A.

Walker MD, Fernando Jimenez MS, Shinil K. Shah DO, and Charles S. Cox,

Jr. MD have received grant support from BD Biosciences, Inc. Jason A.

Hamilton PhD and Robert W. Mays PhD are employed by Athersys, Inc.

Athersys Inc. supplied the bone marrow derived progenitor cells for all

experiments.; NIH T32 GM 08 79201; M01 RR 02558; Texas Higher Education

Coordinating Board; Children's Memorial Hermann Hospital Foundation;

Texas Emerging Technology Fund; Athersys, Inc.; BD Biosciences.

CR Aloisi F, 2001, GLIA, V36, P165, DOI 10.1002/glia.1106

Beck KD, 2010, BRAIN, V133, P433, DOI 10.1093/brain/awp322

Chirumamilla S, 2002, J NEUROTRAUM, V19, P693, DOI 10.1089/08977150260139084

Fischer UM, 2009, STEM CELLS DEV, V18, P683, DOI 10.1089/scd.2008.0253

Gordon S, 2003, NAT REV IMMUNOL, V3, P23, DOI 10.1038/nri978

Graeber MB, 2010, SCIENCE, V330, P783, DOI 10.1126/science.1190929

Harting MT, 2008, SURGERY, V144, P803, DOI 10.1016/j.surg.2008.05.017

Jiang YH, 2002, EXP HEMATOL, V30, P896, DOI 10.1016/S0301-472X(02)00869-X

Kigerl KA, 2009, J NEUROSCI, V29, P13435, DOI 10.1523/JNEUROSCI.3257-09.2009

Kovacsovics-Bankowski M, 2008, CYTOTHERAPY, V10, P730, DOI 10.1080/14653240802320245

Kovacsovics-Bankowski M, 2009, CELL IMMUNOL, V255, P55, DOI 10.1016/j.cellimm.2008.10.004

Lakhan SE, 2009, J TRANSL MED, V7, DOI 10.1186/1479-5876-7-97

Lighthall JW, 1988, J NEUROTRAUM, V5, P1, DOI 10.1089/neu.1988.5.1

Loane DJ, 2010, NEUROTHERAPEUTICS, V7, P366, DOI 10.1016/j.nurt.2010.07.002

Olson JK, 2004, J IMMUNOL, V173, P3916, DOI 10.4049/jimmunol.173.6.3916

Smith HS, 2010, PAIN PHYSICIAN, V13, P295

Thurman DJ, 1999, J HEAD TRAUMA REHAB, V14, P602, DOI 10.1097/00001199-199912000-00009

Walker PA, 2010, EXP NEUROL, V225, P341, DOI 10.1016/j.expneurol.2010.07.005

NR 18

TC 83

Z9 84

U1 0

U2 13

PU BIOMED CENTRAL LTD

PI LONDON

PA 236 GRAYS INN RD, FLOOR 6, LONDON WC1X 8HL, ENGLAND

SN 1742-2094

J9 J NEUROINFLAMM

JI J. Neuroinflamm.

PD SEP 28

PY 2012

VL 9

AR 228

DI 10.1186/1742-2094-9-228

PG 13

WC Immunology; Neurosciences

WE Science Citation Index Expanded (SCI-EXPANDED)

SC Immunology; Neurosciences & Neurology

GA 087GW

UT WOS:000314750500002

PM 23020860

OA Green Published, gold

DA 2023-06-10

ER

PT J

AU Huang, XT

Wan, DH

Lin, YP

Xue, NZ

Hao, JH

Ma, N

Pei, XL

Li, RL

Zhang, WJ

AF Huang, Xintao

Wan, Dahai

Lin, Yunpeng

Xue, Naizhao

Hao, Jiehe

Ma, Ning

Pei, Xile

Li, Ruilong

Zhang, Wenju

TI Endothelial Progenitor Cells Correlated with Oxidative Stress after Mild

Traumatic Brain Injury

SO YONSEI MEDICAL JOURNAL

LA English

DT Article

DE Traumatic brain injury; endothelial progenitor cells; superoxide

dismutase; malonyldialdehyde

ID ISCHEMIC-STROKE; STEM-CELLS; TRANSPLANTATION; ANGIOGENESIS; MODEL;

NEUROPROTECTION; INFLAMMATION; THERAPIES; RATS; MICE

AB Purpose: Endothelial progenitor cells (EPCs) play a key role in tissue repair and regeneration. Previous studies have shown that infusion of human umbilical cord blood-derived endothelial colony-forming cells improves outcomes in mice subjected to experimental traumatic brain injury (TBI). However, the efficiency of cell transplantation is not satisfactory. Oxidative stress plays a significant role in the survival of transplanted cells following ischemic reperfusion injury. This observational clinical study investigated the correlation between the number of circulating EPCs and plasma levels of superoxide dismutase (SOD) and malonyldialdehyde (MDA).

Materials and Methods: Peripheral blood samples were collected from 20 patients with mild TBI at day-1, day-2, day-3, day-4, and day-7 post TBI. The number of circulating EPCs and the plasma levels of SOD and MDA were measured.

Results: The average of circulating EPCs in TBI patients decreased initially, but increased thereafter, compared with healthy controls. Plasma levels of SOD in TBI patients were significantly lower than those in healthy controls at day-4 post-TBI. MDA levels showed no difference between the two groups. Furthermore, when assessed on day-7 post-TBI, the circulating EPC number were correlated with the plasma levels of SOD and MDA.

Conclusion: These results suggest that the number of circulating EPCs is weakly to moderately correlated with plasma levels of SOD and MDA at day-7 post-TBI, which may offer a novel antioxidant strategy for EPCs transplantation after TBI.

C1 [Huang, Xintao; Wan, Dahai; Xue, Naizhao; Hao, Jiehe; Ma, Ning; Pei, Xile; Li, Ruilong; Zhang, Wenju] Shanxi Med Univ, Affiliated Hosp 1, Dept Neurosurg, 85 Jiefangnan Rd, Taiyuan 030001, Shanxi, Peoples R China.

[Lin, Yunpeng] Tianjin Med Univ, Gen Hosp, Dept Neurosurg, Tianjin, Peoples R China.

C3 Shanxi Medical University; Tianjin Medical University

RP Huang, XT (通讯作者)，Shanxi Med Univ, Affiliated Hosp 1, Dept Neurosurg, 85 Jiefangnan Rd, Taiyuan 030001, Shanxi, Peoples R China.

EM xintaoh@hotmail.com

OI Huang, Xintao/0000-0002-9683-9518

FU Shanxi Health and Family Planning Commission [2015023]; Shanxi Science

and Technology Fund [2016D202097]

FX The present work was supported by the Shanxi Health and Family Planning

Commission Research Fund (grant no.: 2015023) and the Shanxi Science and

Technology Fund for Young Scholars (grant no.: 2016D202097).

CR Abdul-Muneer PM, 2015, MOL NEUROBIOL, V51, P966, DOI 10.1007/s12035-014-8752-3

Abdul-Muneer PM, 2013, FREE RADICAL BIO MED, V60, P282, DOI 10.1016/j.freeradbiomed.2013.02.029

Arent AM, 2014, BIOMED RES INT, V2014, DOI 10.1155/2014/723060

Asahara T, 1997, SCIENCE, V275, P964, DOI 10.1126/science.275.5302.964

Bayir H, 2002, PEDIATR RES, V51, P571, DOI 10.1203/00006450-200205000-00005

Briasoulis A, 2011, CARDIOVASC THER, V29, P125, DOI 10.1111/j.1755-5922.2009.00131.x

Cernak I, 2000, J NEUROTRAUM, V17, P53, DOI 10.1089/neu.2000.17.53

Cornelius C, 2013, ANTIOXID REDOX SIGN, V19, P836, DOI 10.1089/ars.2012.4981

Fukui M, 2010, FREE RADICAL BIO MED, V48, P821, DOI 10.1016/j.freeradbiomed.2009.12.024

Gennai S, 2015, BRIT J ANAESTH, V115, P203, DOI 10.1093/bja/aev229

Hall ED, 2010, NEUROTHERAPEUTICS, V7, P51, DOI 10.1016/j.nurt.2009.10.021

Hayward NMEA, 2010, J NEUROTRAUM, V27, P2203, DOI 10.1089/neu.2010.1448

Huang XT, 2013, J NEUROTRAUM, V30, P2080, DOI 10.1089/neu.2013.2996

Kochanek PM, 2015, SEMIN NEUROL, V35, P83, DOI 10.1055/s-0035-1544237

Liao YL, 2013, PLOS ONE, V8, DOI 10.1371/journal.pone.0068963

Liu L, 2007, J NEUROTRAUM, V24, P936, DOI 10.1089/neu.2006.0250

Liu L, 2011, CRIT CARE MED, V39, P1760, DOI 10.1097/CCM.0b013e3182186cee

Lu XY, 2014, NEUROCHEM INT, V69, P14, DOI 10.1016/j.neuint.2014.02.006

Mojtahedzadeh M, 2014, J RES MED SCI, V19, P867

Monsel A, 2014, ANESTHESIOLOGY, V121, P1099, DOI 10.1097/ALN.0000000000000446

Nakagomi N, 2009, STEM CELLS, V27, P2185, DOI 10.1002/stem.161

Rodriguez-Rodriguez A, 2014, CURR MED CHEM, V21, P1201, DOI 10.2174/0929867321666131217153310

Sakata H, 2012, STROKE, V43, P2423, DOI 10.1161/STROKEAHA.112.656900

Sakata H, 2012, J NEUROSCI, V32, P3462, DOI 10.1523/JNEUROSCI.5686-11.2012

Shen LH, 2012, NEUROSCIENCE, V223, P315, DOI 10.1016/j.neuroscience.2012.08.001

Xiong Y, 2010, CURR OPIN INVEST DR, V11, P298

Yu P, 2016, CELL PROLIFERAT, V49, P48, DOI 10.1111/cpr.12231

Zhang YQ, 2013, J SURG RES, V185, P441, DOI 10.1016/j.jss.2013.05.073

NR 28

TC 8

Z9 8

U1 0

U2 6

PU YONSEI UNIV COLL MEDICINE

PI SEOUL

PA 50-1 YONSEI-RO, SEODAEMUN-GU, SEOUL 120-752, SOUTH KOREA

SN 0513-5796

EI 1976-2437

J9 YONSEI MED J

JI Yonsei Med. J.

PD SEP

PY 2017

VL 58

IS 5

BP 1012

EP 1017

DI 10.3349/ymj.2017.58.5.1012

PG 6

WC Medicine, General & Internal

WE Science Citation Index Expanded (SCI-EXPANDED)

SC General & Internal Medicine

GA FH1JT

UT WOS:000410897000017

PM 28792147

OA gold, Green Published, Green Submitted

DA 2023-06-10

ER

PT J

AU Liu, L

Wei, HJ

Chen, FL

Wang, JH

Dong, JF

Zhang, JN

AF Liu, Li

Wei, Huijie

Chen, Fanglian

Wang, Jinghua

Dong, Jing-fei

Zhang, Jianning

TI Endothelial progenitor cells correlate with clinical outcome of

traumatic brain injury

SO CRITICAL CARE MEDICINE

LA English

DT Article

DE endothelial progenitor cells; traumatic brain injury; clinical outcome;

prognostic marker

ID ACTIVATED PLATELETS; PRECURSOR CELLS; CD34(+) CELLS; STEM-CELLS;

DIFFERENTIATION; MOBILIZATION; ANGIOGENESIS; REPAIR; STROKE

AB Objective: Endothelial progenitor cells play an active role in vascular repair and revascularization of tissue damaged by traumatic, inflammatory, and ischemic injures. We correlate the changes in circulating endothelial progenitor cells with the severity of traumatic brain injury. The study is designed to investigate the endothelial progenitor cell mobilization after injury and a potential use of circulating endothelial progenitor cells as a prognostic marker for evaluating trauma severity and clinical outcomes.

Design: A prospective cohort study conducted in two neurosurgical intensive care units of Tianjin Medical University General Hospital and Tianjin Huanhu Hospital (Tianjin, China).

Patients: Patients with traumatic brain injury and age-and gender-matched healthy controls.

Interventions: None.

Measurements and Main Results: Changes in the levels of circulating endothelial progenitor cells were monitored for up to 21 days in 84 patients with traumatic brain injury. Results were correlated with the clinical assessment of injury severity as determined by the Glasgow Coma Scale. The level of circulating endothelial progenitor cells was found to be suppressed 24-48 hrs after injury but rapidly increased, reaching the highest at days 5-7 post-trauma. Circulating endothelial progenitor cells in patients with improved Glasgow Coma Scale scores were significantly higher than those with deteriorated conditions and remained persistently low in patients who died of trauma.

Conclusions: The results suggest that the level of circulating endothelial progenitor cells correlates with the clinical severity and outcome of traumatic brain injury and may offer potential as a prognostic marker for traumatic brain injury. A long-term follow-up of these patients is ongoing. (Crit Care Med 2011; 39: 1760-1765)

C1 [Liu, Li; Wei, Huijie; Chen, Fanglian; Wang, Jinghua; Zhang, Jianning] Tianjin Med Univ Gen Hosp, Tianjin Neurol Inst, Dept Neurosurg, Tianjin, Peoples R China.

Tianjin Key Lab Injuries Variat & Regenerat Nervo, Tianjin, Peoples R China.

[Dong, Jing-fei] Baylor Coll Med, Dept Med, Thrombosis Res Sect, Houston, TX 77030 USA.

C3 Tianjin Medical University; Baylor College of Medicine

RP Liu, L (通讯作者)，Tianjin Med Univ Gen Hosp, Tianjin Neurol Inst, Dept Neurosurg, Tianjin, Peoples R China.

EM jfdong@bcm.tmc.edu; jianningzhang@hotmail.com

FU Chinese Science Foundation (Beijing, China) [2005CB522600, 30772229];

National Institutes of Health (Bethesda, MD) [HL71895]

FX Supported, in part, by grants 2005CB522600 (JZ) and 30772229 (JZ) from

the Chinese Science Foundation (Beijing, China) and grant HL71895 (JFD)

from the National Institutes of Health (Bethesda, MD).

CR Asahara T, 1997, SCIENCE, V275, P964, DOI 10.1126/science.275.5302.964

de Boer HC, 2006, ARTERIOSCL THROM VAS, V26, P1653, DOI 10.1161/01.ATV.0000222982.55731.f1

Dimmeler S, 2004, J MOL MED, V82, P671, DOI 10.1007/s00109-004-0580-x

Gehling UM, 2000, BLOOD, V95, P3106

Gill M, 2001, CIRC RES, V88, P167

Guo XB, 2009, J NEUROTRAUM, V26, P1337, DOI [10.1089/neu.2008.0733, 10.1089/neu.2008-0733]

Hill JM, 2003, NEW ENGL J MED, V348, P593, DOI 10.1056/NEJMoa022287

Hristov M, 2004, J CELL MOL MED, V8, P498, DOI 10.1111/j.1582-4934.2004.tb00474.x

Korbling M, 2003, NEW ENGL J MED, V349, P570, DOI 10.1056/NEJMra022361

Laing AJ, 2007, J ORTHOP RES, V25, P44, DOI 10.1002/jor.20228

Langer HF, 2007, SEMIN THROMB HEMOST, V33, P136, DOI 10.1055/s-2007-969026

Lev EI, 2005, J VASC RES, V42, P408, DOI 10.1159/000087370

Lev EI, 2006, THROMB HAEMOSTASIS, V96, P498, DOI 10.1160/TH06-05-0250

Liu L, 2007, J NEUROTRAUM, V24, P936, DOI 10.1089/neu.2006.0250

Peichev M, 2000, BLOOD, V95, P952, DOI 10.1182/blood.V95.3.952.003k27_952_958

Pintucci G, 2002, THROMB HAEMOSTASIS, V88, P834, DOI 10.1055/s-0037-1613311

Rouhl RPW, 2008, STROKE, V39, P2158, DOI 10.1161/STROKEAHA.107.507251

Stocchetti N, 2007, J NEUROTRAUM, V24, P1339, DOI 10.1089/neu.2007.0300

Walter DH, 2002, CIRCULATION, V105, P3017, DOI 10.1161/01.CIR.0000018166.84319.55

Yip HK, 2008, STROKE, V39, P69, DOI 10.1161/STROKEAHA.107.489401

NR 20

TC 33

Z9 39

U1 0

U2 2

PU LIPPINCOTT WILLIAMS & WILKINS

PI PHILADELPHIA

PA 530 WALNUT ST, PHILADELPHIA, PA 19106-3621 USA

SN 0090-3493

J9 CRIT CARE MED

JI Crit. Care Med.

PD JUL

PY 2011

VL 39

IS 7

BP 1760

EP 1765

DI 10.1097/CCM.0b013e3182186cee

PG 6

WC Critical Care Medicine

WE Science Citation Index Expanded (SCI-EXPANDED)

SC General & Internal Medicine

GA 778RF

UT WOS:000291721800019

PM 21460712

OA Green Accepted

DA 2023-06-10

ER

PT J

AU Ata, MT

Turgut, G

Akbulut, M

Kocyigit, A

Karabulut, A

Senol, H

Turgut, S

AF Ata, Melek Tunc

Turgut, Gunfer

Akbulut, Metin

Kocyigit, Ali

Karabulut, Aysun

Senol, Hande

Turgut, Sebahat

TI Effect of Erythropoietin and Stem Cells on Traumatic Brain Injury

SO WORLD NEUROSURGERY

LA English

DT Article

DE Erythropoietin; Stem cell; Traumatic brain injury

ID ELDERLY GLIOBLASTOMA PATIENTS; OLDER PATIENTS; FRAILTY INDEX;

POSTOPERATIVE COMPLICATIONS; PERIOPERATIVE COMPLICATIONS; RECURRENT

GLIOBLASTOMA; ABBREVIATED COURSE; RADIATION-THERAPY; ADVERSE OUTCOMES;

UNITED-STATES

AB OBJECTIVE: To investigate the healing effects of erythropoietin (EPO) and stem cells (SCs) in traumatic brain injury (TBI).

METHODS: Twenty-nine Wistar albino rats were used and separated into the following groups: control (C), EPO, SC, and SC+EPO. Group C received a TBI only, with no treatment. In the EPO group, 1000 U/kg EPO was given intraperitoneally at 30 minutes after TBI. In SC group, immediately after formation of TBI, 3 x 10,000 CD34(+) stem cells were injected into the affected area. In the SC+EPO group, half an hour after TBI and the injection of stemcells, 1000 U/kg EPO was injected. Before and after injury, trauma coordination performance was measured by the rotarod and inclined plane tests.

RESULTS: Seven weeks after trauma, rat brains were examined by radiology and histology. Rotarod performance test did not change remarkably, even after the injury. Compared with group C, the SC+EPO group was found to have significant differences in the inclined plane test results.

CONCLUSIONS: Separately given, SCs and EPO have a positive effect on TBI, and our findings suggest that their coadministration is even more powerful.

C1 [Ata, Melek Tunc; Turgut, Gunfer; Turgut, Sebahat] Pamukkale Univ, Dept Physiol, Denizli, Turkey.

[Akbulut, Metin] Pamukkale Univ, Dept Pathol, Denizli, Turkey.

[Kocyigit, Ali] Pamukkale Univ, Dept Radiol, Denizli, Turkey.

[Karabulut, Aysun] Pamukkale Univ, Dept Obstet & Gynecol, Denizli, Turkey.

[Senol, Hande] Pamukkale Univ, Dept Biostat, Denizli, Turkey.

C3 Pamukkale University; Akdeniz University; Pamukkale University;

Pamukkale University; Pamukkale University; Pamukkale University

RP Ata, MT (通讯作者)，Pamukkale Univ, Dept Physiol, Denizli, Turkey.

EM tuncmelekk@hotmail.com

RI Turgut, Sebahat/AGZ-2624-2022

OI Turgut, Sebahat/0000-0002-7629-2631; Turgut, Gunfer/0000-0002-6329-4398

CR Ackerl M, 2014, CLIN NEUROPATHOL, V33, P399, DOI 10.5414/NP300761

Adams P, 2013, JAMA OTOLARYNGOL, V139, P783, DOI 10.1001/jamaoto.2013.3969

Chang SM, 2003, J NEUROSURG, V98, P1175, DOI 10.3171/jns.2003.98.6.1175

CHARLSON ME, 1987, J CHRON DIS, V40, P373, DOI 10.1016/0021-9681(87)90171-8

Clegg A, 2013, LANCET, V381, P752, DOI 10.1016/S0140-6736(12)62167-9

Clegg A, 2011, CLIN MED, V11, P72, DOI 10.7861/clinmedicine.11-1-72

Connon FV, 2016, NEUROSURG REV, V39, P55, DOI 10.1007/s10143-015-0652-0

Conroy S, 2012, ACUTE MED, V12, P74

D'Amico RS, 2015, WORLD NEUROSURG, V84, P913, DOI 10.1016/j.wneu.2015.05.072

Dasgupta M, 2009, ARCH GERONTOL GERIAT, V48, P78, DOI 10.1016/j.archger.2007.10.007

De Lepeleire J, 2009, BRIT J GEN PRACT, V59, P364, DOI 10.3399/bjgp09X420653

Dubrow R, 2011, BMC CANCER, V11, DOI 10.1186/1471-2407-11-325

Evans SJ, 2014, AGE AGEING, V43, P127, DOI 10.1093/ageing/aft156

Fisher JL, 2007, NEUROL CLIN, V25, P867, DOI 10.1016/j.ncl.2007.07.002

Gulati S, 2011, WORLD NEUROSURG, V76, P572, DOI 10.1016/j.wneu.2011.06.014

Hartmann C, 2010, ACTA NEUROPATHOL, V120, P707, DOI 10.1007/s00401-010-0781-z

Hartmann C, 2009, ACTA NEUROPATHOL, V118, P469, DOI 10.1007/s00401-009-0561-9

Iwamoto FM, 2009, CANCER-AM CANCER SOC, V115, P3758, DOI 10.1002/cncr.24413

Iwamoto FM, 2008, ANN NEUROL, V64, P628, DOI 10.1002/ana.21521

Jeremic B, 1999, J NEURO-ONCOL, V44, P85, DOI 10.1023/A:1006356021734

Joseph B, 2014, JAMA SURG, V149, P766, DOI 10.1001/jamasurg.2014.296

Karam J, 2013, ANN VASC SURG, V27, P904, DOI 10.1016/j.avsg.2012.09.015

KARNOFSKY DA, 1948, CANCER-AM CANCER SOC, V1, P634, DOI 10.1002/1097-0142(194811)1:4<634::AID-CNCR2820010410>3.0.CO;2-L

Kulminski A, 2007, MECH AGEING DEV, V128, P250, DOI 10.1016/j.mad.2006.12.004

Lamborn KR, 2004, NEURO-ONCOLOGY, V6, P227, DOI 10.1215/S1152851703000620

Laperriere N, 2013, CANCER TREAT REV, V39, P350, DOI 10.1016/j.ctrv.2012.05.008

Lee TH, 1999, CIRCULATION, V100, P1043, DOI 10.1161/01.CIR.100.10.1043

Makary MA, 2010, J AM COLL SURGEONS, V210, P901, DOI 10.1016/j.jamcollsurg.2010.01.028

Nghiemphu PL, 2009, NEUROLOGY, V72, P1217, DOI 10.1212/01.wnl.0000345668.03039.90

POMPEI P, 1988, J CLIN EPIDEMIOL, V41, P275, DOI 10.1016/0895-4356(88)90132-1

Reyngold M, 2012, J NEURO-ONCOL, V110, P369, DOI 10.1007/s11060-012-0972-7

Roa W, 2004, J CLIN ONCOL, V22, P1583, DOI 10.1200/JCO.2004.06.082

Roa W, 2009, EXPERT REV ANTICANC, V9, P1643, DOI 10.1586/ERA.09.128

Robinson TN, 2013, AM J SURG, V206, P544, DOI 10.1016/j.amjsurg.2013.03.012

Rockwood K, 2005, CAN MED ASSOC J, V173, P489, DOI 10.1503/cmaj.050051

Rockwood K, 2007, J GERONTOL A-BIOL, V62, P722, DOI 10.1093/gerona/62.7.722

Schuurmans H, 2004, J GERONTOL A-BIOL, V59, P962

Socha J, 2015, J NEUROONCOL, P1

Tanaka S, 2013, J NEUROSURG, V118, P786, DOI 10.3171/2012.10.JNS112268

Tsang DS, 2015, CLIN ONCOL-UK, V27, P176, DOI 10.1016/j.clon.2014.11.026

Tsiouris A, 2013, J SURG RES, V183, P40, DOI 10.1016/j.jss.2012.11.059

van den Bent MJ, 2015, NAT REV NEUROL, V11, P374, DOI 10.1038/nrneurol.2015.82

Vives KP, 1999, J NEURO-ONCOL, V42, P289, DOI 10.1023/A:1006163328765

Vuorinen V, 2003, ACTA NEUROCHIR, V145, P5, DOI 10.1007/s00701-002-1030-6

Wiestler B, 2013, NEURO-ONCOLOGY, V15, P1017, DOI 10.1093/neuonc/not043

Wroe PC, 2012, J INFECT DIS, V205, P1589, DOI 10.1093/infdis/jis240

Yovino S, 2011, CURR TREAT OPTION ON, V12, P253, DOI 10.1007/s11864-011-0158-0

Zarnett OJ, 2015, JAMA NEUROL, V72, P589, DOI 10.1001/jamaneurol.2014.3739

NR 48

TC 4

Z9 4

U1 0

U2 7

PU ELSEVIER SCIENCE INC

PI NEW YORK

PA 360 PARK AVE SOUTH, NEW YORK, NY 10010-1710 USA

SN 1878-8750

EI 1878-8769

J9 WORLD NEUROSURG

JI World Neurosurg.

PD MAY

PY 2016

VL 89

BP 355

EP 367

DI 10.1016/j.wneu.2016.01.040

PG 13

WC Clinical Neurology; Surgery

WE Science Citation Index Expanded (SCI-EXPANDED)

SC Neurosciences & Neurology; Surgery

GA DM6GM

UT WOS:000376448700049

PM 26850972

DA 2023-06-10

ER

PT J

AU Shear, DA

Tate, MC

Archer, DR

Hoffman, SW

Hulce, VD

LaPlaca, MC

Stein, DG

AF Shear, DA

Tate, MC

Archer, DR

Hoffman, SW

Hulce, VD

LaPlaca, MC

Stein, DG

TI Neural progenitor cell transplants promote long-term functional recovery

after traumatic brain injury

SO BRAIN RESEARCH

LA English

DT Article

DE traumatic brain injury; rotorod; spatial learning; Morris water maze;

neural stem cell; NG2; oligodendrocyte progenitor cell; neurosphere

ID CENTRAL-NERVOUS-SYSTEM; STEM-CELLS; NEUROTROPHIC FACTORS; SPINAL-CORD;

RESTRICTED PRECURSORS; CLINICAL-APPLICATION; ADULT BRAIN; RAT MODEL;

DIFFERENTIATE; LESIONS

AB Studies demonstrating the versatility of neural progenitor cells (NPCs) have recently rekindled interest in neurotransplantation methods aimed at treating traumatic brain injury (TBI). However, few studies have evaluated the safety and functional efficacy of transplanted NPCs beyond a few months. The purpose of this study was to assess the long-term survival, migration, differentiation and functional significance of NPCs transplanted into a mouse model of TBI out to 1 year post-transplant. NPCs were derived from E14.5 mouse brains containing a transgene-expressing green fluorescent protein (GFP) and cultured as neurospheres in FGF2-containing medium. Neurospheres were injected into the ipsilateral striatum of adult C57BL/6 mice I week following unilateral cortical impact injury. Behavioral testing revealed significant improvements in motor abilities in NPC-treated mice as early as I week, and the recovery was sustained out to I year post-transplant. In addition, mice receiving NPC transplants showed significant improvement in spatial learning abilities at 3 months and 1 year, whereas an intermediate treatment effect on this behavioral parameter was detected at 1 month. At 14 months post-transplant, GFP(+) NPCs were observed throughout the injured hippocampus and adjacent cortical regions of transplanted brains. Immumohistochemical analysis revealed that the majority of transplanted cells co-labeled for NG2, an oligodendrocyte progenitor cell marker, but not for neuronal, astrocytic or microglial markers. In conclusion, transplanted NPCs survive in the host brain up to 14 months, migrate to the site of injury, enhance motor and cognitive recovery, and may play a role in trophic support following TBI. (C) 2004 Elsevier B.V. All rights reserved.

C1 Emory Univ, Brain Res Lab, Dept Psychol, Atlanta, GA 30322 USA.

Field Neurosci Inst, Saginaw, MI USA.

Emory Univ, Georgia Inst Technol, Dept Biomed Engn, Atlanta, GA 30322 USA.

Emory Univ, Dept Pediat, Atlanta, GA 30322 USA.

Emory Univ, Dept Emergency Med, Atlanta, GA 30322 USA.

Emory Univ, Dept Neurol, Atlanta, GA 30322 USA.

C3 Emory University; Emory University; University System of Georgia;

Georgia Institute of Technology; Emory University; Emory University;

Emory University

RP Stein, DG (通讯作者)，Emory Univ, Brain Res Lab, Dept Psychol, Evans Bldg,Room 261B,1648 Pierce Dr, Atlanta, GA 30322 USA.

EM dstei04@emory.edu

RI Hoffman, Stuart/CAF-7908-2022; Stein, Donald/AAJ-5139-2020; Shear,

Deborah A./B-3607-2011

CR Aboody KS, 2000, P NATL ACAD SCI USA, V97, P12846, DOI 10.1073/pnas.97.23.12846

AEBISCHER P, 1994, EXP NEUROL, V126, P151, DOI 10.1006/exnr.1994.1053

Benninger Y, 2000, BRAIN PATHOL, V10, P330

Bergles DE, 2000, NATURE, V405, P187, DOI 10.1038/35012083

Bjorklund LM, 2002, P NATL ACAD SCI USA, V99, P2344, DOI 10.1073/pnas.022438099

Borlongan CV, 1998, EXP NEUROL, V149, P310, DOI 10.1006/exnr.1997.6730

BORLONGAN CV, 2004, IN PRESS STROKE

Bullock MR, 1999, NEUROSURGERY, V45, P207, DOI 10.1097/00006123-199908000-00001

Butt AM, 2002, J NEUROCYTOL, V31, P551, DOI 10.1023/A:1025751900356

CHEN LS, 1991, J CELL BIOCHEM, V45, P252, DOI 10.1002/jcb.240450305

Dawson MRL, 2000, J NEUROSCI RES, V61, P471, DOI 10.1002/1097-4547(20000901)61:5<471::AID-JNR1>3.3.CO;2-E

Gage FH, 2000, SCIENCE, V287, P1433, DOI 10.1126/science.287.5457.1433

GAGE FH, 1991, NEURON, V6, P1, DOI 10.1016/0896-6273(91)90116-H

Gennarelli Thomas A., 1993, Journal of Emergency Medicine, V11, P5

Gray JA, 1999, PHILOS T R SOC B, V354, P1407, DOI 10.1098/rstb.1999.0488

Jones LL, 2002, J NEUROSCI, V22, P2792, DOI 10.1523/JNEUROSCI.22-07-02792.2002

KESSLAK JP, 1986, EXP NEUROL, V92, P377, DOI 10.1016/0014-4886(86)90089-0

Kukekov VG, 1999, EXP NEUROL, V156, P333, DOI 10.1006/exnr.1999.7028

LABBE R, 1983, SCIENCE, V221, P470, DOI 10.1126/science.6683427

Levison SW, 1997, J NEUROSCI RES, V48, P83, DOI 10.1002/(SICI)1097-4547(19970415)48:2<83::AID-JNR1>3.0.CO;2-8

Lindvall Olle, 1994, Current Opinion in Neurobiology, V4, P752, DOI 10.1016/0959-4388(94)90019-1

Liu S, 2000, P NATL ACAD SCI USA, V97, P6126, DOI 10.1073/pnas.97.11.6126

Lu DY, 2002, J NEUROSURG, V97, P935, DOI 10.3171/jns.2002.97.4.0935

Mahmood A, 2002, J NEUROTRAUM, V19, P1609, DOI 10.1089/089771502762300265

McIntosh TK, 1998, NEUROPATH APPL NEURO, V24, P251

McKay R, 1997, SCIENCE, V276, P66, DOI 10.1126/science.276.5309.66

McTigue DM, 2001, J NEUROSCI, V21, P3392, DOI 10.1523/JNEUROSCI.21-10-03392.2001

Milward EA, 1997, J NEUROSCI RES, V50, P862, DOI 10.1002/(SICI)1097-4547(19971201)50:5<862::AID-JNR22>3.0.CO;2-1

NIETOSAMPEDRO M, 1987, ANN NY ACAD SCI, V495, P108, DOI 10.1111/j.1749-6632.1987.tb23670.x

Nishiyama A, 1999, J NEUROPATH EXP NEUR, V58, P1113, DOI 10.1097/00005072-199911000-00001

Nishiyama A, 1997, J NEUROSCI RES, V48, P299, DOI 10.1002/(SICI)1097-4547(19970515)48:4<299::AID-JNR2>3.0.CO;2-6

Philips MF, 2001, J NEUROSURG, V94, P765, DOI 10.3171/jns.2001.94.5.0765

Pierce JES, 1998, NEUROSCIENCE, V87, P359, DOI 10.1016/S0306-4522(98)00142-0

Pluchino S, 2003, NATURE, V422, P688, DOI 10.1038/nature01552

Rakic S, 2003, GLIA, V41, P117, DOI 10.1002/glia.10140

Rao MS, 1999, ANAT RECORD, V257, P137

Riess P, 2002, NEUROSURGERY, V51, P1043, DOI 10.1097/00006123-200210000-00035

Roberts I, 1998, J NEUROL NEUROSUR PS, V65, P729, DOI 10.1136/jnnp.65.5.729

STEIN DG, 1988, BRAIN INJURY RECOVER, P249

Tate MC, 2002, CELL TRANSPLANT, V11, P283

Watanabe M, 2002, J NEUROSCI RES, V69, P826, DOI 10.1002/jnr.10338

Whittemore SR, 1996, MOL NEUROBIOL, V12, P13, DOI 10.1007/BF02740745

Windrem MS, 2002, J NEUROSCI RES, V69, P966, DOI 10.1002/jnr.10397

NR 43

TC 133

Z9 142

U1 0

U2 9

PU ELSEVIER SCIENCE BV

PI AMSTERDAM

PA PO BOX 211, 1000 AE AMSTERDAM, NETHERLANDS

SN 0006-8993

EI 1872-6240

J9 BRAIN RES

JI Brain Res.

PD NOV 5

PY 2004

VL 1026

IS 1

BP 11

EP 22

DI 10.1016/j.brainres.2004.07.087

PG 12

WC Neurosciences

WE Science Citation Index Expanded (SCI-EXPANDED)

SC Neurosciences & Neurology

GA 865OU

UT WOS:000224713200002

PM 15476693

DA 2023-06-10

ER

PT J

AU Jackson, ML

Srivastava, AK

Cox, CS

AF Jackson, Margaret L.

Srivastava, Amit K.

Cox, Charles S., Jr.

TI Preclinical progenitor cell therapy in traumatic brain injury: a

meta-analysis

SO JOURNAL OF SURGICAL RESEARCH

LA English

DT Article

DE Meta-analysis; Traumatic brain injury; Progenitor cells; Animal TBI

model; Preclinical studies

ID MESENCHYMAL STEM-CELLS; MARROW STROMAL CELLS; ACTIVATED

MICROGLIAL/MACROPHAGE RESPONSE; RAT MODEL; FUNCTIONAL RECOVERY;

COGNITIVE FUNCTION; TRANSPLANTATION; MICE; PATHOPHYSIOLOGY; EFFICACY

AB Background: No treatment is available to reverse injury associated with traumatic brain injury (TBI). Progenitor cell therapies show promise in both preclinical and clinical studies.We conducted a meta-analysis of preclinical studies using progenitor cells to treat TBI.

Methods: EMBASE, MEDLINE, Cochrane Review, Biosis, and Google Scholar were searched for articles using prespecified search strategies. Studies meeting inclusion criteria underwent data extraction. Analysis was performed using Review Manager 5.3 according to a fixed-effects model, and all studies underwent quality scoring.

Results: Of 430 abstracts identified, 38 met inclusion criteria and underwent analysis. Average quality score was 4.32 of 8 possible points. No study achieved a perfect score. Lesion volume (LV) and neurologic severity score (NSS) outcomes favored cell treatment with standard mean difference (SMD) of 0.86 (95% CI: 0.64-1.09) and 1.36 (95% CI: 1.11-1.60),respectively.Rotarod and Morris water maze outcomes also favored treatment with improvements in SMD of 0.34 (95% CI: 0.02-0.65) and 0.46 (95% CI: 0.17-74), respectively. Although LV and NSS were robust to publication bias assessments, rotarod and Morris water maze tests were not.Heterogeneity (I-2) ranged from 74%-85% among the analyses, indicating a high amount of heterogeneity among studies. Precision as a function of quality score showed a statistically significant increase in the size of the confidence interval as quality improved.

Conclusions: Our meta-analysis study reveals an overall positive effect of progenitor cell therapies on LV and NSS with a trend toward improved motor function and spatial learning in different TBI animal models. (C) 2017 Elsevier Inc. All rights reserved.

C1 [Jackson, Margaret L.; Srivastava, Amit K.; Cox, Charles S., Jr.] Univ Texas Hlth Sci Ctr Houston, Dept Pediat Surg, Houston, TX 77030 USA.

C3 University of Texas System; University of Texas Health Science Center

Houston

RP Jackson, ML (通讯作者)，UTHlth McGovern Med Sch, Dept Pediat Surg, 6431 Fannin St, Houston, TX 77030 USA.

EM margaret.l.jackson@uth.tmc.edu

RI Srivastava, Amit/ISB-5949-2023; Srivastava, Amit/L-7019-2019

OI Srivastava, Amit/0000-0001-7554-7201;

FU Department of Health and Human Services [5T32GM008792-13,

4T3200GM8792-14]

FX This work was supported by the Department of Health and Human Services,

grant numbers 5T32GM008792-13 and 4T3200GM8792-14.

CR Aertker BM, 2016, EXP NEUROL, V275, P411, DOI 10.1016/j.expneurol.2015.01.008

Anbari F, 2014, NEURAL REGEN RES, V9, P919, DOI 10.4103/1673-5374.133133

Arien-Zakay H, 2014, J NEUROTRAUM, V31, P1405, DOI 10.1089/neu.2013.3270

Arrowsmith J, 2013, NAT REV DRUG DISCOV, V12, P568, DOI 10.1038/nrd4090

Bakhtiary Mehrdad, 2010, Iranian Biomedical Journal, V14, P142

Barkhoudarian G, 2011, CLIN SPORT MED, V30, P33, DOI 10.1016/j.csm.2010.09.001

Bedi SS, 2013, STEM CELL TRANSL MED, V2, P953, DOI 10.5966/sctm.2013-0100

Bedi SS, 2013, J TRAUMA ACUTE CARE, V75, P410, DOI 10.1097/TA.0b013e31829617c6

Bonilla C, 2014, CYTOTHERAPY, V16, P1501, DOI 10.1016/j.jcyt.2014.07.007

Chang J, 2015, EXP NEUROL, V273, P225, DOI 10.1016/j.expneurol.2015.08.020

Chen SH, 2014, CELL TRANSPLANT, V23, P959, DOI 10.3727/096368913X667006

Cheng T, 2015, CELL MOL NEUROBIOL, V35, P641, DOI 10.1007/s10571-015-0159-9

Cole TB, 2004, JAMA-J AM MED ASSOC, V291, P2531, DOI 10.1001/jama.291.21.2531

Cox CS, 2011, NEUROSURGERY, V68, P588, DOI 10.1227/NEU.0b013e318207734c

Cox Jr CS, 2016, STEM CELLS

Egger M, 1997, BMJ-BRIT MED J, V315, P629, DOI 10.1136/bmj.315.7109.629

Fisher M, 2009, STROKE, V40, P2244, DOI 10.1161/STROKEAHA.108.541128

Fu XM, 2015, CELL TRANSPLANT, V24, P1533, DOI 10.3727/096368914X679345

Gao JL, 2006, EXP NEUROL, V201, P281, DOI 10.1016/j.expneurol.2006.04.039

Gibb SL, 2015, STEM CELLS, V33, P3530, DOI 10.1002/stem.2189

Goodman JC, 2008, ACTA NEUROCHIR SUPPL, V102, P437, DOI 10.1007/978-3-211-85578-2_85

Han EY, 2013, CURR STEM CELL RES T, V8, P172

Harting MT, 2009, J NEUROSURG, V110, P1189, DOI 10.3171/2008.9.JNS08158

Harting MT, 2009, J SURG RES, V153, P188, DOI 10.1016/j.jss.2008.03.037

Higgins JP, 2010, COLLABORATION SMGOTC

HIGGINS JPT, 2011, COCHRANE HDB SYSTEMA, V0001

Hoane MR, 2004, J NEUROTRAUM, V21, P163, DOI 10.1089/089771504322778622

Hong SQ, 2011, NEUROCHEM RES, V36, P2391, DOI 10.1007/s11064-011-0567-2

Jiang JD, 2012, NEURAL REGEN RES, V7, P46, DOI 10.3969/j.issn.1673-5374.2012.01.008

Johnson VE, 2013, EXP NEUROL, V246, P35, DOI 10.1016/j.expneurol.2012.01.013

Kilkenny C, 2009, PLOS ONE, V4, DOI 10.1371/journal.pone.0007824

Kim HJ, 2010, J NEUROTRAUM, V27, P131, DOI [10.1089/neu.2008.0818, 10.1089/neu.2008-0818]

Landis SC, 2012, NATURE, V490, P187, DOI 10.1038/nature11556

Li LA, 2011, J NEUROTRAUM, V28, P535, DOI 10.1089/neu.2010.1619

Liao GP, 2015, PEDIATR CRIT CARE ME, V16, P245, DOI 10.1097/PCC.0000000000000324

Liao GP, 2014, J SURG RES, V190, P628, DOI 10.1016/j.jss.2014.05.011

Lu D, 2001, NEUROREPORT, V12, P559, DOI 10.1097/00001756-200103050-00025

Lu DY, 2002, CELL TRANSPLANT, V11, P275

Maas AIR, 2008, LANCET NEUROL, V7, P728, DOI 10.1016/S1474-4422(08)70164-9

Mahmood A, 2004, NEUROSURGERY, V55, P1185, DOI 10.1227/01.NEU.0000141042.14476.3C

Mahmood A, 2003, NEUROSURGERY, V53, P697, DOI 10.1227/01.NEU.0000079333.61863.AA

MAHMOOD A, 2003, NEUROSURGERY, V53, P702

Mahmood A, 2013, J NEUROSURG, V118, P381, DOI 10.3171/2012.11.JNS12753

McIntosh TK, 1996, LAB INVEST, V74, P315

Menge T, 2012, SCI TRANSL MED, V4, DOI 10.1126/scitranslmed.3004660

Moher D, 2008, BMC MED, V6, DOI 10.1186/1741-7015-6-13

Morganti-Kossmann MC, 2007, INJURY, V38, P1392, DOI 10.1016/j.injury.2007.10.005

Muthuraju S, 2012, BASAL GANGLIA, V2, P143, DOI 10.1016/j.baga.2012.06.004

Muthuraju S, 2013, J BIOSCIENCES, V38, P93, DOI 10.1007/s12038-012-9290-7

Nichols JE, 2013, STEM CELL RES THER, V4, DOI 10.1186/scrt151

Osanai T, 2012, NEUROSURGERY, V70, P435, DOI 10.1227/NEU.0b013e318230a795

Peng WJ, 2015, STEM CELL RES THER, V6, DOI 10.1186/s13287-015-0034-0

Pischiutta F, 2014, NEUROPHARMACOLOGY, V79, P119, DOI 10.1016/j.neuropharm.2013.11.001

Prins M, 2013, DIS MODEL MECH, V6, P1307, DOI 10.1242/dmm.011585

Prinz F, 2011, NAT REV DRUG DISCOV, V10, P712, DOI 10.1038/nrd3439-c1

Riess P, 2002, NEUROSURGERY, V51, P1043, DOI 10.1097/00006123-200210000-00035

Riess P, 2007, J NEUROTRAUM, V24, P216, DOI 10.1089/neu.2006.0141

Shear DA, 2004, BRAIN RES, V1026, P11, DOI 10.1016/j.brainres.2004.07.087

Silachev DN, 2015, B EXP BIOL MED+, V159, P528, DOI 10.1007/s10517-015-3009-3

Sterne JAC, 2011, BMJ-BRIT MED J, V343, DOI 10.1136/bmj.d4002

Tajiri N, 2014, J NEUROSCI, V34, P313, DOI 10.1523/JNEUROSCI.2425-13.2014

Turtzo LC, 2015, PLOS ONE, V10, DOI 10.1371/journal.pone.0126551

van der Worp HB, 2011, J MOL CELL CARDIOL, V51, P449, DOI 10.1016/j.yjmcc.2011.04.008

Walker PA, 2010, EXP NEUROL, V225, P341, DOI 10.1016/j.expneurol.2010.07.005

Walker PA, 2010, STEM CELLS DEV, V19, P867, DOI 10.1089/scd.2009.0188

Walker PA, 2009, DIS MODEL MECH, V2, P23, DOI 10.1242/dmm.001198

Watanabe J, 2013, NEUROBIOL DIS, V59, P86, DOI 10.1016/j.nbd.2013.06.017

Werner C, 2007, BRIT J ANAESTH, V99, P4, DOI 10.1093/bja/aem131

Wolf JA, 2001, J NEUROSCI, V21, P1923, DOI 10.1523/JNEUROSCI.21-06-01923.2001

Xu HS, 2014, EUR REV MED PHARMACO, V18, P520

Xue S, 2010, NEUROSCI LETT, V473, P186, DOI 10.1016/j.neulet.2010.02.035

Zanier ER, 2011, CRIT CARE MED, V39, P2501, DOI 10.1097/CCM.0b013e31822629ba

Zhang R, 2013, J NEUROINFLAMM, V10, DOI 10.1186/1742-2094-10-106

Zhao J, 2012, J NEUROTRAUM, V29, P1209, DOI 10.1089/neu.2011.1858

Zhao JJ, 2012, NEURAL REGEN RES, V7, P741, DOI 10.3969/j.issn.1673-5374.2012.10.004

Zhao YH, 2016, STEM CELLS, V34, P1263, DOI 10.1002/stem.2310

NR 76

TC 16

Z9 16

U1 0

U2 6

PU ACADEMIC PRESS INC ELSEVIER SCIENCE

PI SAN DIEGO

PA 525 B ST, STE 1900, SAN DIEGO, CA 92101-4495 USA

SN 0022-4804

EI 1095-8673

J9 J SURG RES

JI J. Surg. Res.

PD JUN 15

PY 2017

VL 214

BP 38

EP 48

DI 10.1016/j.jss.2017.02.078

PG 11

WC Surgery

WE Science Citation Index Expanded (SCI-EXPANDED)

SC Surgery

GA FA3RX

UT WOS:000405363200006

PM 28624058

OA Green Accepted

DA 2023-06-10

ER

PT J

AU Jahan-Abad, AJ

Negah, SS

Ravandi, HH

Ghasemi, S

Borhani-Haghighi, M

Stummer, W

Gorji, A

Ghadiri, MK

AF Jahan-Abad, Ali Jahanbazi

Negah, Sajad Sahab

Ravandi, Hassan Hosseini

Ghasemi, Sedigheh

Borhani-Haghighi, Maryam

Stummer, Walter

Gorji, Ali

Ghadiri, Maryam Khaleghi

TI Human Neural Stem/Progenitor Cells Derived From Epileptic Human Brain in

a Self-Assembling Peptide Nanoscaffold Improve Traumatic Brain Injury in

Rats

SO MOLECULAR NEUROBIOLOGY

LA English

DT Article

DE Tissue engineering; Human neural stem cells; Traumatic brain injury;

Epilepsy; Inflammation

ID ADULT HUMAN BRAIN; MESENCHYMAL STEM-CELLS; TEMPORAL-LOBE EPILEPSY;

MARROW STROMAL CELLS; PROGENITOR CELLS; IN-VITRO; NEURONAL

DIFFERENTIATION; PRECURSOR CELLS; SUBVENTRICULAR ZONE; TRANSPLANTATION

AB Traumatic brain injury (TBI) is a disruption in the brain functions following a head trauma. Cell therapy may provide a promising treatment for TBI. Among different cell types, human neural stem cells cultured in self-assembling peptide scaffolds have been suggested as a potential novel method for cell replacement treatment after TBI. In the present study, we accessed the effects of human neural stem/progenitor cells (hNS/PCs) derived from epileptic human brain and human adipose-derived stromal/stem cells (hADSCs) seeded in PuraMatrix hydrogel (PM) on brain function after TBI in an animal model of brain injury. hNS/PCs were isolated from patients with medically intractable epilepsy undergone epilepsy surgery. hNS/PCs and hADSCs have the potential for proliferation and differentiation into both neuronal and glial lineages. Assessment of the growth characteristics of hNS/PCs and hADSCs revealed that the hNS/PCs doubling time was significantly longer and the growth rate was lower than hADSCs. Transplantation of hNS/PCs and hADSCs seeded in PM improved functional recovery, decreased lesion volume, inhibited neuroinflammation, and reduced the reactive gliosis at the injury site. The data suggest the transplantation of hNS/PCs or hADSCs cultured in PM as a promising treatment option for cell replacement therapy in TBI.

C1 [Jahan-Abad, Ali Jahanbazi; Negah, Sajad Sahab; Ravandi, Hassan Hosseini; Ghasemi, Sedigheh; Borhani-Haghighi, Maryam; Gorji, Ali] Khatam Alanbia Hosp, Shefa Neurosci Res Ctr, Tehran, Iran.

[Jahan-Abad, Ali Jahanbazi] Shahid Beheshti Univ Med Sci, Dept Clin Biochem, Tehran, Iran.

[Negah, Sajad Sahab; Gorji, Ali] Mashhad Univ Med Sci, Dept Neurosci, Fac Med, Mashhad, Iran.

[Stummer, Walter; Gorji, Ali; Ghadiri, Maryam Khaleghi] Westfalische Wilhelms Univ Munster, Dept Neurosurg, Munster, Germany.

[Gorji, Ali] Westfalische Wilhelms Univ Munster, Dept Neurol, Munster, Germany.

[Gorji, Ali] Westfalische Wilhelms Univ Munster, Epilepsy Res Ctr, Robert Koch Str 45, D-48149 Munster, Germany.

C3 Shahid Beheshti University Medical Sciences; Mashhad University Medical

Science; University of Munster; University of Munster; University of

Munster

RP Gorji, A (通讯作者)，Khatam Alanbia Hosp, Shefa Neurosci Res Ctr, Tehran, Iran.; Gorji, A (通讯作者)，Mashhad Univ Med Sci, Dept Neurosci, Fac Med, Mashhad, Iran.; Gorji, A (通讯作者)，Westfalische Wilhelms Univ Munster, Dept Neurosurg, Munster, Germany.; Gorji, A (通讯作者)，Westfalische Wilhelms Univ Munster, Dept Neurol, Munster, Germany.; Gorji, A (通讯作者)，Westfalische Wilhelms Univ Munster, Epilepsy Res Ctr, Robert Koch Str 45, D-48149 Munster, Germany.

EM gorjial@uni-muenster.de

RI Jahan-Abad, Ali Jahanbazi/AAW-3641-2021; Stummer, Walter/AAF-9043-2019;

Gorji, Ahmad Mousapour/D-3670-2017; borhani, maryam/AGQ-4261-2022;

Stummer, Walter/AAA-3319-2020; Negah, Sajad Sahab/AAQ-5852-2020

OI Jahan-Abad, Ali Jahanbazi/0000-0001-8629-7762; Gorji,

Ali/0000-0002-4557-3270; Borhani-Haghighi, Maryam/0000-0003-0308-2948;

naseri, shahrokh/0000-0001-8974-2120

FU Iran National Science Foundation (INSF); National Institute for Medical

Research Development; German Academic Exchange Service (DAAD) [57348208,

57403633]

FX This study was supported by the Iran National Science Foundation (INSF),

the National Institute for Medical Research Development, and the German

Academic Exchange Service (DAAD; 57348208 and 57403633) to AG.

CR Abraham R, 2012, PROG BRAIN RES, V201, P17, DOI 10.1016/B978-0-444-59544-7.00002-0

Aligholi H, 2016, BRAIN RES, V1642, P197, DOI 10.1016/j.brainres.2016.03.043

Aligholi H, 2014, J NEUROSCI METH, V225, P81, DOI 10.1016/j.jneumeth.2013.12.008

Ayuso-Sacido A, 2008, NEUROSURGERY, V62, P223, DOI 10.1227/01.NEU.0000311081.50648.4C

Azevedo-Pereira RL, 2010, ARQ NEURO-PSIQUIAT, V68, P956, DOI 10.1590/S0004-282X2010000600023

Beretta S, 2017, CELL TRANSPLANT, V26, P1247, DOI 10.1177/0963689717714107

Bliss T, 2007, STROKE, V38, P817, DOI 10.1161/01.STR.0000247888.25985.62

Blumcke I, 2001, HIPPOCAMPUS, V11, P311, DOI 10.1002/hipo.1045

Buhnemann C, 2006, BRAIN, V129, P3238, DOI 10.1093/brain/awl261

Campbell John N, 2014, Int J Neurol Brain Disord, V1, P1

Cavalcanti BN, 2013, DENT MATER, V29, P97, DOI 10.1016/j.dental.2012.08.002

Chen JL, 2001, STROKE, V32, P1005, DOI 10.1161/01.STR.32.4.1005

Corrigan F, 2016, J NEUROINFLAMM, V13, DOI 10.1186/s12974-016-0738-9

Coulthard LG, 2017, J NEUROSCI, V37, P5395, DOI 10.1523/JNEUROSCI.0525-17.2017

Covacu R, 2017, NEUROSCIENTIST, V23, P27, DOI 10.1177/1073858415616559

Daadi MM, 2010, STROKE, V41, P516, DOI 10.1161/STROKEAHA.109.573691

Dobrowolski S., 2013, AM J NEUROSCIENCE, V4, P13, DOI DOI 10.3844/AMJNSP.2013.13.24

Drury JL, 2003, BIOMATERIALS, V24, P4337, DOI 10.1016/S0142-9612(03)00340-5

Francis NL, 2016, ACS BIOMATER SCI ENG, V2, P1030, DOI 10.1021/acsbiomaterials.6b00156

FREED LE, 1994, BIO-TECHNOL, V12, P689, DOI 10.1038/nbt0794-689

Guan J, 2013, BIOMATERIALS, V34, P5937, DOI 10.1016/j.biomaterials.2013.04.047

Guo JS, 2009, NANOMED-NANOTECHNOL, V5, P345, DOI 10.1016/j.nano.2008.12.001

Gupta K, 2013, NEUROSCI LETT, V543, P95, DOI 10.1016/j.neulet.2013.03.010

Hansson A, 2017, TISSUE CELL, V49, P35, DOI 10.1016/j.tice.2016.12.004

Hunt RF, 2015, CSH PERSPECT MED, V5, DOI 10.1101/cshperspect.a022376

Jahan-Abad AJ, 2018, MOL NEUROBIOL, V55, P4225, DOI 10.1007/s12035-017-0642-z

Jahanbazi Jahan-Abad A, 2017, AVICENNA J PHYTOMED, P1

Jassam YN, 2017, NEURON, V95, P1246, DOI 10.1016/j.neuron.2017.07.010

Joo KM, 2013, EXP NEUROL, V240, P168, DOI 10.1016/j.expneurol.2012.11.021

Karve IP, 2016, BRIT J PHARMACOL, V173, P692, DOI 10.1111/bph.13125

KIRSCHENBAUM B, 1994, CEREB CORTEX, V4, P576, DOI 10.1093/cercor/4.6.576

Kizil C, 2015, EMBO REP, V16, P416, DOI 10.15252/embr.201439702

Knight VB, 2017, PEERJ, V5, DOI 10.7717/peerj.2829

Krabbe C, 2005, APMIS, V113, P831, DOI 10.1111/j.1600-0463.2005.apm_3061.x

Li X., 2017, J BIOMEDICAL MAT R A, V106, P1082

Liedmann A, 2012, BIORESEARCH OPEN ACC, V1, P16, DOI 10.1089/biores.2012.0209

Liu SJ, 2014, J NEUROINFLAMM, V11, DOI 10.1186/1742-2094-11-66

Lu DY, 2001, J NEUROTRAUM, V18, P813, DOI 10.1089/089771501316919175

Lucke-Wold BP, 2015, SEIZURE-EUR J EPILEP, V33, P13, DOI 10.1016/j.seizure.2015.10.002

Lutolf MP, 2005, NAT BIOTECHNOL, V23, P47, DOI 10.1038/nbt1055

Ma PX, 2008, ADV DRUG DELIVER REV, V60, P184, DOI 10.1016/j.addr.2007.08.041

Moe MC, 2005, BRAIN, V128, P2189, DOI 10.1093/brain/awh574

Moradi F, 2012, J NEUROSCI RES, V90, P2335, DOI 10.1002/jnr.23120

Nam H, 2015, WORLD J STEM CELLS, V7, P126, DOI 10.4252/wjsc.v7.i1.126

Negah SS, 2018, CELL TISSUE RES, V371, P223, DOI 10.1007/s00441-017-2717-6

Negah SS, 2017, MOL NEUROBIOL, V54, P8050, DOI 10.1007/s12035-016-0295-3

Negah SS, 2016, IRAN J BASIC MED SCI, V19, P1271, DOI 10.22038/ijbms.2016.7907

Nunes MC, 2003, NAT MED, V9, P439, DOI 10.1038/nm837

Olstorn H, 2007, NEUROSURGERY, V60, P1089, DOI 10.1227/01.NEU.0000255461.91892.0D

Philips MF, 2001, J NEUROSURG, V94, P765, DOI 10.3171/jns.2001.94.5.0765

Pincus D W, 1997, Clin Neurosurg, V44, P17

Ringe J, 2002, NATURWISSENSCHAFTEN, V89, P338, DOI 10.1007/s00114-002-0344-9

Shear DA, 2004, BRAIN RES, V1026, P11, DOI 10.1016/j.brainres.2004.07.087

Shen LH, 2007, STROKE, V38, P2150, DOI 10.1161/STROKEAHA.106.481218

Shetty AK, 2011, NEUROTHERAPEUTICS, V8, P721, DOI 10.1007/s13311-011-0064-y

Shi W, 2016, ACTA BIOMATER, V45, P247, DOI 10.1016/j.actbio.2016.09.001

Shindo T, 2006, J MED INVESTIG, V53, P42, DOI 10.2152/jmi.53.42

Sun D, 2016, NEURAL REGEN RES, V11, P18, DOI 10.4103/1673-5374.169605

Takei J., 2006, AATEX, V11, P170, DOI DOI 10.11232/aatex.11.170

Tang CK, 2009, INT J MOL SCI, V10, P2136, DOI 10.3390/ijms10052136

Thonhoff JR, 2008, BRAIN RES, V1187, P42, DOI 10.1016/j.brainres.2007.10.046

Uemura M, 2010, J NEUROSCI RES, V88, P542, DOI 10.1002/jnr.22223

Varghese M, 2008, NEUROSURGERY, V63, P1022, DOI 10.1227/01.NEU.0000335792.85142.B0

Walton NM, 2006, DEVELOPMENT, V133, P3671, DOI 10.1242/dev.02541

Weick JP, 2016, STEM CELLS INT, V2016, DOI 10.1155/2016/4190438

Woodcock T, 2013, FRONT NEUROL, V4, DOI 10.3389/fneur.2013.00018

Wu HM, 2017, BIOMED RES INT-UK, V2017, DOI 10.1155/2017/3656193

Xue S, 2010, NEUROSCI LETT, V473, P186, DOI 10.1016/j.neulet.2010.02.035

Yan ZJ, 2013, NEUROCHEM RES, V38, P1022, DOI 10.1007/s11064-013-1012-5

Yang LQ, 2011, NEUROREPORT, V22, P370, DOI 10.1097/WNR.0b013e3283469615

Zhang L, 2017, NEURAL REGEN RES, V12, P1103, DOI 10.4103/1673-5374.211189

Zhang R, 2013, J NEUROINFLAMM, V10, DOI 10.1186/1742-2094-10-106

Zhang S, 2005, TISSUE ENG, P217

Zhang SG, 2005, SEMIN CANCER BIOL, V15, P413, DOI 10.1016/j.semcancer.2005.05.007

ZHANG SG, 1993, P NATL ACAD SCI USA, V90, P3334, DOI 10.1073/pnas.90.8.3334

Zhao YN, 2009, TISSUE ENG PT A, V15, P13, DOI 10.1089/ten.tea.2008.0039

Zhong J, 2010, NEUROREHAB NEURAL RE, V24, P636, DOI 10.1177/1545968310361958

Zhou FW, 2015, PLOS ONE, V10, DOI 10.1371/journal.pone.0120281

NR 78

TC 32

Z9 33

U1 1

U2 39

PU SPRINGER

PI NEW YORK

PA 233 SPRING ST, NEW YORK, NY 10013 USA

SN 0893-7648

EI 1559-1182

J9 MOL NEUROBIOL

JI Mol. Neurobiol.

PD DEC

PY 2018

VL 55

IS 12

BP 9122

EP 9138

DI 10.1007/s12035-018-1050-8

PG 17

WC Neurosciences

WE Science Citation Index Expanded (SCI-EXPANDED)

SC Neurosciences & Neurology

GA GY3VF

UT WOS:000448483400026

PM 29651746

DA 2023-06-10

ER

PT J

AU Harting, MT

Sloan, LE

Jimenez, F

Baumgartner, J

Cox, CS

AF Harting, Matthew T.

Sloan, LeeAnn E.

Jimenez, Fernando

Baumgartner, James

Cox, Charles S., Jr.

TI Subacute Neural Stem Cell Therapy for Traumatic Brain Injury

SO JOURNAL OF SURGICAL RESEARCH

LA English

DT Article

DE traumatic brain injury; neural stem cells; cellular therapy

ID COGNITIVE FUNCTION; TRANSPLANTATION; SURVIVAL; RATS; DIFFERENTIATION;

MIGRATION; CYTOKINES; RECOVERY; MEMORY; MOTOR

AB Introduction. Traumatic brain injury (TBI) frequently results in devastating and prolonged morbidity. Cellular therapy is a burgeoning field of experimental treatment that has shown promise in the management of many diseases, including TBI. Previous work suggests that certain stem and progenitor cell populations migrate to sites of inflammation and improve functional outcome in rodents after neural injury. Unfortunately, recent study has revealed potential limitations of acute and intravenous stem cell therapy. We studied subacute, direct intracerebral neural stem and progenitor cell (NSC) therapy for TBI.

Materials and methods. The NSCs were characterized by flow cytometry and placed (400,000 cells in 50 mu L 1 x phosphate-buffered saline) into and around the direct injury area, using stereotactic guidance, of female Sprague Dawley rats 1 wk after undergoing a controlled cortical impact injury. Immunohistochemistry was used to identify cells located in the brain at 48 h and 2 wk after administration. Motor function was assessed using the neurological severity score, foot fault, rotarod, and beam balance. Cognitive function was assessed using the Morris water maze learning paradigm. Repeated measures analysis of variance with post-hoc analysis were used to determine significance at P < 0.05.

Results. Immunohistochemistry analysis revealed that 1.4-1.9% of infused cells remained in the neural tissue at 48 h and 2 wk post placement. Nearly all cells were located along injection tracks at 48 h. At 2 wk some cell dispersion was apparent. Rotarod motor testing revealed significant increases in maximal speed among NSC-treated rats compared with saline controls at d 4 (36.4 versus 27.1 rpm, P < 0.05) and 5 (35.8 versus 28.9 rpm, P < 0.05). All other motor and cognitive evaluations were not significantly different compared to controls.

Conclusions. Placement of NSCs led to the cells incorporating and remaining in the tissues 2 wk after placement. Motor function tests revealed improvements in the ability to run on a rotating rod; however, other motor and cognitive functions were not significantly improved by NSC therapy. Further examination of a dose response and optimization of placement strategy may improve long-term cell survival and maximize functional recovery. (C) 2009 Elsevier Inc. All rights reserved.

C1 [Harting, Matthew T.; Sloan, LeeAnn E.; Jimenez, Fernando; Cox, Charles S., Jr.] Univ Texas Houston, Sch Med, Dept Pediat Surg, Houston, TX 77030 USA.

[Baumgartner, James; Cox, Charles S., Jr.] Childrens Mem Hermann Hosp, Houston, TX USA.

C3 University of Texas System; University of Texas Health Science Center

Houston

RP Cox, CS (通讯作者)，Univ Texas Houston, Sch Med, Dept Pediat Surg, 6431 Fannin St,MSB 5-254, Houston, TX 77030 USA.

EM charles.s.cox@uth.tmc.edu

OI Harting, Matthew/0000-0002-8929-8311

FU Children's Memorial Hermann Hospital Foundation; Texas Higher Education

Coordinating Board; [T32 GM008792-06]; [MO1 RR 02558]; [R21 HD 04

2659-01Al]

FX This work was supported by Grants T32 GM008792-06 (M.T.H.), MO1 RR

02558, R21 HD 04 2659-01Al, and the Children's Memorial Hermann Hospital

Foundation and Texas Higher Education Coordinating Board.

CR Allan SM, 2000, ANN NY ACAD SCI, V917, P84

Arvin B, 1996, NEUROSCI BIOBEHAV R, V20, P445, DOI 10.1016/0149-7634(95)00026-7

Bakshi A, 2006, EUR J NEUROSCI, V23, P2119, DOI 10.1111/j.1460-9568.2006.04743.x

Doppenberg EMR, 2004, J NEUROSURG ANESTH, V16, P87, DOI 10.1097/00008506-200401000-00019

Fujimoto ST, 2004, NEUROSCI BIOBEHAV R, V28, P365, DOI 10.1016/j.neubiorev.2004.06.002

Gao JL, 2006, EXP NEUROL, V201, P281, DOI 10.1016/j.expneurol.2006.04.039

Guzowski JF, 1997, P NATL ACAD SCI USA, V94, P2693, DOI 10.1073/pnas.94.6.2693

HARTING MT, 2008, J SURG RES, V144, P425

Keel M, 2005, INJURY, V36, P691, DOI 10.1016/j.injury.2004.12.037

Kelly S, 2004, P NATL ACAD SCI USA, V101, P11839, DOI 10.1073/pnas.0404474101

KRAUS JF, 1987, PEDIATRICS, V79, P501

Lighthall JW, 1988, J NEUROTRAUM, V5, P1, DOI 10.1089/neu.1988.5.1

Mahmood A, 2001, NEUROSURGERY, V49, P1196, DOI 10.1097/00006123-200111000-00031

Molcanyi M, 2007, J NEUROTRAUM, V24, P625, DOI 10.1089/neu.2006.0180

Morganti-Kossmann Maria Cristina, 2002, Curr Opin Crit Care, V8, P101

Okano H, 2002, J NEUROSCI RES, V69, P698, DOI 10.1002/jnr.10343

Ragnarsson KT, 1999, JAMA-J AM MED ASSOC, V282, P974

Riess P, 2002, NEUROSURGERY, V51, P1043, DOI 10.1097/00006123-200210000-00035

SCHENK F, 1985, EXP BRAIN RES, V58, P11

Shear DA, 2004, BRAIN RES, V1026, P11, DOI 10.1016/j.brainres.2004.07.087

Shindo T, 2006, J MED INVESTIG, V53, P42, DOI 10.2152/jmi.53.42

Shohami E, 1999, CYTOKINE GROWTH F R, V10, P119, DOI 10.1016/S1359-6101(99)00008-8

Sinson G, 1996, J NEUROSURG, V84, P655, DOI 10.3171/jns.1996.84.4.0655

Tate MC, 2002, CELL TRANSPLANT, V11, P283

Wennersten A, 2004, J NEUROSURG, V100, P88, DOI 10.3171/jns.2004.100.1.0088

Zhang C, 2005, J NEUROTRAUM, V22, P1456, DOI 10.1089/neu.2005.22.1456

NR 26

TC 71

Z9 79

U1 0

U2 8

PU ACADEMIC PRESS INC ELSEVIER SCIENCE

PI SAN DIEGO

PA 525 B ST, STE 1900, SAN DIEGO, CA 92101-4495 USA

SN 0022-4804

EI 1095-8673

J9 J SURG RES

JI J. Surg. Res.

PD MAY 15

PY 2009

VL 153

IS 2

BP 188

EP 194

DI 10.1016/j.jss.2008.03.037

PG 7

WC Surgery

WE Science Citation Index Expanded (SCI-EXPANDED)

SC Surgery

GA 438VX

UT WOS:000265585100002

PM 18694578

OA Green Accepted

DA 2023-06-10

ER

PT J

AU Zhou, YX

Shao, AW

Xu, WL

Wu, HJ

Deng, YC

AF Zhou, Yunxiang

Shao, Anwen

Xu, Weilin

Wu, Haijian

Deng, Yongchuan

TI Advance of Stem Cell Treatment for Traumatic Brain Injury

SO FRONTIERS IN CELLULAR NEUROSCIENCE

LA English

DT Review

DE traumatic brain injury; stem cell; mechanism; treatment; review

ID ENDOTHELIAL PROGENITOR CELLS; INDUCED PLURIPOTENT STEM; ACTIVATED

MICROGLIAL/MACROPHAGE RESPONSE; MESENCHYMAL STROMAL CELLS; NEUROVASCULAR

PLASTICITY; PROMOTES ANGIOGENESIS; FUNCTIONAL OUTCOMES; TRANSPLANTATION;

THERAPY; IMPROVES

AB Traumatic brain injury (TBI) is an important cause of human mortality and morbidity, which can induce serious neurological damage. At present, clinical treatments for neurological dysfunction after TBI include hyperbaric oxygen, brain stimulation and behavioral therapy, but the therapeutic effect is not satisfactory. Recent studies have found that exogenous stem cells can migrate to damaged brain tissue, then participate in the repair of damaged brain tissue by further differentiation to replace damaged cells, while releasing anti-inflammatory factors and growth factors, thereby significantly improving neurological function. This article will mainly review the effects, deficiencies and related mechanisms of different types of stem cells in TBI.

C1 [Zhou, Yunxiang; Deng, Yongchuan] Zhejiang Univ, Affiliated Hosp 2, Sch Med, Dept Surg Oncol, Hangzhou, Zhejiang, Peoples R China.

[Shao, Anwen; Xu, Weilin; Wu, Haijian] Zhejiang Univ, Affiliated Hosp 2, Sch Med, Dept Neurosurg, Hangzhou, Zhejiang, Peoples R China.

C3 Zhejiang University; Zhejiang University

RP Deng, YC (通讯作者)，Zhejiang Univ, Affiliated Hosp 2, Sch Med, Dept Surg Oncol, Hangzhou, Zhejiang, Peoples R China.; Shao, AW (通讯作者)，Zhejiang Univ, Affiliated Hosp 2, Sch Med, Dept Neurosurg, Hangzhou, Zhejiang, Peoples R China.

EM 21118116@zju.edu.cn; dyc001@zju.edu.cn

RI Shao, Anwen/GRK-0113-2022; Shao, Anwen/R-9023-2019; Zhou,

Yunxiang/AAK-7758-2020

OI Zhou, Yunxiang/0000-0003-1000-0330; Shao, Anwen/0000-0001-9986-6290

FU China Postdoctoral Science Foundation [2017M612010]; National Natural

Science Foundation of China [81701144]

FX This work was funded by China Postdoctoral Science Foundation

(2017M612010) and National Natural Science Foundation of China

(81701144).

CR Adibi A, 2016, WORLD NEUROSURG, V86, P390, DOI 10.1016/j.wneu.2015.10.082

An MC, 2012, CELL STEM CELL, V11, P253, DOI 10.1016/j.stem.2012.04.026

Barteneva NS, 2013, FRONT CELL INFECT MI, V3, DOI 10.3389/fcimb.2013.00049

Bedi SS, 2013, STEM CELL TRANSL MED, V2, P953, DOI 10.5966/sctm.2013-0100

Bedi SS, 2013, J TRAUMA ACUTE CARE, V75, P410, DOI 10.1097/TA.0b013e31829617c6

Boyer-Di Ponio J, 2014, PLOS ONE, V9, DOI 10.1371/journal.pone.0084179

Carmeliet P, 2005, NATURE, V436, P193, DOI 10.1038/nature03875

Cary WA, 2015, WORLD NEUROSURG, V84, P1256, DOI 10.1016/j.wneu.2015.05.076

Chen X, 2013, ACTA RADIOL, V54, P313, DOI 10.1258/ar.2012.120605

Cox CS, 2018, PEDIATR RES, V83, P325, DOI 10.1038/pr.2017.253

Meirelles LDS, 2006, J CELL SCI, V119, P2204, DOI 10.1242/jcs.02932

Dang BQ, 2017, NEURAL PLAST, V2017, DOI 10.1155/2017/1582182

Dekmak A, 2018, BEHAV BRAIN RES, V340, P49, DOI 10.1016/j.bbr.2016.12.039

Djouad F, 2003, BLOOD, V102, P3837, DOI 10.1182/blood-2003-04-1193

Dunkerson J, 2014, RESTOR NEUROL NEUROS, V32, P675, DOI 10.3233/RNN-140408

Escudero CA, 2016, FRONT PHYSIOL, V7, DOI 10.3389/fphys.2016.00098

Gao X, 2016, SCI REP-UK, V6, DOI 10.1038/srep22490

Ghobrial GM, 2017, NEUROSURGERY, V64, P87, DOI 10.1093/neuros/nyx242

Gold EM, 2013, REGEN MED, V8, P483, DOI 10.2217/rme.13.41

Guo SW, 2017, NEUROPSYCH DIS TREAT, V13, P2757, DOI 10.2147/NDT.S141534

Guo XB, 2017, SCI REP-UK, V7, DOI 10.1038/s41598-017-04153-2

Guo XB, 2009, J NEUROTRAUM, V26, P1337, DOI [10.1089/neu.2008.0733, 10.1089/neu.2008-0733]

Haus DL, 2016, EXP NEUROL, V281, P1, DOI 10.1016/j.expneurol.2016.04.008

Huang XT, 2013, J NEUROTRAUM, V30, P2080, DOI 10.1089/neu.2013.2996

Kobayashi Y, 2012, PLOS ONE, V7, DOI 10.1371/journal.pone.0052787

Lin Y, 2000, J CLIN INVEST, V105, P71, DOI 10.1172/JCI8071

Lin YP, 2017, AGING DIS, V8, P115, DOI 10.14336/AD.2016.0610

Lyu Q, 2017, CELL TRANSPLANT, V26, P1622, DOI 10.1177/0963689717723014

Maas AIR, 2017, LANCET NEUROL, V16, P987, DOI 10.1016/S1474-4422(17)30371-X

Malinovskaya NA, 2016, FRONT PHYSIOL, V7, DOI 10.3389/fphys.2016.00599

Park KJ, 2014, J CEREBR BLOOD F MET, V34, P357, DOI 10.1038/jcbfm.2013.216

Philips MF, 2001, J NEUROSURG, V94, P765, DOI 10.3171/jns.2001.94.5.0765

Ran QS, 2015, NEURAL REGEN RES, V10, P1258, DOI 10.4103/1673-5374.162758

Reis C, 2017, STEM CELLS INT, V2017, DOI 10.1155/2017/6392592

Roobrouck VD, 2011, STEM CELLS, V29, P871, DOI 10.1002/stem.633

Sanchez-Ramos J, 2000, EXP NEUROL, V164, P247, DOI 10.1006/exnr.2000.7389

Shi XD, 2018, CELL TISSUE RES, V372, P67, DOI 10.1007/s00441-017-2716-7

Takahashi K, 2006, CELL, V126, P663, DOI 10.1016/j.cell.2006.07.024

Walker PA, 2012, J NEUROINFLAMM, V9, DOI 10.1186/1742-2094-9-228

Walker PA, 2010, EXP NEUROL, V225, P341, DOI 10.1016/j.expneurol.2010.07.005

Wallenquist U, 2009, RESTOR NEUROL NEUROS, V27, P323, DOI 10.3233/RNN-2009-0481

Wang L, 2015, TRANSL STROKE RES, V6, P50, DOI 10.1007/s12975-014-0362-x

Wang SY, 2013, INT J DEV NEUROSCI, V31, P30, DOI 10.1016/j.ijdevneu.2012.09.004

Wang ZG, 2017, EXP THER MED, V13, P3613, DOI 10.3892/etm.2017.4423

Wei ZZ, 2016, CELL TRANSPLANT, V25, P797, DOI 10.3727/096368916X690403

Wong SP, 2015, PHARMACOL THERAPEUT, V151, P107, DOI 10.1016/j.pharmthera.2015.03.006

Wu XY, 2013, PLOS ONE, V8, DOI 10.1371/journal.pone.0079975

Xin HQ, 2013, J CEREBR BLOOD F MET, V33, P1711, DOI 10.1038/jcbfm.2013.152

Xiong Y, 2008, BRAIN RES, V1230, P247, DOI 10.1016/j.brainres.2008.06.127

Xiong Y, 2017, NEURAL REGEN RES, V12, P19, DOI 10.4103/1673-5374.198966

Xue S, 2010, NEUROSCI LETT, V473, P186, DOI 10.1016/j.neulet.2010.02.035

Yeo RWY, 2013, ADV DRUG DELIVER REV, V65, P336, DOI 10.1016/j.addr.2012.07.001

Yu P, 2016, CELL PROLIFERAT, V49, P48, DOI 10.1111/cpr.12231

Zhang C, 2005, J NEUROTRAUM, V22, P1456, DOI 10.1089/neu.2005.22.1456

Zhang R, 2013, J NEUROINFLAMM, V10, DOI 10.1186/1742-2094-10-106

Zhang YL, 2017, NEUROCHEM INT, V111, P69, DOI 10.1016/j.neuint.2016.08.003

Zhang YL, 2015, J NEUROSURG, V122, P856, DOI 10.3171/2014.11.JNS14770

Zhang ZX, 2008, CYTOTHERAPY, V10, P134, DOI 10.1080/14653240701883061

NR 58

TC 36

Z9 37

U1 1

U2 12

PU FRONTIERS MEDIA SA

PI LAUSANNE

PA AVENUE DU TRIBUNAL FEDERAL 34, LAUSANNE, CH-1015, SWITZERLAND

EI 1662-5102

J9 FRONT CELL NEUROSCI

JI Front. Cell. Neurosci.

PD AUG 13

PY 2019

VL 13

AR 301

DI 10.3389/fncel.2019.00301

PG 9

WC Neurosciences

WE Science Citation Index Expanded (SCI-EXPANDED)

SC Neurosciences & Neurology

GA IQ2LE

UT WOS:000480579200001

PM 31456663

OA Green Published, gold

DA 2023-06-10

ER

PT J

AU Loe, ML

Indharty, RRS

Siahaan, AMP

Tandean, S

Riawan, W

AF Loe, Michael Lumintang

Indharty, R. R. Suzy

Siahaan, Andre M. P.

Tandean, Steven

Riawan, Wibi

TI The Effect of Intranasal Administration of ACTH Analogue Toward Neural

Progenitor/Stem Cells Proliferation after Traumatic Brain Injury

SO SAINS MALAYSIANA

LA English

DT Article

DE ACTH; BDNF; neural stem cells; SOX2; TrkB; SEM4X (R); traumatic brain

injury

ID NEUROTROPHIC FACTOR; STEM-CELLS; EXPRESSION; SEMAX; NEUROGENESIS;

HIPPOCAMPUS; NEURONS; BDNF; RECEPTOR

AB Traumatic brain injury (TBI) is a major health problem because of its high mortality and long-term disability worldwide. Neural progenitor/stem cells (NPSCs) that survive in certain parts of the brain, enable brain to produce new neurons and glia. ACTH(4-10)Pro(8)-Gly(9)-Pro(10) has a modulation effect on the expression and activation of the BDNF/TrkB system in the hippocampus area. The BDNF/TrkB pathway system is a potential therapeutic target toward NPSCs proliferation after TBI. Thirty male Sprague-Dawley rats were divided into three groups, i.e A=sham-operated controls; B=TBI; C=TBI+intranasal AACTH(4-10)Pro(8)-Gly(9)-Pro(10) achninistration. After 24 h, rats' brains were immunohistochemically processed, to observe the number of cells expressing mBDNF, TrkB, and SOX2 in the subgranular zone(SGZ) of the hippocampus dentate gyrus(DG). Data were analyzed with SPSS 17, ANOV4, Post Hoc Tukey HSD test, with p value < 0,05. Mean expression of BDNF group C= 16.33 +/- 2.83 increased significantly compared to group A=8.33 +/- 1.32(p=0.0001) and group B=5.89 +/- 1 .69(p=0.0001). Mean expression of TrkB group C=17.00 +/- 1.58 increased significantly compared to group A=4.33 +/- 1.73(p 0.0001) and group B=5.89 +/- 2.47(p=0.0001), TrkB expression in group B increased insignificantly compared to group A (p= 0.234). Mean expression of SOX2 in group C=12.56 +/- 2.07 increased significantly compared to group B = 8.89 +/- 2.318(p=0.0001) and group A=4.89 +/- 2.42(p=0.0001). ACTH(4-10)Pro(8)-Gly(9)-Pro(10) can increase the expression of BDNF and TrkB, and the proliferation of NPSCs in the subgranular zone (SGZ) of the hippocampus dentate gyrus (DG).

C1 [Loe, Michael Lumintang; Indharty, R. R. Suzy; Siahaan, Andre M. P.; Tandean, Steven] Univ Sumatera Utara, Fac Med, Dept Neurosurg, Haji Adam Malik Gen Hosp, Medan 20155, Indonesia.

[Riawan, Wibi] Univ Brawijaya, Fac Med, Dept Biochem, Malang 65145, Indonesia.

C3 University of North Sumatra; Brawijaya University

RP Loe, ML (通讯作者)，Univ Sumatera Utara, Fac Med, Dept Neurosurg, Haji Adam Malik Gen Hosp, Medan 20155, Indonesia.

EM dr.michael.lumintang@gmail.com

RI Tandean, Steven/AAZ-9237-2021; Tandean, Steven/HIR-9563-2022

OI Tandean, Steven/0000-0001-7924-032X; Siahaan, Andre Marolop

Pangihutan/0000-0003-1107-055X; Indharty, Suzy/0000-0001-7276-8746; Loe,

Michael Lumintang/0000-0003-4950-7104

CR Agapova TY, 2007, NEUROSCI LETT, V417, P201, DOI 10.1016/j.neulet.2007.02.042

Atwal JK, 2000, NEURON, V27, P265, DOI 10.1016/S0896-6273(00)00035-0

Cacialli P, 2018, NEURAL REGEN RES, V13, P941, DOI 10.4103/1673-5374.233430

Carney N., 2016, GUIDELINES MANAGEMEN, P244

Centers for Disease Control and Prevention, 2015, C TRAUM BRAIN INJ US

Conte V, 2008, RESTOR NEUROL NEUROS, V26, P45

Dewan MC, 2019, J NEUROSURG, V130, P1080, DOI 10.3171/2017.10.JNS17352

Dolotov OV, 2006, BRAIN RES, V1117, P54, DOI 10.1016/j.brainres.2006.07.108

Faigle R, 2013, BBA-GEN SUBJECTS, V1830, P2435, DOI 10.1016/j.bbagen.2012.09.002

Failla MD, 2016, NEUROREHAB NEURAL RE, V30, P83, DOI 10.1177/1545968315586465

Faried A, 2017, WORLD NEUROSURG, V100, P195, DOI 10.1016/j.wneu.2016.12.133

Gage FH, 2013, NEURON, V80, P588, DOI 10.1016/j.neuron.2013.10.037

Galgano M, 2017, CELL TRANSPLANT, V26, P1118, DOI 10.1177/0963689717714102

Gao X, 2009, J NEUROTRAUM, V26, P1325, DOI 10.1089/neu.2008-0744

Girgis F, 2016, FRONT SYST NEUROSCI, V10, DOI 10.3389/fnsys.2016.00008

Gupta VK, 2013, INT J MOL SCI, V14, P10122, DOI 10.3390/ijms140510122

Hicks RR, 1998, MOL BRAIN RES, V59, P264, DOI 10.1016/S0169-328X(98)00158-2

Jin K, 2003, MOL CELL NEUROSCI, V24, P171, DOI 10.1016/S1044-7431(03)00159-3

Kaplan GB, 2010, BEHAV PHARMACOL, V21, P427, DOI 10.1097/FBP.0b013e32833d8bc9

Koroleva SV, 2018, BIOL BULL+, V45, P589, DOI 10.1134/S1062359018060055

Lindvall O, 2015, CSH PERSPECT BIOL, V7, DOI 10.1101/cshperspect.a019034

Loe ML, 2019, BALI MED J, V8, P287, DOI 10.15562/bmj.v8i2.1454

Medvedeva EV, 2013, J MOL NEUROSCI, V49, P328, DOI 10.1007/s12031-012-9853-y

Numakawa T, 2010, HISTOL HISTOPATHOL, V25, P237, DOI 10.14670/HH-25.237

O'Dell DM, 2000, J NEUROSCI, V20, P4821

Poduslo JF, 1996, MOL BRAIN RES, V36, P280, DOI 10.1016/0169-328X(95)00250-V

Prins M, 2013, DIS MODEL MECH, V6, P1307, DOI 10.1242/dmm.011585

Rabinowitz AR, 2014, PSYCHIAT CLIN N AM, V37, P1, DOI 10.1016/j.psc.2013.11.004

Rolfe A, 2015, BRAIN NEUROTRAUMA MO

Sandhir R, 2008, EXP NEUROL, V213, P372, DOI 10.1016/j.expneurol.2008.06.013

Seo DE, 2019, AM J EMERG MED, V37, P1709, DOI 10.1016/j.ajem.2018.12.022

Sun D, 2014, NEURAL REGEN RES, V9, P688, DOI 10.4103/1673-5374.131567

Urrea C, 2007, RESTOR NEUROL NEUROS, V25, P65

Wolf JA, 2017, J NEUROTRAUM, V34, P2303, DOI 10.1089/neu.2016.4848

Zhang CL, 2008, NATURE, V451, P1004, DOI 10.1038/nature06562

Zhang YH, 2008, J PHYSIOL-LONDON, V586, P3113, DOI 10.1113/jphysiol.2008.152439

Zheng WM, 2013, J NEUROTRAUM, V30, P1872, DOI 10.1089/neu.2010.1579

Zuccato C, 2011, PLOS ONE, V6, DOI 10.1371/journal.pone.0022966

NR 38

TC 0

Z9 0

U1 0

U2 1

PU UNIV KEBANGSAAN MALAYSIA

PI SELANGOR

PA FACULTY SCIENCE & TECHNOLOGY, BANGI, SELANGOR, 43600, MALAYSIA

SN 0126-6039

J9 SAINS MALAYS

JI Sains Malays.

PD FEB

PY 2020

VL 49

IS 2

BP 375

EP 382

DI 10.17576/jsm-2020-4902-15

PG 8

WC Multidisciplinary Sciences

WE Science Citation Index Expanded (SCI-EXPANDED)

SC Science & Technology - Other Topics

GA LM6UE

UT WOS:000532384500015

OA Green Accepted, gold

DA 2023-06-10

ER

PT J

AU Badner, A

Cummings, BJ

AF Badner, Anna

Cummings, Brian J.

TI The endogenous progenitor response following traumatic brain injury: a

target for cell therapy paradigms

SO NEURAL REGENERATION RESEARCH

LA English

DT Review

DE cell therapy; endogenous repair; neurogenic niche; progenitors;

traumatic brain injury

ID NEURAL STEM-CELLS; OLIGODENDROCYTE PRECURSOR CELLS; ECTOPIC GRANULE

CELLS; SUBVENTRICULAR ZONE; HIPPOCAMPAL NEUROGENESIS; NEUROTROPHIC

FACTOR; ADULT MICE; CIRCUMVENTRICULAR ORGANS; STEM/PROGENITOR CELLS;

BEHAVIORAL RECOVERY

AB Although there is ample evidence that central nervous system progenitor pools respond to traumatic brain injury, the reported effects are variable and likely contribute to both recovery as well as pathophysiology. Through a better understanding of the diverse progenitor populations in the adult brain and their niche-specific reactions to traumatic insult, treatments can be tailored to enhance the benefits and dampen the deleterious effects of this response. This review provides an overview of endogenous precursors, the associated effects on cognitive recovery, and the potential of exogenous cell therapeutics to modulate these endogenous repair mechanisms. Beyond the hippocampal dentate gyrus and subventricular zone of the lateral ventricles, more recently identified sites of adult neurogenesis, the meninges, as well as circumventricular organs, are also discussed as targets for endogenous repair. Importantly, this review highlights that progenitor proliferation alone is no longer a meaningful outcome and studies must strive to better characterize precursor spatial localization, transcriptional profile, morphology, and functional synaptic integration. With improved insight and a more targeted approach, the stimulation of endogenous neurogenesis remains a promising strategy for recovery following traumatic brain injury.

C1 [Badner, Anna] Stanford Univ, Dept Psychiat & Behav Sci, Palo Alto, CA 94305 USA.

[Cummings, Brian J.] Univ Calif Irvine, Sue & Bill Gross Stem Cell Ctr, Irvine, CA USA.

[Cummings, Brian J.] Univ Calif Irvine, Inst Memory Impairments & Neurol Disorders, Irvine, CA USA.

[Cummings, Brian J.] Univ Calif Irvine, Phys Med & Rehabil, Irvine, CA USA.

[Cummings, Brian J.] Univ Calif Irvine, Dept Anat & Neurobiol, Irvine, CA 92717 USA.

C3 Stanford University; University of California System; University of

California Irvine; University of California System; University of

California Irvine; University of California System; University of

California Irvine; University of California System; University of

California Irvine

RP Badner, A (通讯作者)，Stanford Univ, Dept Psychiat & Behav Sci, Palo Alto, CA 94305 USA.

EM anna.badner@gmail.com

RI Badner, Anna/N-8035-2019

OI Badner, Anna/0000-0002-5244-6165

CR Ayoub R, 2020, NAT MED, V26, P1285, DOI 10.1038/s41591-020-0985-2

Badner A, 2021, J NEUROTRAUM, V38, P2731, DOI 10.1089/neu.2021.0045

Badner A, 2017, EXPERT OPIN BIOL TH, V17, P529, DOI 10.1080/14712598.2017.1308481

Baker SJ, 1996, ONCOGENE, V12, P1

Bao XJ, 2011, BRAIN RES, V1367, P103, DOI 10.1016/j.brainres.2010.10.063

Benner EJ, 2013, NATURE, V497, P369, DOI 10.1038/nature12069

Bennett L, 2009, MOL CELL NEUROSCI, V41, P337, DOI 10.1016/j.mcn.2009.04.007

Bennett LB, 2010, NEUROSCI LETT, V475, P1, DOI 10.1016/j.neulet.2010.03.019

Beretta S, 2017, CELL TRANSPLANT, V26, P1247, DOI 10.1177/0963689717714107

Bergles DE, 2000, NATURE, V405, P187, DOI 10.1038/35012083

Bifari F, 2017, CELL STEM CELL, V20, P360, DOI 10.1016/j.stem.2016.10.020

Blaiss CA, 2011, J NEUROSCI, V31, P4906, DOI 10.1523/JNEUROSCI.5265-10.2011

Blurton-Jones M, 2009, P NATL ACAD SCI USA, V106, P13594, DOI 10.1073/pnas.0901402106

Cacialli P, 2021, INT J MOL SCI, V22, DOI 10.3390/ijms22041585

Cameron MC, 2011, J COMP NEUROL, V519, P2175, DOI 10.1002/cne.22623

Carlson SW, 2014, J NEUROPATH EXP NEUR, V73, P734, DOI 10.1097/NEN.0000000000000092

Chang EH, 2016, FRONT NEUROSCI-SWITZ, V10, DOI 10.3389/fnins.2016.00332

Choi SH, 2009, MOL NEURODEGENER, V4, DOI 10.1186/1750-1326-4-52

Costine BA, 2015, DEV NEUROSCI-BASEL, V37, P115, DOI 10.1159/000369091

Dadwal P, 2015, STEM CELL REP, V5, P166, DOI 10.1016/j.stemcr.2015.06.011

Dash PK, 2001, J NEUROSCI RES, V63, P313, DOI 10.1002/1097-4547(20010215)63:4<313::AID-JNR1025>3.3.CO;2-W

Decimo I, 2021, NEUROSCIENTIST, V27, P506, DOI 10.1177/1073858420954826

Decimo I, 2011, STEM CELLS, V29, P2062, DOI 10.1002/stem.766

Derkach D, 2021, CELL REP MED, V2, DOI 10.1016/j.xcrm.2021.100231

DiBona VL, 2021, NEUROSCI RES, V172, P99, DOI 10.1016/j.neures.2021.05.007

Dimou L, 2008, J NEUROSCI, V28, P10434, DOI 10.1523/JNEUROSCI.2831-08.2008

Dixon KJ, 2015, J NEUROTRAUM, V32, P753, DOI 10.1089/neu.2014.3390

Dominici M, 2006, CYTOTHERAPY, V8, P315, DOI 10.1080/14653240600855905

Encinas JM, 2012, BEHAV BRAIN RES, V227, P433, DOI 10.1016/j.bbr.2011.10.010

Falnikar A, 2018, J NEUROTRAUM, V35, P2195, DOI 10.1089/neu.2017.5497

Frankland PW, 2013, TRENDS NEUROSCI, V36, P497, DOI 10.1016/j.tins.2013.05.002

Galvao RP, 2008, J NEUROSCI, V28, P13368, DOI 10.1523/JNEUROSCI.2918-08.2008

Gibson EM, 2014, SCIENCE, V344, P487, DOI 10.1126/science.1252304

Goings GE, 2004, BRAIN RES, V996, P213, DOI 10.1016/j.brainres.2003.10.034

Goodus MT, 2015, DEV NEUROSCI-BASEL, V37, P29, DOI 10.1159/000367784

Greer K, 2020, SCI REP-UK, V10, DOI 10.1038/s41598-020-72380-1

Hampton DW, 2004, NEUROSCIENCE, V127, P813, DOI 10.1016/j.neuroscience.2004.05.028

Haus DL, 2016, EXP NEUROL, V281, P1, DOI 10.1016/j.expneurol.2016.04.008

Henry RA, 2007, EUR J NEUROSCI, V25, P3513, DOI 10.1111/j.1460-9568.2007.05625.x

Houlton J, 2019, FRONT NEUROSCI-SWITZ, V13, DOI 10.3389/fnins.2019.00790

Hughes AN, 2019, NAT COMMUN, V10, DOI 10.1038/s41467-019-12059-y

Hughes EG, 2013, NAT NEUROSCI, V16, P668, DOI 10.1038/nn.3390

Ibrahim S, 2016, SCI REP-UK, V6, DOI 10.1038/srep21793

IP NY, 1993, NEURON, V10, P137, DOI 10.1016/0896-6273(93)90306-C

Jin KL, 2011, BRAIN RES, V1374, P56, DOI 10.1016/j.brainres.2010.12.037

Johanson C, 2011, J NEURAL TRANSM, V118, P115, DOI 10.1007/s00702-010-0498-0

Kan I, 2011, STEM CELL REV REP, V7, P404, DOI 10.1007/s12015-010-9190-x

Kase Y, 2020, INFLAMM REGEN, V40, DOI 10.1186/s41232-020-00122-x

Kumar M, 2014, ANN CLIN TRANSL NEUR, V1, P968, DOI 10.1002/acn3.137

Lee J, 2002, J NEUROCHEM, V82, P1367, DOI 10.1046/j.1471-4159.2002.01085.x

Lin RH, 2015, NEUROBIOL DIS, V74, P229, DOI 10.1016/j.nbd.2014.11.016

Littlejohn EL, 2021, FRONT CELL DEV BIOL, V9, DOI 10.3389/fcell.2021.663456

Littlejohn EL, 2020, ACTA NEUROPATHOL COM, V8, DOI 10.1186/s40478-020-00925-6

Liu XY, 2020, J NEUROCHEM, V153, P230, DOI 10.1111/jnc.14859

Lu DY, 2003, J NEUROSURG, V99, P351, DOI 10.3171/jns.2003.99.2.0351

Lu P, 2003, EXP NEUROL, V181, P115, DOI 10.1016/S0014-4886(03)00037-2

Maas AIR, 2017, LANCET NEUROL, V16, P987, DOI 10.1016/S1474-4422(17)30371-X

MacPhee IJ, 1997, J BIOL CHEM, V272, P23547, DOI 10.1074/jbc.272.38.23547

Mendez MF, 2017, J ALZHEIMERS DIS, V57, P667, DOI 10.3233/JAD-161002

Menon DK, 2010, ARCH PHYS MED REHAB, V91, P1637, DOI 10.1016/j.apmr.2010.05.017

Mine Y, 2013, NEUROBIOL DIS, V52, P191, DOI 10.1016/j.nbd.2012.12.006

Nakagomi Takayuki, 2017, Stem Cell Investig, V4, P22, DOI 10.21037/sci.2017.03.09

Nakagomi T, 2015, HISTOL HISTOPATHOL, V30, P391, DOI 10.14670/HH-30.391

Nakagomi T, 2012, STEM CELLS DEV, V21, P2350, DOI 10.1089/scd.2011.0657

Nakagomi T, 2011, STEM CELLS DEV, V20, P2037, DOI 10.1089/scd.2011.0279

Neuberger EJ, 2017, STEM CELL REP, V9, P972, DOI 10.1016/j.stemcr.2017.07.015

Ngwenya LB, 2019, FRONT NEUROSCI-SWITZ, V12, DOI 10.3389/fnins.2018.01014

Potts MB, 2012, CELL STEM CELL, V11, P5, DOI 10.1016/j.stem.2012.06.003

Radomski KL, 2013, BMC NEUROSCI, V14, DOI 10.1186/1471-2202-14-142

Robinson C, 2016, NEURAL PLAST, V2016, DOI 10.1155/2016/1347987

Rostami E, 2014, BRAIN RES, V1542, P195, DOI 10.1016/j.brainres.2013.10.047

Saha B, 2013, STEM CELL RES, V11, P965, DOI 10.1016/j.scr.2013.06.006

Sanin V, 2013, NEUROPATH APPL NEURO, V39, P510, DOI 10.1111/j.1365-2990.2012.01301.x

Scharfman H, 2005, EXP NEUROL, V192, P348, DOI 10.1016/j.expneurol.2004.11.016

Sritawan N, 2020, BIOMED PHARMACOTHER, V131, DOI 10.1016/j.biopha.2020.110651

Sundholm-Peters NL, 2005, J NEUROPATH EXP NEUR, V64, P1089, DOI 10.1097/01.jnen.0000190066.13312.8f

Tee AR, 2016, SEMIN CELL DEV BIOL, V52, P12, DOI 10.1016/j.semcdb.2016.01.040

Urrea C, 2007, RESTOR NEUROL NEUROS, V25, P65

van Gils A, 2020, PRACT NEUROL, V20, P213, DOI 10.1136/practneurol-2018-002087

Vilar M, 2016, FRONT NEUROSCI-SWITZ, V10, DOI 10.3389/fnins.2016.00026

von Streitberg A, 2021, FRONT CELL DEV BIOL, V9, DOI 10.3389/fcell.2021.662056

Wang J, 2012, CELL STEM CELL, V11, P23, DOI 10.1016/j.stem.2012.03.016

Wang XT, 2016, ENEURO, V3, DOI 10.1523/ENEURO.0162-16.2016

Wang XT, 2016, J NEUROTRAUM, V33, P721, DOI 10.1089/neu.2015.4097

Xie XHP, 2020, P NATL ACAD SCI USA, V117, P31448, DOI 10.1073/pnas.2014389117

Yoo SW, 2008, EXP MOL MED, V40, P387, DOI 10.3858/emm.2008.40.4.387

Yu TS, 2008, J NEUROSCI, V28, P12901, DOI 10.1523/JNEUROSCI.4629-08.2008

Zawadzka M, 2010, CELL STEM CELL, V6, P578, DOI 10.1016/j.stem.2010.04.002

Zheng WM, 2013, J NEUROTRAUM, V30, P1872, DOI 10.1089/neu.2010.1579

NR 89

TC 2

Z9 2

U1 3

U2 4

PU WOLTERS KLUWER MEDKNOW PUBLICATIONS

PI MUMBAI

PA WOLTERS KLUWER INDIA PVT LTD , A-202, 2ND FLR, QUBE, C T S NO 1498A-2

VILLAGE MAROL, ANDHERI EAST, MUMBAI, Maharashtra, INDIA

SN 1673-5374

EI 1876-7958

J9 NEURAL REGEN RES

JI Neural Regen. Res.

PD NOV

PY 2022

VL 17

IS 11

BP 2351

EP 2354

DI 10.4103/1673-5374.335833

PG 4

WC Cell Biology; Neurosciences

WE Science Citation Index Expanded (SCI-EXPANDED)

SC Cell Biology; Neurosciences & Neurology

GA 5F5JL

UT WOS:000866351500023

PM 35535870

OA Green Submitted, gold, Green Published

DA 2023-06-10

ER

PT J

AU Badner, A

Cummings, BJ

AF Badner, Anna

Cummings, Brian J.

TI The endogenous progenitor response following traumatic brain injury: a

target for cell therapy paradigms

SO NEURAL REGENERATION RESEARCH

LA English

DT Review

DE cell therapy; endogenous repair; neurogenic niche; progenitors;

traumatic brain injury

ID NEURAL STEM-CELLS; OLIGODENDROCYTE PRECURSOR CELLS; ECTOPIC GRANULE

CELLS; SUBVENTRICULAR ZONE; HIPPOCAMPAL NEUROGENESIS; NEUROTROPHIC

FACTOR; ADULT MICE; CIRCUMVENTRICULAR ORGANS; STEM/PROGENITOR CELLS;

BEHAVIORAL RECOVERY

AB Although there is ample evidence that central nervous system progenitor pools respond to traumatic brain injury, the reported effects are variable and likely contribute to both recovery as well as pathophysiology. Through a better understanding of the diverse progenitor populations in the adult brain and their niche-specific reactions to traumatic insult, treatments can be tailored to enhance the benefits and dampen the deleterious effects of this response. This review provides an overview of endogenous precursors, the associated effects on cognitive recovery, and the potential of exogenous cell therapeutics to modulate these endogenous repair mechanisms. Beyond the hippocampal dentate gyrus and subventricular zone of the lateral ventricles, more recently identified sites of adult neurogenesis, the meninges, as well as circumventricular organs, are also discussed as targets for endogenous repair. Importantly, this review highlights that progenitor proliferation alone is no longer a meaningful outcome and studies must strive to better characterize precursor spatial localization, transcriptional profile, morphology, and functional synaptic integration. With improved insight and a more targeted approach, the stimulation of endogenous neurogenesis remains a promising strategy for recovery following traumatic brain injury.

C1 [Badner, Anna] Stanford Univ, Dept Psychiat & Behav Sci, Palo Alto, CA 94305 USA.

[Cummings, Brian J.] Univ Calif Irvine, Sue & Bill Gross Stem Cell Ctr, Irvine, CA USA.

[Cummings, Brian J.] Univ Calif Irvine, Inst Memory Impairments & Neurol Disorders, Irvine, CA USA.

[Cummings, Brian J.] Univ Calif Irvine, Phys Med & Rehabil, Irvine, CA USA.

[Cummings, Brian J.] Univ Calif Irvine, Dept Anat & Neurobiol, Irvine, CA 92717 USA.

C3 Stanford University; University of California System; University of

California Irvine; University of California System; University of

California Irvine; University of California System; University of

California Irvine; University of California System; University of

California Irvine

RP Badner, A (通讯作者)，Stanford Univ, Dept Psychiat & Behav Sci, Palo Alto, CA 94305 USA.

EM anna.badner@gmail.com

CR Ayoub R, 2020, NAT MED, V26, P1285, DOI 10.1038/s41591-020-0985-2

Badner A, 2021, J NEUROTRAUM, V38, P2731, DOI 10.1089/neu.2021.0045

Badner A, 2017, EXPERT OPIN BIOL TH, V17, P529, DOI 10.1080/14712598.2017.1308481

Baker SJ, 1996, ONCOGENE, V12, P1

Bao XJ, 2011, BRAIN RES, V1367, P103, DOI 10.1016/j.brainres.2010.10.063

Benner EJ, 2013, NATURE, V497, P369, DOI 10.1038/nature12069

Bennett L, 2009, MOL CELL NEUROSCI, V41, P337, DOI 10.1016/j.mcn.2009.04.007

Bennett LB, 2010, NEUROSCI LETT, V475, P1, DOI 10.1016/j.neulet.2010.03.019

Beretta S, 2017, CELL TRANSPLANT, V26, P1247, DOI 10.1177/0963689717714107

Bergles DE, 2000, NATURE, V405, P187, DOI 10.1038/35012083

Bifari F, 2017, CELL STEM CELL, V20, P360, DOI 10.1016/j.stem.2016.10.020

Blaiss CA, 2011, J NEUROSCI, V31, P4906, DOI 10.1523/JNEUROSCI.5265-10.2011

Blurton-Jones M, 2009, P NATL ACAD SCI USA, V106, P13594, DOI 10.1073/pnas.0901402106

Cacialli P, 2021, INT J MOL SCI, V22, DOI 10.3390/ijms22041585

Cameron MC, 2011, J COMP NEUROL, V519, P2175, DOI 10.1002/cne.22623

Carlson SW, 2014, J NEUROPATH EXP NEUR, V73, P734, DOI 10.1097/NEN.0000000000000092

Chang EH, 2016, FRONT NEUROSCI-SWITZ, V10, DOI 10.3389/fnins.2016.00332

Choi SH, 2009, MOL NEURODEGENER, V4, DOI 10.1186/1750-1326-4-52

Costine BA, 2015, DEV NEUROSCI-BASEL, V37, P115, DOI 10.1159/000369091

Dadwal P, 2015, STEM CELL REP, V5, P166, DOI 10.1016/j.stemcr.2015.06.011

Dash PK, 2001, J NEUROSCI RES, V63, P313, DOI 10.1002/1097-4547(20010215)63:4<313::AID-JNR1025>3.3.CO;2-W

Decimo I, 2021, NEUROSCIENTIST, V27, P506, DOI 10.1177/1073858420954826

Decimo I, 2011, STEM CELLS, V29, P2062, DOI 10.1002/stem.766

Derkach D, 2021, CELL REP MED, V2, DOI 10.1016/j.xcrm.2021.100231

DiBona VL, 2021, NEUROSCI RES, V172, P99, DOI 10.1016/j.neures.2021.05.007

Dimou L, 2008, J NEUROSCI, V28, P10434, DOI 10.1523/JNEUROSCI.2831-08.2008

Dixon KJ, 2015, J NEUROTRAUM, V32, P753, DOI 10.1089/neu.2014.3390

Dominici M, 2006, CYTOTHERAPY, V8, P315, DOI 10.1080/14653240600855905

Encinas JM, 2012, BEHAV BRAIN RES, V227, P433, DOI 10.1016/j.bbr.2011.10.010

Falnikar A, 2018, J NEUROTRAUM, V35, P2195, DOI 10.1089/neu.2017.5497

Frankland PW, 2013, TRENDS NEUROSCI, V36, P497, DOI 10.1016/j.tins.2013.05.002

Galvao RP, 2008, J NEUROSCI, V28, P13368, DOI 10.1523/JNEUROSCI.2918-08.2008

Gibson EM, 2014, SCIENCE, V344, P487, DOI 10.1126/science.1252304

Goings GE, 2004, BRAIN RES, V996, P213, DOI 10.1016/j.brainres.2003.10.034

Goodus MT, 2015, DEV NEUROSCI-BASEL, V37, P29, DOI 10.1159/000367784

Greer K, 2020, SCI REP-UK, V10, DOI 10.1038/s41598-020-72380-1

Hampton DW, 2004, NEUROSCIENCE, V127, P813, DOI 10.1016/j.neuroscience.2004.05.028

Haus DL, 2016, EXP NEUROL, V281, P1, DOI 10.1016/j.expneurol.2016.04.008

Henry RA, 2007, EUR J NEUROSCI, V25, P3513, DOI 10.1111/j.1460-9568.2007.05625.x

Houlton J, 2019, FRONT NEUROSCI-SWITZ, V13, DOI 10.3389/fnins.2019.00790

Hughes AN, 2019, NAT COMMUN, V10, DOI 10.1038/s41467-019-12059-y

Hughes EG, 2013, NAT NEUROSCI, V16, P668, DOI 10.1038/nn.3390

Ibrahim S, 2016, SCI REP-UK, V6, DOI 10.1038/srep21793

IP NY, 1993, NEURON, V10, P137, DOI 10.1016/0896-6273(93)90306-C

Jin KL, 2011, BRAIN RES, V1374, P56, DOI 10.1016/j.brainres.2010.12.037

Johanson C, 2011, J NEURAL TRANSM, V118, P115, DOI 10.1007/s00702-010-0498-0

Kan I, 2011, STEM CELL REV REP, V7, P404, DOI 10.1007/s12015-010-9190-x

Kase Y, 2020, INFLAMM REGEN, V40, DOI 10.1186/s41232-020-00122-x

Kumar M, 2014, ANN CLIN TRANSL NEUR, V1, P968, DOI 10.1002/acn3.137

Lee J, 2002, J NEUROCHEM, V82, P1367, DOI 10.1046/j.1471-4159.2002.01085.x

Lin RH, 2015, NEUROBIOL DIS, V74, P229, DOI 10.1016/j.nbd.2014.11.016

Littlejohn EL, 2021, FRONT CELL DEV BIOL, V9, DOI 10.3389/fcell.2021.663456

Littlejohn EL, 2020, ACTA NEUROPATHOL COM, V8, DOI 10.1186/s40478-020-00925-6

Liu XY, 2020, J NEUROCHEM, V153, P230, DOI 10.1111/jnc.14859

Lu DY, 2003, J NEUROSURG, V99, P351, DOI 10.3171/jns.2003.99.2.0351

Lu P, 2003, EXP NEUROL, V181, P115, DOI 10.1016/S0014-4886(03)00037-2

Maas AIR, 2017, LANCET NEUROL, V16, P987, DOI 10.1016/S1474-4422(17)30371-X

MacPhee IJ, 1997, J BIOL CHEM, V272, P23547, DOI 10.1074/jbc.272.38.23547

Mendez MF, 2017, J ALZHEIMERS DIS, V57, P667, DOI 10.3233/JAD-161002

Menon DK, 2010, ARCH PHYS MED REHAB, V91, P1637, DOI 10.1016/j.apmr.2010.05.017

Mine Y, 2013, NEUROBIOL DIS, V52, P191, DOI 10.1016/j.nbd.2012.12.006

Nakagomi Takayuki, 2017, Stem Cell Investig, V4, P22, DOI 10.21037/sci.2017.03.09

Nakagomi T, 2015, HISTOL HISTOPATHOL, V30, P391, DOI 10.14670/HH-30.391

Nakagomi T, 2012, STEM CELLS DEV, V21, P2350, DOI 10.1089/scd.2011.0657

Nakagomi T, 2011, STEM CELLS DEV, V20, P2037, DOI 10.1089/scd.2011.0279

Neuberger EJ, 2017, STEM CELL REP, V9, P972, DOI 10.1016/j.stemcr.2017.07.015

Ngwenya LB, 2019, FRONT NEUROSCI-SWITZ, V12, DOI 10.3389/fnins.2018.01014

Potts MB, 2012, CELL STEM CELL, V11, P5, DOI 10.1016/j.stem.2012.06.003

Radomski KL, 2013, BMC NEUROSCI, V14, DOI 10.1186/1471-2202-14-142

Robinson C, 2016, NEURAL PLAST, V2016, DOI 10.1155/2016/1347987

Rostami E, 2014, BRAIN RES, V1542, P195, DOI 10.1016/j.brainres.2013.10.047

Saha B, 2013, STEM CELL RES, V11, P965, DOI 10.1016/j.scr.2013.06.006

Sanin V, 2013, NEUROPATH APPL NEURO, V39, P510, DOI 10.1111/j.1365-2990.2012.01301.x

Scharfman H, 2005, EXP NEUROL, V192, P348, DOI 10.1016/j.expneurol.2004.11.016

Sritawan N, 2020, BIOMED PHARMACOTHER, V131, DOI 10.1016/j.biopha.2020.110651

Sundholm-Peters NL, 2005, J NEUROPATH EXP NEUR, V64, P1089, DOI 10.1097/01.jnen.0000190066.13312.8f

Tee AR, 2016, SEMIN CELL DEV BIOL, V52, P12, DOI 10.1016/j.semcdb.2016.01.040

Urrea C, 2007, RESTOR NEUROL NEUROS, V25, P65

van Gils A, 2020, PRACT NEUROL, V20, P213, DOI 10.1136/practneurol-2018-002087

Vilar M, 2016, FRONT NEUROSCI-SWITZ, V10, DOI 10.3389/fnins.2016.00026

von Streitberg A, 2021, FRONT CELL DEV BIOL, V9, DOI 10.3389/fcell.2021.662056

Wang J, 2012, CELL STEM CELL, V11, P23, DOI 10.1016/j.stem.2012.03.016

Wang XT, 2016, ENEURO, V3, DOI 10.1523/ENEURO.0162-16.2016

Wang XT, 2016, J NEUROTRAUM, V33, P721, DOI 10.1089/neu.2015.4097

Xie XHP, 2020, P NATL ACAD SCI USA, V117, P31448, DOI 10.1073/pnas.2014389117

Yoo SW, 2008, EXP MOL MED, V40, P387, DOI 10.3858/emm.2008.40.4.387

Yu TS, 2008, J NEUROSCI, V28, P12901, DOI 10.1523/JNEUROSCI.4629-08.2008

Zawadzka M, 2010, CELL STEM CELL, V6, P578, DOI 10.1016/j.stem.2010.04.002

Zheng WM, 2013, J NEUROTRAUM, V30, P1872, DOI 10.1089/neu.2010.1579

NR 89

TC 0

Z9 0

U1 2

U2 2

PU WOLTERS KLUWER MEDKNOW PUBLICATIONS

PI MUMBAI

PA WOLTERS KLUWER INDIA PVT LTD , A-202, 2ND FLR, QUBE, C T S NO 1498A-2

VILLAGE MAROL, ANDHERI EAST, MUMBAI, Maharashtra, INDIA

SN 1673-5374

EI 1876-7958

J9 NEURAL REGEN RES

JI Neural Regen. Res.

PD NOV

PY 2022

VL 17

IS 11

PG 4

WC Cell Biology; Neurosciences

WE Science Citation Index Expanded (SCI-EXPANDED)

SC Cell Biology; Neurosciences & Neurology

GA 5F5JL

UT WOS:000866351500013

DA 2023-06-10

ER

PT J

AU Longhi, L

Zanier, ER

Royo, N

Stocchetti, N

McIntosh, TK

AF Longhi, L

Zanier, ER

Royo, N

Stocchetti, N

McIntosh, TK

TI Stem cell transplantation as a therapeutic strategy for traumatic brain

injury

SO TRANSPLANT IMMUNOLOGY

LA English

DT Article

DE traumatic brain injury; neuroprotection; transplantation; stem cell

ID MARROW STROMAL CELLS; BONE-MARROW; PROGENITOR CELLS; COGNITIVE FUNCTION;

NEURONAL CELLS; RAT MODEL; SURVIVAL; MIGRATION; RECOVERY;

DIFFERENTIATION

AB Stem cell transplantation has enormous potential to be a viable therapeutic approach to replace the lost tissue/cells following traumatic brain injury (TBI). Several types of cell lines such as immortalized progenitors cells, embryonic rodent and human stem cells and bone marrow-derived cells have been successfully transplanted in experimental models of TBI, resulting in reduced neurobehavioral deficits and attenuation of histological damage. To date, it remains unclear whether stem cell are effective following transplantation into the injured brain via either cell replacement, trophic support, or manipulation of the local environment to stimulate endogenous neuroprotection/regeneration. This paper will review the most current and exciting pre-clinical data regarding the utility of cellular transplantation in experimental models of TBI. We believe that further work must continue to better understand the interaction between the host and the transplanted cells as well as the mechanisms regulating their differentiation into mature and functionally active neurons/glia. (C) 2005 Elsevier B.V. All rights reserved.

C1 Univ Milan, Osped Maggiore Policlin, Fdn IRCCS, Dept Anesthesia & Crit Care Med,Neurosurg Intens, I-20100 Milan, Italy.

Univ Penn, Dept Neurosurg, Head Injury Lab, Philadelphia, PA 19104 USA.

C3 IRCCS Ca Granda Ospedale Maggiore Policlinico; University of Milan;

University of Pennsylvania

RP Longhi, L (通讯作者)，Univ Milan, Osped Maggiore Policlin, Fdn IRCCS, Dept Anesthesia & Crit Care Med,Neurosurg Intens, Via Sforza 35, I-20100 Milan, Italy.

EM lucalonghi@policlinico.mi.it

RI Stocchetti, Nino/O-7444-2017; Longhi, Luca/AAF-9903-2021; Zanier,

Elisa/AAA-8095-2020

OI Stocchetti, Nino/0000-0003-3250-6834; Longhi, Luca/0000-0001-9894-8788;

Zanier, Elisa/0000-0002-3011-8718

CR Boockvar JA, 2005, NEUROSURGERY, V56, P163, DOI 10.1227/01.NEU.0000145866.25433.FF

Dezawa M, 2004, J CLIN INVEST, V113, P1701, DOI 10.1172/JCI200420935

EMSLEY JG, 2005, PROG NEUROBIOL

Englund U, 2002, DEV BRAIN RES, V134, P123, DOI 10.1016/S0165-3806(01)00330-3

Gage FH, 2000, SCIENCE, V287, P1433, DOI 10.1126/science.287.5457.1433

Hagan M, 2003, NEUROSCI LETT, V351, P149, DOI 10.1016/j.neulet.2003.07.021

Hoane MR, 2004, J NEUROTRAUM, V21, P163, DOI 10.1089/089771504322778622

Jennett B., 1997, HEAD INJURY, P439

Keirstead HS, 2005, J NEUROSCI, V25, P4694, DOI 10.1523/JNEUROSCI.0311-05.2005

LE J, 2002, BIODRUGS, V16, P389

Lenzlinger PM, 2002, EXP NEUROL, V175, P421

Lindvall O, 2004, STROKE, V35, P2691, DOI 10.1161/01.STR.0000143323.84008.f4

Lindvall O, 2004, NAT MED, V10, pS42, DOI 10.1038/nm1064

Longhi L, 2004, J NEUROTRAUM, V21, P1723, DOI 10.1089/0897715042664876

Lu DY, 2001, J NEUROTRAUM, V18, P813, DOI 10.1089/089771501316919175

Lundberg C, 1996, BRAIN RES, V737, P295, DOI 10.1016/0006-8993(96)00923-7

Mahmood A, 2004, NEUROSURGERY, V55, P1185, DOI 10.1227/01.NEU.0000141042.14476.3C

Mahmood A, 2004, J NEUROTRAUM, V21, P33, DOI 10.1089/089771504772695922

Mahmood A, 2001, NEUROSURGERY, V49, P1196, DOI 10.1097/00006123-200111000-00031

Mahmood A, 2001, J NEUROSURG, V94, P589, DOI 10.3171/jns.2001.94.4.0589

MartinezSerrano A, 1996, J NEUROSCI, V16, P4604

McIntosh TK, 1998, J NEUROTRAUM, V15, P731, DOI 10.1089/neu.1998.15.731

McIntosh TK, 1998, NEUROPATH APPL NEURO, V24, P251

McKay R, 1997, SCIENCE, V276, P66, DOI 10.1126/science.276.5309.66

Philips MF, 1999, J NEUROSURG, V90, P116, DOI 10.3171/jns.1999.90.1.0116

Philips MF, 2001, J NEUROSURG, V94, P765, DOI 10.3171/jns.2001.94.5.0765

Ragnarsson KT, 1999, JAMA-J AM MED ASSOC, V282, P974

RENFRANZ PJ, 1991, CELL, V66, P713, DOI 10.1016/0092-8674(91)90116-G

Riess P, 2002, NEUROSURGERY, V51, P1043, DOI 10.1097/00006123-200210000-00035

Royo NC, 2003, CURR OPIN PHARMACOL, V3, P27, DOI 10.1016/S1471-4892(02)00006-1

RYDER EF, 1990, J NEUROBIOL, V21, P356, DOI 10.1002/neu.480210209

Sanchez-Ramos JR, 2002, J NEUROSCI RES, V69, P880, DOI 10.1002/jnr.10337

Schouten JW, 2004, J NEUROTRAUM, V21, P1501, DOI 10.1089/0897715042441774

Seaberg RM, 2003, TRENDS NEUROSCI, V26, P125, DOI 10.1016/S0166-2236(03)00031-6

Shear DA, 2004, BRAIN RES, V1026, P11, DOI 10.1016/j.brainres.2004.07.087

Sinden JD, 1997, NEUROSCIENCE, V81, P599, DOI 10.1016/S0306-4522(97)00330-8

Sinson G, 1996, J NEUROSURG, V84, P655, DOI 10.3171/jns.1996.84.4.0655

Smith DH, 1997, J NEUROTRAUM, V14, P715, DOI 10.1089/neu.1997.14.715

SOARES H, 1991, Journal of Neural Transplantation and Plasticity, V2, P207

Sortwell CE, 2003, FRONT BIOSCI, V8, pS522, DOI 10.2741/1096

Svendsen CN, 2004, NAT MED, V10, P224, DOI 10.1038/nm0304-224

Tate MC, 2002, CELL TRANSPLANT, V11, P283

The Brain Trauma Foundation, 2000, J NEUROTRAUM, V17, P457

Thomson JA, 1998, SCIENCE, V282, P1145, DOI 10.1126/science.282.5391.1145

Vescovi AL, 1999, J NEUROTRAUM, V16, P689, DOI 10.1089/neu.1999.16.689

Watson DJ, 2003, J NEUROPATH EXP NEUR, V62, P368, DOI 10.1093/jnen/62.4.368

Wennersten A, 2004, J NEUROSURG, V100, P88, DOI 10.3171/jns.2004.100.1.0088

ZHANG C, 2005, J NEUROTRAUMA

NR 48

TC 45

Z9 52

U1 0

U2 5

PU ELSEVIER SCIENCE BV

PI AMSTERDAM

PA PO BOX 211, 1000 AE AMSTERDAM, NETHERLANDS

SN 0966-3274

EI 2210-3384

J9 TRANSPL IMMUNOL

JI Transpl. Immunol.

PD DEC

PY 2005

VL 15

IS 2

BP 143

EP 148

DI 10.1016/j.trim.2005.09.003

PG 6

WC Immunology; Transplantation

WE Science Citation Index Expanded (SCI-EXPANDED)

SC Immunology; Transplantation

GA 007GM

UT WOS:000234956800007

PM 16412958

DA 2023-06-10

ER

PT J

AU Hong, S

Washington, PM

Kim, A

Yang, CP

Yu, TS

Kernie, SG

AF Hong, Sue

Washington, Patricia M.

Kim, Ahleum

Yang, Cui-Ping

Yu, Tzong-Shiue

Kernie, Steven G.

TI Apolipoprotein E Regulates Injury-Induced Activation of Hippocampal

Neural Stem and Progenitor Cells

SO JOURNAL OF NEUROTRAUMA

LA English

DT Article

DE apolipoprotein E; hippocampus; neural stem; progenitor cells;

neurogenesis; traumatic brain injury

ID TRAUMATIC BRAIN-INJURY; FIBRILLARY ACIDIC PROTEIN; AMYLOID-BETA-PEPTIDE;

ALZHEIMERS-DISEASE; DENTATE GYRUS; EXPRESSING PROGENITORS;

STEM/PROGENITOR CELLS; CEREBRAL-ISCHEMIA; ADULT HIPPOCAMPUS; TRANSGENIC

MICE

AB Partial recovery from even severe traumatic brain injury (TBI) is ubiquitous and occurs largely through unknown mechanisms. Recent evidence suggests that hippocampal neural stem/progenitor cell (NSPC) activation and subsequent neurogenesis are responsible for at least some aspects of spontaneous recovery following TBI. Apolipoprotein E (ApoE) regulates postnatal neurogenesis in the hippocampus and is therefore a putative mediator of injury-induced neurogenesis. Further, ApoE isoforms in humans are associated with different cognitive outcomes following TBI. To investigate the role of ApoE in injury-induced neurogenesis, we exposed wild-type, ApoE-deficient, and human ApoE isoform-specific (ApoE3 and ApoE4) transgenic mice crossed with nestin-green fluorescent protein (GFP) reporter mice to controlled cortical impact (CCI) and assessed progenitor activation at 2 d post-injury using unbiased stereology. GFP+ progenitor cells were increased by approximately 120% in the ipsilateral hippocampus in injured wild-type mice, compared with sham mice (p<0.01). Co-localization of GFP+ cells with bromodeoxyrudine (BrdU) to label dividing cells indicated increased proliferation of progenitors in the injured hippocampus (p<0.001). This proliferative injury response was absent in ApoE-deficient mice, as no increase in GFP+ cells was observed in the injured hippocampus, compared with sham mice, despite an overall increase in proliferation indicated by increased BrdU+ cells (86%; p<0.05). CCI-induced proliferation of GFP+ cells in both ApoE3 and ApoE4 mice but the overall response was attenuated in ApoE4 mice due to fewer GFP+ cells at baseline. We demonstrate that ApoE is required for injury-induced proliferation of NSPCs after experimental TBI, and that this response is influenced by human APOE genotype.

C1 [Hong, Sue; Washington, Patricia M.; Kim, Ahleum; Yu, Tzong-Shiue; Kernie, Steven G.] Columbia Univ Coll Phys & Surg, Dept Pediat, New York, NY 10032 USA.

[Hong, Sue; Washington, Patricia M.; Kim, Ahleum; Yu, Tzong-Shiue; Kernie, Steven G.] Columbia Univ Coll Phys & Surg, Dept Pathol & Cell Biol, New York, NY 10032 USA.

[Yang, Cui-Ping] Chinese Acad Sci, Key Lab Anim Models & Human Dis Mech, Kunming, Yunnan, Peoples R China.

C3 Columbia University; Columbia University; Chinese Academy of Sciences

RP Kernie, SG (通讯作者)，Columbia Univ Coll Phys & Surg, 3959 Broadway,CHN 10-24, New York, NY 10032 USA.

EM sk3516@columbia.edu

RI Kernie, Steven/AAT-9912-2020; Kernie, Steven/HZM-4287-2023

OI Yu, Tzong-Shiue/0000-0003-4277-6213; Kernie, Steven/0000-0003-1371-0549

FU NINDS NIH HHS [R01 NS095803, R56 NS089523, R21 NS083077] Funding Source:

Medline

CR Bartkowska K, 2007, DEVELOPMENT, V134, P4369, DOI 10.1242/dev.008227

Bell RD, 2007, J CEREBR BLOOD F MET, V27, P909, DOI 10.1038/sj.jcbfm.9600419

Bell RD, 2012, NATURE, V485, P512, DOI 10.1038/nature11087

Blaiss CA, 2011, J NEUROSCI, V31, P4906, DOI 10.1523/JNEUROSCI.5265-10.2011

CORDER EH, 1993, SCIENCE, V261, P921, DOI 10.1126/science.8346443

Coronado Victor G., 2011, Morbidity and Mortality Weekly Report, V60, P1

Crawford F, 2009, NEUROSCIENCE, V159, P1349, DOI 10.1016/j.neuroscience.2009.01.033

Dash PK, 2001, J NEUROSCI RES, V63, P313, DOI 10.1002/1097-4547(20010215)63:4<313::AID-JNR1025>3.3.CO;2-W

Dean DC, 2014, JAMA NEUROL, V71, P11, DOI 10.1001/jamaneurol.2013.4544

Deane R, 2008, J CLIN INVEST, V118, P4002, DOI 10.1172/JCI36663

Denise A, 2004, NAT NEUROSCI, V7, P1233, DOI 10.1038/nn1340

Doetsch F, 1996, P NATL ACAD SCI USA, V93, P14895, DOI 10.1073/pnas.93.25.14895

Eng LF, 2000, NEUROCHEM RES, V25, P1439, DOI 10.1023/A:1007677003387

Eriksson PS, 1998, NAT MED, V4, P1313, DOI 10.1038/3305

Fukuda S, 2003, J NEUROSCI, V23, P9357

Gilley JA, 2011, HIPPOCAMPUS, V21, P33, DOI 10.1002/hipo.20719

Gu F, 2005, J NEUROSCI RES, V81, P163, DOI 10.1002/jnr.20561

Halliday MR, 2013, JAMA NEUROL, V70, P1198, DOI 10.1001/jamaneurol.2013.3841

Hartman RE, 2001, EXP NEUROL, V170, P326, DOI 10.1006/exnr.2001.7715

Hauser PS, 2011, PROG LIPID RES, V50, P62, DOI 10.1016/j.plipres.2010.09.001

Hong YT, 2014, JAMA NEUROL, V71, P23, DOI 10.1001/jamaneurol.2013.4847

James ML, 2009, J STROKE CEREBROVASC, V18, P144, DOI 10.1016/j.jstrokecerebrovasdis.2008.09.012

Jiang Q, 2008, NEURON, V58, P681, DOI 10.1016/j.neuron.2008.04.010

Jin KL, 2001, P NATL ACAD SCI USA, V98, P4710, DOI 10.1073/pnas.081011098

Kalani MYS, 2008, P NATL ACAD SCI USA, V105, P16970, DOI 10.1073/pnas.0808616105

Kasai M, 2005, GENES CELLS, V10, P777, DOI 10.1111/j.1365-2443.2005.00876.x

Kernie SG, 2001, J NEUROSCI RES, V66, P317, DOI 10.1002/jnr.10013

Kernie SG, 2010, NEUROBIOL DIS, V37, P267, DOI 10.1016/j.nbd.2009.11.002

Kronenberg G, 2003, J COMP NEUROL, V467, P455, DOI 10.1002/cne.10945

Laplagne DA, 2006, PLOS BIOL, V4, P2349, DOI 10.1371/journal.pbio.0040409

Li G, 2009, CELL STEM CELL, V5, P634, DOI 10.1016/j.stem.2009.10.015

Loane DJ, 2011, J NEUROTRAUM, V28, P225, DOI 10.1089/neu.2010.1595

Loane DJ, 2009, NAT MED, V15, P377, DOI 10.1038/nm.1940

Mannix RC, 2011, J CEREBR BLOOD F MET, V31, P351, DOI 10.1038/jcbfm.2010.99

Mauch DH, 2001, SCIENCE, V294, P1354, DOI 10.1126/science.294.5545.1354

Mauri M, 2006, FUNCT NEUROL, V21, P223

Methia N, 2001, MOL MED, V7, P810, DOI 10.1007/BF03401973

Miles DK, 2008, HIPPOCAMPUS, V18, P793, DOI 10.1002/hipo.20439

Nishitsuji K, 2011, J BIOL CHEM, V286, P17536, DOI 10.1074/jbc.M111.225532

Otaegi G, 2006, J CELL SCI, V119, P2739, DOI 10.1242/jcs.03012

Parent JM, 1997, J NEUROSCI, V17, P3727

Ponsford J, 2011, J NEUROTRAUM, V28, P1683, DOI 10.1089/neu.2010.1623

Qi JP, 2007, J ALZHEIMERS DIS, V12, P335

Quesseveur G, 2013, TRANSL PSYCHIAT, V3, DOI 10.1038/tp.2013.30

Rajat S., 2008, EXP NEUROL, V213, P372

Ridet JL, 1997, TRENDS NEUROSCI, V20, P570, DOI 10.1016/S0166-2236(97)01139-9

ROBERTS GW, 1991, LANCET, V338, P1422, DOI 10.1016/0140-6736(91)92724-G

Shaw P, 2007, LANCET NEUROL, V6, P494, DOI 10.1016/S1474-4422(07)70106-0

Shetty AK, 2005, GLIA, V51, P173, DOI 10.1002/glia.20187

Smith DH, 2003, J NEUROSURG, V98, P1072, DOI 10.3171/jns.2003.98.5.1072

STERN Y, 1995, NEUROLOGY, V45, P55, DOI 10.1212/WNL.45.1.55

Sun D., 2014, J NEUROTRAU IN PRESS

Sun YL, 1998, J NEUROSCI, V18, P3261

van Praag H, 2002, NATURE, V415, P1030, DOI 10.1038/4151030a

Vance JE, 2000, BBA-MOL CELL BIOL L, V1486, P84, DOI 10.1016/S1388-1981(00)00050-0

Washington PM, 2014, J NEUROTRAUM, V31, P125, DOI 10.1089/neu.2013.3017

Xu Q, 2006, J NEUROSCI, V26, P4985, DOI 10.1523/JNEUROSCI.5476-05.2006

Yang CP, 2011, DEVELOPMENT, V138, P4351, DOI 10.1242/dev.065540

Yu T. S., 2014, NEUROSCIENTIST

Yu TS, 2008, J NEUROSCI, V28, P12901, DOI 10.1523/JNEUROSCI.4629-08.2008

Zhao CM, 2008, CELL, V132, P645, DOI 10.1016/j.cell.2008.01.033

Zhou WD, 2008, J NEUROTRAUM, V25, P279, DOI 10.1089/neu.2007.0489

Zlokovic BV, 2011, NAT REV NEUROSCI, V12, P723, DOI 10.1038/nrn3114

NR 63

TC 17

Z9 17

U1 1

U2 3

PU MARY ANN LIEBERT, INC

PI NEW ROCHELLE

PA 140 HUGUENOT STREET, 3RD FL, NEW ROCHELLE, NY 10801 USA

SN 0897-7151

EI 1557-9042

J9 J NEUROTRAUM

JI J. Neurotrauma

PD FEB 15

PY 2016

VL 33

IS 4

BP 362

EP 374

DI 10.1089/neu.2014.3860

PG 13

WC Critical Care Medicine; Clinical Neurology; Neurosciences

WE Science Citation Index Expanded (SCI-EXPANDED)

SC General & Internal Medicine; Neurosciences & Neurology

GA DD2NH

UT WOS:000369758800006

PM 25905575

OA Green Published

DA 2023-06-10

ER

PT J

AU Richardson, RM

Singh, A

Sun, D

Fillmore, HL

Dietrich, DW

Bullock, MR

AF Richardson, R. Mark

Singh, Amanpreet

Sun, Dong

Fillmore, Helen L.

Dietrich, Dalton W., III

Bullock, M. Ross

TI Stem cell biology in traumatic brain injury: effects of injury and

strategies for repair

SO JOURNAL OF NEUROSURGERY

LA English

DT Review

DE traumatic brain injury; neurogenesis; neural progenitors; cell

transplantation

ID CENTRAL-NERVOUS-SYSTEM; SPINAL-CORD-INJURY; ADULT HUMAN BRAIN;

CONTROLLED CORTICAL IMPACT; NEURAL PROGENITOR CELLS; OLFACTORY

ENSHEATHING CELLS; NEUROTROPHIC PROTEIN S100B; SUBCORTICAL WHITE-MATTER;

HUMAN NEURONAL CELLS; HUMAN HEAD-INJURY

AB Approximately 350,000 individuals in the US are affected annually by severe and moderate traumatic brain injuries (TBI) that may result in long-term disability. This rate of injury has produced similar to 3.3 million disabled survivors in the US alone. There is currently no specific treatment available for TBI other than supportive care, but aggressive prehospital resuscitation, rapid triage, and intensive care have reduced mortality rates. With the recent demonstration that neurogenesis occurs in all mammals (including man) throughout adult life, albeit at a low rate, the concept of replacing neurons lost after TBI is now becoming a reality. Experimental rodent models have shown that neurogenesis is accelerated after TBI, especially in juveniles. Two approaches have been followed in these rodent models to test possible therapeutic approaches that could enhance neuronal replacement in humans after TBI. The first has been to define and quantify the phenomenon of de novo hippocampal and cortical neurogenesis after TBI and find ways to enhance this (for example by exogenous trophic factor administration). A second approach has been the transplantation of different types of neural progenitor cells after TBI. In this review the authors discuss some of the processes that follow after acute TBI including the changes in the brain microenvironment and the role of trophic factor dynamics with regard to the effects on endogenous neurogenesis and gliagenesis. The authors also discuss strategies to clinically harness the factors influencing these processes and repair strategies using exogenous neural progenitor cell transplantation. Each strategy is discussed with an emphasis on highlighting the progress and limiting factors relevant to the development of clinical trials of cellular replacement therapy for severe TBI in humans. (DOI: 10.3171/2009.4.JNS081087)

C1 [Bullock, M. Ross] Univ Miami, Miller Sch Med, Lois Pope LIFE Ctr, Dept Neurosurg,Miami Project Cure Paralysis, Miami, FL 33136 USA.

[Dietrich, Dalton W., III; Bullock, M. Ross] Univ Miami, Miller Sch Med, Dept Neurol Surg, Miami, FL 33136 USA.

[Richardson, R. Mark] Univ Calif San Francisco, Dept Neurol Surg, San Francisco, CA 94143 USA.

[Sun, Dong; Fillmore, Helen L.] Virginia Commonwealth Univ, Coll Med, Virginia Hosp, Dept Neurosurg, Richmond, VA USA.

C3 University of Miami; University of Miami; University of California

System; University of California San Francisco; Virginia Commonwealth

University

RP Bullock, MR (通讯作者)，Univ Miami, Miller Sch Med, Lois Pope LIFE Ctr, Dept Neurosurg,Miami Project Cure Paralysis, Room 3-20,1095 NW 14th Terrace, Miami, FL 33136 USA.

EM rbullock@med.miami.edu

RI Richardson, R Mark/C-6819-2016

OI Richardson, R Mark/0000-0003-2620-7387; Fillmore,

Helen/0000-0003-4131-579X

FU National Institute of Neurological Disorders and Stroke (NINDS) [1RO1

NS055086-01A2]

FX This work was funded by National Institute of Neurological Disorders and

Stroke (NINDS 1RO1 NS055086-01A2).

CR ADAMS JH, 1989, HISTOPATHOLOGY, V15, P49, DOI 10.1111/j.1365-2559.1989.tb03040.x

Alvarez-Buylla A, 2002, J NEUROSCI, V22, P629, DOI 10.1523/JNEUROSCI.22-03-00629.2002

Alvarez-Buylla A, 2004, NEURON, V41, P683, DOI 10.1016/S0896-6273(04)00111-4

ALVAREZBUYLLA A, 1992, EXP NEUROL, V115, P110, DOI 10.1016/0014-4886(92)90232-F

Ao Q, 2007, MED HYPOTHESES, V69, P1234, DOI 10.1016/j.mehy.2007.04.011

Arsenijevic Y, 2001, EXP NEUROL, V170, P48, DOI 10.1006/exnr.2001.7691

Bachoud-Levi AC, 2006, LANCET NEUROL, V5, P303, DOI 10.1016/S1474-4422(06)70381-7

Bakshi A, 2006, EUR J NEUROSCI, V23, P2119, DOI 10.1111/j.1460-9568.2006.04743.x

Becerra GD, 2007, BEHAV BRAIN RES, V179, P118, DOI 10.1016/j.bbr.2007.01.024

Bjorklund A, 2003, LANCET NEUROL, V2, P437, DOI 10.1016/S1474-4422(03)00442-3

Boockvar JA, 2005, NEUROSURGERY, V56, P163, DOI 10.1227/01.NEU.0000145866.25433.FF

Chirumamilla S, 2002, J NEUROTRAUM, V19, P693, DOI 10.1089/08977150260139084

Clark RSB, 2000, J NEUROCHEM, V74, P740, DOI 10.1046/j.1471-4159.2000.740740.x

Dash PK, 2001, J NEUROSCI RES, V63, P313, DOI 10.1002/1097-4547(20010215)63:4<313::AID-JNR1025>3.3.CO;2-W

Driscoll I, 2006, NEUROSCIENCE, V139, P1173, DOI 10.1016/j.neuroscience.2006.01.040

Dunnett S B, 2000, Prog Brain Res, V127, P345

Edwards P, 2005, LANCET, V365, P1957

Ekdahl CT, 2003, P NATL ACAD SCI USA, V100, P13632, DOI 10.1073/pnas.2234031100

Emery DL, 2005, J NEUROTRAUM, V22, P978, DOI 10.1089/neu.2005.22.978

Eriksson PS, 1998, NAT MED, V4, P1313, DOI 10.1038/3305

Falo MC, 2006, J NEUROSCI RES, V84, P768, DOI 10.1002/jnr.20986

Feron F, 2005, BRAIN, V128, P2951, DOI 10.1093/brain/awh657

Fitch MT, 2008, EXP NEUROL, V209, P294, DOI 10.1016/j.expneurol.2007.05.014

Freed CR, 2001, NEW ENGL J MED, V344, P710, DOI 10.1056/NEJM200103083441002

Gao JL, 2006, EXP NEUROL, V201, P281, DOI 10.1016/j.expneurol.2006.04.039

Gennarelli, 1998, Semin Clin Neuropsychiatry, V3, P160

Goldman S, 2005, NAT BIOTECHNOL, V23, P862, DOI 10.1038/nbt1119

Hagell P, 2002, NAT NEUROSCI, V5, P627, DOI 10.1038/nn863

Hastings NB, 1999, J COMP NEUROL, V413, P146, DOI 10.1002/(SICI)1096-9861(19991011)413:1<146::AID-CNE10>3.0.CO;2-B

He ZG, 2004, ANNU REV NEUROSCI, V27, P341, DOI 10.1146/annurev.neuro.27.070203.144340

Heine VM, 2004, NEUROBIOL AGING, V25, P361, DOI 10.1016/S0197-4580(03)00090-3

Hicks RR, 1997, MOL BRAIN RES, V48, P401, DOI 10.1016/S0169-328X(97)00158-7

Hoane MR, 2004, J NEUROTRAUM, V21, P163, DOI 10.1089/089771504322778622

Ibrahim A, 2006, LANCET NEUROL, V5, P453, DOI 10.1016/S1474-4422(06)70444-6

Itoh T, 2005, NEUROREPORT, V16, P1687, DOI 10.1097/01.wnr.0000183330.44112.ab

Kelley BJ, 2007, J NEUROPATH EXP NEUR, V66, P989, DOI 10.1097/NEN.0b013e3181588245

Kempermann G, 1998, J NEUROSCI, V18, P3206

Kendall A L, 2000, Prog Brain Res, V127, P381

Kernie SG, 2001, J NEUROSCI RES, V66, P317, DOI 10.1002/jnr.10013

Kleindienst A, 2005, J NEUROTRAUM, V22, P645, DOI 10.1089/neu.2005.22.645

Kleindienst A, 2004, J NEUROTRAUM, V21, P541, DOI 10.1089/089771504774129874

Kleindienst A, 2006, J NEUROTRAUM, V23, P1185, DOI 10.1089/neu.2006.23.1185

Kondziolka D, 2000, NEUROLOGY, V55, P565, DOI 10.1212/WNL.55.4.565

Kondziolka D, 2005, J NEUROSURG, V103, P38, DOI 10.3171/jns.2005.103.1.0038

Kornack DR, 2001, P NATL ACAD SCI USA, V98, P4752, DOI 10.1073/pnas.081074998

KOTAPKA MJ, 1992, ACTA NEUROPATHOL, V83, P530, DOI 10.1007/BF00310031

KOTAPKA MJ, 1994, J NEUROTRAUM, V11, P317, DOI 10.1089/neu.1994.11.317

Kuhn HG, 1996, J NEUROSCI, V16, P2027

Laurer HL, 1999, CURR OPIN NEUROL, V12, P715, DOI 10.1097/00019052-199912000-00010

Lenzlinger PM, 2005, NEUROSCIENCE, V134, P1047, DOI 10.1016/j.neuroscience.2005.04.048

Leuner B, 2006, HIPPOCAMPUS, V16, P216, DOI 10.1002/hipo.20153

Lie DC, 2004, ANNU REV PHARMACOL, V44, P399, DOI 10.1146/annurev.pharmtox.44.101802.121631

Lim DA, 2007, NEUROSURG CLIN N AM, V18, P81, DOI 10.1016/j.nec.2006.10.002

Lindvall Olle, 2004, NeuroRx, V1, P382

Lu DY, 2005, J NEUROTRAUM, V22, P1011, DOI 10.1089/neu.2005.22.1011

Lu DY, 2003, J NEUROSURG, V99, P351, DOI 10.3171/jns.2003.99.2.0351

Magavi SS, 2000, NATURE, V405, P951, DOI 10.1038/35016083

Manganas LN, 2007, SCIENCE, V318, P980, DOI 10.1126/science.1147851

Manley GT, 2006, J NEUROTRAUM, V23, P128, DOI 10.1089/neu.2006.23.128

Markakis EA, 1999, J COMP NEUROL, V406, P449

Marklund N, 2006, EXP NEUROL, V197, P70, DOI 10.1016/j.expneurol.2005.08.029

Marklund N, 2007, J NEUROSURG, V107, P844, DOI 10.3171/JNS-07/10/0844

McIntosh TK, 1998, NEUROPATH APPL NEURO, V24, P251

Mignone RG, 2006, BRAIN RES, V1111, P26, DOI 10.1016/j.brainres.2006.06.093

Molcanyi M, 2007, J NEUROTRAUM, V24, P625, DOI 10.1089/neu.2006.0180

Monje ML, 2003, SCIENCE, V302, P1760, DOI 10.1126/science.1088417

Morales DM, 2005, NEUROSCIENCE, V136, P971, DOI 10.1016/j.neuroscience.2005.08.030

Morganti-Kossmann MC, 2007, INJURY, V38, P1392, DOI 10.1016/j.injury.2007.10.005

Muir JK, 1999, J NEUROTRAUM, V16, P403, DOI 10.1089/neu.1999.16.403

Myckatyn TM, 2004, TRANSPL IMMUNOL, V12, P343, DOI 10.1016/j.trim.2003.12.017

Nunes MC, 2003, NAT MED, V9, P439, DOI 10.1038/nm837

Olanow CW, 2003, ANN NEUROL, V54, P403, DOI 10.1002/ana.10720

Olson AK, 2006, HIPPOCAMPUS, V16, P250, DOI 10.1002/hipo.20157

Oyesiku NM, 1999, BRAIN RES, V833, P161, DOI 10.1016/S0006-8993(99)01501-2

Parent JM, 2003, NEUROSCIENTIST, V9, P261, DOI 10.1177/1073858403252680

Pencea V, 2001, EXP NEUROL, V172, P1, DOI 10.1006/exnr.2001.7768

Philips MF, 1999, J NEUROSURG, V90, P116, DOI 10.3171/jns.1999.90.1.0116

POVLISHOCK JT, 1994, J NEUROTRAUM, V11, P723, DOI 10.1089/neu.1994.11.723

Povlishock JT, 2005, J HEAD TRAUMA REHAB, V20, P76, DOI 10.1097/00001199-200501000-00008

Prins ML, 1996, DEV BRAIN RES, V95, P272, DOI 10.1016/0165-3806(96)00098-3

Quinones-Hinojosa A, 2006, J COMP NEUROL, V494, P415, DOI 10.1002/cne.20798

Rabinovich SS, 2003, BIOMED PHARMACOTHER, V57, P428, DOI 10.1016/j.biopha.2003.05.001

Ramaswamy S, 2005, BRAIN RES, V1053, P38, DOI 10.1016/j.brainres.2005.06.042

REYNOLDS BA, 1992, SCIENCE, V255, P1707, DOI 10.1126/science.1553558

Rice AC, 2003, EXP NEUROL, V183, P406, DOI 10.1016/S0014-4886(03)00241-3

Richardson RM, 2007, NEUROSURG CLIN N AM, V18, P169, DOI 10.1016/j.nec.2006.10.007

Richardson RM, 2006, ACTA NEUROCHIR, V148, P773, DOI 10.1007/s00701-006-0778-5

Richardson RM, 2005, MOL CELL NEUROSCI, V28, P674, DOI 10.1016/j.mcn.2004.11.013

Richardson RM, 2004, J NEUROSURG, V100, P659, DOI 10.3171/jns.2004.100.4.0659

Riess P, 2002, NEUROSURGERY, V51, P1043, DOI 10.1097/00006123-200210000-00035

Rose VL, 1999, AM FAM PHYSICIAN, V59, P1051

Roy NS, 2000, NAT MED, V6, P271

Sahuquillo J, 2001, CURR PHARM DESIGN, V7, P1475, DOI 10.2174/1381612013397311

Salman H, 2004, J NEUROTRAUM, V21, P283, DOI 10.1089/089771504322972077

Sanai N, 2004, NATURE, V427, P740, DOI 10.1038/nature02301

Sanai N, 2007, SCIENCE, V318, DOI 10.1126/science.1145011

Sawamoto K, 2006, SCIENCE, V311, P629, DOI 10.1126/science.1119133

Schouten JW, 2004, J NEUROTRAUM, V21, P1501, DOI 10.1089/0897715042441774

Seri B, 2001, J NEUROSCI, V21, P7153, DOI 10.1523/JNEUROSCI.21-18-07153.2001

Shah SA, 2006, EXP GERONTOL, V41, P1201, DOI 10.1016/j.exger.2006.07.006

Shear DA, 2004, BRAIN RES, V1026, P11, DOI 10.1016/j.brainres.2004.07.087

Shors TJ, 2001, NATURE, V410, P372, DOI 10.1038/35066584

Silver J, 2004, NAT REV NEUROSCI, V5, P146, DOI 10.1038/nrn1326

Singleton RH, 2002, J NEUROSCI, V22, P791, DOI 10.1523/JNEUROSCI.22-03-00791.2002

Skold MK, 2005, J NEUROTRAUM, V22, P353, DOI 10.1089/neu.2005.22.353

Smith DH, 1991, J NEUROTRAUM, V8, P259, DOI 10.1089/neu.1991.8.259

Smith FM, 2000, ACTA NEUROPATHOL, V100, P537, DOI 10.1007/s004010000222

Stahel PF, 1998, BRAIN RES REV, V27, P243, DOI 10.1016/S0165-0173(98)00015-0

Sun D, 2005, J NEUROTRAUM, V22, P95, DOI 10.1089/neu.2005.22.95

Sun D, 2007, EXP NEUROL, V204, P264, DOI 10.1016/j.expneurol.2006.11.005

Tate CC, 2007, J NEUROTRAUM, V24, P226, DOI 10.1089/neu.2006.0043

Tate MC, 2002, CELL TRANSPLANT, V11, P283

Tolias Christos M, 2004, NeuroRx, V1, P71, DOI 10.1007/BF03206568

Truettner J, 1999, J NEUROTRAUM, V16, P471, DOI 10.1089/neu.1999.16.471

Umile EM, 2002, ARCH PHYS MED REHAB, V83, P1506, DOI 10.1053/apmr.2002.35092

Urrea C, 2007, RESTOR NEUROL NEUROS, V25, P65

van Praag H, 2002, NATURE, V415, P1030, DOI 10.1038/4151030a

Walton NM, 2006, DEVELOPMENT, V133, P3671, DOI 10.1242/dev.02541

Wang YM, 2007, J NEUROSCI RES, V85, P740, DOI 10.1002/jnr.21169

Watson DJ, 2003, J NEUROPATH EXP NEUR, V62, P368, DOI 10.1093/jnen/62.4.368

Will B, 2004, PROG NEUROBIOL, V72, P167, DOI 10.1016/j.pneurobio.2004.03.001

Williams S, 2001, ACTA NEUROPATHOL, V102, P581, DOI 10.1007/s004010100410

Windrem MS, 2002, J NEUROSCI RES, V69, P966, DOI 10.1002/jnr.10397

Wirth ED, 2001, J NEUROTRAUM, V18, P911, DOI 10.1089/089771501750451839

Yang K, 1996, J NEUROSCI RES, V44, P157, DOI 10.1002/(SICI)1097-4547(19960415)44:2<157::AID-JNR8>3.0.CO;2-C

Yoon SH, 2007, STEM CELLS, V25, P2066, DOI 10.1634/stemcells.2006-0807

Yoshimura S, 2003, J CLIN INVEST, V112, P1202, DOI 10.1172/JCI200316618

Zhang CL, 2008, NATURE, V451, P1004, DOI 10.1038/nature06562

Zhu J, 2005, CURR DRUG TARGETS, V6, P97, DOI 10.2174/1389450053345055

Zhu JH, 2006, NEW ENGL J MED, V355, P2376, DOI 10.1056/NEJMc055304

NR 130

TC 86

Z9 92

U1 0

U2 21

PU AMER ASSOC NEUROLOGICAL SURGEONS

PI ROLLING MEADOWS

PA 5550 MEADOWBROOK DRIVE, ROLLING MEADOWS, IL 60008 USA

SN 0022-3085

EI 1933-0693

J9 J NEUROSURG

JI J. Neurosurg.

PD MAY

PY 2010

VL 112

IS 5

BP 1125

EP 1138

DI 10.3171/2009.4.JNS081087

PG 14

WC Clinical Neurology; Surgery

WE Science Citation Index Expanded (SCI-EXPANDED)

SC Neurosciences & Neurology; Surgery

GA 591BW

UT WOS:000277273600032

PM 19499984

DA 2023-06-10

ER

PT J

AU Xue, S

Zhang, HT

Zhang, P

Luo, J

Chen, ZZ

Jang, XD

Xu, RX

AF Xue, Sha

Zhang, Hong-tian

Zhang, Peng

Luo, Jie

Chen, Zhen-zhou

Jang, Xiao-dan

Xu, Ru-xiang

TI Functional endothelial progenitor cells derived from adipose tissue show

beneficial effect on cell therapy of traumatic brain injury

SO NEUROSCIENCE LETTERS

LA English

DT Article

DE Adipose tissue; Endothelial progenitor cells; Traumatic brain injury

ID MESENCHYMAL STEM-CELLS; UMBILICAL-CORD BLOOD; HUMAN BONE-MARROW;

CEREBRAL-ISCHEMIA; TRANSPLANTATION; RAT; NEOVASCULARIZATION; EXPRESSION;

MICE

AB Endothelial progenitor cells (EPCs) are responsible for postnatal vasculogenesis in physiological and pathological neovascularization. Adipose tissue (AT) is an abundant source of mesenchymal stem cells (MSCs), which have multipotent differentiation ability. We successfully derived EPCs from AT, which maintained a strong proliferative capacity and demonstrated the characteristic endothelial function of uptaking of acetylated low-density lipoprotein. They formed tube-like structures in vitro. Endothelial nitric oxide synthase (eNOS) gene expression in EPCs was similar to that in mature endothelial cells. Transplantation of EPCs derived from AT after the acute phase was applied in rats with traumatic brain injury (TBI). Transplanted EPCs participated in the neovascularization of injured brain. Improving functional recovery, reducement of deficiency volume of brain, host astrogliosis and inflammation were found. These results suggest that adult AT derived stem cells can be induced to functional EPCs and have beneficial effect on cell therapy. (C) 2010 Elsevier Ireland Ltd. All rights reserved.

C1 [Xue, Sha; Zhang, Hong-tian; Zhang, Peng; Luo, Jie; Chen, Zhen-zhou; Jang, Xiao-dan; Xu, Ru-xiang] So Med Univ, Zhujiang Hosp, Dept Neurosurg, Guangzhou 510282, Guangdong, Peoples R China.

[Xue, Sha; Zhang, Hong-tian; Zhang, Peng; Luo, Jie; Chen, Zhen-zhou; Jang, Xiao-dan; Xu, Ru-xiang] So Med Univ, Inst Neurosurg, Key Lab Brain Funct Repair & Regenerat Guangdong, Guangzhou 510282, Guangdong, Peoples R China.

[Xu, Ru-xiang] Mil Gen Hosp Beijing PLA, Dept Neurosurg, Beijing 100700, Peoples R China.

C3 Southern Medical University - China; Southern Medical University - China

RP Jang, XD (通讯作者)，So Med Univ, Zhujiang Hosp, Dept Neurosurg, 253 Gongye Rd, Guangzhou 510282, Guangdong, Peoples R China.

EM jiangxiao_dan@163.com; zjxuruxiang@163.com

FU Natural Science Foundation of China (NSFC) [U0632008, 30772232,

30801184, 30500526]; Funds for Key Sci-Tech Research Projects of

Guangdong Province [2006Z3-E522, YUE KEJIBAN (2007) 05/06-7005206,

05/06-7005213, YUECAIJIAO (2008) 258-2008A030201019]; Funds for Key

Sci-Tech Research Projects of Guangzhou [YUEKETIAOZI

(2008)3-2008A1-E4011-6, 09B52120112]

FX This research was supported by the Natural Science Foundation of China

(NSFC) (U0632008, 30772232, 30801184, 30500526), Funds for Key Sci-Tech

Research Projects of Guangdong Province [2006Z3-E522, YUE KEJIBAN (2007)

05/06-7005206, 05/06-7005213, YUECAIJIAO (2008) 258-2008A030201019], and

Funds for Key Sci-Tech Research Projects of Guangzhou [YUEKETIAOZI

(2008)3-2008A1-E4011-6, 09B52120112].

CR Asahara T, 1999, CIRC RES, V85, P221, DOI 10.1161/01.RES.85.3.221

Becerra GD, 2007, BEHAV BRAIN RES, V179, P118, DOI 10.1016/j.bbr.2007.01.024

Chen ZZ, 2008, NEUROSCI LETT, V445, P36, DOI 10.1016/j.neulet.2008.08.039

Cheng J, 1996, BLOOD, V87, P479, DOI 10.1182/blood.V87.2.479.bloodjournal872479

FEENEY DM, 1981, BRAIN RES, V211, P67, DOI 10.1016/0006-8993(81)90067-6

Kawamoto Atsuhiko, 2002, Cardiovasc Radiat Med, V3, P221, DOI 10.1016/S1522-1865(03)00082-9

Kern S, 2006, STEM CELLS, V24, P1294, DOI 10.1634/stemcells.2005-0342

Lee RH, 2004, CELL PHYSIOL BIOCHEM, V14, P311, DOI 10.1159/000080341

Loomans CJM, 2006, ARTERIOSCL THROM VAS, V26, P1760, DOI 10.1161/01.ATV.0000229243.49320.c9

Qian C, 2007, CARDIOVASC PATHOL, V16, P127, DOI 10.1016/j.carpath.2006.11.008

Rebelatto CK, 2008, EXP BIOL MED, V233, P901, DOI 10.3181/0712-RM-356

Schatteman GC, 2007, AM J PHYSIOL-HEART C, V292, pH1, DOI 10.1152/ajpheart.00662.2006

Schmittgen TD, 2008, NAT PROTOC, V3, P1101, DOI 10.1038/nprot.2008.73

Shyu WC, 2006, J NEUROSCI, V26, P3444, DOI 10.1523/JNEUROSCI.5165-05.2006

Suzuki A, 1996, BLOOD, V87, P3550, DOI 10.1182/blood.V87.9.3550.bloodjournal8793550

Wagner W, 2005, EXP HEMATOL, V33, P1402, DOI 10.1016/j.exphem.2005.07.003

Yin T, 2008, CELL RES, V18, P792, DOI 10.1038/cr.2008.69

Zhang HT, 2009, NEUROSCI LETT, V458, P116, DOI 10.1016/j.neulet.2009.04.045

Zhang SJ, 2006, CELL RES, V16, P577, DOI 10.1038/sj.cr.7310075

Zhang ZG, 2002, CIRC RES, V90, P284, DOI 10.1161/hh0302.104460

Zuk PA, 2001, TISSUE ENG, V7, P211, DOI 10.1089/107632701300062859

NR 21

TC 38

Z9 44

U1 0

U2 7

PU ELSEVIER IRELAND LTD

PI CLARE

PA ELSEVIER HOUSE, BROOKVALE PLAZA, EAST PARK SHANNON, CO, CLARE, 00000,

IRELAND

SN 0304-3940

EI 1872-7972

J9 NEUROSCI LETT

JI Neurosci. Lett.

PD APR 12

PY 2010

VL 473

IS 3

BP 186

EP 191

DI 10.1016/j.neulet.2010.02.035

PG 6

WC Neurosciences

WE Science Citation Index Expanded (SCI-EXPANDED)

SC Neurosciences & Neurology

GA 588ML

UT WOS:000277074300006

PM 20178832

DA 2023-06-10

ER

PT J

AU Dehghanian, F

Soltani, Z

Khaksari, M

AF Dehghanian, Fatemeh

Soltani, Zahra

Khaksari, Mohammad

TI Can Mesenchymal Stem Cells Act Multipotential in Traumatic Brain Injury?

SO JOURNAL OF MOLECULAR NEUROSCIENCE

LA English

DT Article

DE Mesenchymal stem cells; Traumatic brain injury; Apoptosis; Inflammation

ID TUMOR-NECROSIS-FACTOR; OXIDATIVE STRESS; MOUSE MODEL; NEUROPROTECTION;

TRANSPLANTATION; PATHOPHYSIOLOGY; ACTIVATION; EXPRESSION; ISCHEMIA;

CALCIUM

AB Traumatic brain injury (TBI), a leading cause of morbidity and mortality throughout the world, will probably become the third cause of death in the world by the year 2020. Lack of effective treatments approved for TBI is a major health problem. TBI is a heterogeneous disease due to the different mechanisms of injury. Therefore, it requires combination therapies or multipotential therapy that can affect multiple targets. In recent years, mesenchymal stem cells (MSCs) transplantation has considered one of the most promising therapeutic strategies to repair of brain injuries including TBI. In these studies, it has been shown that MSCs can migrate to the site of injury and differentiate into the cells secreting growth factors and anti-inflammatory cytokines. The reduction in brain edema, neuroinflammation, microglia accumulation, apoptosis, ischemia, the improvement of motor and cognitive function, and the enhancement in neurogenesis, angiogenesis, and neural stem cells survival, proliferation, and differentiation have been indicated in these studies. However, translation of MSCs research in TBI into a clinical setting will require additional preclinical trials.

C1 [Dehghanian, Fatemeh] Kerman Univ Med Sci, Neurosci Res Ctr, Inst Neuropharmacol, Kerman, Iran.

[Dehghanian, Fatemeh] Bam Univ Med Sci, Bam, Iran.

[Soltani, Zahra] Kerman Univ Med Sci, Fac Med, Endocrinol & Metab Res Ctr, Inst Basic & Clin Physiol Sci, Kerman, Iran.

[Khaksari, Mohammad] Kerman Univ Med Sci, Physiol Res Ctr, Inst Neuropharmacol, Kerman, Iran.

C3 Kerman University of Medical Sciences; Kerman University of Medical

Sciences; Kerman University of Medical Sciences

RP Soltani, Z (通讯作者)，Kerman Univ Med Sci, Fac Med, Endocrinol & Metab Res Ctr, Inst Basic & Clin Physiol Sci, Kerman, Iran.

EM soltaniy@yahoo.com

RI Haddad, Mohammad Khaksari/AAB-9025-2019

OI Haddad, Mohammad Khaksari/0000-0003-0770-4281; Soltani,

Zahra/0000-0002-8423-6189

CR Aarts M, 2003, CELL, V115, P863, DOI 10.1016/S0092-8674(03)01017-1

Arnold L, 2007, J EXP MED, V204, P1057, DOI 10.1084/jem.20070075

Bayir H, 2005, J CEREBR BLOOD F MET, V25, P673, DOI 10.1038/sj.jcbfm.9600068

Butovsky O, 2007, MOL CELL NEUROSCI, V35, P490, DOI 10.1016/j.mcn.2007.04.009

Bye N, 2007, EXP NEUROL, V204, P220, DOI 10.1016/j.expneurol.2006.10.013

Chang CP, 2013, CLIN SCI, V124, P165, DOI 10.1042/CS20120226

Chen DY, 2013, BIOMATERIALS, V34, P1995, DOI 10.1016/j.biomaterials.2012.11.045

Chen Q, 2005, J NEUROSCI RES, V80, P611, DOI 10.1002/jnr.20494

Choi H, 2011, BLOOD, V118, P330, DOI 10.1182/blood-2010-12-327353

Chong ZZ, 2005, PROG NEUROBIOL, V75, P207, DOI 10.1016/j.pneurobio.2005.02.004

Corso P, 2006, INJURY PREV, V12, P212, DOI 10.1136/ip.2005.010983

Cox CS, 2018, PEDIATR RES, V83, P325, DOI 10.1038/pr.2017.253

Czigner A, 2007, ACTA NEUROCHIR, V149, P281, DOI 10.1007/s00701-006-1095-8

Dang BQ, 2017, NEURAL PLAST, V2017, DOI 10.1155/2017/1582182

Das M, 2019, REV NEUROSCIENCE, V30, P839, DOI 10.1515/revneuro-2019-0002

Dehghan F, 2018, INFLAMMOPHARMACOLOGY, V26, P1017, DOI 10.1007/s10787-017-0417-1

Dewitt DS, 2003, J NEUROTRAUM, V20, P795, DOI 10.1089/089771503322385755

Dirnagl U, 1999, TRENDS NEUROSCI, V22, P391, DOI 10.1016/S0166-2236(99)01401-0

Dodson MV, 2010, INT J BIOL SCI, V6, P465

Du YY, 2008, CYTOTHERAPY, V10, P469, DOI 10.1080/14653240802129893

Fabricius M, 2006, BRAIN, V129, P778, DOI 10.1093/brain/awh716

Floyd CL, 2005, GLIA, V51, P35, DOI 10.1002/glia.20183

Gage FH, 2013, NEURON, V80, P588, DOI 10.1016/j.neuron.2013.10.037

Galindo LT, 2011, NEUROL RES INT, V2011, DOI 10.1155/2011/564089

Gennai G, 2005, LESSICO INTERCULTURA

GENNARELLI TA, 1982, ANN NEUROL, V12, P564, DOI 10.1002/ana.410120611

Ghajar J, 2000, LANCET, V356, P923, DOI 10.1016/S0140-6736(00)02689-1

Ghosh M, 2016, J NEUROINFLAMM, V13, DOI 10.1186/s12974-015-0463-9

Gibb SL, 2015, STEM CELLS, V33, P3530, DOI 10.1002/stem.2189

Gincberg G, 2018, CYTOTHERAPY, V20, P245, DOI 10.1016/j.jcyt.2017.11.008

Goforth PB, 1999, J NEUROSCI, V19, P7367, DOI 10.1523/JNEUROSCI.19-17-07367.1999

Greve MW, 2009, MT SINAI J MED, V76, P97, DOI 10.1002/msj.20104

Guo SW, 2017, NEUROPSYCH DIS TREAT, V13, P2757, DOI 10.2147/NDT.S141534

Gupta N, 2007, J IMMUNOL, V179, P1855, DOI 10.4049/jimmunol.179.3.1855

Hall ED, 2010, NEUROTHERAPEUTICS, V7, P51, DOI 10.1016/j.nurt.2009.10.021

Herson PS, 1999, J BIOL CHEM, V274, P833, DOI 10.1074/jbc.274.2.833

Higuchi M, 2005, J BIOL CHEM, V280, P15229, DOI 10.1074/jbc.M500939200

Hlatky R, 2002, J NEUROSURG, V97, P1054, DOI 10.3171/jns.2002.97.5.1054

Hofer HR, 2016, STEM CELL RES THER, V7, DOI 10.1186/s13287-016-0394-0

Itoh T, 2005, NEUROREPORT, V16, P1687, DOI 10.1097/01.wnr.0000183330.44112.ab

Johnson VE, 2013, BRAIN, V136, P28, DOI 10.1093/brain/aws322

Jorge RE, 2004, ARCH GEN PSYCHIAT, V61, P42, DOI 10.1001/archpsyc.61.1.42

Khaksari M, 2018, TRANSL STROKE RES, V9, P393, DOI 10.1007/s12975-017-0588-5

Khaksari M, 2018, IRAN J BASIC MED SCI, V21, P615, DOI 10.22038/IJBMS.2018.26586.6512

Kim C, 2018, MOL NEUROBIOL, V55, P4870, DOI 10.1007/s12035-017-0683-3

Kim HJ, 2010, J NEUROTRAUM, V27, P131, DOI [10.1089/neu.2008.0818, 10.1089/neu.2008-0818]

Kobeissy F, 2015, BRAIN NEUROTRAUMA MO

Konsman JP, 2007, CLIN SCI, V112, P1, DOI 10.1042/CS20060043

Kota DJ, 2017, STEM CELLS, V35, P1416, DOI 10.1002/stem.2603

Koutsoudaki PN, 2016, GLIA, V64, P763, DOI 10.1002/glia.22959

Kroemer G, 2000, NAT MED, V6, P513, DOI 10.1038/74994

Kubes P, 2000, BRAIN PATHOL, V10, P127

Kwon YW, 2013, BBA-MOL BASIS DIS, V1832, P2136, DOI 10.1016/j.bbadis.2013.08.002

Lang EW, 2003, CRIT CARE MED, V31, P267, DOI 10.1097/00003246-200301000-00042

Lee JY, 2019, THERANOSTICS, V9, P1029, DOI 10.7150/thno.29868

Li G, 2018, TURK NEUROSURG, V28, P696, DOI 10.5137/1019-5149.JTN.20829-17.1

Liao YWK, 2013, PLOS ONE, V8, DOI 10.1371/journal.pone.0076090

Liu N, 2009, CELL MOL IMMUNOL, V6, P207, DOI 10.1038/cmi.2009.28

Longhi L, 2013, J CEREBR BLOOD F MET, V33, P1182, DOI 10.1038/jcbfm.2013.65

Ponte AL, 2007, STEM CELLS, V25, P1737, DOI 10.1634/stemcells.2007-0054

Lumpkins K, 2008, J TRAUMA, V64, P358, DOI 10.1097/TA.0b013e318160df9b

Maas AIR, 2010, NEUROTHERAPEUTICS, V7, P115, DOI 10.1016/j.nurt.2009.10.022

Marmarou A, 2000, J NEUROSURG, V93, P183, DOI 10.3171/jns.2000.93.2.0183

Marmarou A, 2006, J NEUROSURG, V104, P720, DOI 10.3171/jns.2006.104.5.720

Marmarou Anthony, 2007, Neurosurg Focus, V22, pE1

Marshall LF, 2000, HEAD INJURY RECENT P

Mastro-Martinez I, 2015, BRAIN INJURY, V29, P1497, DOI 10.3109/02699052.2015.1053525

Meirelles LD, 2009, CYTOKINE GROWTH F R, V20, P419, DOI 10.1016/j.cytogfr.2009.10.002

Menge T, 2012, SCI TRANSL MED, V4, DOI 10.1126/scitranslmed.3004660

Meymandi MS, 2018, BRAIN RES BULL, V140, P169, DOI 10.1016/j.brainresbull.2018.05.001

Morganti-Kossmann C, 2010, ELSEV INSIGHT, P193, DOI 10.1016/B978-0-12-384691-4.00010-9

Morganti-Kossmann MC, 2001, SHOCK, V16, P165, DOI 10.1097/00024382-200116030-00001

Mustafa AG, 2013, NEUROSCIENCES, V18, P222

Mustafa AG, 2010, J NEUROCHEM, V114, P271, DOI 10.1111/j.1471-4159.2010.06749.x

Nahrendorf M, 2007, J EXP MED, V204, P3037, DOI 10.1084/jem.20070885

Neuss S, 2004, STEM CELLS, V22, P405, DOI 10.1634/stemcells.22-3-405

Ni HQ, 2019, FRONT NEUROSCI-SWITZ, V13, DOI 10.3389/fnins.2019.00014

Nortje J, 2004, CURR OPIN NEUROL, V17, P711, DOI 10.1097/00019052-200412000-00011

O'Connor WT, 2011, PHARMACOL THERAPEUT, V130, P106, DOI 10.1016/j.pharmthera.2011.01.001

O'Phelan KH, 2009, NEUROCRIT CARE, V10, P280, DOI 10.1007/s12028-008-9183-7

Ooi YY, 2010, INT IMMUNOPHARMACOL, V10, P1532, DOI 10.1016/j.intimp.2010.09.001

Ortiz LA, 2007, P NATL ACAD SCI USA, V104, P11002, DOI 10.1073/pnas.0704421104

Paschen W, 1999, J CEREBR BLOOD F MET, V19, P1, DOI 10.1097/00004647-199901000-00001

Qi LF, 2018, J CRANIOFAC SURG, V29, P1689, DOI 10.1097/SCS.0000000000005042

Racay P, 2009, NEUROCHEM RES, V34, P1469, DOI 10.1007/s11064-009-9934-7

Reis C, 2017, STEM CELLS INT, V2017, DOI 10.1155/2017/6392592

Rhodes JKJ, 2009, J NEUROTRAUM, V26, P507, DOI 10.1089/neu.2008.0686

Riess P, 2002, NEUROSURGERY, V51, P1043, DOI 10.1097/00006123-200210000-00035

Rosova I, 2008, STEM CELLS, V26, P2173, DOI 10.1634/stemcells.2007-1104

Rossetti MF, 2016, J NEUROENDOCRINOL, V28, DOI 10.1111/jne.12402

Russo MV, 2016, SCIENCE, V353, P783, DOI 10.1126/science.aaf6260

Sato A, 2012, J NEUROINFLAMM, V9, DOI 10.1186/1742-2094-9-65

Schmidt EA, 2003, J NEUROSURG, V99, P991, DOI 10.3171/jns.2003.99.6.0991

Schneider C. M., 2019, PRINCIPLES REGENERAT, P369

Schouten JW, 2007, CURR OPIN CRIT CARE, V13, P134, DOI 10.1097/MCC.0b013e3280895d5c

Semple BD, 2010, J CEREBR BLOOD F MET, V30, P769, DOI 10.1038/jcbfm.2009.262

Shao CX, 2006, FREE RADICAL BIO MED, V41, P77, DOI 10.1016/j.freeradbiomed.2006.03.007

Shear DA, 2011, RESTOR NEUROL NEUROS, V29, P215, DOI 10.3233/RNN-2011-0593

Singh IN, 2013, J CEREBR BLOOD F MET, V33, P593, DOI 10.1038/jcbfm.2012.211

Skendelas JP, 2015, CURRENT PHYS MED REH, V3, P115, DOI [10.1007/s40141-015-0091-4, DOI 10.1007/S40141-015-0091-4]

Soltani Z, 2017, BRAIN INJURY, V31, P16, DOI 10.1080/02699052.2016.1213421

Soltani Z, 2015, PHYSIOL BEHAV, V152, P26, DOI 10.1016/j.physbeh.2015.08.037

Stiefel MF, 2005, J NEUROSURG, V103, P707, DOI 10.3171/jns.2005.103.4.0707

Szydlowska K, 2010, CELL CALCIUM, V47, P122, DOI 10.1016/j.ceca.2010.01.003

Tagliaferri F, 2006, ACTA NEUROCHIR, V148, P255, DOI 10.1007/s00701-005-0651-y

Tajiri N, 2014, J NEUROSCI, V34, P313, DOI 10.1523/JNEUROSCI.2425-13.2014

Tajiri N, 2013, PLOS ONE, V8, DOI 10.1371/journal.pone.0074857

Tao X, 1998, NEURON, V20, P709, DOI 10.1016/S0896-6273(00)81010-7

Torrente D, 2014, HUM EXP TOXICOL, V33, P673, DOI 10.1177/0960327113509659

Unterberg AW, 2004, NEUROSCIENCE, V129, P1021, DOI 10.1016/j.neuroscience.2004.06.046

Van Landeghem FKH, 2006, J NEUROTRAUM, V23, P1518, DOI 10.1089/neu.2006.23.1518

Wan Jiangbo, 2012, Nan Fang Yi Ke Da Xue Xue Bao, V32, P1730

Wang SY, 2013, INT J DEV NEUROSCI, V31, P30, DOI 10.1016/j.ijdevneu.2012.09.004

Webster KM, 2017, J NEUROINFLAMM, V14, DOI 10.1186/s12974-016-0786-1

Werner C, 2007, BRIT J ANAESTH, V99, P4, DOI 10.1093/bja/aem131

Westermann B, 2010, NAT REV MOL CELL BIO, V11, P872, DOI 10.1038/nrm3013

Wu JG, 2016, INT J CLIN EXP MED, V9, P12649

Xiong Y, 2008, BRAIN RES, V1230, P247, DOI 10.1016/j.brainres.2008.06.127

Xiong ZG, 2004, CELL, V118, P687, DOI 10.1016/j.cell.2004.08.026

Yin Y, 2017, AGEING RES REV, V34, P3, DOI 10.1016/j.arr.2016.08.008

Zhang R, 2013, J NEUROINFLAMM, V10, DOI 10.1186/1742-2094-10-106

Zhang YL, 2017, NEUROCHEM INT, V111, P69, DOI 10.1016/j.neuint.2016.08.003

Zhao K, 2017, STEM CELLS INT, V2017, DOI 10.1155/2017/2153629

Zhao YH, 2016, STEM CELLS, V34, P1263, DOI 10.1002/stem.2310

Ziebell JM, 2010, NEUROTHERAPEUTICS, V7, P22, DOI 10.1016/j.nurt.2009.10.016

NR 125

TC 15

Z9 15

U1 2

U2 8

PU HUMANA PRESS INC

PI TOTOWA

PA 999 RIVERVIEW DRIVE SUITE 208, TOTOWA, NJ 07512 USA

SN 0895-8696

EI 1559-1166

J9 J MOL NEUROSCI

JI J. Mol. Neurosci.

PD MAY

PY 2020

VL 70

IS 5

BP 677

EP 688

DI 10.1007/s12031-019-01475-w

EA JAN 2020

PG 12

WC Biochemistry & Molecular Biology; Neurosciences

WE Science Citation Index Expanded (SCI-EXPANDED)

SC Biochemistry & Molecular Biology; Neurosciences & Neurology

GA LC8FQ

UT WOS:000505463700004

PM 31897971

DA 2023-06-10

ER

PT J

AU Sun, D

Gugliotta, M

Rolfe, A

Reid, W

McQuiston, AR

Hu, WH

Young, H

AF Sun, Dong

Gugliotta, Marinella

Rolfe, Andrew

Reid, Wendy

McQuiston, A. Rory

Hu, Wenhui

Young, Harold

TI Sustained Survival and Maturation of Adult Neural Stem/Progenitor Cells

after Transplantation into the Injured Brain

SO JOURNAL OF NEUROTRAUMA

LA English

DT Article

DE neural stem/progenitor cells; subventricular zone; transplantation;

traumatic brain injury

ID MARROW STROMAL CELLS; PROGENITOR CELLS; STEM-CELLS; INFLAMMATORY

RESPONSE; COGNITIVE RECOVERY; RAT-BRAIN; DIFFERENTIATION; NEURONS;

MIGRATION; IMPROVES

AB Multipotent neural stem/progenitor cells (NS/NPCs) that are capable of generating neurons and glia offer enormous potential for treating neurological diseases. Adult NS/NPCs that reside in the mature mammalian brain can be isolated and expanded in vitro, and could be a potential source for autologous transplantation to replace cells lost to brain injury or disease. When these cells are transplanted into the normal brain, they can survive and become region-specific cells. However, it has not been reported whether these cells can survive for an extended period and become functional cells in an injured heterotypic environment. In this study, we tested survival, maturation fate, and electrophysiological properties of adult NS/NPCs after transplantation into the injured rat brain. NS/NPCs were isolated from the subventricular zone of adult Fisher 344 rats and cultured as a monolayer. Recipient adult Fisher 344 rats were first subjected to a moderate fluid percussive injury. Two days later, cultured NS/NPCs were injected into the injured brain in an area between the white matter tracts and peri-cortical region directly underneath the injury impact. The animals were sacrificed 2 or 4 weeks after transplantation for immunohistochemical staining or patch-clamp recording. We found that transplanted cells survived well at 2 and 4 weeks. Many cells migrated out of the injection site into surrounding areas expressing astrocyte or oligodendrocyte markers. Whole cell patch-clamp recording at 4 weeks showed that transplanted cells possessed typical mature glial cell properties. These data demonstrate that adult NS/NPCs can survive in an injured heterotypic environment for an extended period and become functional cells.

C1 [Sun, Dong; Gugliotta, Marinella; Rolfe, Andrew; Reid, Wendy; Young, Harold] Virginia Commonwealth Univ, Dept Neurosurg, Richmond, VA 23298 USA.

[McQuiston, A. Rory] Virginia Commonwealth Univ, Dept Anat & Neurobiol, Richmond, VA 23298 USA.

[Hu, Wenhui] Temple Univ, Dept Neurosci, Philadelphia, PA 19122 USA.

C3 Virginia Commonwealth University; Virginia Commonwealth University;

Pennsylvania Commonwealth System of Higher Education (PCSHE); Temple

University

RP Sun, D (通讯作者)，Virginia Commonwealth Univ, Dept Neurosurg, POB 980631,Med Coll Virginia Campus, Richmond, VA 23298 USA.

EM dsun@vcu.edu

RI McQuiston, Adam Rory/A-3072-2008

FU A.D. William Fund; National Institutes of Health/National Institute of

Neurological Disorders and Stroke (NIH/NINDS) [NS062369, NS055086,

5P30NS047463]; Virginia Commonwealth Neurotrauma Initiative Trust

[RFP07-302]

FX Sponsored by the A.D. William Fund (Sun), National Institutes of

Health/National Institute of Neurological Disorders and Stroke

(NIH/NINDS) grant NS062369 (Sun), NIH/NINDS grant NS055086 (Sun), and

the Virginia Commonwealth Neurotrauma Initiative Trust Fund (RFP07-302,

Sun). Microscopy work was performed at the VCU-Department of Anatomy and

Neurobiology Microscopy Facility, supported in part by funding from

NIH/NINDS center core grant 5P30NS047463.

CR ALTMAN J, 1965, J COMP NEUROL, V124, P319, DOI 10.1002/cne.901240303

Bjorklund A, 2000, BRAIN RES, V886, P82, DOI 10.1016/S0006-8993(00)02915-2

Boockvar JA, 2005, NEUROSURGERY, V56, P163, DOI 10.1227/01.NEU.0000145866.25433.FF

Dziewczapolski G, 2003, EXP NEUROL, V183, P653, DOI 10.1016/S0014-4886(03)00212-7

GAGE FH, 1995, P NATL ACAD SCI USA, V92, P11879, DOI 10.1073/pnas.92.25.11879

Gage FH, 1998, J NEUROBIOL, V36, P249, DOI 10.1002/(SICI)1097-4695(199808)36:2<249::AID-NEU11>3.0.CO;2-9

Gao JL, 2006, EXP NEUROL, V201, P281, DOI 10.1016/j.expneurol.2006.04.039

Gates M A, 2000, Prog Brain Res, V127, P115

Hagan M, 2003, NEUROSCI LETT, V351, P149, DOI 10.1016/j.neulet.2003.07.021

Herrera DG, 1999, ANN NEUROL, V46, P867, DOI 10.1002/1531-8249(199912)46:6<867::AID-ANA9>3.0.CO;2-Z

Hill-Felberg SJ, 1999, J NEUROSCI RES, V57, P271, DOI 10.1002/(SICI)1097-4547(19990715)57:2<271::AID-JNR13>3.0.CO;2-Z

Hoane MR, 2004, J NEUROTRAUM, V21, P163, DOI 10.1089/089771504322778622

Hu W, 2006, BIOCHEM BIOPH RES CO, V343, P1038, DOI 10.1016/j.bbrc.2006.03.079

Klassen H, 2003, VISION RES, V43, P947, DOI 10.1016/S0042-6989(03)00094-4

Lenzlinger PM, 2001, MOL NEUROBIOL, V24, P169

Li Y, 2009, NEUROSCI LETT, V456, P120, DOI 10.1016/j.neulet.2008.03.096

Ligon KL, 2004, J NEUROPATH EXP NEUR, V63, P499, DOI 10.1093/jnen/63.5.499

LOIS C, 1993, P NATL ACAD SCI USA, V90, P2074, DOI 10.1073/pnas.90.5.2074

Mahmood A, 2006, J NEUROSURG, V104, P272, DOI 10.3171/jns.2006.104.2.272

Mahmood A, 2004, J NEUROTRAUM, V21, P33, DOI 10.1089/089771504772695922

Mahmood A, 2003, NEUROSURGERY, V53, P697, DOI 10.1227/01.NEU.0000079333.61863.AA

Mahmood A, 2001, NEUROSURGERY, V49, P1196, DOI 10.1097/00006123-200111000-00031

Mahmood A, 2001, J NEUROSURG, V94, P589, DOI 10.3171/jns.2001.94.4.0589

McKay R, 1997, SCIENCE, V276, P66, DOI 10.1126/science.276.5309.66

McQuiston AR, 2007, J NEUROPHYSIOL, V97, P2301, DOI 10.1152/jn.01179.2006

Molcanyi M, 2007, J NEUROTRAUM, V24, P625, DOI 10.1089/neu.2006.0180

PALMER TD, 1995, MOL CELL NEUROSCI, V6, P474, DOI 10.1006/mcne.1995.1035

Philips MF, 2001, J NEUROSURG, V94, P765, DOI 10.3171/jns.2001.94.5.0765

Qu CS, 2009, J NEUROSURG, V111, P658, DOI 10.3171/2009.4.JNS081681

Reid WM, 2010, J NEUROTRAUM, V27, P1243, DOI 10.1089/neu.2010.1270

Richardson RM, 2005, MOL CELL NEUROSCI, V28, P674, DOI 10.1016/j.mcn.2004.11.013

Richardson RM, 2005, BRAIN RES, V1032, P11, DOI 10.1016/j.brainres.2004.10.043

Riess P, 2002, NEUROSURGERY, V51, P1043, DOI 10.1097/00006123-200210000-00035

Riess P, 2007, J NEUROTRAUM, V24, P216, DOI 10.1089/neu.2006.0141

Shear DA, 2004, BRAIN RES, V1026, P11, DOI 10.1016/j.brainres.2004.07.087

Sinson G, 1996, J NEUROSURG, V84, P655, DOI 10.3171/jns.1996.84.4.0655

Soares HD, 1995, J NEUROSCI, V15, P8223

SONTHEIMER H, 1994, GLIA, V11, P156, DOI 10.1002/glia.440110210

Studer L, 1998, NAT NEUROSCI, V1, P290, DOI 10.1038/1105

Suhonen JO, 1996, NATURE, V383, P624, DOI 10.1038/383624a0

Sun D, 2007, EXP NEUROL, V204, P264, DOI 10.1016/j.expneurol.2006.11.005

Sun D, 2010, J NEUROTRAUM, V27, P923, DOI 10.1089/neu.2009.1209

Sun D, 2009, EXP NEUROL, V216, P56, DOI 10.1016/j.expneurol.2008.11.011

Tate CC, 2009, J TISSUE ENG REGEN M, V3, P208, DOI 10.1002/term.154

Tate MC, 2002, CELL TRANSPLANT, V11, P283

Wallenquist U, 2009, RESTOR NEUROL NEUROS, V27, P323, DOI 10.3233/RNN-2009-0481

Wennersten A, 2004, J NEUROSURG, V100, P88, DOI 10.3171/jns.2004.100.1.0088

NR 47

TC 27

Z9 29

U1 0

U2 6

PU MARY ANN LIEBERT INC

PI NEW ROCHELLE

PA 140 HUGUENOT STREET, 3RD FL, NEW ROCHELLE, NY 10801 USA

SN 0897-7151

J9 J NEUROTRAUM

JI J. Neurotrauma

PD JUN

PY 2011

VL 28

IS 6

BP 961

EP 972

DI 10.1089/neu.2010.1697

PG 12

WC Critical Care Medicine; Clinical Neurology; Neurosciences

WE Science Citation Index Expanded (SCI-EXPANDED)

SC General & Internal Medicine; Neurosciences & Neurology

GA 788PT

UT WOS:000292457600010

PM 21332258

OA Green Published, Green Submitted

DA 2023-06-10

ER

PT J

AU Zhang, B

Zhu, XL

Wang, L

Hao, SY

Xu, XJ

Niu, F

He, W

Liu, BY

AF Zhang, Bin

Zhu, Xueli

Wang, Liang

Hao, Shuyu

Xu, Xiaojian

Niu, Fei

He, Wen

Liu, Baiyun

TI Dexamethasone impairs neurofunctional recovery in rats following

traumatic brain injury by reducing circulating endothelial progenitor

cells and angiogenesis

SO BRAIN RESEARCH

LA English

DT Article

DE Traumatic brain injury; Dexamethasone; Endothelial progenitor cells;

Angiogenesis

ID CEREBRAL-ISCHEMIA; BLOOD-FLOW; NEUROGENESIS; DESTRUCTION; EXPRESSION;

CROSSTALK; IMPROVES; TISSUE

AB The administration of glucocorticoids (GCs) after traumatic brain injury (TBI) is controversial. Clinical evidence reveals the deleterious effects of GCs, but the mechanism remains unclear. Previous studies indicate that GCs impair wound healing by affecting endothelial progenitor cell (EPC) function and inhibiting angiogenesis after skin injury. Thus, we hypothesize that the central deleterious effect of GCs is associated with reduced EPCs and angiogenesis after TBI. Using a controlled cortical impact model, we examined the dynamic changes in circulating EPCs and in the regional microcirculation within 14 days of TBI by flow cytometry analysis and contrast-enhanced ultrasound, respectively. The modified neurological severity score (mNSS) and Morris water maze assay were used to assess neurological recovery. Angiogenesis and hippocampal neuron counts were assessed using immunohistochemistry analysis and hematoxylin and eosin staining 14 days after TBI. Compared with the TBI control group, dexamethasone treatment significantly reduced the number of circulating EPCs on days 1, 3, 7 and 14 (P < 0.05); decreased the number of CD31 + cells, the peak intensity and the number of hippocampal neurons on day 14 (P < 0.05); increased the latency on days 12 and 13 (P < 0.05); and reduced the percentage of time spent in the goal quadrant (P < 0.05) on day 14. Similarly, dexamethasone increased the mNSS on days 7 and 14 (P < 0.05). A strong correlation was observed between these results at 14 days after TBI (r = 0.815-0.892, P < 0.05). These data indicate that DEX inhibits the mobilization of EPC levels and angiogenesis around the lesion after TBI, which may contribute to neuronal cell loss and impaired neurofunction.

C1 [Xu, Xiaojian; Niu, Fei; Liu, Baiyun] Capital Med Univ, Beijing Neurosurg Inst, Beijing Key Lab Cent Nervous Syst Injury, Beijing, Peoples R China.

[Zhang, Bin; Hao, Shuyu; Liu, Baiyun] Capital Med Univ, Beijing Tian Tan Hosp, Dept Neurosurg, Beijing, Peoples R China.

[Liu, Baiyun] Beijing Inst Brain Disorders, Nerve Injury & Repair Ctr, Beijing, Peoples R China.

[Liu, Baiyun] China Natl Clin Res Ctr Neurol Dis, Beijing, Peoples R China.

[Zhu, Xueli; He, Wen] Capital Med Univ, Beijing Tian Tan Hosp, Dept Ultrasound, Beijing, Peoples R China.

[Wang, Liang] Tianjin Fifth Ctr Hosp, Dept Neurosurg, Tianjin, Peoples R China.

C3 Capital Medical University; Capital Medical University; Capital Medical

University

RP Liu, BY (通讯作者)，Capital Med Univ, Beijing Neurosurg Inst, Dept Neurosurg, 119 Nan Si Huan W Rd, Beijing 100070, Peoples R China.

EM liubaiyun1212@163.com

FU National Natural Science Foundation of China [81771327]

FX This work was funded by a research grant from the National Natural

Science Foundation of China (no. 81771327).

CR Asahara T, 1997, SCIENCE, V275, P964, DOI 10.1126/science.275.5302.964

Badaut J, 2014, TRANSL STROKE RES, V5, P394, DOI 10.1007/s12975-013-0304-z

Burlacu A, 2013, STEM CELLS DEV, V22, P643, DOI 10.1089/scd.2012.0273

Cain DW, 2017, NAT REV IMMUNOL, V17, P233, DOI 10.1038/nri.2017.1

Carenza E, 2014, NANOMED-NANOTECHNOL, V10, P225, DOI 10.1016/j.nano.2013.06.005

Carolina E, 2018, FRONT MED-LAUSANNE, V5, DOI 10.3389/fmed.2018.00276

Chen C, 2013, JOINT BONE SPINE, V80, P70, DOI 10.1016/j.jbspin.2012.02.015

Chen JL, 2001, STROKE, V32, P1005, DOI 10.1161/01.STR.32.4.1005

Edwards P, 2005, LANCET, V365, P1957

Eyding J, 2006, J CEREBR BLOOD F MET, V26, P576, DOI 10.1038/sj.jcbfm.9600216

FRENCH L A, 1964, Clin Neurosurg, V10, P212

Grisar J, 2007, ANN RHEUM DIS, V66, P1284, DOI 10.1136/ard.2006.066605

HALL ED, 1992, J NEUROSURG, V76, P13, DOI 10.3171/jns.1992.76.1.0013

Hayward NMEA, 2010, J NEUROTRAUM, V27, P2203, DOI 10.1089/neu.2010.1448

Hayward NMEA, 2011, J CEREBR BLOOD F MET, V31, P166, DOI 10.1038/jcbfm.2010.67

Kato T, 2017, BIOCHEM BIOPH RES CO, V493, P1010, DOI 10.1016/j.bbrc.2017.09.100

Kenney K, 2016, EXP NEUROL, V275, P353, DOI 10.1016/j.expneurol.2015.05.019

Kern R, 2004, STROKE, V35, P1665, DOI 10.1161/01.STR.0000129332.10721.7e

Krogias C, 2005, ULTRASOUND MED BIOL, V31, P1007, DOI 10.1016/j.ultrasmedbio.2005.01.001

Li ZY, 2012, J NEUROTRAUM, V29, P343, DOI 10.1089/neu.2011.1807

Liu L, 2007, J NEUROTRAUM, V24, P936, DOI 10.1089/neu.2006.0250

Liu Y, 2019, BRAIN RES, V1708, P160, DOI 10.1016/j.brainres.2018.12.023

Long JA, 2015, J CEREBR BLOOD F MET, V35, P1852, DOI 10.1038/jcbfm.2015.143

Ma FF, 2015, BRAIN RES, V1623, P150, DOI 10.1016/j.brainres.2015.02.010

Miyamoto N, 2014, CELL MOL LIFE SCI, V71, P1055, DOI 10.1007/s00018-013-1488-9

Nauck M, 1998, EUR J PHARMACOL, V341, P309, DOI 10.1016/S0014-2999(97)01464-7

Ohab JJ, 2006, J NEUROSCI, V26, P13007, DOI 10.1523/JNEUROSCI.4323-06.2006

Pang QY, 2017, BRAIN RES, V1663, P9, DOI 10.1016/j.brainres.2017.03.012

Salehi A, 2018, J CEREBR BLOOD F MET, V38, P274, DOI 10.1177/0271678X17744124

Seidel G, 2004, STROKE, V35, P1107, DOI 10.1161/01.STR.0000124125.19773.40

Shlosberg D, 2010, NAT REV NEUROL, V6, P393, DOI 10.1038/nrneurol.2010.74

Taguchi A, 2004, CIRCULATION, V109, P2972, DOI 10.1161/01.CIR.0000133311.25587.DE

Taylor AN, 2010, J NEUROTRAUM, V27, P1081, DOI 10.1089/neu.2009.1252

Vissapragada R, 2014, BRAIN RES, V1565, P8, DOI 10.1016/j.brainres.2014.03.018

Vorhees CV, 2006, NAT PROTOC, V1, P848, DOI 10.1038/nprot.2006.116

Wang B, 2012, J NEUROL SCI, V319, P117, DOI 10.1016/j.jns.2012.04.015

Wang L, 2015, TRANSL STROKE RES, V6, P50, DOI 10.1007/s12975-014-0362-x

Wei K, 1998, CIRCULATION, V97, P473

Wilson L, 2017, LANCET NEUROL, V16, P813, DOI 10.1016/S1474-4422(17)30279-X

Xiong Y, 2011, TRANSL STROKE RES, V2, P619, DOI 10.1007/s12975-011-0120-2

Yip HK, 2011, CRIT CARE, V15, DOI 10.1186/cc10002

NR 41

TC 7

Z9 8

U1 1

U2 11

PU ELSEVIER

PI AMSTERDAM

PA RADARWEG 29, 1043 NX AMSTERDAM, NETHERLANDS

SN 0006-8993

EI 1872-6240

J9 BRAIN RES

JI Brain Res.

PD DEC 15

PY 2019

VL 1725

AR 146469

DI 10.1016/j.brainres.2019.146469

PG 9

WC Neurosciences

WE Science Citation Index Expanded (SCI-EXPANDED)

SC Neurosciences & Neurology

GA JN9ZS

UT WOS:000497248100005

PM 31541641

DA 2023-06-10

ER

PT J

AU Chen, ZY

Tortella, FC

Dave, JR

Marshall, VS

Clarke, DL

Sing, G

Du, F

Lu, XCM

AF Chen, Zhiyong

Tortella, Frank C.

Dave, Jitendra R.

Marshall, Vivienne S.

Clarke, Diana L.

Sing, George

Du, Fu

Lu, X. -C. May

TI Human Amnion-Derived Multipotent Progenitor Cell Treatment Alleviates

Traumatic Brain Injury-Induced Axonal Degeneration

SO JOURNAL OF NEUROTRAUMA

LA English

DT Article

DE axon degeneration; human amnion; penetrating ballistic-like brain

injury; progenitor cells; traumatic brain injury

ID CENTRAL-NERVOUS-SYSTEM; MARROW STROMAL CELLS; EMBRYONIC STEM-CELLS;

EPITHELIAL-CELLS; RAT MODEL; PRECURSOR CELLS; SPINAL-CORD;

TRANSPLANTATION; NEURONS; NEUROGENESIS

AB To identify a viable cell source with potential neuroprotective effects, we studied amnion-derived multipotent progenitor (AMP) cells in a rat model of penetrating ballistic-like brain injury (PBBI). AMP cells were labeled with fluorescent dye PKH26 and injected in rats immediately following right hemispheric PBBI or sham PBBI surgery by ipsilateral i.c.v. administration. At 2 weeks post-injury, severe necrosis developed along the PBBI tract and axonal degeneration was prominent along the corpus callosum (cc) and in the ipsilateral thalamus. Injected AMP cells first entered the subventricular zone (SVZ) in both sham and PBBI rats. Further AMP cell migration along the cc only occurred in PBBI animals. No significant difference in injury volume was observed across all treatment groups. In contrast, treatment with AMP cells significantly attenuated axonal degeneration in both the thalamus and the cc. Interestingly, PKH26-labeled AMP cells were detected only in the SVZ and the cc (in parallel with the axonal degeneration), but not in the thalamus. None of the labeled AMP cells appeared to express neural differentiation, as evidenced by the lack of double labeling with nestin, S-100, GFAP, and MAP-2 immunostaining. In conclusion, AMP cell migration was specifically induced by PBBI and requires SVZ homing, yet the neuroprotective effect of intracerebral ventrical treatment using AMP cells was not limited to the area where the cells were present. This suggests that the attenuation of the secondary brain injury following PBBI was likely to be mediated by mechanisms other than cell replacement, possibly through delivery or sustained secretion of neurotrophic factors.

C1 [Chen, Zhiyong; Tortella, Frank C.; Dave, Jitendra R.; Lu, X. -C. May] Walter Reed Army Inst Res, Dept Appl Neurobiol, Div Psychiat & Neurosci, Silver Spring, MD 20910 USA.

[Marshall, Vivienne S.; Clarke, Diana L.; Sing, George] Stemnion Inc, Pittsburgh, PA USA.

[Du, Fu] FD NeuroTechnol Inc, Ellicott City, MD USA.

C3 United States Department of Defense; United States Army; Walter Reed

Army Institute of Research (WRAIR)

RP Chen, ZY (通讯作者)，Walter Reed Army Inst Res, Dept Appl Neurobiol, Div Psychiat & Neurosci, 503 Robert Grant Ave, Silver Spring, MD 20910 USA.

EM Zhiyong.chen@amedd.army.mil

RI Dave, Jitendra R/A-8940-2011

CR Bantubungi K, 2008, MOL CELL NEUROSCI, V37, P454, DOI 10.1016/j.mcn.2007.11.001

Bell RS, 2009, J TRAUMA, V66, pS104, DOI 10.1097/TA.0b013e31819d88c8

Ben Menachem-Zidon O, 2008, NEUROPSYCHOPHARMACOL, V33, P2251, DOI 10.1038/sj.npp.1301606

Brustle O, 1998, NAT BIOTECHNOL, V16, P1040, DOI 10.1038/3481

Cao QL, 2005, J NEUROSCI, V25, P6947, DOI 10.1523/JNEUROSCI.1065-05.2005

*CDC, 1999, TRAUM BRAIN INJ US R

DUNNETT SB, 1994, J NEUROL, V242, pS43, DOI 10.1007/BF00939242

Harting MT, 2008, NEUROSURG FOCUS, V24, DOI 10.3171/FOC/2008/24/3-4/E17

Hentze H, 2007, TRENDS BIOTECHNOL, V25, P24, DOI 10.1016/j.tibtech.2006.10.010

HernitGrant CS, 1996, EXP NEUROL, V139, P131, DOI 10.1006/exnr.1996.0088

Hokari M, 2008, J NEUROSCI RES, V86, P1024, DOI 10.1002/jnr.21572

Janardhan Vallabh, 2004, Curr Cardiol Rep, V6, P117, DOI 10.1007/s11886-004-0009-8

Kakishita K, 2000, EXP NEUROL, V165, P27, DOI 10.1006/exnr.2000.7449

Kakishita K, 2003, BRAIN RES, V980, P48, DOI 10.1016/S0006-8993(03)02875-0

Kerr DA, 2003, J NEUROSCI, V23, P5131

Kong XY, 2008, BRAIN RES, V1205, P108, DOI 10.1016/j.brainres.2008.02.040

Lee SH, 2000, NAT BIOTECHNOL, V18, P675, DOI 10.1038/76536

Lie DC, 2004, ANNU REV PHARMACOL, V44, P399, DOI 10.1146/annurev.pharmtox.44.101802.121631

Lindvall O, 2004, STROKE, V35, P2691, DOI 10.1161/01.STR.0000143323.84008.f4

Lu DY, 2001, J NEUROTRAUM, V18, P813, DOI 10.1089/089771501316919175

Mahmood U, 2004, RADIOLOGY, V233, P625, DOI 10.1148/radiol.2333041190

McDonald JW, 2004, J NEUROTRAUM, V21, P383, DOI 10.1089/089771504323004539

Mezey E, 2003, P NATL ACAD SCI USA, V100, P1364, DOI 10.1073/pnas.0336479100

Pierret C, 2007, STEM CELLS DEV, V16, P1017, DOI 10.1089/scd.2007.0012

Richardson RM, 2007, NEUROSURG CLIN N AM, V18, P169, DOI 10.1016/j.nec.2006.10.007

Sakuragawa N, 1996, NEUROSCI LETT, V209, P9, DOI 10.1016/0304-3940(96)12599-4

Schouten JW, 2004, J NEUROTRAUM, V21, P1501, DOI 10.1089/0897715042441774

Schuldiner M, 2001, BRAIN RES, V913, P201, DOI 10.1016/S0006-8993(01)02776-7

Snyder E Y, 1997, Adv Neurol, V72, P121

SOTELO C, 1991, TRENDS NEUROSCI, V14, P350, DOI 10.1016/0166-2236(91)90161-M

Steed David L, 2008, Eplasty, V8, pe18

SWANSON LW, 1980, NEUROENDOCRINOLOGY, V31, P410, DOI 10.1159/000123111

TAKAHASHI K, 1970, Development Growth and Differentiation, V12, P65

Uchida S, 2000, J NEUROSCI RES, V62, P585, DOI 10.1002/1097-4547(20001115)62:4<585::AID-JNR13>3.0.CO;2-U

van Praag H, 2002, NATURE, V415, P1030, DOI 10.1038/4151030a

Weimann JM, 2003, P NATL ACAD SCI USA, V100, P2088, DOI 10.1073/pnas.0337659100

Williams AJ, 2005, J NEUROTRAUM, V22, P313, DOI 10.1089/neu.2005.22.313

Zhao CM, 2008, CELL, V132, P645, DOI 10.1016/j.cell.2008.01.033

NR 38

TC 28

Z9 31

U1 0

U2 1

PU MARY ANN LIEBERT, INC

PI NEW ROCHELLE

PA 140 HUGUENOT STREET, 3RD FL, NEW ROCHELLE, NY 10801 USA

SN 0897-7151

EI 1557-9042

J9 J NEUROTRAUM

JI J. Neurotrauma

PD NOV

PY 2009

VL 26

IS 11

BP 1987

EP 1997

DI 10.1089/neu.2008.0863

PG 11

WC Critical Care Medicine; Clinical Neurology; Neurosciences

WE Science Citation Index Expanded (SCI-EXPANDED)

SC General & Internal Medicine; Neurosciences & Neurology

GA 523CO

UT WOS:000272049600014

PM 19886807

DA 2023-06-10

ER

PT J

AU Maegele, M

Schafer, U

AF Maegele, Marc

Schaefer, Ute

TI Stem cell-based cellular replacement strategies following traumatic

brain injury (TBI)

SO MINIMALLY INVASIVE THERAPY & ALLIED TECHNOLOGIES

LA English

DT Review

DE traumatic brain injury; cell replacement therapy; stem cells

ID MARROW STROMAL CELLS; CENTRAL-NERVOUS-SYSTEM; NEURAL STEM/PROGENITOR

CELLS; ADULT-RAT STRIATUM; BONE-MARROW; PROGENITOR CELLS; EXPERIMENTAL

STROKE; MAMMALIAN BRAIN; NEURONAL CELLS; GENE-THERAPY

AB Given the limited capacity of the central nervous system for self-repair, the use of stem cells holds an enormous potential in cell replacement therapy following traumatic brain injury and has thus received a great deal of scientific and public interest in recent years. During the past decade, several stem/progenitor cell types and lines from various sources such as embryonic rodent and human stem cells, immortalized progenitor cells, bone marrow derived cells or even post-mitotic neurons derived from human teratocarcinoma cells have been assessed for their potential to improve neurofunctional and behavioural outcome after transplantation into the experimentally injured brain. A number of studies indicate that cells engrafted into the injured brain can survive and, at least in part, may reverse behavioural dysfunction and histomorphological damage. Although these results emphasized their potential therapeutic role in traumatic brain injury, the detailed mechansim on how stem cells generate their mode of action, e.g. via integration into surviving neuronal circuits, local trophic support, or modification of the local mircoenvironment to enhance endogenous regeneration and potection remain yet to be identified. A review on current pre-clinical knowledge with respect to cellular replacement into the experimentally injured brain is presented.

C1 [Maegele, Marc; Schaefer, Ute] Univ Witten Herdecke, Inst Res Operat Med, Cologne Merheim Med Ctr, D-51109 Cologne, Germany.

[Maegele, Marc] Dept Trauma & Orthoped Surg, Intens Care Unit, Cologne, Germany.

C3 Witten Herdecke University

RP Schafer, U (通讯作者)，Univ Witten Herdecke, Inst Res Operat Med, Cologne Merheim Med Ctr, Ostmerheimerstr 200, D-51109 Cologne, Germany.

EM ute.schaefer@uni-wh.de

CR ALTMAN J, 1967, NATURE, V214, P1098, DOI 10.1038/2141098a0

Arnhold S, 2000, J NEUROSURG, V93, P1026, DOI 10.3171/jns.2000.93.6.1026

Bakshi A, 2005, BRAIN RES, V1065, P8, DOI 10.1016/j.brainres.2005.09.059

Bentz K, 2007, J NEUROSCI RES, V85, P1057, DOI 10.1002/jnr.21219

Bjorklund A, 2000, NAT NEUROSCI, V3, P537, DOI 10.1038/75705

Boockvar JA, 2005, NEUROSURGERY, V56, P163, DOI 10.1227/01.NEU.0000145866.25433.FF

Bruns TJ, 2003, EPILEPSIA, V44, P2, DOI 10.1046/j.1528-1157.44.s10.3.x

Chen XG, 2002, J NEUROSCI RES, V69, P687, DOI 10.1002/jnr.10334

Dezawa M, 2004, J CLIN INVEST, V113, P1701, DOI 10.1172/JCI200420935

Dunnett SB, 2001, NAT REV NEUROSCI, V2, P365, DOI 10.1038/35072572

Emsley JG, 2005, PROG NEUROBIOL, V75, P321, DOI 10.1016/j.pneurobio.2005.04.002

Englund U, 2002, DEV BRAIN RES, V134, P123, DOI 10.1016/S0165-3806(01)00330-3

Erdo F, 2003, J CEREBR BLOOD F MET, V23, P780, DOI 10.1097/01.WCB.0000071886.63724.FB

Erdo Franciska, 2004, Orvosi Hetilap, V145, P1307

Gage FH, 2000, SCIENCE, V287, P1433, DOI 10.1126/science.287.5457.1433

Ghajar J, 2000, LANCET, V356, P923, DOI 10.1016/S0140-6736(00)02689-1

GIULIAN D, 1989, J NEUROSCI, V9, P4416

GOLDMAN SA, 1983, P NATL ACAD SCI-BIOL, V80, P2390, DOI 10.1073/pnas.80.8.2390

Hagan M, 2003, NEUROSCI LETT, V351, P149, DOI 10.1016/j.neulet.2003.07.021

Harrahill M, 1997, J Emerg Nurs, V23, P282, DOI 10.1016/S0099-1767(97)90032-5

Hoane MR, 2004, J NEUROTRAUM, V21, P163, DOI 10.1089/089771504322778622

Hoehn M, 2002, P NATL ACAD SCI USA, V99, P16267, DOI 10.1073/pnas.242435499

HOEHN M, 2007, J PHYSL, V594, P25

Horner PJ, 2000, NATURE, V407, P963, DOI 10.1038/35039559

Jennett B, 1972, Clin Neurosurg, V19, P200

Keeling KL, 2000, J NEUROIMMUNOL, V105, P20, DOI 10.1016/S0165-5728(00)00183-1

Kondziolka D, 2000, NEUROLOGY, V55, P565, DOI 10.1212/WNL.55.4.565

Le Belle JE, 2002, BIODRUGS, V16, P389, DOI 10.2165/00063030-200216060-00001

Lenzlinger PM, 2002, J NEUROIMMUNOL, V122, P167, DOI 10.1016/S0165-5728(01)00466-0

LINDVALL O, 1991, TRENDS NEUROSCI, V14, P376, DOI 10.1016/0166-2236(91)90167-S

Longhi L, 2004, J NEUROTRAUM, V21, P1723, DOI 10.1089/0897715042664876

Lu D, 2001, NEUROREPORT, V12, P559, DOI 10.1097/00001756-200103050-00025

Lu DY, 2001, J NEUROTRAUM, V18, P813, DOI 10.1089/089771501316919175

Lundberg C, 1996, BRAIN RES, V737, P295, DOI 10.1016/0006-8993(96)00923-7

Maegele M, 2007, EUR SURG RES, V39, P372, DOI 10.1159/000107097

Mahmood A, 2005, NEUROSURGERY, V57, P1026, DOI 10.1227/01.NEU.0000181369.76323.50

Mahmood A, 2004, J NEUROTRAUM, V21, P33, DOI 10.1089/089771504772695922

Mahmood A, 2001, NEUROSURGERY, V49, P1196, DOI 10.1097/00006123-200111000-00031

Mahmood A, 2001, J NEUROSURG, V94, P589, DOI 10.3171/jns.2001.94.4.0589

MAHMOOD A, 2001, NEUROSURGERY, V39, P203

Mahmood A, 2007, NEUROSURGERY, V60, P546, DOI 10.1227/01.NEU.0000255346.25959.99

McIntosh TK, 1998, J NEUROTRAUM, V15, P731, DOI 10.1089/neu.1998.15.731

McKay R, 1997, SCIENCE, V276, P66, DOI 10.1126/science.276.5309.66

Molcanyi M, 2007, J NEUROTRAUM, V24, P625, DOI 10.1089/neu.2006.0180

Murray CJL, 1997, LANCET, V349, P1436, DOI 10.1016/S0140-6736(96)07495-8

Okano H, 2002, J NEUROSCI RES, V69, P698, DOI 10.1002/jnr.10343

PERSSON L, 1976, VIRCHOWS ARCH B, V22, P21

Philips MF, 2001, J NEUROSURG, V94, P765, DOI 10.3171/jns.2001.94.5.0765

Prestoz L, 2001, MOL CELL NEUROSCI, V18, P473, DOI 10.1006/mcne.2001.1037

RENFRANZ PJ, 1991, CELL, V66, P713, DOI 10.1016/0092-8674(91)90116-G

Riess P, 2002, NEUROSURGERY, V51, P1043, DOI 10.1097/00006123-200210000-00035

Riess P, 2007, J NEUROTRAUM, V24, P216, DOI 10.1089/neu.2006.0141

Royo NC, 2003, CURR OPIN PHARMACOL, V3, P27, DOI 10.1016/S1471-4892(02)00006-1

RYDER EF, 1990, J NEUROBIOL, V21, P356, DOI 10.1002/neu.480210209

Sanchez-Ramos JR, 2002, J NEUROSCI RES, V69, P880, DOI 10.1002/jnr.10337

Schouten JW, 2004, J NEUROTRAUM, V21, P1501, DOI 10.1089/0897715042441774

Seaberg RM, 2003, TRENDS NEUROSCI, V26, P125, DOI 10.1016/S0166-2236(03)00031-6

Shear DA, 2004, BRAIN RES, V1026, P11, DOI 10.1016/j.brainres.2004.07.087

Shindo T, 2006, J MED INVESTIG, V53, P42, DOI 10.2152/jmi.53.42

Sinden J. D, 2000, NOVART FDN SYMP, V231

Sinden J. D, 2000, NOVARITS FDN S, V231

Sinden JD, 2000, NOVART FDN SYMP, V231, P270, DOI 10.1002/0470870834.ch16

Sinden JD, 1997, NEUROSCIENCE, V81, P599, DOI 10.1016/S0306-4522(97)00330-8

Smith DH, 1997, J NEUROTRAUM, V14, P715, DOI 10.1089/neu.1997.14.715

SOARES H, 1991, Journal of Neural Transplantation and Plasticity, V2, P207

Soares HD, 1995, J NEUROSCI, V15, P8223

Sortwell CE, 2003, FRONT BIOSCI, V8, pS522, DOI 10.2741/1096

Sosin DM, 1996, PEDIATRICS, V98, P868

Tate MC, 2002, CELL TRANSPLANT, V11, P283

Thompson HJ, 2005, J CEREBR BLOOD F MET, V25, P163, DOI 10.1038/sj.jcbfm.9600008

Thomson JA, 1998, SCIENCE, V282, P1145, DOI 10.1126/science.282.5391.1145

Trojanowski JQ, 1997, EXP NEUROL, V144, P92, DOI 10.1006/exnr.1996.6393

Vescovi AL, 1999, J NEUROTRAUM, V16, P689, DOI 10.1089/neu.1999.16.689

Wennersten A, 2004, J NEUROSURG, V100, P88, DOI 10.3171/jns.2004.100.1.0088

Wennersten A, 2006, EXP NEUROL, V199, P339, DOI 10.1016/j.expneurol.2005.12.035

Wong AM, 2005, BRAIN RES, V1063, P140, DOI 10.1016/j.brainres.2005.09.049

Zhang C, 2005, J NEUROTRAUM, V22, P1456, DOI 10.1089/neu.2005.22.1456

NR 77

TC 23

Z9 23

U1 0

U2 9

PU TAYLOR & FRANCIS LTD

PI ABINGDON

PA 2-4 PARK SQUARE, MILTON PARK, ABINGDON OR14 4RN, OXON, ENGLAND

SN 1364-5706

EI 1365-2931

J9 MINIM INVASIV THER

JI Minim. Invasive Ther. Allied Technol.

PY 2008

VL 17

IS 2

BP 119

EP 131

DI 10.1080/13645700801970087

PG 13

WC Surgery

WE Science Citation Index Expanded (SCI-EXPANDED)

SC Surgery

GA 306GC

UT WOS:000256235300006

PM 18465446

DA 2023-06-10

ER

PT J

AU Flygt, J

Gumucio, A

Ingelsson, M

Skoglund, K

Holm, J

Alafuzoff, I

Marklund, N

AF Flygt, Johanna

Gumucio, Astrid

Ingelsson, Martin

Skoglund, Karin

Holm, Jonatan

Alafuzoff, Irina

Marklund, Niklas

TI Human Traumatic Brain Injury Results in Oligodendrocyte Death and

Increases the Number of Oligodendrocyte Progenitor Cells

SO JOURNAL OF NEUROPATHOLOGY AND EXPERIMENTAL NEUROLOGY

LA English

DT Article

DE Apoptosis; Human; Immunohistochemistry; Oligodendrocyte; Oligodendrocyte

progenitor cells; Traumatic brain injury

ID DIFFUSE AXONAL INJURY; CENTRAL-NERVOUS-SYSTEM; RAT SPINAL-CORD;

MULTIPLE-SCLEROSIS LESIONS; SITU DNA FRAGMENTATION;

FACTOR-ALPHA-RECEPTOR; FATAL HEAD-INJURY; WHITE-MATTER; ADULT CNS;

INCREASED EXPRESSION

AB Oligodendrocyte (OL) death may contribute to white matter pathology, a common cause of network dysfunction and persistent cognitive problems in patients with traumatic brain injury (TBI). Oligodendrocyte progenitor cells (OPCs) persist throughout the adult CNS and may replace dead OLs. OL death and OPCs were analyzed by immunohistochemistry of human brain tissue samples, surgically removed due to life-threatening contusions and/or focal brain swelling at 60.6 +/- 75 hours (range 4-192 hours) postinjury in 10 severe TBI patients (age 51.7 +/- 18.5 years). Control brain tissue was obtained postmortem from 5 age-matched patients without CNS disorders. TUNEL and CC1 co-labeling was used to analyze apoptotic OLs, which were increased in injured brain tissue (p < 0.05), without correlation with time from injury until surgery. The OPC markers Olig2, A2B5, NG2, and PDGFR-alpha were used. In contrast to the number of single-labeled Olig2, A2B5, NG2, and PDGFR-alpha-positive cells, numbers of Olig2 and A2B5 co-labeled cells were increased in TBI samples (p < 0.05); this was inversely correlated with time from injury to surgery (r = -0.8, p < 0.05). These results indicate that severe focal human TBI results in OL death and increases in OPCs postinjury, which may influence white matter function following TBI.

C1 [Flygt, Johanna; Skoglund, Karin; Holm, Jonatan; Marklund, Niklas] Uppsala Univ, Dept Neurosci, Neurosurg, Uppsala, Sweden.

[Gumucio, Astrid; Ingelsson, Martin] Uppsala Univ, Dept Publ Hlth & Caring Sci, Geriatr, Uppsala, Sweden.

[Alafuzoff, Irina] Uppsala Univ, Dept Immunol Genet & Pathol, Uppsala, Sweden.

C3 Uppsala University; Uppsala University; Uppsala University

RP Marklund, N (通讯作者)，Univ Uppsala Hosp, Dept Neurosurg, Ing 85,2 Tr, SE-75185 Uppsala, Sweden.

EM Niklas.Marklund@neuro.uu.se

OI Marklund, Niklas/0000-0002-9797-5626

CR Abrous DN, 2005, PHYSIOL REV, V85, P523, DOI 10.1152/physrev.00055.2003

ADAMS JH, 1989, HISTOPATHOLOGY, V15, P49, DOI 10.1111/j.1365-2559.1989.tb03040.x

Algattas H, 2014, INT J MOL SCI, V15, P309, DOI 10.3390/ijms15010309

Baracskay KL, 2007, GLIA, V55, P1001, DOI 10.1002/glia.20519

Baumann N, 2001, PHYSIOL REV, V81, P871, DOI 10.1152/physrev.2001.81.2.871

Beschorner R, 2000, ACTA NEUROPATHOL, V100, P627, DOI 10.1007/s004010000232

Boulanger JJ, 2014, NEUROSCIENCE, V269, P343, DOI 10.1016/j.neuroscience.2014.03.063

Bradl M, 2010, ACTA NEUROPATHOL, V119, P37, DOI 10.1007/s00401-009-0601-5

Bramlett HM, 2002, ACTA NEUROPATHOL, V103, P607, DOI 10.1007/s00401-001-0510-8

Browne KD, 2011, J NEUROTRAUM, V28, P1747, DOI 10.1089/neu.2011.1913

Carroll WM, 1998, BRAIN, V121, P293, DOI 10.1093/brain/121.2.293

Chari DM, 2007, INT REV NEUROBIOL, V79, P589, DOI 10.1016/S0074-7742(07)79026-8

Chen S, 2003, EXP NEUROL, V182, P87, DOI 10.1016/S0014-4886(03)00002-5

Clark RSB, 1999, FASEB J, V13, P813, DOI 10.1096/fasebj.13.8.813

Clarke LE, 2012, J NEUROSCI, V32, P8173, DOI 10.1523/JNEUROSCI.0928-12.2012

Cole TB, 2004, JAMA-J AM MED ASSOC, V291, P2531, DOI 10.1001/jama.291.21.2531

Conti AC, 1998, J NEUROSCI, V18, P5663

Corral L, 2007, BRAIN INJURY, V21, P1225, DOI 10.1080/02699050701727460

Crowe MJ, 1997, NAT MED, V3, P73, DOI 10.1038/nm0197-73

Cudrici C, 2006, J REHABIL RES DEV, V43, P123, DOI 10.1682/JRRD.2004.08.0111

Cui QL, 2013, AM J PATHOL, V183, P516, DOI 10.1016/j.ajpath.2013.04.016

Czepiel M, 2015, GLIA, V63, P513, DOI 10.1002/glia.22769

Dawson MRL, 2000, J NEUROSCI RES, V61, P471, DOI 10.1002/1097-4547(20000901)61:5<471::AID-JNR1>3.3.CO;2-E

Dent KA, 2015, PLOS ONE, V10, DOI 10.1371/journal.pone.0121541

Dewar D, 2003, J CEREBR BLOOD F MET, V23, P263, DOI 10.1097/01.WCB.0000053472.41007.F9

Di Bello IC, 1999, J NEUROCYTOL, V28, P365, DOI 10.1023/A:1007069815302

Dore-Duffy P, 2006, J CEREBR BLOOD F MET, V26, P613, DOI 10.1038/sj.jcbfm.9600272

Draper K, 2008, NEUROPSYCHOLOGY, V22, P618, DOI 10.1037/0894-4105.22.5.618

Elf K, 2002, CRIT CARE MED, V30, P2129, DOI 10.1097/00003246-200209000-00029

Fancy SPJ, 2004, MOL CELL NEUROSCI, V27, P247, DOI 10.1016/j.mcn.2004.06.015

Fancy SPJ, 2011, ANNU REV NEUROSCI, V34, P21, DOI 10.1146/annurev-neuro-061010-113629

Flygt J, 2013, EUR J NEUROSCI, V38, P2153, DOI 10.1111/ejn.12179

Franklin RJM, 2002, NAT REV NEUROSCI, V3, P705, DOI 10.1038/nrn917

Geha S, 2010, BRAIN PATHOL, V20, P399, DOI 10.1111/j.1750-3639.2009.00295.x

Gennarelli TA, 1998, NEUROSCIENTIST, V4, P202, DOI 10.1177/107385849800400316

Gensert JM, 1997, NEURON, V19, P197, DOI 10.1016/S0896-6273(00)80359-1

GROVES AK, 1993, NATURE, V362, P453, DOI 10.1038/362453a0

Holmin S, 1998, NEUROSURGERY, V42, P291, DOI 10.1097/00006123-199802000-00047

Horner PJ, 2000, J NEUROSCI, V20, P2218

Jackson EL, 2006, NEURON, V51, P187, DOI 10.1016/j.neuron.2006.06.012

Jadasz JJ, 2012, CELL TISSUE RES, V349, P331, DOI 10.1007/s00441-012-1331-x

Johnson VE, 2013, BRAIN, V136, P28, DOI 10.1093/brain/aws322

Johnson VE, 2013, EXP NEUROL, V246, P35, DOI 10.1016/j.expneurol.2012.01.013

Johnstone JT, 2013, PLOS ONE, V8, DOI 10.1371/journal.pone.0080975

Keirstead HS, 1998, GLIA, V22, P161, DOI 10.1002/(SICI)1098-1136(199802)22:2<161::AID-GLIA7>3.0.CO;2-A

Kelley BJ, 2007, J NEUROPATH EXP NEUR, V66, P989, DOI 10.1097/NEN.0b013e3181588245

Kinnunen KM, 2011, BRAIN, V134, P449, DOI 10.1093/brain/awq347

LEVINE JM, 1994, J NEUROSCI, V14, P4716

Li GL, 1996, J NEUROPATH EXP NEUR, V55, P280, DOI 10.1097/00005072-199603000-00003

Lojewski X, 2014, STEM CELL TRANSL MED, V3, P458, DOI 10.5966/sctm.2013-0117

Lotocki G, 2011, THER HYPOTHERMIA TEM, V1, DOI 10.1089/ther.2010.0011

Lotocki G, 2011, NEUROSCI LETT, V499, P143, DOI 10.1016/j.neulet.2011.05.056

LUDWIN SK, 1978, LAB INVEST, V39, P597

Maas AIR, 2008, LANCET NEUROL, V7, P728, DOI 10.1016/S1474-4422(08)70164-9

Maeda Y, 2001, ANN NEUROL, V49, P776, DOI 10.1002/ana.1015

MAJOCHA RE, 1989, J NEUROCHEM, V53, P953, DOI 10.1111/j.1471-4159.1989.tb11798.x

Maki T, 2013, FRONT CELL NEUROSCI, V7, DOI 10.3389/fncel.2013.00275

Masel BE, 2010, J NEUROTRAUM, V27, P1529, DOI 10.1089/neu.2010.1358

Mateer CA, 2006, NEUROREHABILITATION, V21, P315

McTigue DM, 2008, J NEUROCHEM, V107, P1, DOI 10.1111/j.1471-4159.2008.05570.x

McTigue DM, 2001, J NEUROSCI, V21, P3392, DOI 10.1523/JNEUROSCI.21-10-03392.2001

Merrill JE, 1999, NEUROPATH APPL NEURO, V25, P435

Mierzwa AJ, 2015, J NEUROPATH EXP NEUR, V74, P218, DOI 10.1097/NEN.0000000000000165

Miller BA, 2007, J NEUROINFLAMM, V4, DOI 10.1186/1742-2094-4-28

Moransard M, 2011, BRAIN, V134, P1315, DOI 10.1093/brain/awr070

Morelli PI, 2006, ATHEROSCLEROSIS, V184, P39, DOI 10.1016/j.atherosclerosis.2005.03.026

Oliver-De La Cruz J, 2014, PLOS ONE, V9, DOI 10.1371/journal.pone.0099253

Oumesmar BN, 1997, J NEUROSCI, V17, P125, DOI 10.1523/JNEUROSCI.17-01-00125.1997

Piaton G, 2010, J NEUROCHEM, V114, P1243, DOI 10.1111/j.1471-4159.2010.06831.x

PONSFORD JL, 1995, BRAIN INJURY, V9, P11, DOI 10.3109/02699059509004566

Raghupathi R, 2004, BRAIN PATHOL, V14, P215, DOI 10.1111/j.1750-3639.2004.tb00056.x

Ramlackhansingh AF, 2011, ANN NEUROL, V70, P374, DOI 10.1002/ana.22455

Rhee W, 2009, GLIA, V57, P510, DOI 10.1002/glia.20780

Sahinkaya FR, 2014, EXP NEUROL, V255, P113, DOI 10.1016/j.expneurol.2014.02.025

Sanchez I, 1996, J NEUROSCI, V16, P5095

Scolding N, 1998, BRAIN, V121, P2221, DOI 10.1093/brain/121.12.2221

Sharp DJ, 2014, NAT REV NEUROL, V10, P156, DOI 10.1038/nrneurol.2014.15

Sharp DJ, 2011, BRAIN, V134, P2233, DOI 10.1093/brain/awr175

Shaw K, 2001, CLIN NEUROPATHOL, V20, P106

Shuman SL, 1997, J NEUROSCI RES, V50, P798, DOI 10.1002/(SICI)1097-4547(19971201)50:5<798::AID-JNR16>3.3.CO;2-#

Sidaros A, 2008, BRAIN, V131, P559, DOI 10.1093/brain/awm294

Simpson JE, 2007, NEUROPATH APPL NEURO, V33, P410, DOI 10.1111/j.1365-2990.2007.00828.x

Skoglund K, 2012, CRIT CARE MED, V40, P216, DOI 10.1097/CCM.0b013e31822d7dbd

Smith DH, 2003, J HEAD TRAUMA REHAB, V18, P307, DOI 10.1097/00001199-200307000-00003

Smith DH, 2013, J NEUROTRAUM, V30, P307, DOI 10.1089/neu.2012.2825

Smith FM, 2000, ACTA NEUROPATHOL, V100, P537, DOI 10.1007/s004010000222

Sullivan GM, 2013, J NEUROPATH EXP NEUR, V72, P1106, DOI 10.1097/NEN.0000000000000009

Sun F, 2010, GLIA, V58, P1304, DOI 10.1002/glia.21009

Takahashi C, 2013, CELL DEATH DIS, V4, DOI 10.1038/cddis.2013.335

Trotter J, 2010, BRAIN RES REV, V63, P72, DOI 10.1016/j.brainresrev.2009.12.006

Uschkureit T, 2000, J NEUROSCI, V20, P5225, DOI 10.1523/JNEUROSCI.20-14-05225.2000

VIGNAIS L, 1995, NEUROREPORT, V6, P1993, DOI 10.1097/00001756-199510010-00010

Wegner M, 2001, MICROSC RES TECHNIQ, V52, P746, DOI 10.1002/jemt.1059

Williams S, 2001, ACTA NEUROPATHOL, V102, P581, DOI 10.1007/s004010100410

Wilson HC, 2006, J NEUROIMMUNOL, V176, P162, DOI 10.1016/j.jneuroim.2006.04.014

Wilson S, 2004, J NEUROTRAUM, V21, P239, DOI 10.1089/089771504322972031

Windrem MS, 2004, NAT MED, V10, P93, DOI 10.1038/nm974

Yeung MSY, 2014, CELL, V159, P766, DOI 10.1016/j.cell.2014.10.011

Young KM, 2013, NEURON, V77, P873, DOI 10.1016/j.neuron.2013.01.006

NR 99

TC 33

Z9 34

U1 1

U2 6

PU OXFORD UNIV PRESS INC

PI CARY

PA JOURNALS DEPT, 2001 EVANS RD, CARY, NC 27513 USA

SN 0022-3069

EI 1554-6578

J9 J NEUROPATH EXP NEUR

JI J. Neuropathol. Exp. Neurol.

PD JUN

PY 2016

VL 75

IS 6

BP 503

EP 515

DI 10.1093/jnen/nlw025

PG 13

WC Clinical Neurology; Neurosciences; Pathology

WE Science Citation Index Expanded (SCI-EXPANDED)

SC Neurosciences & Neurology; Pathology

GA DO3FC

UT WOS:000377665000003

PM 27105664

OA Bronze

DA 2023-06-10

ER

PT J

AU Wang, ZG

Luo, Y

Chen, LA

Liang, W

AF Wang, Zhigang

Luo, Yong

Chen, Lvan

Liang, Wu

TI Safety of neural stem cell transplantation in patients with severe

traumatic brain injury

SO EXPERIMENTAL AND THERAPEUTIC MEDICINE

LA English

DT Article

DE cell transplantation; traumatic brain injury; stem cell

ID AMYOTROPHIC-LATERAL-SCLEROSIS; SPINAL-CORD-INJURY; NEUROTROPHIC FACTORS;

FUNCTIONAL RECOVERY; PRACTICAL SCALE; RATS; DIFFERENTIATE; EPIDEMIOLOGY;

SCAFFOLDS; NEURONS

AB Neural stem cell (NSC) therapy is a promising treatment for traumatic brain injury (TBI). In addition, mesenchymal stem cells (MSCs) have been investigated for the treatment of TBI due to their functions in neural regeneration and their neurotrophic effect. In the present study, the safety, feasibility and biological effects of autologous MSC-derived NSC-like cell transplantation were investigated in 10 patients with severe TBI. All patients received intravenous or intrathecal injections of human NSC-like cells and were evaluated with physical and neurological examinations, routine laboratory tests and neuroradiological findings. The results indicated that the majority of patients experienced improved neurological function in different degrees during the follow-up period. No mortality or serious adverse events were observed in any patient subsequent to transplantation. Higher serum levels of nerve growth factor and brain-derived neurotrophic factor were detected following the transplantation, as compared with the levels prior to treatment. Overall, the present results suggest that transplantation of autologous NSC-like cells is feasible and appears to be safe for the treatment of non-acute severe TBI.

C1 [Wang, Zhigang; Luo, Yong; Chen, Lvan; Liang, Wu] First Peoples Hosp Jingmen, Dept Neurosurg, 67 Xiangshan Rd, Jingmen 448000, Hubei, Peoples R China.

RP Liang, W (通讯作者)，First Peoples Hosp Jingmen, Dept Neurosurg, 67 Xiangshan Rd, Jingmen 448000, Hubei, Peoples R China.

EM 360234044@qq.com

FU Science and Technology Bureau of Jingmen [2012YD34]

FX The present study was supported by the Science and Technology Bureau of

Jingmen (grant no. 2012YD34).

CR Anbari F, 2014, NEURAL REGEN RES, V9, P919, DOI 10.4103/1673-5374.133133

Badri S, 2012, INTENS CARE MED, V38, P1800, DOI 10.1007/s00134-012-2655-4

Brain Trauma F, 2008, J NEUROTRAUM, V25, P276, DOI DOI 10.1089/NEU.2007.9989.ERRATUM

Caplan AI, 2007, J CELL PHYSIOL, V213, P341, DOI 10.1002/jcp.21200

Chen GJ, 2013, J TRANSL MED, V11, DOI 10.1186/1479-5876-11-21

Chu K, 2004, BRAIN RES, V1016, P145, DOI 10.1016/j.brainres.2004.04.038

Fujiwara Y, 2004, NEUROSCI LETT, V366, P287, DOI 10.1016/j.neulet.2004.05.080

Gao JL, 2006, EXP NEUROL, V201, P281, DOI 10.1016/j.expneurol.2006.04.039

Glass JD, 2012, STEM CELLS, V30, P1144, DOI 10.1002/stem.1079

Guan J, 2013, BIOMATERIALS, V34, P5937, DOI 10.1016/j.biomaterials.2013.04.047

Hage Ver, 2011, NURSE COM NURSING SP, V24, P44

Harting MT, 2008, NEUROSURG FOCUS, V24, DOI 10.3171/FOC/2008/24/3-4/E17

Harting MT, 2009, J SURG RES, V153, P188, DOI 10.1016/j.jss.2008.03.037

JENNETT B, 1975, LANCET, V1, P480

Karimi-Abdolrezaee S, 2006, J NEUROSCI, V26, P3377, DOI 10.1523/JNEUROSCI.4184-05.2006

Karussis D, 2010, ARCH NEUROL-CHICAGO, V67, P1187, DOI 10.1001/archneurol.2010.248

Langlois JA, 2005, J HEAD TRAUMA REHAB, V20, P187, DOI 10.1097/00001199-200505000-00001

Langlois JA, 2006, J HEAD TRAUMA REHAB, V21, P375, DOI 10.1097/00001199-200609000-00001

Lenzlinger PM, 2001, MOL NEUROBIOL, V24, P169

Lindvall O, 2006, NATURE, V441, P1094, DOI 10.1038/nature04960

Liu Y, 2014, MOL MED REP, V9, P333, DOI 10.3892/mmr.2013.1803

Llado J, 2004, MOL CELL NEUROSCI, V27, P322, DOI 10.1016/j.mcn.2004.07.010

Lu P, 2003, EXP NEUROL, V181, P115, DOI 10.1016/S0014-4886(03)00037-2

Ma HY, 2011, MOL MED REP, V4, P849, DOI 10.3892/mmr.2011.510

Ma K, 2011, NEUROL RES, V33, P1083, DOI 10.1179/1743132811Y.0000000053

Maas AIR, 2008, LANCET NEUROL, V7, P728, DOI 10.1016/S1474-4422(08)70164-9

Maegele M, 2008, MINIM INVASIV THER, V17, P119, DOI 10.1080/13645700801970087

Modo M, 2002, STROKE, V33, P2270, DOI 10.1161/01.STR.0000027693.50675.C5

Pluchino S, 2005, NATURE, V436, P266, DOI 10.1038/nature03889

Riess P, 2002, NEUROSURGERY, V51, P1043, DOI 10.1097/00006123-200210000-00035

Schouten JW, 2004, J NEUROTRAUM, V21, P1501, DOI 10.1089/0897715042441774

Shear DA, 2004, BRAIN RES, V1026, P11, DOI 10.1016/j.brainres.2004.07.087

Skardelly M, 2011, J NEUROTRAUM, V28, P401, DOI 10.1089/neu.2010.1526

Stabenfeldt SE, 2011, CURR STEM CELL RES T, V6, P208

Tagliaferri F, 2006, ACTA NEUROCHIR, V148, P255, DOI 10.1007/s00701-005-0651-y

TEASDALE G, 1974, LANCET, V2, P81

Wang EY, 2012, J NEUROTRAUM, V29, P295, DOI 10.1089/neu.2011.2043

Yan ZJ, 2013, NEUROCHEM RES, V38, P1022, DOI 10.1007/s11064-013-1012-5

NR 38

TC 30

Z9 31

U1 0

U2 8

PU SPANDIDOS PUBL LTD

PI ATHENS

PA POB 18179, ATHENS, 116 10, GREECE

SN 1792-0981

EI 1792-1015

J9 EXP THER MED

JI Exp. Ther. Med.

PD JUN

PY 2017

VL 13

IS 6

BP 3613

EP 3618

DI 10.3892/etm.2017.4423

PG 6

WC Medicine, Research & Experimental

WE Science Citation Index Expanded (SCI-EXPANDED)

SC Research & Experimental Medicine

GA EU5NW

UT WOS:000401080000149

PM 28588689

OA gold, Green Published

DA 2023-06-10

ER

PT J

AU Borlongan, MC

Rosi, S

AF Borlongan, Mia C.

Rosi, Susanna

TI Stem Cell Therapy for Sequestration of Traumatic Brain Injury-Induced

Inflammation

SO INTERNATIONAL JOURNAL OF MOLECULAR SCIENCES

LA English

DT Review

DE trauma; traumatic brain injury; stem cell-based therapy; resident stem

cells; neurogenesis; inflammation; brain repair

ID UNITED-STATES; MOUSE MODEL; DISEASE; DYSFUNCTION; STRATEGIES; RESPONSES;

RECOVERY; SUBACUTE; SYSTEM; NICHE

AB Traumatic brain injury (TBI) is one of the leading causes of long-term neurological disabilities in the world. TBI is a signature disease for soldiers and veterans, but also affects civilians, including adults and children. Following TBI, the brain resident and immune cells turn into a "reactive" state, characterized by the production of inflammatory mediators that contribute to the development of cognitive deficits. Other injuries to the brain, including radiation exposure, may trigger TBI-like pathology, characterized by inflammation. Currently there are no treatments to prevent or reverse the deleterious consequences of brain trauma. The recognition that TBI predisposes stem cell alterations suggests that stem cell-based therapies stand as a potential treatment for TBI. Here, we discuss the inflamed brain after TBI and radiation injury. We further review the status of stem cells in the inflamed brain and the applications of cell therapy in sequestering inflammation in TBI.

C1 [Borlongan, Mia C.; Rosi, Susanna] Univ Calif San Francisco, Dept Phys Therapy & Rehabil Sci, San Francisco, CA 94143 USA.

[Rosi, Susanna] Univ Calif San Francisco, Brain & Spinal Injury Ctr, San Francisco, CA 94110 USA.

[Rosi, Susanna] Univ Calif San Francisco, Dept Neurol Surg, San Francisco, CA 94143 USA.

[Rosi, Susanna] Univ Calif San Francisco, Weill Inst Neurosci, San Francisco, CA 94158 USA.

[Rosi, Susanna] Altos Labs, Redwood City, CA 94065 USA.

C3 University of California System; University of California San Francisco;

University of California System; University of California San Francisco;

University of California System; University of California San Francisco;

University of California System; University of California San Francisco

RP Rosi, S (通讯作者)，Univ Calif San Francisco, Dept Phys Therapy & Rehabil Sci, San Francisco, CA 94143 USA.; Rosi, S (通讯作者)，Univ Calif San Francisco, Brain & Spinal Injury Ctr, San Francisco, CA 94110 USA.; Rosi, S (通讯作者)，Univ Calif San Francisco, Dept Neurol Surg, San Francisco, CA 94143 USA.; Rosi, S (通讯作者)，Univ Calif San Francisco, Weill Inst Neurosci, San Francisco, CA 94158 USA.; Rosi, S (通讯作者)，Altos Labs, Redwood City, CA 94065 USA.

EM srosi@altoslabs.com

FU National Institutes of Health [R01CA133216, R01CA213441, R01AG056770];

NASA [NNX14AC94G, 80NSSC19K1581]

FX This work was supported by National Institutes of Health grants

R01CA133216, R01CA213441 and R01AG056770 (S.R.) and NASA grants

NNX14AC94G (S.R.) and 80NSSC19K1581 (S.R.).

CR Acosta SA, 2014, PLOS ONE, V9, DOI 10.1371/journal.pone.0090953

Acosta SA, 2013, PLOS ONE, V8, DOI 10.1371/journal.pone.0053376

Alizada M, 2021, FOLIA NEUROPATHOL, V59, P298, DOI 10.5114/fn.2021.108536

Allen AR, 2014, INT J RADIAT BIOL, V90, P214, DOI 10.3109/09553002.2014.859761

Amaroli A, 2022, J TISSUE ENG, V13, DOI 10.1177/20417314221110192

Barretto TA, 2021, J NEUROTRAUM, V38, P2747, DOI 10.1089/neu.2021.0158

Bartl M, 2022, NEUROBIOL DIS, V170, DOI 10.1016/j.nbd.2022.105744

Belarbi K, 2013, CANCER RES, V73, P1201, DOI 10.1158/0008-5472.CAN-12-2989

Blaya MO, 2022, NEUROBIOL DIS, V164, DOI 10.1016/j.nbd.2022.105613

Broglio SP, 2012, EXERC SPORT SCI REV, V40, P138, DOI 10.1097/JES.0b013e3182524273

Caplan HW, 2021, STEM CELLS, V39, P358, DOI 10.1002/stem.3320

Chaban V, 2020, J NEUROTRAUM, V37, P2120, DOI 10.1089/neu.2019.6963

Chou A, 2018, INT J MOL SCI, V19, DOI 10.3390/ijms19061616

Cole JH, 2015, ANN NEUROL, V77, P571, DOI 10.1002/ana.24367

Coronado V.G., 2002, SURVEILL SUMM, V60, P32

Coronado VG, 2015, J HEAD TRAUMA REHAB, V30, P185, DOI 10.1097/HTR.0000000000000156

Corrigan F, 2021, SCI REP-UK, V11, DOI 10.1038/s41598-021-88237-0

Corrigan JD, 2014, J HEAD TRAUMA REHAB, V29, pE1, DOI 10.1097/HTR.0000000000000020

Cox CS, 2017, STEM CELLS, V35, P1065, DOI 10.1002/stem.2538

Cozene B, 2021, CELL TRANSPLANT, V30, DOI 10.1177/09636897211035715

dela Pena I, 2015, TRANSL STROKE RES, V6, P421, DOI 10.1007/s12975-015-0430-x

Dijkland SA, 2019, J NEUROTRAUM, DOI 10.1089/neu.2019.6401

Faden AI, 2015, NEUROTHERAPEUTICS, V12, P143, DOI 10.1007/s13311-014-0319-5

Fang MM, 2018, J IMMUNOL, V201, P2414, DOI 10.4049/jimmunol.1800252

Feng X, 2021, J NEUROINFLAMM, V18, DOI 10.1186/s12974-021-02290-0

Feng X, 2018, ELIFE, V7, DOI 10.7554/eLife.38865

Feng X, 2016, J NEUROINFLAMM, V13, DOI 10.1186/s12974-016-0671-y

Flanagan SR, 2015, ARCH PHYS MED REHAB, V96, P1753, DOI 10.1016/j.apmr.2015.07.001

Fleminger S, 2003, J NEUROL NEUROSUR PS, V74, P857, DOI 10.1136/jnnp.74.7.857

Foertsch S, 2020, NEUROSCI BIOBEHAV R, V113, P169, DOI 10.1016/j.neubiorev.2020.02.025

Frobel J, 2021, FRONT CELL DEV BIOL, V9, DOI 10.3389/fcell.2021.705410

Galgano M, 2017, CELL TRANSPLANT, V26, P1118, DOI 10.1177/0963689717714102

Gao JL, 2016, CELL TRANSPLANT, V25, P1863, DOI 10.3727/096368916X691150

Gill J, 2018, NEUROLOGY, V91, pE1385, DOI 10.1212/WNL.0000000000006321

Girard D, 2021, FRONT CELL DEV BIOL, V9, DOI 10.3389/fcell.2021.611842

Glushakova OY, 2018, J NEUROTRAUM, V35, P157, DOI 10.1089/neu.2017.4999

Granata V, 2022, FRONT IMMUNOL, V13, DOI 10.3389/fimmu.2022.884024

Gupte R, 2019, J NEUROTRAUM, V36, P3063, DOI 10.1089/neu.2018.6171

Hasselbalch HC, 2013, LEUKEMIA RES, V37, P214, DOI 10.1016/j.leukres.2012.10.020

Hawthorne AL, 2011, NEUROTHERAPEUTICS, V8, P252, DOI 10.1007/s13311-011-0032-6

Hohsfield LA, 2020, J NEUROINFLAMM, V17, DOI 10.1186/s12974-020-01931-0

Huin-Schohn C, 2013, FASEB J, V27, P333, DOI 10.1096/fj.12-217547

Jurick SM, 2021, J NEUROPSYCH CLIN N, V33, P98, DOI 10.1176/appi.neuropsych.20050128

Krukowski K, 2021, SCI ADV, V7, DOI 10.1126/sciadv.abg6702

Krukowski K, 2018, INT J MOL SCI, V19, DOI 10.3390/ijms19123753

Krukowski K, 2018, BRAIN BEHAV IMMUN, V74, P106, DOI 10.1016/j.bbi.2018.08.008

Laiakis EC, 2021, INT J MOL SCI, V22, DOI 10.3390/ijms22063070

Lee JY, 2019, THERANOSTICS, V9, P1029, DOI 10.7150/thno.29868

Lee S, 2016, J NEUROINFLAMM, V13, DOI 10.1186/s12974-016-0544-4

Li YJ, 2017, PLOS ONE, V12, DOI 10.1371/journal.pone.0169650

Lin X, 2020, INT J MOL SCI, V21, DOI 10.3390/ijms21093031

Lippa SM, 2021, J NEUROTRAUM, V38, P3137, DOI 10.1089/neu.2021.0120

Lippert T, 2019, CNS NEUROSCI THER, V25, P815, DOI 10.1111/cns.13124

Lozano D, 2015, NEUROPSYCH DIS TREAT, V11, P97, DOI 10.2147/NDT.S65815

Malec JF, 2007, J NEUROTRAUM, V24, P1417, DOI 10.1089/neu.2006.0245

Marcet Paul, 2017, Neuroimmunol Neuroinflamm, V4, P82, DOI 10.20517/2347-8659.2017.07

Masel BE, 2010, J NEUROTRAUM, V27, P1529, DOI 10.1089/neu.2010.1358

Mason HD, 2021, JCI INSIGHT, V6, DOI 10.1172/jci.insight.149229

Mazzitelli JA, 2022, NAT NEUROSCI, V25, P555, DOI 10.1038/s41593-022-01029-1

Miller GF, 2021, INJURY, V52, P1138, DOI 10.1016/j.injury.2021.01.042

Morganti JM, 2016, J NEUROINFLAMM, V13, DOI 10.1186/s12974-016-0547-1

Morganti JM, 2016, PLOS ONE, V11, DOI 10.1371/journal.pone.0148001

Morganti JM, 2015, J NEUROSCI, V35, P748, DOI 10.1523/JNEUROSCI.2405-14.2015

Morin A, 2018, FRONT AGING NEUROSCI, V10, DOI 10.3389/fnagi.2018.00292

Morrison SJ, 2014, NATURE, V505, P327, DOI 10.1038/nature12984

Neal EG, 2018, EXPERT REV NEUROTHER, V18, P557, DOI 10.1080/14737175.2018.1491309

Newell-Rogers MK, 2020, INT J MOL SCI, V21, DOI 10.3390/ijms21207448

Pabon MM, 2016, CNS NEUROSCI THER, V22, P200, DOI 10.1111/cns.12485

Paul AM, 2020, ISCIENCE, V23, DOI 10.1016/j.isci.2020.101747

Qiu XC, 2020, EXP NEUROL, V330, DOI 10.1016/j.expneurol.2020.113335

Rola R, 2008, RADIAT RES, V169, P626, DOI 10.1667/RR1263.1

Rostami T, 2022, EXP HEMATOL, V109, P27, DOI 10.1016/j.exphem.2022.02.003

San Martin Molina Isabel, 2020, eNeuro, V7, DOI 10.1523/ENEURO.0476-19.2020

Shahim P, 2020, NEUROLOGY, V95, pE623, DOI 10.1212/WNL.0000000000009985

Shi SX, 2021, SCI TRANSL MED, V13, DOI 10.1126/scitranslmed.abc7029

Song SJ, 2021, CANNABIS CANNABINOID, V6, P48, DOI 10.1089/can.2019.0090

Song SJ, 2016, J NEUROSCI RES, V94, P409, DOI 10.1002/jnr.23714

Sullivan DR, 2021, J CEREBR BLOOD F MET, V41, P886, DOI 10.1177/0271678X20935190

Taylor CA, 2017, MMWR SURVEILL SUMM, V66, P1, DOI 10.15585/mmwr.ss6609a1

Tian CL, 2013, EXP CLIN TRANSPLANT, V11, P176, DOI 10.6002/ect.2012.0053

Toyoshima A, 2015, PLOS ONE, V10, DOI 10.1371/journal.pone.0127302

Tweedie D, 2020, ELIFE, V9, DOI 10.7554/eLife.55827

Vedantam A, 2021, J NEUROTRAUM, V38, P53, DOI 10.1089/neu.2019.6979

Verboon LN, 2021, FRONT IMMUNOL, V12, DOI 10.3389/fimmu.2021.620698

Waclawiczek A, 2020, J CLIN INVEST, V130, P3038, DOI 10.1172/JCI133187

Wang J, 2022, J CLIN MED, V11, DOI 10.3390/jcm11113223

Washington PM, 2016, EXP NEUROL, V275, P381, DOI 10.1016/j.expneurol.2015.06.015

Weiss E, 2020, CLIN EXP EMERG MED, V7, P87, DOI 10.15441/ceem.19.050

Wnorowski A, 2019, STEM CELL REP, V13, P960, DOI 10.1016/j.stemcr.2019.10.006

Wolf D, 2015, CIRC RES, V116, P389, DOI 10.1161/CIRCRESAHA.114.305678

Wright WG, 2022, J NEUROTRAUM, V39, P821, DOI 10.1089/neu.2020.7600

Xu LB, 2021, J NEUROTRAUM, V38, P918, DOI 10.1089/neu.2020.7177

Zhao YF, 2022, J NEUROINFLAMM, V19, DOI 10.1186/s12974-022-02563-2

NR 93

TC 0

Z9 0

U1 3

U2 5

PU MDPI

PI BASEL

PA ST ALBAN-ANLAGE 66, CH-4052 BASEL, SWITZERLAND

EI 1422-0067

J9 INT J MOL SCI

JI Int. J. Mol. Sci.

PD SEP

PY 2022

VL 23

IS 18

AR 10286

DI 10.3390/ijms231810286

PG 11

WC Biochemistry & Molecular Biology; Chemistry, Multidisciplinary

WE Science Citation Index Expanded (SCI-EXPANDED)

SC Biochemistry & Molecular Biology; Chemistry

GA 4S6QX

UT WOS:000857564200001

PM 36142198

OA Green Published, gold

DA 2023-06-10

ER

PT J

AU Corral, L

Conde, L

Guillamo, E

Blasi, J

Juncadella, M

Javierre, C

Viscor, G

Ventura, JL

AF Corral, Luisa

Conde, Laura

Guillamo, Elisabet

Blasi, Juan

Juncadella, Montserrat

Javierre, Casimiro

Viscor, Gines

Ventura, Josep L.

TI Circulating progenitor cells during exercise, muscle electro-stimulation

and intermittent hypobaric hypoxia in patients with traumatic brain

injury: A pilot study

SO NEUROREHABILITATION

LA English

DT Article

DE Traumatic brain injury; circulating progenitor cells; exercise;

physiology

ID PHYSICAL-ACTIVITY; NEUROGENESIS

AB BACKGROUND: Circulating progenitor cells (CPC) treatments may have great potential for the recovery of neurons and brain function.

OBJECTIVE: To increase and maintain CPC with a program of exercise, muscle electro-stimulation (ME) and/or intermittent-hypobaric-hypoxia (IHH), and also to study the possible improvement in physical or psychological functioning of participants with Traumatic Brain Injury (TBI).

METHODS: Twenty-one participants. Four groups: exercise and ME group (EEG), cycling group (CyG), IHH and ME group (HEG) and control group (CG). Psychological and physical stress tests were carried out. CPC were measured in blood several times during the protocol.

RESULTS: Psychological tests did not change. In the physical stress tests the VO2 uptake increased in the EEG and the CyG, and the maximal tolerated workload increased in the HEG. CPC levels increased in the last three weeks in EEG, but not in CyG, CG and HEG.

CONCLUSIONS: CPC levels increased in the last three weeks of the EEG program, but not in the other groups and we did not detect performed psychological test changes in any group. The detected aerobic capacity or workload improvement must be beneficial for the patients who have suffered TBI, but exercise type and the mechanisms involved are not clear.

C1 [Corral, Luisa; Conde, Laura; Juncadella, Montserrat; Ventura, Josep L.] Bellvitge Univ Hosp, Intens Care Unit, Barcelona 08907, Spain.

[Corral, Luisa; Conde, Laura; Juncadella, Montserrat; Ventura, Josep L.] Bellvitge Univ Hosp, Neuropsychol Dept, Barcelona 08907, Spain.

[Corral, Luisa; Guillamo, Elisabet; Blasi, Juan; Javierre, Casimiro; Viscor, Gines] Univ Barcelona, Dept Physiol Sci Pathol & Expt Therapeut & Physio, Barcelona 08907, Spain.

C3 Institut d'Investigacio Biomedica de Bellvitge (IDIBELL); Bellvitge

University Hospital; University of Barcelona; Institut d'Investigacio

Biomedica de Bellvitge (IDIBELL); Bellvitge University Hospital;

University of Barcelona; University of Barcelona

RP Corral, L (通讯作者)，Bellvitge Univ Hosp, Intens Care Unit, Feixa Llarga S-N, Barcelona 08907, Spain.

EM lcorral@bellvitgehospital.cat

RI Viscor, Ginés/A-9519-2008; Blasi, Juan/K-7943-2014

OI Viscor, Ginés/0000-0003-4942-2346; Blasi, Juan/0000-0002-0482-9444;

Corral, Luisa/0000-0002-2888-7833

CR Bennie SD, 2002, EUR J APPL PHYSIOL, V88, P13, DOI 10.1007/s00421-002-0711-4

Bonsignore MR, 2010, J APPL PHYSIOL, V109, P60, DOI 10.1152/japplphysiol.01344.2009

Corral L, 2007, BRAIN INJURY, V21, P1225, DOI 10.1080/02699050701727460

Driver S, 2009, BRAIN INJURY, V23, P203, DOI 10.1080/02699050802695574

Elder GA, 2006, MT SINAI J MED, V73, P931

Guo X., 2009, J NEUROTRAUMA

Keeney M, 1998, CYTOMETRY, V34, P61, DOI 10.1002/(SICI)1097-0320(19980415)34:2<61::AID-CYTO1>3.3.CO;2-6

Koutroumpi M, 2012, WORLD J CARDIOL, V4, P312, DOI 10.4330/wjc.v4.i12.312

Lysak D, 2010, INT J LAB HEMATOL, V32, pE229, DOI 10.1111/j.1751-553X.2010.01244.x

Mobius-Winkler S, 2009, J APPL PHYSIOL, V107, P1943, DOI 10.1152/japplphysiol.00532.2009

Ploughman M, 2008, DEV NEUROREHABIL, V11, P236, DOI 10.1080/17518420801997007

Viscor G, 2009, J TRANSL MED, V7, DOI 10.1186/1479-5876-7-91

Wang JS, 2014, INT J CARDIOL, V170, P315, DOI 10.1016/j.ijcard.2013.11.005

Whitnall L, 2006, J NEUROL NEUROSUR PS, V77, P640, DOI 10.1136/jnnp.2005.078246

Xu Q, 2007, CLIN EXP PHARMACOL P, V34, P624, DOI 10.1111/j.1440-1681.2007.04619.x

Zhu LL, 2005, BRAIN RES, V1055, P1, DOI 10.1016/j.brainres.2005.04.075

NR 16

TC 3

Z9 3

U1 0

U2 8

PU IOS PRESS

PI AMSTERDAM

PA NIEUWE HEMWEG 6B, 1013 BG AMSTERDAM, NETHERLANDS

SN 1053-8135

EI 1878-6448

J9 NEUROREHABILITATION

JI Neurorehabilitation

PY 2014

VL 35

IS 4

BP 763

EP 769

DI 10.3233/NRE-141172

PG 7

WC Clinical Neurology; Rehabilitation

WE Science Citation Index Expanded (SCI-EXPANDED); Social Science Citation Index (SSCI)

SC Neurosciences & Neurology; Rehabilitation

GA AW4HK

UT WOS:000346241600014

PM 25318779

OA Green Published

DA 2023-06-10

ER

PT J

AU Wang, L

Wang, XN

Su, H

Han, ZY

Yu, HJ

Wang, D

Jiang, RC

Liu, ZL

Zhang, JN

AF Wang, Liang

Wang, Xiaonan

Su, Hua

Han, Zhenying

Yu, Huijie

Wang, Dong

Jiang, Rongcai

Liu, Zhenlin

Zhang, Jianning

TI Recombinant Human Erythropoietin Improves the Neurofunctional Recovery

of Rats Following Traumatic Brain Injury via an Increase in Circulating

Endothelial Progenitor Cells

SO TRANSLATIONAL STROKE RESEARCH

LA English

DT Article

DE Traumatic brain injury; Erythropoietin; Endothelial progenitor cells;

Angiogenesis

ID ISCHEMIA-REPERFUSION INJURY; FUNCTIONAL OUTCOMES; CEREBRAL-ISCHEMIA;

NEOVASCULARIZATION; MICE; TRANSPLANTATION; PROTECTS; ANGIOGENESIS;

MYOCARDIUM; STROKE

AB Previous studies show that circulating endothelial progenitor cells (EPCs) promote angiogenesis, which is a process associated with improved recovery in animal models of traumatic brain injury (TBI), and that recombinant human erythropoietin (rhEPO) plays a protective role following stroke. Thus, it was hypothesized that rhEPO would enhance recovery following brain injury in a rat model of TBI via an increase in the mobilization of EPCs and, subsequently, in angiogenesis. Flow cytometry assays using CD34- and CD133-specific antibodies were utilized to identify alterations in EPC levels, CD31 and CD34 antibody-stained brain tissue sections were used to quantify angiogenesis, and the Morris water maze (MWM) test and the modified Neurological Severity Score (mNSS) test were used to evaluate behavioral recovery. Compared with saline treatment, treatment with rhEPO significantly increased the number of circulating EPCs on days 1, 4, 7, and 14 (P < 0.05), improved spatial learning ability on days 24 and 25 (P < 0.05), and enhanced memory recovery on day 26 (P < 0.05). Moreover, rhEPO treatment decreased mNSS assessment scores on days 14, 21, and 25 (P < 0.05). There was a strong correlation between levels of circulating EPCs and CD34- and CD31-positive cells within the injured boundary zone (CD34(+) r = 0.910, P < 0.01; CD31(+) r = 0.894, P < 0.01) and the ipsilateral hippocampus (CD34(+) r = 0.841, P < 0.01; CD31(+) r = 0.835, P < 0.01). The present data demonstrate that rhEPO treatment improved functional outcomes in rats following TBI via an increase in the mobilization of EPCs and in subsequent angiogenesis.

C1 [Wang, Liang; Liu, Zhenlin] Tianjin 5th Ctr Hosp, Dept Neurosurg, Tianjin 300450, Peoples R China.

[Wang, Xiaonan] Nankai Hosp, Dept Neurosurg, Tianjin 300102, Peoples R China.

[Su, Hua] Univ Calif San Francisco, Dept Anesthesia & Perioperat Care, Cerebrovasc Res Ctr, San Francisco, CA 94110 USA.

[Han, Zhenying; Yu, Huijie; Wang, Dong; Jiang, Rongcai; Zhang, Jianning] Tianjin Med Univ, Gen Hosp, Dept Neurosurg, Tianjin 300052, Peoples R China.

C3 University of California System; University of California San Francisco;

Tianjin Medical University

RP Liu, ZL (通讯作者)，Tianjin 5th Ctr Hosp, Dept Neurosurg, 41 Zhejiang Rd, Tianjin 300450, Peoples R China.

EM wjzhenlin817@sina.com; jianningzhang@hotmail.com

FU Emerging Project Committee of Science and Technology of Tanggu District

of Tianjin, China [2012XQ15-07]; National Natural Science Foundation of

China [81100920, 81200907]; Tianjin Research Program of Application

Foundation and Advanced Technology [12JCQNJC6800]

FX We thank Li Liu, Weiyun Cui, Fanglian Chen, and Lei Zhang for their

excellent technical support. The authors thank Voltaire Gungab for the

editorial assistance. This work was supported by grants from the

Emerging Project Committee of Science and Technology of Tanggu District

of Tianjin (2012XQ15-07), China; the National Natural Science Foundation

of China (grants 81100920 and 81200907); and Tianjin Research Program of

Application Foundation and Advanced Technology (grants12JCQNJC6800).

CR Asahara T, 1997, SCIENCE, V275, P964, DOI 10.1126/science.275.5302.964

Bahlmann FH, 2004, BLOOD, V103, P921, DOI 10.1182/blood-2003-04-1284

Calvillo L, 2003, P NATL ACAD SCI USA, V100, P4802, DOI 10.1073/pnas.0630444100

Celik M, 2002, P NATL ACAD SCI USA, V99, P2258, DOI 10.1073/pnas.042693799

Chen JL, 2001, STROKE, V32, P1005, DOI 10.1161/01.STR.32.4.1005

Chopp M, 2008, J NEUROL SCI, V265, P97, DOI 10.1016/j.jns.2007.06.013

Clarkson AN, 2007, BRAIN RES, V1171, P111, DOI 10.1016/j.brainres.2007.06.100

Fan YF, 2010, ANN NEUROL, V67, P488, DOI 10.1002/ana.21919

Gao DC, 2008, SCIENCE, V319, P195, DOI 10.1126/science.1150224

Gehling UM, 2000, BLOOD, V95, P3106

Gong DS, 2012, BRAIN INJURY, V26, P291, DOI 10.3109/02699052.2011.648710

Grisar JC, 2011, BIOMARK MED, V5, P731, DOI [10.2217/BMM.11.92, 10.2217/bmm.11.92]

Hirata A, 2006, J AM COLL CARDIOL, V48, P176, DOI 10.1016/j.jacc.2006.04.008

Hyder AA, 2007, NEUROREHABILITATION, V22, P341

Junk AK, 2002, P NATL ACAD SCI USA, V99, P10659, DOI 10.1073/pnas.152321399

Kalka C, 2000, P NATL ACAD SCI USA, V97, P3422, DOI 10.1073/pnas.070046397

Kawamoto A, 2001, CIRCULATION, V103, P634

Ladhoff J, 2010, CARDIOVASC RES, V88, P121, DOI 10.1093/cvr/cvq109

Li B, 2006, FASEB J, V20, P1495, DOI 10.1096/fj.05-5137fje

Li ZY, 2012, J NEUROTRAUM, V29, P343, DOI 10.1089/neu.2011.1807

Lipsic E, 2004, J CARDIOVASC PHARM, V44, P473, DOI 10.1097/01.fjc.0000140209.04675.c3

Liu L, 2007, J NEUROTRAUM, V24, P936, DOI 10.1089/neu.2006.0250

Lu DY, 2007, J NEUROTRAUM, V24, P1132, DOI 10.1089/neu.2007.0288

Maeng YS, 2009, BLOOD, V113, P233, DOI 10.1182/blood-2008-06-162891

MCINTOSH TK, 1989, NEUROSCIENCE, V28, P233, DOI 10.1016/0306-4522(89)90247-9

MORRIS R, 1984, J NEUROSCI METH, V11, P47, DOI 10.1016/0165-0270(84)90007-4

Ozisik PA, 2007, SURG NEUROL, V68, P547, DOI 10.1016/j.surneu.2007.01.030

Park KJ, 2014, J CEREBR BLOOD F MET, V34, P357, DOI 10.1038/jcbfm.2013.216

Potts Mathew B, 2006, NeuroRx, V3, P143

Resch T, 2012, STEM CELL REV REP, V8, P926, DOI 10.1007/s12015-011-9332-9

Sakanaka M, 1998, P NATL ACAD SCI USA, V95, P4635, DOI 10.1073/pnas.95.8.4635

Satoh K, 2006, CIRCULATION, V113, P1442, DOI 10.1161/CIRCULATIONAHA.105.583732

Sobrino T, 2007, STROKE, V38, P2759, DOI 10.1161/STROKEAHA.107.484386

Strauer BE, 2002, CIRCULATION, V106, P1913, DOI 10.1161/01.CIR.0000034046.87607.1C

Tei K, 2008, STEM CELLS, V26, P819, DOI 10.1634/stemcells.2007-0671

Verdonck O, 2007, J CEREBR BLOOD F MET, V27, P1369, DOI 10.1038/sj.jcbfm.9600443

Wang B, 2012, J NEUROL SCI, V319, P117, DOI 10.1016/j.jns.2012.04.015

Wang L, 2004, NEUROREPORT, V15, P1225, DOI 10.1097/01.wnr.0000127636.15181.c1

Westenbrink BD, 2007, EUR HEART J, V28, P2018, DOI 10.1093/eurheartj/ehm177

Xiong Y, 2008, BRAIN RES, V1230, P247, DOI 10.1016/j.brainres.2008.06.127

Xiong Y, 2008, J NEUROSURG, V109, P510, DOI 10.3171/JNS/2008/109/9/0510

Xiong Y, 2011, TRANSL STROKE RES, V2, P619, DOI 10.1007/s12975-011-0120-2

Xiong Y, 2010, J NEUROTRAUM, V27, P205, DOI 10.1089/neu.2009.1001

Yip HK, 2011, CRIT CARE, V15, DOI 10.1186/cc10002

Yuan Q, 2012, INJURY, V43, P2094, DOI 10.1016/j.injury.2012.03.028

Zhang YQ, 2013, J SURG RES, V185, P441, DOI 10.1016/j.jss.2013.05.073

Zhang ZG, 2002, CIRC RES, V90, P284, DOI 10.1161/hh0302.104460

NR 47

TC 44

Z9 48

U1 0

U2 7

PU SPRINGER

PI NEW YORK

PA 233 SPRING ST, NEW YORK, NY 10013 USA

SN 1868-4483

EI 1868-601X

J9 TRANSL STROKE RES

JI Transl. Stroke Res.

PD FEB

PY 2015

VL 6

IS 1

BP 50

EP 59

DI 10.1007/s12975-014-0362-x

PG 10

WC Clinical Neurology; Neurosciences

WE Science Citation Index Expanded (SCI-EXPANDED)

SC Neurosciences & Neurology

GA AZ3IT

UT WOS:000348121500007

PM 25085436

OA Green Accepted, Green Submitted

DA 2023-06-10

ER

PT J

AU Park, KJ

Park, E

Liu, E

Baker, AJ

AF Park, Katya J.

Park, Eugene

Liu, Elaine

Baker, Andrew J.

TI Bone marrow-derived endothelial progenitor cells protect postischemic

axons after traumatic brain injury

SO JOURNAL OF CEREBRAL BLOOD FLOW AND METABOLISM

LA English

DT Article

DE endothelial progenitor cell; fluid percussion injury; ischemia;

traumatic brain injury; vasculature; white matter injury

ID FOCAL CEREBRAL-ISCHEMIA; CYTOCHEMICAL EVIDENCE; CALCIUM INFLUX; CD34(+)

CELLS; NERVE-FIBERS; OPTIC-NERVE; ANGIOGENESIS; STROKE; PROTEOGLYCANS;

EXPRESSION

AB White matter sparing after traumatic brain injury (TBI) is an important predictor of survival and outcome. Blood vessels and axons are intimately associated anatomically and developmentally. Neural input is required for appropriate vascular patterning, and vascular signaling is important for neuron development and axon growth. Owing to this codependence between endothelial cells and axons during development and the contribution of endothelial progenitor cells (EPCs) in ischemic injury, we hypothesized that EPCs are important in axonal survival after TBI. We examined the effects of allogenic-cultured EPCs on white matter protection and microvascular maintenance after midline fluid percussion injury in adult Sprague-Dawley rats. We used two in vitro models of injury, mechanical stretch and oxygen-glucose deprivation (OGD), to examine the effects of EPCs on the mechanical and ischemic components of brain trauma, respectively. Our results indicate that EPCs improve the white matter integrity and decrease capillary breakdown after injury. Cultured cortical neurons exposed to OGD had less axon degeneration when treated with EPC-conditioned media, whereas no effect was seen in axons injured by mechanical stretch. The results indicate that EPCs are important for the protection of the white matter after trauma and represent a potential avenue for therapy.

C1 [Park, Katya J.; Park, Eugene; Liu, Elaine; Baker, Andrew J.] St Michaels Hosp, Keenan Res Ctr, Li Ka Shing Knowledge Inst, Toronto, ON M5B 1W8, Canada.

[Baker, Andrew J.] St Michaels Hosp, Dept Crit Care, Toronto, ON M5B 1W8, Canada.

[Baker, Andrew J.] St Michaels Hosp, Dept Anesthesia, Toronto, ON M5B 1W8, Canada.

[Baker, Andrew J.] Univ Toronto, Dept Anesthesia, Toronto, ON, Canada.

[Baker, Andrew J.] Univ Toronto, Interdepartmental Div Crit Care, Toronto, ON, Canada.

C3 University of Toronto; Li Ka Shing Knowledge Institute; Saint Michaels

Hospital Toronto; University of Toronto; Saint Michaels Hospital

Toronto; University of Toronto; Saint Michaels Hospital Toronto;

University of Toronto; University of Toronto

RP Park, E (通讯作者)，St Michaels Hosp, Keenan Res Ctr, Li Ka Shing Knowledge Inst, 30 Bond St, Toronto, ON M5B 1W8, Canada.

EM parke@smh.ca

FU Ministry of Economic Development and Innovation, Government of Ontario

FX This study was funded by the Ministry of Economic Development and

Innovation, Government of Ontario.

CR Baker AJ, 2002, J NEUROTRAUM, V19, P587, DOI 10.1089/089771502753754064

Bell JD, 2009, CELL DEATH DIFFER, V16, P1665, DOI 10.1038/cdd.2009.106

Borlongan CV, 2009, STROKE, V40, pS146, DOI 10.1161/STROKEAHA.108.533091

Buki A, 2006, ACTA NEUROCHIR, V148, P181, DOI 10.1007/s00701-005-0674-4

Burlacu A, 2013, STEM CELLS DEV, V22, P643, DOI 10.1089/scd.2012.0273

Carmeliet P, 2005, NATURE, V436, P193, DOI 10.1038/nature03875

Chen J, 2012, PLOS ONE, V7, DOI 10.1371/journal.pone.0050105

Chen X, 2013, ACTA RADIOL, V54, P313, DOI 10.1258/ar.2012.120605

Di Santo S, 2009, PLOS ONE, V4, DOI 10.1371/journal.pone.0005643

Fan Y, ANN NEUROL, V67, P488

Farahvar A, 2012, J NEUROSURG, V117, P729, DOI 10.3171/2012.7.JNS111816

Gong DS, 2012, BRAIN INJURY, V26, P291, DOI 10.3109/02699052.2011.648710

He TR, 2004, STROKE, V35, P2378, DOI 10.1161/01.STR.0000141893.33677.5d

He XY, 2011, CYTOTHERAPY, V13, P46, DOI 10.3109/14653249.2010.510505

Honma Y, 2002, NEURON, V35, P267, DOI 10.1016/S0896-6273(02)00774-2

JOHANSSON S, 1985, BIOCHEM J, V232, P161, DOI 10.1042/bj2320161

Johnson VE, 2013, EXP NEUROL, V246, P35, DOI 10.1016/j.expneurol.2012.01.013

Joyce N, 2010, REGEN MED, V5, P933, DOI 10.2217/RME.10.72

KALARIA RN, 1993, ANN NY ACAD SCI, V695, P190, DOI 10.1111/j.1749-6632.1993.tb23050.x

Kuruvilla R, 2004, CELL, V118, P243, DOI 10.1016/j.cell.2004.06.021

Ladhoff J, 2010, CARDIOVASC RES, V88, P121, DOI 10.1093/cvr/cvq109

Lee AF, 2004, J NEUROSCI, V24, P9174, DOI 10.1523/JNEUROSCI.1588-04.2004

Maxwell WL, 1995, J NEUROCYTOL, V24, P925, DOI 10.1007/BF01215643

Maxwell WL, 1999, J NEUROTRAUM, V16, P273, DOI 10.1089/neu.1999.16.273

Maxwell WL, 1997, J NEUROTRAUM, V14, P419, DOI 10.1089/neu.1997.14.419

Mielke JG, 2005, J NEUROCHEM, V92, P103, DOI 10.1111/j.1471-4159.2004.02841.x

Mukouyama Y, 2002, CELL, V109, P693, DOI 10.1016/S0092-8674(02)00757-2

Ohta T, 2006, NEUROSURGERY, V59, P679, DOI 10.1227/01.NEU.0000229058.08706.88

Park E, 2009, J CEREBR BLOOD F MET, V29, P575, DOI 10.1038/jcbfm.2008.151

Peichev M, 2000, BLOOD, V95, P952, DOI 10.1182/blood.V95.3.952.003k27_952_958

Resch T, 2012, STEM CELL REV REP, V8, P926, DOI 10.1007/s12015-011-9332-9

Siddiq I, 2012, J NEUROTRAUM, V29, P2647, DOI 10.1089/neu.2012.2444

Siebert JR, 2011, J NEUROCHEM, V119, P176, DOI 10.1111/j.1471-4159.2011.07370.x

Sobrino T, 2007, STROKE, V38, P2759, DOI 10.1161/STROKEAHA.107.484386

Stein SC, 2004, NEUROSURGERY, V54, P687, DOI 10.1227/01.NEU.0000108641.98845.88

Taguchi A, 2004, J CLIN INVEST, V114, P330, DOI 10.1172/jci200420622

Urbich C, 2004, TRENDS CARDIOVAS MED, V14, P318, DOI 10.1016/j.tcm.2004.10.001

Wolf JA, 2001, J NEUROSCI, V21, P1923, DOI 10.1523/JNEUROSCI.21-06-01923.2001

YAMAGATA M, 1986, J BIOL CHEM, V261, P3526

Zhang ZG, 2002, CIRC RES, V90, P284, DOI 10.1161/hh0302.104460

NR 40

TC 35

Z9 35

U1 0

U2 8

PU SAGE PUBLICATIONS INC

PI THOUSAND OAKS

PA 2455 TELLER RD, THOUSAND OAKS, CA 91320 USA

SN 0271-678X

EI 1559-7016

J9 J CEREBR BLOOD F MET

JI J. Cereb. Blood Flow Metab.

PD FEB

PY 2014

VL 34

IS 2

BP 357

EP 366

DI 10.1038/jcbfm.2013.216

PG 10

WC Endocrinology & Metabolism; Hematology; Neurosciences

WE Science Citation Index Expanded (SCI-EXPANDED)

SC Endocrinology & Metabolism; Hematology; Neurosciences & Neurology

GA 304LK

UT WOS:000330748200022

PM 24301295

OA Bronze, Green Published

DA 2023-06-10

ER

PT J

AU Assis-Nascimento, P

Umland, O

Cepero, ML

Liebl, DJ

AF Assis-Nascimento, Poincyane

Umland, Oliver

Cepero, Maria L.

Liebl, Daniel J.

TI A flow cytometric approach to analyzing mature and progenitor

endothelial cells following traumatic brain injury

SO JOURNAL OF NEUROSCIENCE METHODS

LA English

DT Article

DE Flow cytometry; Endothelial cells; Endothelial progenitor cells; Blood;

Vessels; Traumatic brain injury

ID RAT MODEL; ANGIOGENESIS; TISSUE; PERFUSION; IDENTITY

AB Background: Traumatic brain injury (TBI) continues to be a major source of death and disability worldwide, and one of the earliest and most profound deficits comes from vascular damage and breakdown of the blood-brain barrier (BBB). Cerebral vascular endothelial cells (cvECs) and endothelial progenitor cells (EPCs) have been shown to play essential roles in vessel repair and BBB stability, although their individual contributions remain poorly defined.

New method: We employ TruCount beads with flow cytometry to precisely quantify cvECs, EPCs, and peripheral leukocytes in the murine cortex after controlled cortical impact (CCI) injury.

Results: We found a significant reduction in the number of cvECs at 3 days post-injury (dpi), whereas the EPCs and invading peripheral leukocytes were significantly increased compared with sham controls. Proliferation studies demonstrate that both cvECs and EPCs are undergoing cell expansion in the first week post-injury. Furthermore, analysis of protein expression using mean fluorescence intensity found increases in PECAM-1, VEGFR-2, and VE-Cadherin expression per cell at 3 dpi, which is consistent with western blot analysis.

Comparison with existing methods: Classic methods of cell analysis, such as histological cell counts, in the traumatic injured brain are labor intensive, time-consuming, and potentially biased; whereas flow cytometry provides an efficient, non-biased approach to simultaneously quantify multiple cell types. However, conventional flow cytometry that employs capped events can provide misleading results in CNS injured tissues.

Conclusions: We demonstrate that TruCount quantification using flow cytometry is a powerful tool for quantifying mature and progenitor endothelial cell changes after TBI. (C) 2016 Elsevier B.V. All rights reserved.

C1 [Assis-Nascimento, Poincyane; Cepero, Maria L.; Liebl, Daniel J.] Univ Miami, Miller Sch Med, Dept Neurosurg, Miami Project Cure Paralysis, Miami, FL 33136 USA.

[Umland, Oliver] Univ Miami, Miller Sch Med, Diabet Res Inst, Miami, FL 33136 USA.

C3 University of Miami; University of Miami

RP Liebl, DJ (通讯作者)，Univ Miami, Neurol Surg, Miami Project Cure Paralysis, 1095 NW 14th Terrace,R-48, Miami, FL 33136 USA.

EM dliebl@miami.edu

FU NINDS NIH HHS [P50 NS030291, F31 NS089325, R01 NS049545] Funding Source:

Medline

CR Abbott NJ, 2010, NEUROBIOL DIS, V37, P13, DOI 10.1016/j.nbd.2009.07.030

Abdul-Muneer PM, 2013, FREE RADICAL BIO MED, V60, P282, DOI 10.1016/j.freeradbiomed.2013.02.029

Bendall SC, 2012, TRENDS IMMUNOL, V33, P323, DOI 10.1016/j.it.2012.02.010

Chen S, 2003, EXP NEUROL, V182, P87, DOI 10.1016/S0014-4886(03)00002-5

Chen X, 2013, ACTA RADIOL, V54, P313, DOI 10.1258/ar.2012.120605

Das M, 2012, J NEUROINFLAMM, V9, DOI 10.1186/1742-2094-9-236

De Rosa SC, 2001, NAT MED, V7, P245, DOI 10.1038/84701

Greve MW, 2009, MT SINAI J MED, V76, P97, DOI 10.1002/msj.20104

Guo XB, 2009, J NEUROTRAUM, V26, P1337, DOI [10.1089/neu.2008.0733, 10.1089/neu.2008-0733]

Hayashi T, 2003, J CEREBR BLOOD F MET, V23, P166, DOI 10.1097/00004647-200302000-00004

Immonen R, 2010, J CEREBR BLOOD F MET, V30, P1318, DOI 10.1038/jcbfm.2010.15

Jin XM, 2012, PLOS ONE, V7, DOI [10.1371/journal.pone.0040637, 10.1371/journal.pone.0041892]

Kan EM, 2012, BRAIN RES BULL, V87, P359, DOI 10.1016/j.brainresbull.2012.01.007

Lin Y, 2012, LAB INVEST, V92, P1623, DOI 10.1038/labinvest.2012.118

Liu L, 2007, J NEUROTRAUM, V24, P936, DOI 10.1089/neu.2006.0250

Lugli E, 2010, CYTOM PART A, V77A, P705, DOI 10.1002/cyto.a.20901

Mair F, 2016, EUR J IMMUNOL, V46, P34, DOI 10.1002/eji.201545774

Melero-Martin JM, 2007, BLOOD, V109, P4761, DOI 10.1182/blood-2006-12-062471

Morgan R, 2007, NEUROL RES, V29, P375, DOI 10.1179/016164107X204693

Nag S, 1997, J NEUROPATH EXP NEUR, V56, P912, DOI 10.1097/00005072-199708000-00009

Perfetto SP, 2004, NAT REV IMMUNOL, V4, P648, DOI 10.1038/nri1416

Sorensen I, 2009, BLOOD, V113, P5680, DOI 10.1182/blood-2008-08-174508

Timmermans F, 2009, J CELL MOL MED, V13, P87, DOI 10.1111/j.1582-4934.2008.00598.x

Toklu H. Z., 2015, BRAIN NEUROTRAUMA MO

Wright DW, 2013, MMWR-MORBID MORTAL W, V62, P549

Wylot B, 2015, CYTOM PART A, V87A, P908, DOI 10.1002/cyto.a.22677

Xue S, 2010, NEUROSCI LETT, V473, P186, DOI 10.1016/j.neulet.2010.02.035

Zhang XP, 2005, CRIT CARE, V9, P66, DOI 10.1186/cc2950

NR 28

TC 11

Z9 12

U1 2

U2 11

PU ELSEVIER SCIENCE BV

PI AMSTERDAM

PA PO BOX 211, 1000 AE AMSTERDAM, NETHERLANDS

SN 0165-0270

EI 1872-678X

J9 J NEUROSCI METH

JI J. Neurosci. Methods

PD APR 1

PY 2016

VL 263

BP 57

EP 67

DI 10.1016/j.jneumeth.2016.01.025

PG 11

WC Biochemical Research Methods; Neurosciences

WE Science Citation Index Expanded (SCI-EXPANDED)

SC Biochemistry & Molecular Biology; Neurosciences & Neurology

GA DJ4SY

UT WOS:000374199500007

PM 26854397

OA Green Accepted

DA 2023-06-10

ER

PT J

AU He, ZH

Lang, LJ

Hui, JY

Ma, YX

Yang, C

Weng, WJ

Huang, JL

Zhao, XF

Zhang, XQ

Liang, Q

Jiang, JY

Feng, JF

AF He, Zhenghui

Lang, Lijian

Hui, Jiyuan

Ma, Yuxiao

Yang, Chun

Weng, Weiji

Huang, Jialin

Zhao, Xiongfei

Zhang, Xiaoqi

Liang, Qian

Jiang, Jiyao

Feng, Junfeng

TI Brain Extract of Subacute Traumatic Brain Injury Promotes the Neuronal

Differentiation of Human Neural Stem Cells via Autophagy

SO JOURNAL OF CLINICAL MEDICINE

LA English

DT Article

DE traumatic brain injury; neural stem cells; differentiation; autophagy

ID TRANSPLANTATION; PROLIFERATION; SCAFFOLDS; COMPLEX

AB Background: After a traumatic brain injury (TBI), the cell environment is dramatically changed, which has various influences on grafted neural stem cells (NSCs). At present, these influences on NSCs have not been fully elucidated, which hinders the finding of an optimal timepoint for NSC transplantation. Methods: Brain extracts of TBI mice were used in vitro to simulate the different phase TBI influences on the differentiation of human NSCs. Protein profiles of brain extracts were analyzed. Neuronal differentiation and the activation of autophagy and the WNT/CTNNB pathway were detected after brain extract treatment. Results: Under subacute TBI brain extract conditions, the neuronal differentiation of hNSCs was significantly higher than that under acute brain extract conditions. The autophagy flux and WNT/CTNNB pathway were activated more highly within the subacute brain extract than in the acute brain extract. Autophagy activation by rapamycin could rescue the neuronal differentiation of hNSCs within acute TBI brain extract. Conclusions: The subacute phase around 7 days after TBI in mice could be a candidate timepoint to encourage more neuronal differentiation after transplantation. The autophagy flux played a critical role in regulating neuronal differentiation of hNSCs and could serve as a potential target to improve the efficacy of transplantation in the early phase.

C1 [He, Zhenghui; Lang, Lijian; Hui, Jiyuan; Ma, Yuxiao; Yang, Chun; Jiang, Jiyao; Feng, Junfeng] Shanghai Jiao Tong Univ, Renji Hosp, Brain Injury Ctr, Sch Med,Dept Neurosurg, Shanghai 200127, Peoples R China.

[Huang, Jialin; Jiang, Jiyao; Feng, Junfeng] Shanghai Inst Head Trauma, Shanghai 200127, Peoples R China.

[Weng, Weiji] Shanghai Jiao Tong Univ, Sch Med, Dept Biochem & Mol Cell Biol, Shanghai Key Lab Tumor Microenvironm & Inflammat, Shanghai 200025, Peoples R China.

[Zhao, Xiongfei; Zhang, Xiaoqi] Shanghai Angecon Biotechnol Co Ltd, Shanghai 201318, Peoples R China.

[Liang, Qian] Univ Texas Southwestern Med Ctr Dallas, Dept Pathol, Dallas, TX 75390 USA.

C3 Shanghai Jiao Tong University; Shanghai Jiao Tong University; University

of Texas System; University of Texas Southwestern Medical Center Dallas

RP Feng, JF (通讯作者)，Shanghai Jiao Tong Univ, Renji Hosp, Brain Injury Ctr, Sch Med,Dept Neurosurg, Shanghai 200127, Peoples R China.; Feng, JF (通讯作者)，Shanghai Inst Head Trauma, Shanghai 200127, Peoples R China.

EM hezhenghui8@sjtu.edu.cn; langlijian@sjtu.edu.cn; 18424@renji.com;

mqsmyx@sjtu.edu.cn; yc_shsmu@sjtu.edu.cn; wengweiji@alumni.sjtu.edu.cn;

19220@renji.com; zhaox@angecon.com; zhangxq@angecon.com;

qian.liang@utsouthwestern.edu; jiyaojiang@126.com; fengjfmail@163.com

RI Ma, Yuxiao/GWC-7034-2022; Weng, Weiji/HLH-2707-2023

OI Feng, Jun-feng/0000-0002-3243-3842

FU National Natural Science Foundation of China [82071358]; Program of

Shanghai Academic Research Leader [21XD1422400]; Project of Shanghai

Medical and Health Development Foundation [20224Z0012]

FX This research was funded by The National Natural Science Foundation of

China, grant number 82071358, The Program of Shanghai Academic Research

Leader, grant number 21XD1422400, and Project of Shanghai Medical and

Health Development Foundation, grant number 20224Z0012.

CR Ashina H, 2021, LANCET NEUROL, V20, P460, DOI 10.1016/S1474-4422(21)00094-6

Bankston AN, 2019, GLIA, V67, P1745, DOI 10.1002/glia.23646

Beretta S, 2017, CELL TRANSPLANT, V26, P1247, DOI 10.1177/0963689717714107

Chudickova M, 2017, J TISSUE ENG REGEN M, V11, P1588, DOI 10.1002/term.2059

Dinet V, 2019, FRONT NEUROSCI-SWITZ, V13, DOI 10.3389/fnins.2019.01178

Duan HM, 2016, ACTA BIOMATER, V45, P182, DOI 10.1016/j.actbio.2016.08.043

Fan Q, 2018, J EXP CLIN CANC RES, V37, DOI 10.1186/s13046-018-0673-y

Fleming A, 2020, TRENDS NEUROSCI, V43, P767, DOI 10.1016/j.tins.2020.07.003

Frankowski JC, 2021, COMMUN BIOL, V4, DOI 10.1038/s42003-021-02808-5

Gao JL, 2006, EXP NEUROL, V201, P281, DOI 10.1016/j.expneurol.2006.04.039

Ha S, 2019, EXP NEUROBIOL, V28, P229, DOI 10.5607/en.2019.28.2.229

Hachem LD, 2016, STEM CELLS DEV, V25, P1223, DOI 10.1089/scd.2015.0389

Hu Z, 2020, J TRAUMA ACUTE CARE, V88, P477, DOI 10.1097/TA.0000000000002510

Hwang I, 2021, FREE RADICAL BIO MED, V169, P74, DOI 10.1016/j.freeradbiomed.2021.03.043

Ibrahim S, 2016, SCI REP-UK, V6, DOI 10.1038/srep21793

Ismail H, 2020, ANTIOXIDANTS-BASEL, V9, DOI 10.3390/antiox9100943

Jha RM, 2019, NEUROPHARMACOLOGY, V145, P230, DOI 10.1016/j.neuropharm.2018.08.004

Jiang JY, 2019, LANCET NEUROL, V18, P286, DOI 10.1016/S1474-4422(18)30469-1

Lee JY, 2019, THERANOSTICS, V9, P1029, DOI 10.7150/thno.29868

Levin HS, 2015, LANCET NEUROL, V14, P506, DOI 10.1016/S1474-4422(15)00002-2

Li CY, 2020, ANGEW CHEM INT EDIT, V59, P247, DOI 10.1002/anie.201911803

Li HY, 2019, STEM CELLS, V37, P504, DOI 10.1002/stem.2968

Li W, 2022, NEURAL REGEN RES, V17, P401, DOI 10.4103/1673-5374.317987

Li YM, 2021, ACS CHEM NEUROSCI, V12, P1363, DOI 10.1021/acschemneuro.1c00002

Littlejohn EL, 2020, ACTA NEUROPATHOL COM, V8, DOI 10.1186/s40478-020-00925-6

Liu SW, 2018, LIFE SCI, V209, P34, DOI 10.1016/j.lfs.2018.07.054

Liu XY, 2020, J NEUROCHEM, V153, P230, DOI 10.1111/jnc.14859

Nicklas S, 2019, CELL DEATH DIFFER, V26, P728, DOI 10.1038/s41418-018-0144-1

Romine J, 2014, JOVE-J VIS EXP, DOI 10.3791/51781

Rosenfeld JV, 2013, LANCET NEUROL, V12, P882, DOI 10.1016/S1474-4422(13)70161-3

Shi W, 2012, BIOMATERIALS, V33, P3119, DOI 10.1016/j.biomaterials.2012.01.009

Simon DW, 2017, NAT REV NEUROL, V13, P171, DOI 10.1038/nrneurol.2017.13

Tang JS, 2020, ACTA PHARMACOL SIN B, V10, P987, DOI 10.1016/j.apsb.2020.02.015

Turtzo LC, 2021, J NEUROTRAUM, V38, P3107, DOI 10.1089/neu.2021.0037

van Vliet EA, 2020, NEUROBIOL DIS, V145, DOI 10.1016/j.nbd.2020.105080

Weston NM, 2018, CURR NEUROL NEUROSCI, V18, DOI 10.1007/s11910-018-0812-z

Wu YX, 2020, FRONT PHYSIOL, V11, DOI 10.3389/fphys.2020.01030

Xiong LL, 2018, MOL NEUROBIOL, V55, P2696, DOI 10.1007/s12035-017-0551-1

Xiong Y, 2013, NAT REV NEUROSCI, V14, P128, DOI 10.1038/nrn3407

Zeng ZQ, 2020, J CELL PHYSIOL, V235, P1973, DOI 10.1002/jcp.29173

Zhao S, 2017, NEUROSCI LETT, V643, P125, DOI 10.1016/j.neulet.2016.12.006

Zhou CF, 2021, AUTOPHAGY, V17, P3175, DOI 10.1080/15548627.2020.1826689

Zou ZM, 2021, J NEUROINFLAMM, V18, DOI 10.1186/s12974-021-02192-1

NR 43

TC 0

Z9 0

U1 2

U2 6

PU MDPI

PI BASEL

PA ST ALBAN-ANLAGE 66, CH-4052 BASEL, SWITZERLAND

EI 2077-0383

J9 J CLIN MED

JI J. Clin. Med.

PD MAY

PY 2022

VL 11

IS 10

AR 2709

DI 10.3390/jcm11102709

PG 13

WC Medicine, General & Internal

WE Science Citation Index Expanded (SCI-EXPANDED)

SC General & Internal Medicine

GA 1P1WT

UT WOS:000801808500001

PM 35628836

OA Green Published, gold

DA 2023-06-10

ER

PT J

AU Lam, PK

Lo, AWI

Wang, KKW

Lau, HCH

Leung, KKC

Li, KTC

Lai, PBS

Poon, WS

AF Lam, Ping Kuen

Lo, Anthony Wing Ip

Wang, Kevin Ka Wan

Lau, Henry Chi Ho

Leung, Kevin Kai Chung

Li, Kathy Tung Ching

Lai, Paul Bo San

Poon, Wai Sang

TI Transplantation of mesenchymal stem cells to the brain by topical

application in an experimental traumatic brain injury model

SO JOURNAL OF CLINICAL NEUROSCIENCE

LA English

DT Article

DE Mesenchymal stem cell; Topical application; Traumatic brain injury

ID NEUROLOGICAL DISEASES; MIGRATION; THERAPY

AB Mesenchymal stem cells (MSCs) have been shown in various animal models to be capable of neurorepair and neuroprotection. To carry out a therapeutic function, MSCs must be delivered to the target organ. MSCs are administered to patients via systemic infusion, which has many drawbacks, including a low engraftment rate and the migration of MSCs to non-target organs. However, other approaches such as direct intracerebral injection of MSCs might cause cerebral bleeding. In this study, a traumatic brain injury (TBI) was induced over the right parietal cerebral cortex in Sprague Dawley rats, and green fluorescent protein (GFP)-expressing MSCs (GFP-MSCs), together with a thin layer of fibrin, were applied to the external surface of the contralateral side 2 days later. Within 5 days of topical application, the GFP-MSCs had migrated from the site of application on the cortical surface, through the white matter, and had emerged at the cortical surface of the TBI site on the contralateral cerebral hemisphere, apparently following axons along the corpus callosum. In sham-injured control animals, the topically applied GFP-MSCs proliferated superficially on the cortex at the site of application, and no GFP-MSCs were found at the contralateral cortical surface. In all instances, GFP-MSCs were not detected in other organs of either the test or the control animals. Our study demonstrated that MSCs topically applied to the brain surface can migrate to a TBI site. (C) 2012 Published by Elsevier Ltd.

C1 [Lam, Ping Kuen; Wang, Kevin Ka Wan; Lau, Henry Chi Ho; Leung, Kevin Kai Chung; Li, Kathy Tung Ching; Lai, Paul Bo San; Poon, Wai Sang] Chinese Univ Hong Kong, Prince Wales Hosp, Dept Surg, Shatin, Hong Kong, Peoples R China.

[Lam, Ping Kuen] Chinese Univ Hong Kong, Prince Wales Hosp, Chow Tai Fook Cheng Yu Tung Surg Stem Cell Res Ct, Shatin, Hong Kong, Peoples R China.

[Lo, Anthony Wing Ip] Chinese Univ Hong Kong, Prince Wales Hosp, Dept Anat & Cellular Pathol, Shatin, Hong Kong, Peoples R China.

[Wang, Kevin Ka Wan] Banyan Biomarkers, Ctr Innovat Res, Alachua, FL USA.

[Wang, Kevin Ka Wan] Univ Florida, Ctr Neuroprote & Biomarkers Res, Dept Psychiat & Neurosci, Gainesville, FL USA.

C3 Chinese University of Hong Kong; Prince of Wales Hospital; Chinese

University of Hong Kong; Prince of Wales Hospital; Chinese University of

Hong Kong; Prince of Wales Hospital; Banyan Biomarkers Inc.; State

University System of Florida; University of Florida

RP Poon, WS (通讯作者)，Chinese Univ Hong Kong, Prince Wales Hosp, Dept Surg, Shatin, Hong Kong, Peoples R China.

EM wpoon@surgery.cuhk.edu.hk

RI Lo, Anthony W. I./G-5414-2015; Poon, Wai Sang/F-1558-2011; Lai, Paul

BS/K-8556-2015; Poon, Wai S/HGD-3084-2022

OI Lo, Anthony W. I./0000-0002-9388-310X; Lai, Paul BS/0000-0002-9469-6728;

Wang, Kevin/0000-0002-9343-6473

CR Andriessen TMJC, 2010, J CELL MOL MED, V14, P2381, DOI 10.1111/j.1582-4934.2010.01164.x

Barone FC, 2006, CLIN NEUROSCI RES, V6, P329, DOI 10.1016/j.cnr.2006.09.010

Brody DL, 2007, J NEUROTRAUM, V24, P657, DOI 10.1089/neu.2006.0011

Gogel S, 2011, GENE THER, V18, P1, DOI 10.1038/gt.2010.130

Harting MT, 2009, J NEUROSURG, V110, P1189, DOI 10.3171/2008.9.JNS08158

Imitola J, 2004, P NATL ACAD SCI USA, V101, P18117, DOI 10.1073/pnas.0408258102

Jackson MR, 2001, AM J SURG, V182, p1S, DOI 10.1016/S0002-9610(01)00770-X

Ji JF, 2004, STEM CELLS, V22, P415, DOI 10.1634/stemcells.22-3-415

Kim SU, 2009, J NEUROSCI RES, V87, P2183, DOI 10.1002/jnr.22054

Lam PK, 2011, TRANSPLANTATION, V92, pE9, DOI 10.1097/TP.0b013e318221d3b7

Leon-Carrion Jose, 2005, Pituitary, V8, P197, DOI 10.1007/s11102-006-6041-5

Mendick R, BABY DEATH SCANDAL S

Poon WS, 2006, ACT NEUR S, V99, P123, DOI 10.1007/978-3-211-35205-2_23

Walczak P, 2008, STROKE, V39, P1569, DOI 10.1161/STROKEAHA.107.502047

Walker PA, 2009, DIS MODEL MECH, V2, P23, DOI 10.1242/dmm.001198

NR 15

TC 22

Z9 26

U1 0

U2 13

PU ELSEVIER SCI LTD

PI OXFORD

PA THE BOULEVARD, LANGFORD LANE, KIDLINGTON, OXFORD OX5 1GB, OXON, ENGLAND

SN 0967-5868

J9 J CLIN NEUROSCI

JI J. Clin. Neurosci.

PD FEB

PY 2013

VL 20

IS 2

BP 300

EP 303

DI 10.1016/j.jocn.2012.03.028

PG 4

WC Clinical Neurology; Neurosciences

WE Science Citation Index Expanded (SCI-EXPANDED)

SC Neurosciences & Neurology

GA 097LX

UT WOS:000315476700023

PM 23219830

DA 2023-06-10

ER

PT J

AU Adugna, DG

Aragie, H

Kibret, AA

Belay, DG

AF Adugna, Dagnew Getnet

Aragie, Hailu

Kibret, Anteneh Ayelign

Belay, Daniel Gashaneh

TI Therapeutic Application of Stem Cells in the Repair of Traumatic Brain

Injury

SO STEM CELLS AND CLONING-ADVANCES AND APPLICATIONS

LA English

DT Review

DE traumatic brain injury; stem cell therapy; neural stem cells; endogenous

neurogenesis; cell transplantation

ID CENTRAL-NERVOUS-SYSTEM; DENTATE GYRUS; STROMAL CELLS; FUNCTIONAL

RECOVERY; OLFACTORY-BULB; ENHANCED NEUROGENESIS; ENRICHED ENVIRONMENT;

ADULT NEUROGENESIS; COGNITIVE RECOVERY; ADRENAL-STEROIDS

AB Traumatic brain injury is the main cause of injury-related deaths and disabilities throughout the world, which is characterized by a disruption of the normal physiology of the brain following trauma. It can potentially cause severe complications such as physical, cognitive, and emotional impairment. In addition to understanding traumatic brain injury pathophysiology, this review explains the therapeutic potential of stem cells following brain injury in two pathways: response of endogenous neurogenic cells and transplantation of exogenous stem cell therapy. After traumatic brain injuries, clinical evidence indicated that endogenous neural progenitor cells might play an important role in regenerative medicine to treat brain injury. This is due to an increased neurogenic regeneration ability of these cells following brain injury. Besides, exogenous stem cell transplantation has also accelerated immature neuronal development and increased endogenous cellular proliferation in the damaged brain region. Therefore, a better understanding of the endogenous neural stem cell's regenerative ability and the effect of exogenous stem cells on proliferation and differentiation ability may help researchers to understand how to increase functional recovery and tissue repair following injury.

C1 [Adugna, Dagnew Getnet; Aragie, Hailu; Kibret, Anteneh Ayelign; Belay, Daniel Gashaneh] Univ Gondar, Coll Med & Hlth Sci, Sch Med, Dept Human Anat, Gondar, Emhara Region, Ethiopia.

[Belay, Daniel Gashaneh] Univ Gondar, Inst Publ Hlth, Coll Med & Hlth Sci, Dept Epidemiol, Gondar, Amhara Region, Ethiopia.

[Adugna, Dagnew Getnet] Univ Gondar, Coll Med & Hlth Sci, Sch Med, Dept Human Anat, POB 196, Gondar, Amhara Region, Ethiopia.

C3 University of Gondar; University of Gondar; University of Gondar

RP Adugna, DG (通讯作者)，Univ Gondar, Coll Med & Hlth Sci, Sch Med, Dept Human Anat, POB 196, Gondar, Amhara Region, Ethiopia.

EM dagnewgetnet5@gmail.com

OI Belay, Daniel Gashaneh/0000-0002-5724-6234; Kibret, Anteneh

Ayelign/0000-0002-4487-5015; Adugna, Dagnew Getnet/0000-0002-3670-4953;

Aragie, Hailu/0000-0002-0700-2952

CR Bambakidis T, 2022, SHOCK, V57, P281, DOI 10.1097/SHK.0000000000001889

Banasr M, 2001, EUR J NEUROSCI, V14, P1417, DOI 10.1046/j.0953-816x.2001.01763.x

Becerra GD, 2007, BEHAV BRAIN RES, V179, P118, DOI 10.1016/j.bbr.2007.01.024

Bergmann O, 2012, NEURON, V74, P634, DOI 10.1016/j.neuron.2012.03.030

Bhalala OG, 2012, J NEUROSCI, V32, P17935, DOI 10.1523/JNEUROSCI.3860-12.2012

Blaya MO, 2015, EXP NEUROL, V264, P67, DOI 10.1016/j.expneurol.2014.11.014

Bonilla C, 2009, BRAIN INJURY, V23, P760, DOI 10.1080/02699050903133970

Boockvar JA, 2005, NEUROSURGERY, V56, P163, DOI 10.1227/01.NEU.0000145866.25433.FF

Bramlett HM, 2004, J CEREBR BLOOD F MET, V24, P133, DOI 10.1097/01.WCB.0000111614.19196.04

Brown J, 2003, EUR J NEUROSCI, V17, P2042, DOI 10.1046/j.1460-9568.2003.02647.x

Bye N, 2011, J NEUROSCI RES, V89, P986, DOI 10.1002/jnr.22635

CAMERON HA, 1994, NEUROSCIENCE, V61, P203, DOI 10.1016/0306-4522(94)90224-0

Cameron HA, 2001, J COMP NEUROL, V435, P406, DOI 10.1002/cne.1040

Chen C, 2022, BRAIN RES, V1775, DOI 10.1016/j.brainres.2021.147711

Chirumamilla S, 2002, J NEUROTRAUM, V19, P693, DOI 10.1089/08977150260139084

Meirelles LDS, 2006, J CELL SCI, V119, P2204, DOI 10.1242/jcs.02932

Das M, 2019, REV NEUROSCIENCE, V30, P839, DOI 10.1515/revneuro-2019-0002

Dash PK, 2001, J NEUROSCI RES, V63, P313, DOI 10.1002/1097-4547(20010215)63:4<313::AID-JNR1025>3.3.CO;2-W

Djouad F, 2003, BLOOD, V102, P3837, DOI 10.1182/blood-2003-04-1193

Dunkerson J, 2014, RESTOR NEUROL NEUROS, V32, P675, DOI 10.3233/RNN-140408

Emery DL, 2005, J NEUROTRAUM, V22, P978, DOI 10.1089/neu.2005.22.978

Eriksson PS, 1998, NAT MED, V4, P1313, DOI 10.1038/3305

Faigle R, 2013, BBA-GEN SUBJECTS, V1830, P2435, DOI 10.1016/j.bbagen.2012.09.002

Gage FH, 1998, J NEUROBIOL, V36, P249, DOI 10.1002/(SICI)1097-4695(199808)36:2<249::AID-NEU11>3.0.CO;2-9

Gage FH, 2013, NEURON, V80, P588, DOI 10.1016/j.neuron.2013.10.037

Gao JL, 2007, EXP NEUROL, V204, P490, DOI 10.1016/j.expneurol.2006.10.001

Gao X, 2009, EXP NEUROL, V219, P516, DOI 10.1016/j.expneurol.2009.07.007

Gould E, 1997, DEV BRAIN RES, V103, P91, DOI 10.1016/S0165-3806(97)00079-5

Gould E, 1999, BIOL PSYCHIAT, V46, P1472, DOI 10.1016/S0006-3223(99)00247-4

Gritti A, 2002, J NEUROSCI, V22, P437, DOI 10.1523/JNEUROSCI.22-02-00437.2002

Hentze H, 2007, TRENDS BIOTECHNOL, V25, P24, DOI 10.1016/j.tibtech.2006.10.010

Hyder AA, 2007, NEUROREHABILITATION, V22, P341

Imayoshi I, 2008, NAT NEUROSCI, V11, P1153, DOI 10.1038/nn.2185

Jorge RE, 2004, ARCH GEN PSYCHIAT, V61, P42, DOI 10.1001/archpsyc.61.1.42

Joseph C, 2012, J CEREBR BLOOD F MET, V32, P1632, DOI 10.1038/jcbfm.2012.65

Kempermann G, 1997, P NATL ACAD SCI USA, V94, P10409, DOI 10.1073/pnas.94.19.10409

Kempermann G, 2000, NOVART FDN SYMP, V231, P220, DOI 10.1002/0470870834.ch14

Kempermann G, 2000, Prog Brain Res, V127, P35

Kempermann G, 1998, CURR BIOL, V8, P939, DOI 10.1016/S0960-9822(07)00377-6

Kim M, 2022, CELLS-BASEL, V11, DOI 10.3390/cells11050892

Lee ST, 2008, BRAIN, V131, P616, DOI 10.1093/brain/awm306

Li Y, 2009, NEUROSCI LETT, V456, P120, DOI 10.1016/j.neulet.2008.03.096

LOIS C, 1993, P NATL ACAD SCI USA, V90, P2074, DOI 10.1073/pnas.90.5.2074

Ponte AL, 2007, STEM CELLS, V25, P1737, DOI 10.1634/stemcells.2007-0054

Lu D, 2001, NEUROREPORT, V12, P559, DOI 10.1097/00001756-200103050-00025

Ma HY, 2012, NEUROCHEM RES, V37, P69, DOI 10.1007/s11064-011-0584-1

Maas AIR, 2008, LANCET NEUROL, V7, P728, DOI 10.1016/S1474-4422(08)70164-9

Mahmood A, 2003, NEUROSURGERY, V53, P697, DOI 10.1227/01.NEU.0000079333.61863.AA

Mahmood A, 2001, J NEUROSURG, V94, P589, DOI 10.3171/jns.2001.94.4.0589

Meirelles LD, 2009, CYTOKINE GROWTH F R, V20, P419, DOI 10.1016/j.cytogfr.2009.10.002

Moreno MM, 2009, P NATL ACAD SCI USA, V106, P17980, DOI 10.1073/pnas.0907063106

Muhammad SA, MOL NEUROBIOL, V2022, P1, DOI [10.1007/s12035-021-02552-1, DOI 10.1007/S12035-021-02552-1]

Narouiepour A, 2022, SCI REP-UK, V12, DOI 10.1038/s41598-022-07367-1

Ortega F, 2013, NAT CELL BIOL, V15, P602, DOI 10.1038/ncb2736

Parent JM, 2002, ANN NEUROL, V52, P802, DOI 10.1002/ana.10393

Park D, 2012, CELL TRANSPLANT, V21, P365, DOI 10.3727/096368911X586765

Petcu EB, 2018, BIOFABRICATION, V10, DOI 10.1088/1758-5090/aaaf50

Popa-Wagner A, 2014, FRONT CELL NEUROSCI, V8, DOI 10.3389/fncel.2014.00347

Viet QHN, 2022, NEUROL SCI, V43, P2157, DOI 10.1007/s10072-021-05529-z

Reis C, 2017, STEM CELLS INT, V2017, DOI 10.1155/2017/6392592

Rice AC, 2003, EXP NEUROL, V183, P406, DOI 10.1016/S0014-4886(03)00241-3

Riess P, 2002, NEUROSURGERY, V51, P1043, DOI 10.1097/00006123-200210000-00035

Sanai N, 2004, NATURE, V427, P740, DOI 10.1038/nature02301

Sanai N, 2011, NATURE, V478, P382, DOI 10.1038/nature10487

Sanchez-Ramos J, 2000, EXP NEUROL, V164, P247, DOI 10.1006/exnr.2000.7389

Seth AK, 2015, J NEUROSCI, V35, P3293, DOI 10.1523/JNEUROSCI.4399-14.2015

Shear DA, 2004, BRAIN RES, V1026, P11, DOI 10.1016/j.brainres.2004.07.087

Sun D, 2007, EXP NEUROL, V204, P264, DOI 10.1016/j.expneurol.2006.11.005

Sun D, 2016, EXP NEUROL, V275, P405, DOI 10.1016/j.expneurol.2015.04.017

Sun D, 2015, J NEUROTRAUM, V32, P495, DOI 10.1089/neu.2014.3545

Sun D, 2011, J NEUROTRAUM, V28, P961, DOI 10.1089/neu.2010.1697

Sun D, 2009, EXP NEUROL, V216, P56, DOI 10.1016/j.expneurol.2008.11.011

Tajiri N, 2013, PLOS ONE, V8, DOI 10.1371/journal.pone.0074857

Tang HL, 2013, CELL REPROGRAM, V15, P435, DOI 10.1089/cell.2012.0081

Taylor CA, 2017, MMWR SURVEILL SUMM, V66, P1, DOI 10.15585/mmwr.ss6609a1

van Praag H, 1999, P NATL ACAD SCI USA, V96, P13427, DOI 10.1073/pnas.96.23.13427

van Praag H, 2002, NATURE, V415, P1030, DOI 10.1038/4151030a

Vickers NJ, 2017, CURR BIOL, V27, pR713, DOI 10.1016/j.cub.2017.05.064

Walker PA, 2009, STEM CELL REV REP, V5, P283, DOI 10.1007/s12015-009-9081-1

Wang G, 2022, NEURAL REGEN RES, V17, P354, DOI 10.4103/1673-5374.317985

Wang SY, 2013, INT J DEV NEUROSCI, V31, P30, DOI 10.1016/j.ijdevneu.2012.09.004

Wennersten A, 2004, J NEUROSURG, V100, P88, DOI 10.3171/jns.2004.100.1.0088

Weston NM, 2018, CURR NEUROL NEUROSCI, V18, DOI 10.1007/s11910-018-0812-z

Xiong Y, 2008, BRAIN RES, V1230, P247, DOI 10.1016/j.brainres.2008.06.127

Yuan JY, 2020, STEM CELL REV REP, V16, P323, DOI 10.1007/s12015-019-09927-x

Zhang R, 2013, J NEUROINFLAMM, V10, DOI 10.1186/1742-2094-10-106

Zhang YL, 2017, NEUROCHEM INT, V111, P69, DOI 10.1016/j.neuint.2016.08.003

Zhang YC, 2022, INT J MOL SCI, V23, DOI 10.3390/ijms23052550

Zhang ZX, 2008, CYTOTHERAPY, V10, P134, DOI 10.1080/14653240701883061

Zhang ZW, 2022, OPEN LIFE SCI, V17, P189, DOI 10.1515/biol-2022-0022

NR 90

TC 4

Z9 4

U1 3

U2 7

PU DOVE MEDICAL PRESS LTD

PI ALBANY

PA PO BOX 300-008, ALBANY, AUCKLAND 0752, NEW ZEALAND

SN 1178-6957

J9 STEM CELLS CLONING

JI Stem Cells Cloning

PY 2022

VL 15

BP 53

EP 61

DI 10.2147/SCCAA.S369577

PG 9

WC Cell Biology

WE Emerging Sources Citation Index (ESCI)

SC Cell Biology

GA 3F0UQ

UT WOS:000830389600001

PM 35859889

OA Green Published, gold

DA 2023-06-10

ER

PT J

AU Harting, MT

Jimenez, F

Xue, H

Fischer, UM

Baumgartner, J

Dash, PK

Cox, CS

AF Harting, Matthew T.

Jimenez, Fernando

Xue, Hasan

Fischer, Uwe M.

Baumgartner, James

Dash, Pramod K.

Cox, Charles S., Jr.

TI Intravenous mesenchymal stem cell therapy for traumatic brain injury

Laboratory investigation

SO JOURNAL OF NEUROSURGERY

LA English

DT Article

DE adult stem cell; cellular therapy; in vivo tracking; mesenchymal stem

cell; traumatic brain injury

ID CORD BLOOD-CELLS; PROGENITOR-CELLS; MARROW-CELLS; TRANSPLANTATION;

RECOVERY; DELIVERY; RATS; OSTEOGENESIS; DIFFERENTIATE; EXPRESSION

AB Object. Cell therapy has shown preclinical promise in the treatment of many diseases, and its application is being translated to the clinical arena. Intravenous mesenchymal stein cell (MSC) therapy has been shown to improve functional recovery after traumatic brain injury (TBI). Herein, the authors report on their attempts to reproduce such observations, including detailed characterizations of the MSC Population, non-bromodeoxyuridine-based cell labeling, macroscopic and microscopic cell tracking, quantification of cells traversing the pulmonary microvasculature, and well-validated measurement of motor and cognitive function recovery.

Methods. Rat MSCs were isolated, expanded in vitro, immunophenotyped, and labeled. Four million MSCs were intravenously infused into Sprague-Dawley rats 24 hours after receiving a moderate, unilateral controlled cortical impact TBI. Infrared macroscopic cell tracking was used to identify cell distribution. Immunohistochemical analysis of brain and lung tissues 48 hours and 2 weeks postinfusion revealed transplanted cells in these locations, and these cells were quantified. Intraarterial blood sampling and flow cytometry were used to quantity the number of transplanted cells reaching the arterial circulation. Motor and cognitive behavioral testing was performed to evaluate functional recovery.

Results. At 48 hours post-MSC infusion, the majority of cells were localized to the lungs. Between 1.5 and 3.7% of the infused cells were estimated to traverse the lungs and reach the arterial circulation, 0.295% reached the carotid artery, and a very small percentage reached the cerebral parenchyma (0.0005%) and remained there. Almost no cells were identified in the brain tissue at 2 weeks postinfusion. No motor or cognitive functional improvements in recovery were identified.

Conclusions. The intravenous infusion of MSCs appeared neither to result in significant acute or prolonged cerebral engraftment of cells nor to modify the recovery of motor or cognitive function. Less than 4% of the infused cells were likely to traverse the Pulmonary microvasculature and reach the arterial circulation, a phenomenon termed the "pulmonary first-pass effect," which may limit the efficacy of this therapeutic approach. The data in this study contradict the findings of previous reports and highlight the potential shortcomings of acute, single-dose, intravenous MSC therapy for TBI. (DOI: 10.3171/2008.9.JNS08158)

C1 [Harting, Matthew T.; Jimenez, Fernando; Xue, Hasan; Fischer, Uwe M.; Baumgartner, James; Cox, Charles S., Jr.] Univ Texas Houston, Sch Med, Dept Pediat Surg, Houston, TX 77030 USA.

[Dash, Pramod K.] Univ Texas Houston, Sch Med, Dept Neurobiol & Anat, Houston, TX 77030 USA.

[Dash, Pramod K.] Univ Texas Houston, Sch Med, Vivian L Smith Ctr Neurol Res, Houston, TX 77030 USA.

C3 University of Texas System; University of Texas Health Science Center

Houston; University of Texas System; University of Texas Health Science

Center Houston; University of Texas System; University of Texas Health

Science Center Houston

RP Cox, CS (通讯作者)，Univ Texas Houston, Sch Med, Dept Pediat Surg, 6431 Fannin St,MSB 5-254, Houston, TX 77030 USA.

EM charles.s.cox@uth.tmc.edu

RI Dash, Pramod Kumar/F-5832-2011

OI Dash, Pramod Kumar/0000-0001-6746-1002; Harting,

Matthew/0000-0002-8929-8311

FU National Institutes of Health [T32 GM008792-06, MO1 RR 02558, R21 HD 04

2659-01A1]; Children's Memorial Hermann Hospital Foundation; Texas

Higher Education Coordinating Board

FX This work was supported by National Institutes of Health Grant Nos. T32

GM008792-06 (M.T.H.), MO1 RR 02558 (C.S.C.), and R21 HD 04 2659-01A1

(C.S.C.), as well as funds from the Children's Memorial Hermann Hospital

Foundation (C.S.C.) and Texas Higher Education Coordinating Board

(C.S.C.).

CR Badillo AT, 2007, BIOL BLOOD MARROW TR, V13, P412, DOI 10.1016/j.bbmt.2006.12.447

Barbash IM, 2003, CIRCULATION, V108, P863, DOI 10.1161/01.CIR.0000084828.50310.6A

Bentzon JF, 2005, BIOCHEM BIOPH RES CO, V330, P633, DOI 10.1016/j.bbrc.2005.03.072

Breyer A, 2006, EXP HEMATOL, V34, P1596, DOI 10.1016/j.exphem.2006.07.013

Burns TC, 2006, STEM CELLS, V24, P1121, DOI 10.1634/stemcells.2005-0463

Castro RF, 2002, SCIENCE, V297, P1299, DOI 10.1126/science.297.5585.1299

Chamberlain JR, 2004, SCIENCE, V303, P1198, DOI 10.1126/science.1088757

English D, 2006, EXP NEUROL, V199, P10, DOI 10.1016/j.expneurol.2006.03.005

FANG JM, 1995, INT J DEV BIOL, V39, P519

Freyman T, 2006, EUR HEART J, V27, P1114, DOI 10.1093/eurheartj/ehi818

Fujimoto ST, 2004, NEUROSCI BIOBEHAV R, V28, P365, DOI 10.1016/j.neubiorev.2004.06.002

Garbuzova-Davis S, 2003, J HEMATOTH STEM CELL, V12, P255, DOI 10.1089/152581603322022990

Guzowski JF, 1997, P NATL ACAD SCI USA, V94, P2693, DOI 10.1073/pnas.94.6.2693

Horwitz EM, 1999, NAT MED, V5, P309, DOI 10.1038/6529

Khakoo AY, 2006, J EXP MED, V203, P1235, DOI 10.1084/jem.20051921

Klass M, 2007, ANESTH ANALG, V104, P944, DOI 10.1213/01.ane.0000258021.03211.d0

Koc ON, 2000, J CLIN ONCOL, V18, P307, DOI 10.1200/JCO.2000.18.2.307

Kong KY, 2004, STEM CELLS, V22, P981, DOI 10.1634/stemcells.22-6-981

Krause U, 2007, STEM CELLS DEV, V16, P31, DOI 10.1089/scd.2006.0089

Lagasse E, 2000, NAT MED, V6, P1229, DOI 10.1038/81326

LAZARUS HM, 1995, BONE MARROW TRANSPL, V16, P557

Lighthall JW, 1988, J NEUROTRAUM, V5, P1, DOI 10.1089/neu.1988.5.1

Lorenzini S, 2007, STEM CELLS, V25, P2383, DOI 10.1634/stemcells.2007-0056

Losordo DW, 2004, CIRCULATION, V109, P2692, DOI 10.1161/01.CIR.0000128596.49339.05

Lu D, 2001, NEUROREPORT, V12, P559, DOI 10.1097/00001756-200103050-00025

Magliocca JF, 2006, STEM CELLS DEV, V15, P707, DOI 10.1089/scd.2006.15.707

Mahmood A, 2006, J NEUROSURG, V104, P272, DOI 10.3171/jns.2006.104.2.272

Mahmood A, 2005, NEUROSURGERY, V57, P1026, DOI 10.1227/01.NEU.0000181369.76323.50

Mahmood A, 2004, NEUROSURGERY, V55, P1185, DOI 10.1227/01.NEU.0000141042.14476.3C

Mahmood A, 2004, J NEUROTRAUM, V21, P33, DOI 10.1089/089771504772695922

Mahmood A, 2003, NEUROSURGERY, V53, P697, DOI 10.1227/01.NEU.0000079333.61863.AA

Mahmood A, 2001, NEUROSURGERY, V49, P1196, DOI 10.1097/00006123-200111000-00031

Mezey E, 2003, SCIENCE, V299

Perin EC, 2003, CIRCULATION, V107, P2294, DOI 10.1161/01.CIR.0000070596.30552.8B

Priddle H, 2006, STEM CELLS, V24, P815, DOI 10.1634/stemcells.2005-0356

SCHENK F, 1985, EXP BRAIN RES, V58, P11

Schrepfer S, 2007, TRANSPL P, V39, P573, DOI 10.1016/j.transproceed.2006.12.019

Shindo T, 2006, J MED INVESTIG, V53, P42, DOI 10.2152/jmi.53.42

Song L, 2004, FASEB J, V18, P980, DOI 10.1096/fj.03-1100fje

Srivastava D, 2006, NATURE, V441, P1097, DOI 10.1038/nature04961

Theise ND, 2003, SCIENCE, V299, p1317A, DOI 10.1126/science.1078412

Tolar J, 2006, BLOOD, V107, P4182, DOI 10.1182/blood-2005-08-3289

Ukai R, 2007, J NEUROTRAUM, V24, P508, DOI 10.1089/neu.2006.0161

Wagers AJ, 2002, SCIENCE, V297, P2256, DOI 10.1126/science.1074807

Wolf D, 2007, J AM SOC ECHOCARDIOG, V20, P512, DOI 10.1016/j.echo.2006.11.008

Xin H, 2007, STEM CELLS, V25, P1618, DOI 10.1634/stemcells.2006-0461

Xu JG, 2007, AM J PHYSIOL-LUNG C, V293, pL131, DOI 10.1152/ajplung.00431.2006

Ziv Y, 2006, P NATL ACAD SCI USA, V103, P13174, DOI 10.1073/pnas.0603747103

NR 48

TC 199

Z9 211

U1 0

U2 13

PU AMER ASSOC NEUROLOGICAL SURGEONS

PI ROLLING MEADOWS

PA 5550 MEADOWBROOK DRIVE, ROLLING MEADOWS, IL 60008 USA

SN 0022-3085

EI 1933-0693

J9 J NEUROSURG

JI J. Neurosurg.

PD JUN

PY 2009

VL 110

IS 6

BP 1189

EP 1197

DI 10.3171/2008.9.JNS08158

PG 9

WC Clinical Neurology; Surgery

WE Science Citation Index Expanded (SCI-EXPANDED)

SC Neurosciences & Neurology; Surgery

GA 451HH

UT WOS:000266460800012

PM 19301973

OA Green Accepted

DA 2023-06-10

ER

PT J

AU Darkazalli, A

Vied, C

Badger, CD

Levenson, CW

AF Darkazalli, Ali

Vied, Cynthia

Badger, Crystal-Dawn

Levenson, Cathy W.

TI Human Mesenchymal Stem Cell Treatment Normalizes Cortical Gene

Expression after Traumatic Brain Injury

SO JOURNAL OF NEUROTRAUMA

LA English

DT Article

DE controlled cortical impact; genomics; stem cells; traumatic brain injury

ID ALZHEIMERS-DISEASE; HEAD-INJURY; BIOCONDUCTOR PACKAGE; FUNCTIONAL

RECOVERY; RATS; MECHANISMS; RECEPTORS; RISK; DIFFERENTIATION;

TRANSPLANTATION

AB Traumatic brain injury (TBI) results in a progressive disease state with many adverse and long-term neurological consequences. Mesenchymal stem cells (MSCs) have emerged as a promising cytotherapy and have been previously shown to reduce secondary apoptosis and cognitive deficits associated with TBI. Consistent with the established literature, we observed that systemically administered human MSCs (hMSCs) accumulate with high specificity at the TBI lesion boundary zone known as the penumbra. Substantial work has been done to illuminate the mechanisms by which MSCs, and the bioactive molecules they secrete, exert their therapeutic effect. However, no such work has been published to examine the effect of MSC treatment on gene expression in the brain post-TBI. In the present study, we use high-throughput RNA sequencing (RNAseq) of cortical tissue from the TBI penumbra to assess the molecular effects of both TBI and subsequent treatment with intravenously delivered hMSCs. RNAseq revealed that expression of almost 7000 cortical genes in the penumbra were differentially regulated by TBI. Pathway analysis using the KEGG (Kyoto Encyclopedia of Genes and Genomes) pathway database revealed that TBI regulated a large number of genes belonging to pathways involved in metabolism, receptor-mediated cell signaling, neuronal plasticity, immune cell recruitment and infiltration, and neurodegenerative disease. Remarkably, hMSC treatment was found to normalize 49% of all genes disrupted by TBI, with notably robust normalization of specific pathways within the categories mentioned above, including neuroactive receptor-ligand interactions (57%), glycolysis and gluconeogenesis (81%), and Parkinson's disease (100%). These data provide evidence in support of the multi-mechanistic nature of stem cell therapy and suggest that hMSC treatment is capable of simultaneously normalizing a wide variety of important molecular pathways that are disrupted by brain injury.

C1 [Darkazalli, Ali; Vied, Cynthia; Badger, Crystal-Dawn; Levenson, Cathy W.] Florida State Univ, Coll Med, Dept Biomed Sci, Coll Med Res Bldg,MSR 2350-E,1115 West Call St, Tallahassee, FL 32306 USA.

[Darkazalli, Ali; Levenson, Cathy W.] Florida State Univ, Coll Med, Program Neurosci, Coll Med Res Bldg,MSR 2350-E,1115 West Call St, Tallahassee, FL 32306 USA.

C3 State University System of Florida; Florida State University; State

University System of Florida; Florida State University

RP Levenson, CW (通讯作者)，Florida State Univ, Coll Med, Dept Biomed Sci, Coll Med Res Bldg,MSR 2350-E,1115 West Call St, Tallahassee, FL 32306 USA.; Levenson, CW (通讯作者)，Florida State Univ, Coll Med, Program Neurosci, Coll Med Res Bldg,MSR 2350-E,1115 West Call St, Tallahassee, FL 32306 USA.

EM cathy.levenson@med.fsu.edu

OI Levenson, Cathy W/0000-0003-4463-3136; Vied, Cynthia/0000-0003-3941-3614

CR Arundine M, 2004, CELL MOL LIFE SCI, V61, P657, DOI 10.1007/s00018-003-3319-x

Ben Borgens R, 2012, Q REV BIOL, V87, P89, DOI 10.1086/665457

BENJAMINI Y, 1995, J R STAT SOC B, V57, P289, DOI 10.1111/j.2517-6161.1995.tb02031.x

Biervert C, 2001, NEUROSCI LETT, V315, P25, DOI 10.1016/S0304-3940(01)02312-6

Bolger AM, 2014, BIOINFORMATICS, V30, P2114, DOI 10.1093/bioinformatics/btu170

Caplan AI, 2011, CELL STEM CELL, V9, P11, DOI 10.1016/j.stem.2011.06.008

Chang CP, 2013, CLIN SCI, V124, P165, DOI 10.1042/CS20120226

Chang PL, 2011, BMC GENOMICS, V12, DOI 10.1186/1471-2164-12-364

Meirelles LDS, 2006, J CELL SCI, V119, P2204, DOI 10.1242/jcs.02932

Delhomme N, 2012, BIOINFORMATICS, V28, P2532, DOI 10.1093/bioinformatics/bts477

Di Nicola M, 2002, BLOOD, V99, P3838, DOI 10.1182/blood.V99.10.3838

Du J, 2007, CURR PHARM DESIGN, V13, P2507

Gadina M, 2001, CURR OPIN IMMUNOL, V13, P363, DOI 10.1016/S0952-7915(00)00228-4

Gardner RC, 2015, ANN NEUROL, V77, P987, DOI 10.1002/ana.24396

Goldman SM, 2006, ANN NEUROL, V60, P65, DOI 10.1002/ana.20882

Goldshmit Y, 2006, BRAIN RES REV, V52, P327, DOI 10.1016/j.brainresrev.2006.04.006

Guan J, 2013, BIOMATERIALS, V34, P5937, DOI 10.1016/j.biomaterials.2013.04.047

Hung SC, 2007, STEM CELLS, V25, P2363, DOI 10.1634/stemcells.2006-0686

KATAYAMA Y, 1990, J NEUROSURG, V73, P889, DOI 10.3171/jns.1990.73.6.0889

Kean TJ, 2013, STEM CELLS INT, V2013, DOI 10.1155/2013/732742

Kim HJ, 2010, J NEUROTRAUM, V27, P131, DOI [10.1089/neu.2008.0818, 10.1089/neu.2008-0818]

Kinnaird T, 2004, CIRC RES, V94, P678, DOI 10.1161/01.RES.0000118601.37875.AC

Lambert C, 2016, MOL NEUROBIOL, V53, P2297, DOI 10.1007/s12035-015-9138-x

Lanza C, 2009, J NEUROCHEM, V110, P1674, DOI 10.1111/j.1471-4159.2009.06268.x

Li H, 2009, BIOINFORMATICS, V25, P1754, DOI 10.1093/bioinformatics/btp324

Lin MT, 2006, NATURE, V443, P787, DOI 10.1038/nature05292

Love MI, 2014, GENOME BIOL, V15, DOI 10.1186/s13059-014-0550-8

Lu D, 2001, NEUROREPORT, V12, P559, DOI 10.1097/00001756-200103050-00025

Mahmood A, 2003, NEUROSURGERY, V53, P697, DOI 10.1227/01.NEU.0000079333.61863.AA

Mahmood A, 2001, NEUROSURGERY, V49, P1196, DOI 10.1097/00006123-200111000-00031

Meirelles LD, 2009, CYTOKINE GROWTH F R, V20, P419, DOI 10.1016/j.cytogfr.2009.10.002

Menge T, 2012, SCI TRANSL MED, V4, DOI 10.1126/scitranslmed.3004660

Moreno-Flores MT, 1999, NEUROSCIENCE, V91, P193, DOI 10.1016/S0306-4522(98)00568-5

Mortazavi A, 2008, NAT METHODS, V5, P621, DOI 10.1038/nmeth.1226

Muroski ME, 2014, J AM CHEM SOC, V136, P14763, DOI 10.1021/ja505190q

Natale JE, 2003, J NEUROTRAUM, V20, P907, DOI 10.1089/089771503770195777

Nauta AJ, 2008, LIBRARY, V110, P3499, DOI DOI 10.1182/BL00D-2007-02-069716

Nguyen TP, 2015, J LEUKOCYTE BIOL, V97, P1139, DOI 10.1189/jlb.4A0714-345RR

Nunomura A, 2001, J NEUROPATH EXP NEUR, V60, P759, DOI 10.1093/jnen/60.8.759

Peterson TC, 2015, J NEUROTRAUM, V32, P765, DOI 10.1089/neu.2014.3530

Plassman BL, 2000, NEUROLOGY, V55, P1158, DOI 10.1212/WNL.55.8.1158

Pratico D, 2001, J NEUROSCI, V21, P4183, DOI 10.1523/JNEUROSCI.21-12-04183.2001

Redell JB, 2013, J NEUROTRAUM, V30, P752, DOI 10.1089/neu.2012.2437

Robinson MD, 2010, BIOINFORMATICS, V26, P139, DOI 10.1093/bioinformatics/btp616

Rosso SB, 2013, FRONT CELL NEUROSCI, V7, DOI 10.3389/fncel.2013.00103

Schofield PW, 1997, J NEUROL NEUROSUR PS, V62, P119, DOI 10.1136/jnnp.62.2.119

Sotiropoulou PA, 2006, STEM CELLS, V24, P74, DOI 10.1634/stemcells.2004-0359

Trapnell C, 2010, NAT BIOTECHNOL, V28, P511, DOI 10.1038/nbt.1621

Trapnell C, 2009, BIOINFORMATICS, V25, P1105, DOI 10.1093/bioinformatics/btp120

Urban ETR, 2012, MOL CELL BIOCHEM, V369, P267, DOI 10.1007/s11010-012-1390-z

Varela-Nallar L, 2010, P NATL ACAD SCI USA, V107, P21164, DOI 10.1073/pnas.1010011107

Velliquette RA, 2005, J NEUROSCI, V25, P10874, DOI 10.1523/JNEUROSCI.2350-05.2005

Vonder Haar C, 2014, J NEUROTRAUM, V31, P961, DOI 10.1089/neu.2013.3119

Wang J, 2013, NUCLEIC ACIDS RES, V41, pW77, DOI 10.1093/nar/gkt439

Washington PM, 2014, J NEUROTRAUM, V31, P125, DOI 10.1089/neu.2013.3017

Wilkins A, 2009, STEM CELL RES, V3, P63, DOI 10.1016/j.scr.2009.02.006

Wu YJ, 2007, STEM CELLS, V25, P2648, DOI 10.1634/stemcells.2007-0226

Yi JH, 2006, NEUROCHEM INT, V48, P394, DOI 10.1016/j.neuint.2005.12.001

Zhang B, 2005, NUCLEIC ACIDS RES, V33, pW741, DOI 10.1093/nar/gki475

Zhang R, 2013, J NEUROINFLAMM, V10, DOI 10.1186/1742-2094-10-106

Zhang XY, 2014, GENET MOL RES, V13, P9220, DOI 10.4238/2014.November.7.9

Zou YM, 2004, TRENDS NEUROSCI, V27, P528, DOI 10.1016/j.tins.2004.06.015

NR 62

TC 10

Z9 10

U1 0

U2 25

PU MARY ANN LIEBERT, INC

PI NEW ROCHELLE

PA 140 HUGUENOT STREET, 3RD FL, NEW ROCHELLE, NY 10801 USA

SN 0897-7151

EI 1557-9042

J9 J NEUROTRAUM

JI J. Neurotrauma

PD JAN 1

PY 2017

VL 34

IS 1

BP 204

EP 212

DI 10.1089/neu.2015.4322

PG 9

WC Critical Care Medicine; Clinical Neurology; Neurosciences

WE Science Citation Index Expanded (SCI-EXPANDED)

SC General & Internal Medicine; Neurosciences & Neurology

GA EF6DE

UT WOS:000390420100022

PM 27161121

DA 2023-06-10

ER

PT J

AU Mazur, RA

Yokosawa, R

VandeVord, PJ

Lampe, KJ

AF Mazur, Rachel A.

Yokosawa, Ryosuke

VandeVord, Pamela J.

Lampe, Kyle J.

TI The need for tissue-engineered models to facilitate the study of

oligodendrocyte progenitor cells in traumatic brain injury and repair

SO CURRENT OPINION IN BIOMEDICAL ENGINEERING

LA English

DT Article

DE Oligodendrocyte progenitor cells; Traumatic brain injury; Tissue

engi-neering; Biomaterials

ID HYDROGELS; CNS

AB Traumatic brain injury (TBI) is an important public health issue as these high-rate mechanical insults to brain tissue, even on a mild scale, progress to secondary injury cascades that prolong and augment the injury. More effective models are vital to study the injury mechanism and progression. Although oligodendrocyte progenitor cells (OPCs) and mature oligodendrocytes (OLs) have been shown to play a large role in TBI injury and recovery, research into OPC/OL effects post-injury has previously been ignored in favor of neuronal research. Optimizing a hydrogel-based in vitro model system to elucidate the unique contributions of OPC/OL to the injury cascade would elevate molecular knowledge in TBI research. In this manuscript, we identify several key parameters and potential next steps for consideration in the development of such models.

C1 [Mazur, Rachel A.; Lampe, Kyle J.] Univ Virginia, Dept Chem Engn, Charlottesville, VA USA.

[Yokosawa, Ryosuke; VandeVord, Pamela J.] Virginia Polytech Inst & State Univ, Dept Biomed Engn & Mech, Blacksburg, VA 24061 USA.

C3 University of Virginia; Virginia Polytechnic Institute & State

University

RP Lampe, KJ (通讯作者)，Univ Virginia, Dept Chem Engn, Charlottesville, VA USA.

EM lampe@virginia.edu

OI Lampe, Kyle/0000-0003-1202-7609

FU National Science Foundation [2104723]; University of Virginia Dean's

Scholar Fellowship award (RAM); Direct For Mathematical & Physical

Scien; Division Of Materials Research [2104723] Funding Source: National

Science Foundation

FX This work was supported by the National Science Foundation [grant number

2104723] (KJL, PJV), as well as the University of Virginia Dean's

Scholar Fellowship award (RAM). Funding sources were not involved in

study design, data collection or analysis, in the writing of the report,

or in the decision to submit the article for publication.

CR Abu-Rub M, 2018, BRAIN SCI, V8, DOI 10.3390/brainsci8060111

Aguado BA, 2012, TISSUE ENG PT A, V18, P806, DOI [10.1089/ten.tea.2011.0391, 10.1089/ten.TEA.2011.0391]

Anderson J, 2020, EXP NEUROL, V325, DOI 10.1016/j.expneurol.2019.113119

Assinck P, 2017, J NEUROSCI, V37, P8635, DOI 10.1523/JNEUROSCI.2409-16.2017

Baaklini CS, 2019, FRONT MOL NEUROSCI, V12, DOI 10.3389/fnmol.2019.00225

Bradshaw DV, 2021, NEUROTRAUMA REP, V2, P180, DOI 10.1089/neur.2020.0058

Braun M, 2017, BBA-MOL BASIS DIS, V1863, P2614, DOI 10.1016/j.bbadis.2017.05.020

Chendrasekhar A, 2020, GLOB PEDIAT HLTH, V7

Dewan MC, 2019, J NEUROSURG, V130, P1080, DOI 10.3171/2017.10.JNS17352

Duncan ID, 2018, P NATL ACAD SCI USA, V115, pE11807, DOI 10.1073/pnas.1808064115

Duncan ID, 2017, P NATL ACAD SCI USA, V114, pE9685, DOI 10.1073/pnas.1714183114

Fernandez-Castaneda A, 2020, ACTA NEUROPATHOL, V139, P365, DOI 10.1007/s00401-019-02073-1

Fernandez-Castaneda A, 2016, BRAIN BEHAV IMMUN, V57, P1, DOI 10.1016/j.bbi.2016.01.005

Flygt J, 2018, J NEUROTRAUM, V35, P2837, DOI 10.1089/neu.2018.5660

Flygt J, 2017, RESTOR NEUROL NEUROS, V35, P251, DOI 10.3233/RNN-160675

Freedman D, 2020, NEUROL NEUROBIOL, V3, P2

Frondelli MJ, 2022, J NEUROSCI RES, V100, P578, DOI 10.1002/jnr.24984

Haarbauer-Krupa J, 2021, J NEUROTRAUM, V38, P3235, DOI 10.1089/neu.2021.0062

Hlavac N, 2020, NEUROSCI LETT, V739, DOI 10.1016/j.neulet.2020.135405

Hu Q, 2021, FRONT PHYS, V8, P653

Hui E, 2019, BIOMACROMOLECULES, V20, P4126, DOI 10.1021/acs.biomac.9b00965

Kim J, 2020, GLIA, V68, P2070, DOI 10.1002/glia.23827

Kinder HA, 2019, NEURAL REGEN RES, V14, P413, DOI 10.4103/1673-5374.245334

Kuhn S, 2019, CELLS-BASEL, V8, DOI 10.3390/cells8111424

Liaudanskaya V, 2020, ADV HEALTHC MATER, V9, DOI 10.1002/adhm.202000122

Maggiore JC, 2020, J TISSUE ENG REGEN M, V14, P1892, DOI 10.1002/term.3145

Marion CM, 2018, J NEUROSCI, V38, P8723, DOI 10.1523/JNEUROSCI.0819-18.2018

Meco E, 2020, BIOMACROMOLECULES, V21, P4724, DOI 10.1021/acs.biomac.0c00828

Nagoshi N, 2018, STEM CELL TRANSL MED, V7, P806, DOI 10.1002/sctm.17-0269

Nelson LD, 2019, JAMA NEUROL, V76, P1049, DOI 10.1001/jamaneurol.2019.1313

Pu H, J CEREBR BLOOD F MET, V41, P511

Reber J, 2021, P NATL ACAD SCI USA, V118, DOI 10.1073/pnas.2018784118

Sen T, 2020, J NEUROSCI, V40, P424, DOI 10.1523/JNEUROSCI.0718-19.2019

Shi W, 2021, ADV HEALTHC MATER, V10, DOI 10.1002/adhm.202100180

SWANSON LW, 1995, TRENDS NEUROSCI, V18, P471, DOI 10.1016/0166-2236(95)92766-J

Tang JD, 2019, ACS BIOMATER SCI ENG, V5, P2117, DOI 10.1021/acsbiomaterials.9b00389

Tang JD, 2019, J AM CHEM SOC, V141, P4886, DOI 10.1021/jacs.8b13363

Tsui CT, 2021, J MECH BEHAV BIOMED, V114, DOI 10.1016/j.jmbbm.2020.104176

Unal DB, 2020, BIOMACROMOLECULES, V21, P4962, DOI 10.1021/acs.biomac.0c01164

van Tilborg E, 2018, GLIA, V66, P221, DOI 10.1002/glia.23256

Ved R, 2021, SCI REP-UK, V11, P1

Vink R, 2018, J NEUROSCI RES, V96, P527, DOI 10.1002/jnr.24079

Wu YH, 2021, J NEUROTRAUM, V38, P2336, DOI 10.1089/neu.2020.7402

NR 43

TC 0

Z9 0

U1 0

U2 3

PU ELSEVIER

PI AMSTERDAM

PA RADARWEG 29, 1043 NX AMSTERDAM, NETHERLANDS

SN 2468-4511

J9 CURR OPIN BIOMED ENG

JI Curr. Opin. Biomed. Eng.

PD JUN

PY 2022

VL 22

AR 100378

DI 10.1016/j.cobme.2022.100378

EA APR 2022

PG 8

WC Engineering, Biomedical

WE Science Citation Index Expanded (SCI-EXPANDED)

SC Engineering

GA 1D0WB

UT WOS:000793529300004

OA Bronze

DA 2023-06-10

ER

PT J

AU Hasan, A

Deeb, G

Rahal, R

Atwi, K

Mondello, S

Marei, HE

Gali, A

Sleiman, E

AF Hasan, Anwarul

Deeb, George

Rahal, Rahaf

Atwi, Khairallah

Mondello, Stefania

Marei, Hany Elsayed

Gali, Amr

Sleiman, Eliana

TI Mesenchymal Stem Cells in the Treatment of Traumatic Brain Injury

SO FRONTIERS IN NEUROLOGY

LA English

DT Review

DE mesenchymal stem cells; central nervous system; traumatic brain injury;

bone marrow; neurons

ID MARROW STROMAL CELLS; CENTRAL-NERVOUS-SYSTEM; IN-VITRO;

INTERNATIONAL-SOCIETY; EARLY ADULTHOOD; HEAD-INJURY; TRANSPLANTATION;

GROWTH; RISK; PATHOPHYSIOLOGY

AB Traumatic brain injury (TBI) is characterized by a disruption in the normal function of the brain due to an injury following a trauma, which can potentially cause severe physical, cognitive, and emotional impairment. The primary insult to the brain initiates secondary injury cascades consisting of multiple complex biochemical responses of the brain that significantly influence the overall severity of the brain damage and clinical sequelae. The use of mesenchymal stem cells (MSCs) offers huge potential for application in the treatment of TBI. MSCs have immunosuppressive properties that reduce inflammation in injured tissue. As such, they could be used to modulate the secondary mechanisms of injury and halt the progression of the secondary insult in the brain after injury. Particularly, MSCs are capable of secreting growth factors that facilitate the regrowth of neurons in the brain. The relative abundance of harvest sources of MSCs also makes them particularly appealing. Recently, numerous studies have investigated the effects of infusion of MSCs into animal models of TBI. The results have shown significant improvement in the motor function of the damaged brain tissues. In this review, we summarize the recent advances in the application of MSCs in the treatment of TBI. The review starts with a brief introduction of the pathophysiology of TBI, followed by the biology of MSCs, and the application of MSCs in TBI treatment. The challenges associated with the application of MSCs in the treatment of TBI and strategies to address those challenges in the future have also been discussed.

C1 [Hasan, Anwarul] Qatar Univ, Dept Mech & Ind Engn, Doha, Qatar.

[Deeb, George; Rahal, Rahaf; Atwi, Khairallah; Gali, Amr; Sleiman, Eliana] Amer Univ Beirut, Biomed Engn, Beirut, Lebanon.

[Deeb, George; Rahal, Rahaf; Atwi, Khairallah; Gali, Amr; Sleiman, Eliana] Amer Univ Beirut, Dept Mech Engn, Beirut, Lebanon.

[Mondello, Stefania] Univ Messina, Dept Biomed & Dent Sci & Morphofunct Imaging, Messina, Italy.

[Marei, Hany Elsayed] Qatar Univ, Biomed Res Ctr, Doha, Qatar.

C3 Qatar University; American University of Beirut; American University of

Beirut; University of Messina; Qatar University

RP Hasan, A (通讯作者)，Qatar Univ, Dept Mech & Ind Engn, Doha, Qatar.

EM hasan1@mit.edu

RI Mondello, Stefania/A-1813-2012; Hasan, Anwarul/AAX-7440-2020; Marei,

Hany E/AAC-7076-2019

OI Mondello, Stefania/0000-0002-8587-3614; Hasan,

Anwarul/0000-0001-8380-2233; Marei, Hany E/0000-0002-0069-4212; Deeb,

George/0000-0003-1292-7906; Atwi, Khairallah/0000-0002-8103-8654

FU Gulf Cooperation Council [GCC-2017-005]; Qatar Foundation

[NPRP9-144-3-021]; Qatar University [QUUG-CENG-MIE-15/16-7,

QUST-CENG-FALL-15/16-20]

FX The authors acknowledge the grants GCC-2017-005 from Gulf Cooperation

Council research program, NPRP9-144-3-021 from Qatar Foundation, and

QUUG-CENG-MIE-15/16-7 and QUST-CENG-FALL-15/16-20 from Qatar University.

CR Abbott NJ, 1996, MOL MED TODAY, V2, P106, DOI 10.1016/1357-4310(96)88720-X

Alder J, 2011, JOVE-J VIS EXP, DOI 10.3791/3063

Algattas H, 2014, INT J MOL SCI, V15, P309, DOI 10.3390/ijms15010309

Anbari F, 2014, NEURAL REGEN RES, V9, P919, DOI 10.4103/1673-5374.133133

[Anonymous], 2012, Drug Discov Today Technol, V9, pe71, DOI 10.1016/j.ddtec.2011.12.002

AVELLINO AM, 1995, EXP NEUROL, V136, P183, DOI 10.1006/exnr.1995.1095

Azari MF, 2010, CURR NEUROPHARMACOL, V8, P316, DOI 10.2174/157015910793358204

Azizi SA, 1998, P NATL ACAD SCI USA, V95, P3908, DOI 10.1073/pnas.95.7.3908

Barbash IM, 2003, CIRCULATION, V108, P863, DOI 10.1161/01.CIR.0000084828.50310.6A

Bartholomew A, 2002, EXP HEMATOL, V30, P42, DOI 10.1016/S0301-472X(01)00769-X

Cernak Ibolja, 2005, NeuroRx, V2, P410, DOI 10.1602/neurorx.2.3.410

Chamberlain G, 2007, STEM CELLS, V25, P2739, DOI 10.1634/stemcells.2007-0197

Chang CP, 2013, CLIN SCI, V124, P165, DOI 10.1042/CS20120226

Cheng JL, 2010, CHIN J TRAUMATOL, V13, P173, DOI 10.3760/cma.j.issn.1008-1275.2010.03.008

Chuang TJ, 2012, J TRAUMA ACUTE CARE, V73, P1161, DOI 10.1097/TA.0b013e318265d128

Cox CS, 2011, NEUROSURGERY, V68, P588, DOI 10.1227/NEU.0b013e318207734c

Cox J., 2014, TREATMENT OF ADULT S

Meirelles LDS, 2006, J CELL SCI, V119, P2204, DOI 10.1242/jcs.02932

Dardiotis E, 2012, BRAIN INJURY PATHOGE, P23

Di Nicola M, 2002, BLOOD, V99, P3838, DOI 10.1182/blood.V99.10.3838

Djouad F, 2003, BLOOD, V102, P3837, DOI 10.1182/blood-2003-04-1193

Dobrowolski S., 2013, AM J NEUROSCIENCE, V4, P13, DOI DOI 10.3844/AMJNSP.2013.13.24

Dominici M, 2006, CYTOTHERAPY, V8, P315, DOI 10.1080/14653240600855905

Faul M, 2007, J TRAUMA, V63, P1271, DOI 10.1097/TA.0b013e3181493080

Finkelstein EA, 2006, INCIDENCE EC BURDEN, DOI 10.1093/acprof:oso/9780195179484.001.0001

FRIEDENSTEIN AJ, 1976, EXP HEMATOL, V4, P267

Fukuchi Y, 2004, STEM CELLS, V22, P649, DOI 10.1634/stemcells.22-5-649

Galindo LT, 2011, NEUROL RES INT, V2011, DOI 10.1155/2011/564089

Gardner RC, 2015, MOL CELL NEUROSCI, V66, P75, DOI 10.1016/j.mcn.2015.03.001

Gardner RC, 2015, ANN NEUROL, V77, P987, DOI 10.1002/ana.24396

Gardner RC, 2014, JAMA NEUROL, V71, P1490, DOI 10.1001/jamaneurol.2014.2668

Gardner RC, 2014, ANN NEUROL, V75, P339, DOI 10.1002/ana.24121

Greve MW, 2009, MT SINAI J MED, V76, P97, DOI 10.1002/msj.20104

Grigorian AS, 2011, B EXP BIOL MED+, V150, P551, DOI 10.1007/s10517-011-1187-1

Guan J, 2013, BIOMATERIALS, V34, P5937, DOI 10.1016/j.biomaterials.2013.04.047

Hallam TM, 2004, J NEUROTRAUM, V21, P521, DOI 10.1089/089771504774129865

Heile AMB, 2009, NEUROSCI LETT, V463, P176, DOI 10.1016/j.neulet.2009.07.071

Holsinger T, 2002, ARCH GEN PSYCHIAT, V59, P17, DOI 10.1001/archpsyc.59.1.17

Hong SQ, 2011, NEUROCHEM RES, V36, P2391, DOI 10.1007/s11064-011-0567-2

Hoogduijn MJ, 2010, INT IMMUNOPHARMACOL, V10, P1496, DOI 10.1016/j.intimp.2010.06.019

Horwitz EM, 2005, CYTOTHERAPY, V7, P393, DOI 10.1080/14653240500319234

Hyder AA, 2007, NEUROREHABILITATION, V22, P341

in't Anker PS, 2004, STEM CELLS, V22, P1338, DOI 10.1634/stemcells.2004-0058

Jalloh I, 2015, METAB BRAIN DIS, V30, P615, DOI 10.1007/s11011-014-9628-y

Johnson VE, 2013, EXP NEUROL, V246, P35, DOI 10.1016/j.expneurol.2012.01.013

Kan I, 2005, CURR DRUG TARGETS, V6, P31, DOI 10.2174/1389450053344902

Karp JM, 2009, CELL STEM CELL, V4, P206, DOI 10.1016/j.stem.2009.02.001

Kiel MJ, 2008, NAT REV IMMUNOL, V8, P290, DOI 10.1038/nri2279

Kim HJ, 2010, J NEUROTRAUM, V27, P131, DOI [10.1089/neu.2008.0818, 10.1089/neu.2008-0818]

Kim J, 2009, ARCH PHARM RES, V32, P117, DOI 10.1007/s12272-009-1125-1

Kopen GC, 1999, P NATL ACAD SCI USA, V96, P10711, DOI 10.1073/pnas.96.19.10711

Kumagai G, 2013, EXP NEUROL, V248, P369, DOI 10.1016/j.expneurol.2013.06.028

Lam PK, 2013, J CLIN NEUROSCI, V20, P300, DOI 10.1016/j.jocn.2012.03.028

Langlois JA, 2006, TRAUMATIC BRAIN INJU

Lee OK, 2004, BLOOD, V103, P1669, DOI 10.1182/blood-2003-05-1670

Lenzlinger PM, 2001, MOL NEUROBIOL, V24, P169

Liu LN, 2013, STEM CELLS INT, V2013, DOI 10.1155/2013/435093

Liu Y, 2014, MOL MED REP, V9, P333, DOI 10.3892/mmr.2013.1803

Ponte AL, 2007, STEM CELLS, V25, P1737, DOI 10.1634/stemcells.2007-0054

Lucas SM, 2006, BRIT J PHARMACOL, V147, pS232, DOI 10.1038/sj.bjp.0706400

Maas AIR, 2008, LANCET NEUROL, V7, P728, DOI 10.1016/S1474-4422(08)70164-9

Madikians A, 2006, INDIAN J NEUROTRAUM, V3, P9

Mahmood A, 2006, J NEUROSURG, V104, P272, DOI 10.3171/jns.2006.104.2.272

Mahmood A, 2001, J NEUROSURG, V94, P589, DOI 10.3171/jns.2001.94.4.0589

Matsushita T, 2011, NEUROSCI LETT, V502, P41, DOI 10.1016/j.neulet.2011.07.021

Mckee AC, 2009, J NEUROPATH EXP NEUR, V68, P709, DOI 10.1097/NEN.0b013e3181a9d503

Mead B, 2015, NEURAL REGEN RES, V10, P371, DOI 10.4103/1673-5374.153681

Meirelles LD, 2009, CYTOKINE GROWTH F R, V20, P419, DOI 10.1016/j.cytogfr.2009.10.002

Menge T, 2012, SCI TRANSL MED, V4, DOI 10.1126/scitranslmed.3004660

Menon DK, 2010, ARCH PHYS MED REHAB, V91, P1637, DOI 10.1016/j.apmr.2010.05.017

Muguruma Y, 2006, BLOOD, V107, P1878, DOI 10.1182/blood-2005-06-2211

Munoz JR, 2005, P NATL ACAD SCI USA, V102, P18171, DOI 10.1073/pnas.0508945102

Myer DJ, 2006, BRAIN, V129, P2761, DOI 10.1093/brain/awl165

Okuma Y, 2013, NEUROSCI LETT, V554, P156, DOI 10.1016/j.neulet.2013.08.058

Parr AM, 2007, BONE MARROW TRANSPL, V40, P609, DOI 10.1038/sj.bmt.1705757

Pittenger MF, 1999, SCIENCE, V284, P143, DOI 10.1126/science.284.5411.143

Plassman BL, 2000, NEUROLOGY, V55, P1158, DOI 10.1212/WNL.55.8.1158

Prins M, 2013, DIS MODEL MECH, V6, P1307, DOI 10.1242/dmm.011585

PROCKOP DJ, 1997, SCIENCE, V276, P71, DOI [DOI 10.1126/SCIENCE.276.5309.71, 10.1126/science.276.5309.71]

Ramirez JJ, 1999, NEUROREPORT, V10, P1201, DOI 10.1097/00001756-199904260-00008

Rojas M, 2005, AM J RESP CELL MOL, V33, P145, DOI 10.1165/rcmb.2004-0330OC

Rolls A, 2009, NAT REV NEUROSCI, V10, P235, DOI 10.1038/nrn2591

Romine J, 2014, JOVE-J VIS EXP, DOI 10.3791/51781

Ruster B, 2006, BLOOD, V108, P3938, DOI 10.1182/blood-2006-05-025098

Sanchez-Ramos J, 2000, EXP NEUROL, V164, P247, DOI 10.1006/exnr.2000.7389

Schmidt A, 2006, EUR J CELL BIOL, V85, P1179, DOI 10.1016/j.ejcb.2006.05.015

Segers VFM, 2006, AM J PHYSIOL-HEART C, V290, pH1370, DOI 10.1152/ajpheart.00523.2005

Sharma Pushpa, 2012, Int J Crit Illn Inj Sci, V2, P172, DOI 10.4103/2229-5151.100931

Sharp DJ, 2014, NAT REV NEUROL, V10, P156, DOI 10.1038/nrneurol.2014.15

Silver J, 2004, NAT REV NEUROSCI, V5, P146, DOI 10.1038/nrn1326

Snyder Scott R, 2012, EMS World, V41, P40

Steingen C, 2008, J MOL CELL CARDIOL, V44, P1072, DOI 10.1016/j.yjmcc.2008.03.010

Tian CL, 2013, EXP CLIN TRANSPLANT, V11, P176, DOI 10.6002/ect.2012.0053

Toma C, 2002, CIRCULATION, V105, P93, DOI 10.1161/hc0102.101442

Torrente D, 2014, HUM EXP TOXICOL, V33, P673, DOI 10.1177/0960327113509659

Wagner JP, 1999, J NEUROSCI, V19, P6006, DOI 10.1523/JNEUROSCI.19-14-06006.1999

Walker PA, 2009, DIS MODEL MECH, V2, P23, DOI 10.1242/dmm.001198

Wang HS, 2004, STEM CELLS, V22, P1330, DOI 10.1634/stemcells.2004-0013

Wang Z, 2015, STEM CELLS, V33, P456, DOI 10.1002/stem.1878

Werner C, 2007, BRIT J ANAESTH, V99, P4, DOI 10.1093/bja/aem131

Werner C, 2017, TRAUMATIC BRAIN INJU

Wilson A, 2006, NAT REV IMMUNOL, V6, P93, DOI 10.1038/nri1779

Woodbury D, 2000, J NEUROSCI RES, V61, P364, DOI 10.1002/1097-4547(20000815)61:4<364::AID-JNR2>3.0.CO;2-C

Xiong Y, 1997, J NEUROTRAUM, V14, P23, DOI 10.1089/neu.1997.14.23

Xiong Y, 2013, NAT REV NEUROSCI, V14, P128, DOI 10.1038/nrn3407

Yuan Y, 2014, INT J NEUROSCI, V124, P524, DOI 10.3109/00207454.2013.859144

Zanier ER, 2014, NEUROTHERAPEUTICS, V11, P679, DOI 10.1007/s13311-014-0277-y

Zanier ER, 2011, CRIT CARE MED, V39, P2501, DOI 10.1097/CCM.0b013e31822629ba

Zhang R, 2013, J NEUROINFLAMM, V10, DOI 10.1186/1742-2094-10-106

Zhang YL, 2015, J NEUROSURG, V122, P856, DOI 10.3171/2014.11.JNS14770

Zhang ZX, 2008, CYTOTHERAPY, V10, P134, DOI 10.1080/14653240701883061

NR 111

TC 87

Z9 95

U1 1

U2 21

PU FRONTIERS MEDIA SA

PI LAUSANNE

PA AVENUE DU TRIBUNAL FEDERAL 34, LAUSANNE, CH-1015, SWITZERLAND

SN 1664-2295

J9 FRONT NEUROL

JI Front. Neurol.

PD FEB 20

PY 2017

VL 8

AR 28

DI 10.3389/fneur.2017.00028

PG 15

WC Clinical Neurology; Neurosciences

WE Science Citation Index Expanded (SCI-EXPANDED)

SC Neurosciences & Neurology

GA EL0US

UT WOS:000394337900001

PM 28265255

OA gold, Green Published

DA 2023-06-10

ER

PT J

AU Stabenfeldt, SE

Irons, HR

LaPlaca, MC

AF Stabenfeldt, Sarah E.

Irons, Hillary R.

LaPlaca, Michelle C.

TI Stem Cells and Bioactive Scaffolds as a Treatment for Traumatic Brain

Injury

SO CURRENT STEM CELL RESEARCH & THERAPY

LA English

DT Review

DE Traumatic brain injury; stroke; tissue engineering; stem cell; cell

transplantation

ID NEURAL PROGENITOR CELLS; MARROW STROMAL CELLS; NERVE GROWTH-FACTOR;

BONE-MARROW; PARKINSONS-DISEASE; IN-VITRO; NEURONAL DIFFERENTIATION;

FUNCTIONAL RECOVERY; EXTRACELLULAR-MATRIX; CASPASE INHIBITION

AB Successful repair of the injured brain is critical, as traumatic brain injury pathology often involves a secondary cascade of insults that may ultimately lead to worsened neurologic dysfunction. Damage is balanced by the brain's attempt to repair itself, the genetic profile of the person, underlying health issues, and age, among other factors. The challenge in using a tissue engineering approach to repair and regeneration is centered at the heterogeneous and complex environment, variables that are difficult to measure and interpret. The brain must be in a state that minimizes rejection, inflammation, immune response, and donor cell death to maximize the intended benefit. Tissue engineering, using a bioactive based scaffold to both counter some of the hostile factors and to chaperone donor cells into the brain, has merit, yet the complexity of transplanting a combination biologic construct to the brain has yet to be successfully transferred to the clinic. Several options, such as cell source, scaffold composition, as well as delivery methods will be discussed.

C1 [LaPlaca, Michelle C.] Georgia Inst Technol, Coulter Dept Biomed Engn, Atlanta, GA 30332 USA.

Emory Univ, Petit Inst Bioengn & Biosci, Lab Neuroengn, Atlanta, GA 30332 USA.

C3 University System of Georgia; Georgia Institute of Technology; Emory

University

RP LaPlaca, MC (通讯作者)，Georgia Inst Technol, Coulter Dept Biomed Engn, 313 Ferst Dr, Atlanta, GA 30332 USA.

EM michelle.laplaca@bme.gatech.edu

CR AEBISCHER P, 1991, J BIOMECH ENG-T ASME, V113, P178, DOI 10.1115/1.2891231

Aizawa Y, 2008, BIOMATERIALS, V29, P4676, DOI 10.1016/j.biomaterials.2008.08.018

[Anonymous], 2010, HLTH PEOPL 2010 REP

AOTA S, 1994, J BIOL CHEM, V269, P24756

Bakshi A, 2006, EUR J NEUROSCI, V23, P2119, DOI 10.1111/j.1460-9568.2006.04743.x

Bakshi A, 2005, BRAIN RES, V1065, P8, DOI 10.1016/j.brainres.2005.09.059

Bartholomew A, 2002, EXP HEMATOL, V30, P42, DOI 10.1016/S0301-472X(01)00769-X

Bjorklund A, 2005, NOVART FDN SYMP, V265

Bjorklund Anders, 2005, V265, P174

Bondar VM, 2002, MOL CANCER THER, V1, P989

Boockvar JA, 2005, NEUROSURGERY, V56, P163, DOI 10.1227/01.NEU.0000145866.25433.FF

Boontheekul T, 2003, CURR OPIN BIOTECH, V14, P559, DOI 10.1016/j.copbio.2003.08.004

Borlongan CV, 1998, NEUROREPORT, V9, P2837, DOI 10.1097/00001756-199808240-00028

BOWDITCH RD, 1994, J BIOL CHEM, V269, P10856

Bramlett HM, 2002, ACTA NEUROPATHOL, V103, P607, DOI 10.1007/s00401-001-0510-8

Brazelton TR, 2000, SCIENCE, V290, P1775, DOI 10.1126/science.290.5497.1775

BREGMAN BS, 1986, J COMP NEUROL, V244, P86, DOI 10.1002/cne.902440107

Bullock MR, 2007, J NEUROTRAUM, V24, pVII, DOI 10.1089/neu.2007.9998

Busch SA, 2007, CURR OPIN NEUROBIOL, V17, P120, DOI 10.1016/j.conb.2006.09.004

Byrnes KR, 2007, NEUROCHEM RES, V32, P1799, DOI 10.1007/s11064-007-9312-2

Campos LS, 2004, DEVELOPMENT, V131, P3433, DOI 10.1242/dev.01199

Carson AE, 2009, REGEN MED, V4, P593, DOI 10.2217/RME.09.30

Chen JL, 2002, J NEUROL SCI, V199, P17, DOI 10.1016/S0022-510X(02)00075-8

Chen S, 2003, EXP NEUROL, V182, P87, DOI 10.1016/S0014-4886(03)00002-5

Chopp M, 2002, LANCET NEUROL, V1, P92, DOI 10.1016/S1474-4422(02)00040-6

Chothia C, 1997, ANNU REV BIOCHEM, V66, P823, DOI 10.1146/annurev.biochem.66.1.823

Clausen T, 2005, J NEUROSURG, V103, P597, DOI 10.3171/jns.2005.103.4.0597

Clausen T, 2001, CURR PHARM DESIGN, V7, P1517, DOI 10.2174/1381612013397267

Coats B, 2006, J BIOMECH, V39, P2521, DOI 10.1016/j.jbiomech.2005.07.020

Cogle CR, 2004, LANCET, V363, P1432, DOI 10.1016/S0140-6736(04)16102-3

Conti AC, 1998, J NEUROSCI, V18, P5663

Corti S., 2003, Current Gene Therapy, V3, P247, DOI 10.2174/1566523034578375

Crigler L, 2006, EXP NEUROL, V198, P54, DOI 10.1016/j.expneurol.2005.10.029

Cui X, 2007, STEM CELLS, V25, P2777, DOI 10.1634/stemcells.2007-0169

Cullen DK, 2007, J NEUROSCI RES, V85, P3642, DOI 10.1002/jnr.21434

DAI WG, 1994, BIO-TECHNOL, V12, P797, DOI 10.1038/nbt0894-797

Dezawa M, 2006, CELL MOL LIFE SCI, V63, P2764, DOI 10.1007/s00018-006-6191-7

Dhara SK, 2008, J CELL BIOCHEM, V105, P633, DOI 10.1002/jcb.21891

Dihne M, 2006, STEM CELLS, V24, P1458, DOI 10.1634/stemcells.2005-0413

Dimos JT, 2008, SCIENCE, V321, P1218, DOI 10.1126/science.1158799

Dominici M, 2006, CYTOTHERAPY, V8, P315, DOI 10.1080/14653240600855905

Duan WM, 2000, NEUROSCIENCE, V100, P521, DOI 10.1016/S0306-4522(00)00299-2

Dunnett SB, 2001, NAT REV NEUROSCI, V2, P365, DOI 10.1038/35072572

DURING MJ, 1992, EXP NEUROL, V115, P193, DOI 10.1016/0014-4886(92)90053-S

Emgard M, 2003, J NEUROCHEM, V86, P1223, DOI 10.1046/j.1471-4159.2003.01931.x

Engler AJ, 2006, CELL, V126, P677, DOI 10.1016/j.cell.2006.06.044

Faden AI, 1996, PHARMACOL TOXICOL, V78, P12, DOI 10.1111/j.1600-0773.1996.tb00173.x

Faul M., 2010, TRAUMATIC BRAIN INJU

Fawcett JW, 1999, BRAIN RES BULL, V49, P377, DOI 10.1016/S0361-9230(99)00072-6

Flanagan LA, 2006, J NEUROSCI RES, V83, P845, DOI 10.1002/jnr.20778

Freire E, 2002, J CELL SCI, V115, P4867, DOI 10.1242/jcs.00173

Fried A, 1996, J CELL BIOCHEM, V61, P246

Gage FH, 2000, SCIENCE, V287, P1433, DOI 10.1126/science.287.5457.1433

Gao JL, 2006, EXP NEUROL, V201, P281, DOI 10.1016/j.expneurol.2006.04.039

Garcia AJ, 1999, MOL BIOL CELL, V10, P785, DOI 10.1091/mbc.10.3.785

Gary DS, 2003, J NEUROCHEM, V84, P878, DOI 10.1046/j.1471-4159.2003.01579.x

Gary DS, 2001, J NEUROCHEM, V76, P1485, DOI 10.1046/j.1471-4159.2001.00173.x

GATES MA, 1995, J COMP NEUROL, V361, P249, DOI 10.1002/cne.903610205

Gefen A, 2004, J BIOMECH, V37, P1339, DOI 10.1016/j.jbiomech.2003.12.032

Glennie S, 2005, BLOOD, V105, P2821, DOI 10.1182/blood-2004-09-3696

Gronthos S, 2001, BONE, V28, P174, DOI 10.1016/S8756-3282(00)00424-5

Gu ZZ, 2005, J NEUROSCI, V25, P6401, DOI 10.1523/JNEUROSCI.1563-05.2005

Guan KM, 2001, CELL TISSUE RES, V305, P171, DOI 10.1007/s004410100416

Hagan M, 2003, NEUROSCI LETT, V351, P149, DOI 10.1016/j.neulet.2003.07.021

Harms KM, 2010, PLOS ONE, V5, DOI 10.1371/journal.pone.0009767

Hattiangady B, 2006, NEUROSCIENCE, V139, P1369, DOI 10.1016/j.neuroscience.2006.01.058

HEATON MB, 1988, J NEUROSCI RES, V19, P212, DOI 10.1002/jnr.490190206

Hern DL, 1998, J BIOMED MATER RES, V39, P266, DOI 10.1002/(SICI)1097-4636(199802)39:2<266::AID-JBM14>3.0.CO;2-B

Herzog EL, 2003, BLOOD, V102, P3483, DOI 10.1182/blood-2003-05-1664

Hu BY, 2010, P NATL ACAD SCI USA, V107, P4335, DOI 10.1073/pnas.0910012107

Huang RQ, 2010, NEUROSCI LETT, V473, P22, DOI 10.1016/j.neulet.2010.02.011

Hubbell JA, 1999, CURR OPIN BIOTECH, V10, P123, DOI 10.1016/S0958-1669(99)80021-4

Jacques TS, 1998, DEVELOPMENT, V125, P3167

Jeong B, 2002, ADV DRUG DELIVER REV, V54, P37, DOI 10.1016/S0169-409X(01)00242-3

Jiang YH, 2002, NATURE, V418, P41, DOI 10.1038/nature00870

Jin KL, 2002, P NATL ACAD SCI USA, V99, P11946, DOI 10.1073/pnas.182296499

Johnson PJ, 2010, CELL TRANSPLANT, V19, P89, DOI 10.3727/096368909X477273

Kallos MS, 2003, MED BIOL ENG COMPUT, V41, P271, DOI 10.1007/BF02348431

Kanelos SK, 1998, J HEAD TRAUMA REHAB, V13, P1, DOI 10.1097/00001199-199812000-00003

Karumbayaram S, 2009, STEM CELLS, V27, P806, DOI 10.1002/stem.31

Kearns SM, 2003, EXP NEUROL, V182, P240, DOI 10.1016/S0014-4886(03)00124-9

Keselowsky BG, 2003, J BIOMED MATER RES A, V66A, P247, DOI 10.1002/jbm.a.10537

Kim DS, 2007, CELL TRANSPLANT, V16, P117

Kim DE, 2006, RADIOLOGY, V241, P822, DOI 10.1148/radiol.2413050466

Kim HK, 2009, TISSUE ENG PT A, V15, P923, DOI 10.1089/ten.tea.2007.0407

Kim J, 2008, J MATER SCI-MATER M, V19, P3311, DOI 10.1007/s10856-008-3469-3

King WJ, 2010, J BIOMED MATER RES A, V93A, P1110, DOI 10.1002/jbm.a.32601

Kondziolka D, 2002, J CLIN NEUROSCI, V9, P225, DOI 10.1054/jocn.2001.1043

Kornblum HI, 2007, STROKE, V38, P810, DOI 10.1161/01.STR.0000255757.12198.0f

KOUTOUZIS TK, 1994, CRIT REV NEUROBIOL, V8, P125

Kukekov VG, 1999, EXP NEUROL, V156, P333, DOI 10.1006/exnr.1999.7028

LABBE R, 1983, SCIENCE, V221, P470, DOI 10.1126/science.6683427

Le Bellego F, 2002, J ENDOCRINOL, V172, P45, DOI 10.1677/joe.0.1720045

Le Blanc K, 2003, EXP HEMATOL, V31, P890, DOI 10.1016/S0301-472X(03)00110-3

Le Blanc K, 2004, LANCET, V363, P1439, DOI 10.1016/S0140-6736(04)16104-7

Learish RD, 2000, DEV BRAIN RES, V122, P97

Leipzig ND, 2010, J BIOMED MATER RES A, V93A, P625, DOI 10.1002/jbm.a.32573

Leone DP, 2005, J CELL SCI, V118, P2589, DOI 10.1242/jcs.02396

LESCAUDRON L, 1990, NEUROPSYCHOLOGIA, V28, P585, DOI 10.1016/0028-3932(90)90036-N

Lesny P, 2002, J CHEM NEUROANAT, V23, P243, DOI 10.1016/S0891-0618(02)00011-X

Li Y, 2002, NEUROLOGY, V59, P514, DOI 10.1212/WNL.59.4.514

Li Y, 2000, J CEREBR BLOOD F MET, V20, P1311, DOI 10.1097/00004647-200009000-00006

Lim DA, 2007, NEUROSURG CLIN N AM, V18, P81, DOI 10.1016/j.nec.2006.10.002

Lindvall Olle, 2004, NeuroRx, V1, P379, DOI 10.1602/neurorx.1.4.379

Liu CT, 2006, CELL IMMUNOL, V244, P19, DOI 10.1016/j.cellimm.2007.02.003

Liu S, 2000, P NATL ACAD SCI USA, V97, P6126, DOI 10.1073/pnas.97.11.6126

Llado J, 2004, MOL CELL NEUROSCI, V27, P322, DOI 10.1016/j.mcn.2004.07.010

Longhi L, 2005, TRANSPL IMMUNOL, V15, P143, DOI 10.1016/j.trim.2005.09.003

Lu D, 2001, NEUROREPORT, V12, P559, DOI 10.1097/00001756-200103050-00025

Lu DY, 2007, NEUROSURGERY, V61, P596, DOI 10.1227/01.NEU.0000290908.38438.B2

Lu DY, 2002, CELL TRANSPLANT, V11, P275

Lu P, 2003, EXP NEUROL, V181, P115, DOI 10.1016/S0014-4886(03)00037-2

Lutolf MP, 2005, NAT BIOTECHNOL, V23, P47, DOI 10.1038/nbt1055

Lutolf MP, 2003, P NATL ACAD SCI USA, V100, P5413, DOI 10.1073/pnas.0737381100

Maas AIR, 2004, ACT NEUR S, V89, P113

Mahmood A, 2006, J NEUROSURG, V104, P272, DOI 10.3171/jns.2006.104.2.272

Mahmood A, 2004, NEUROSURGERY, V55, P1185, DOI 10.1227/01.NEU.0000141042.14476.3C

Mahmood A, 2001, J NEUROSURG, V94, P589, DOI 10.3171/jns.2001.94.4.0589

Mahmood A, 2007, NEUROSURGERY, V61, P206, DOI 10.1227/01.neu.0000279914.37559.de

Mahoney MJ, 2007, J BIOMED MATER RES A, V81A, P269, DOI 10.1002/jbm.a.30970

Marchionini DM, 2004, CELL TRANSPLANT, V13, P273, DOI 10.3727/000000004783983972

Marchionini DM, 2003, J COMP NEUROL, V464, P172, DOI 10.1002/cne.10785

Marklund N, 2006, CURR PHARM DESIGN, V12, P1645, DOI 10.2174/138161206776843340

McIntosh TK, 1998, NEUROPATH APPL NEURO, V24, P251

McKay R, 1997, SCIENCE, V276, P66, DOI 10.1126/science.276.5309.66

Mezey E, 2000, EUR J PHARMACOL, V405, P297, DOI 10.1016/S0014-2999(00)00561-6

Mezey E, 2003, P NATL ACAD SCI USA, V100, P1364, DOI 10.1073/pnas.0336479100

Micci MA, 2005, NEUROGASTROENT MOTIL, V17, P557, DOI 10.1111/j.1365-2982.2005.00702.x

Nakajima M, 2007, BIOMATERIALS, V28, P1048, DOI 10.1016/j.biomaterials.2006.10.004

Narayan RK, 2002, J NEUROTRAUM, V19, P503, DOI 10.1089/089771502753754037

Ourednik J, 2002, NAT BIOTECHNOL, V20, P1103, DOI 10.1038/nbt750

PARK TG, 1992, J APPL POLYM SCI, V46, P659, DOI 10.1002/app.1992.070460413

Patterson J, 2010, MATER TODAY, V13, P14, DOI 10.1016/S1369-7021(10)70013-4

Philips MF, 2001, J NEUROSURG, V94, P765, DOI 10.3171/jns.2001.94.5.0765

Picard-Riera N, 2004, J NEUROSCI RES, V76, P223, DOI 10.1002/jnr.20040

Pierce JES, 1998, NEUROSCIENCE, V87, P359, DOI 10.1016/S0306-4522(98)00142-0

Pluchino S, 2005, NATURE, V436, P266, DOI 10.1038/nature03889

Potapova IA, 2008, J BIOL CHEM, V283, P13100, DOI 10.1074/jbc.M800184200

Powell SK, 1997, INT J BIOCHEM CELL B, V29, P401, DOI 10.1016/S1357-2725(96)00110-0

Prange MT, 2002, J BIOMECH ENG-T ASME, V124, P244, DOI 10.1115/1.1449907

Prestoz L, 2001, MOL CELL NEUROSCI, V18, P473, DOI 10.1006/mcne.2001.1037

Prockop DJ, 1997, SCIENCE, V276, P71, DOI 10.1126/science.276.5309.71

Qian LC, 2004, BIOMATERIALS, V25, P1331, DOI 10.1016/j.biomaterials.2003.08.013

Qu CS, 2009, J NEUROSURG, V111, P658, DOI 10.3171/2009.4.JNS081681

Qu RJ, 2007, NEUROPATHOLOGY, V27, P355, DOI 10.1111/j.1440-1789.2007.00792.x

Rafuse VF, 2005, NEUROSCIENCE, V131, P899, DOI 10.1016/j.neuroscience.2004.11.048

Raghupathi R, 2004, BRAIN PATHOL, V14, P215, DOI 10.1111/j.1750-3639.2004.tb00056.x

Rao MS, 2006, NEUROBIOL DIS, V21, P276, DOI 10.1016/j.nbd.2005.07.009

Rao MS, 1999, ANAT RECORD, V257, P137

REDMOND DE, 1986, LANCET, V1, P1125

Riess P, 2002, NEUROSURGERY, V51, P1043, DOI 10.1097/00006123-200210000-00035

Riess P, 2007, J NEUROTRAUM, V24, P216, DOI 10.1089/neu.2006.0141

Royo NC, 2003, CURR OPIN PHARMACOL, V3, P27, DOI 10.1016/S1471-4892(02)00006-1

Saha K, 2008, BIOPHYS J, V95, P4426, DOI 10.1529/biophysj.108.132217

Salewski RPF, 2010, J CELL PHYSIOL, V222, P515, DOI 10.1002/jcp.21995

Saltzman WM, 1999, PHARMACEUT RES, V16, P232, DOI 10.1023/A:1018824324275

Saporta S, 1997, CELL TRANSPLANT, V6, P579, DOI 10.1016/S0963-6897(97)00115-2

Schmidt CE, 2003, ANNU REV BIOMED ENG, V5, P293, DOI 10.1146/annurev.bioeng.5.011303.120731

Schouten JW, 2004, J NEUROTRAUM, V21, P1501, DOI 10.1089/0897715042441774

Schumm MA, 2004, EXP NEUROL, V185, P133, DOI 10.1016/j.expneurol.2003.09.017

Seidlits SK, 2010, BIOMATERIALS, V31, P3930, DOI 10.1016/j.biomaterials.2010.01.125

Seledtsov VI, 2005, BIOMED PHARMACOTHER, V59, P415, DOI 10.1016/j.biopha.2005.01.012

Shamekh R, 2005, CELL TRANSPLANT, V14, P551, DOI 10.3727/000000005783982747

Shear DA, 2004, BRAIN RES, V1026, P11, DOI 10.1016/j.brainres.2004.07.087

Shi MX, 2007, HAEMATOLOGICA, V92, P897, DOI 10.3324/haematol.10669

Shindo T, 2006, J MED INVESTIG, V53, P42, DOI 10.2152/jmi.53.42

Sinson G, 1996, J NEUROSURG, V84, P655, DOI 10.3171/jns.1996.84.4.0655

SLADEK JR, 1988, PROG BRAIN RES, V78, P497

Soares HD, 1995, J NEUROTRAUM, V12, P1059, DOI 10.1089/neu.1995.12.1059

Soldner F, 2009, CELL, V136, P964, DOI 10.1016/j.cell.2009.02.013

Sonoda Y, 1999, J BIOL CHEM, V274, P10566, DOI 10.1074/jbc.274.15.10566

Sortwell CE, 1998, J COMP NEUROL, V399, P530, DOI 10.1002/(SICI)1096-9861(19981005)399:4<530::AID-CNE6>3.0.CO;2-2

Sostak P, 2007, J NEUROPATH EXP NEUR, V66, P110, DOI 10.1097/nen.0b013e3180301be8

Stabenfeldt SE, 2006, J BIOMED MATER RES A, V77A, P718, DOI 10.1002/jbm.a.30638

STEIN DG, 1991, J NEUROSURG ANESTH, V3, P170, DOI 10.1097/00008506-199109000-00002

STEIN DG, 1992, ADV EXP MED BIOL, V325, P1

Stile RA, 2004, J BIOMAT SCI-POLYM E, V15, P865, DOI 10.1163/1568562041271129

STOKES BT, 1991, EXP NEUROL, V111, P312, DOI 10.1016/0014-4886(91)90098-W

Subramanian T, 2001, SEMIN NEUROL, V21, P103, DOI 10.1055/s-2001-13125

TABATA Y, 1993, PHARMACEUT RES, V10, P487, DOI 10.1023/A:1018929531410

Takahashi K, 2006, CELL, V126, P663, DOI 10.1016/j.cell.2006.07.024

Tang YM, 2007, CELL TRANSPLANT, V16, P159

Tate CC, 2009, J TISSUE ENG REGEN M, V3, P208, DOI 10.1002/term.154

Tate MC, 2004, MOL CELL NEUROSCI, V27, P22, DOI 10.1016/j.mcn.2004.05.001

Tate MC, 2002, CELL TRANSPLANT, V11, P283

Tate MC, 2001, BIOMATERIALS, V22, P1113, DOI 10.1016/S0142-9612(00)00348-3

Taupin P, 2006, INDIAN J MED RES, V124, P613

Thibault KL, 1998, J BIOMECH, V31, P1119, DOI 10.1016/S0021-9290(98)00122-5

Thurman DJ, 1999, J HEAD TRAUMA REHAB, V14, P602, DOI 10.1097/00001199-199912000-00009

VALOUSKOVA V, 1995, NEUROSCI LETT, V186, P103, DOI 10.1016/0304-3940(95)11295-8

VANDERWOLF CH, 1990, EXP BRAIN RES, V81, P426

Veenith Tonny, 2009, World J Emerg Surg, V4, P7, DOI 10.1186/1749-7922-4-7

Verfaillie CM, 2002, TRENDS CELL BIOL, V12, P502, DOI 10.1016/S0962-8924(02)02386-3

Walker PA, 2009, DIS MODEL MECH, V2, P23, DOI 10.1242/dmm.001198

Warden D, 2006, J HEAD TRAUMA REHAB, V21, P398, DOI 10.1097/00001199-200609000-00004

Watts C, 2000, CELL TRANSPLANT, V9, P223, DOI 10.1177/096368970000900208

Webb K, 2001, BIOMATERIALS, V22, P1017, DOI 10.1016/S0142-9612(00)00353-7

Wernig M, 2008, P NATL ACAD SCI USA, V105, P5856, DOI 10.1073/pnas.0801677105

Whittemore SR, 1999, EXP CELL RES, V252, P75, DOI 10.1006/excr.1999.4621

Willerth SM, 2007, ADV DRUG DELIVER REV, V59, P325, DOI 10.1016/j.addr.2007.03.014

Winkler C, 2005, TRENDS NEUROSCI, V28, P86, DOI 10.1016/j.tins.2004.12.006

Woltjen K, 2009, NATURE, V458, P766, DOI 10.1038/nature07863

Wong AM, 2005, BRAIN RES, V1063, P140, DOI 10.1016/j.brainres.2005.09.049

Wong J, 2005, NEUROCRIT CARE, V3, P177, DOI 10.1385/NCC:3:2:177

Xiong Y, 2009, EXPERT OPIN EMERG DR, V14, P67, DOI [10.1517/14728210902769601 , 10.1517/14728210902769601]

Yan J, 2004, J COMP NEUROL, V480, P101, DOI 10.1002/cne.20344

Yu XJ, 2003, TISSUE ENG, V9, P421, DOI 10.1089/107632703322066606

Zhang XP, 2005, CRIT CARE, V9, P66, DOI 10.1186/cc2950

Zhou HY, 2009, CELL STEM CELL, V4, P381, DOI 10.1016/j.stem.2009.04.005

NR 209

TC 6

Z9 6

U1 1

U2 40

PU BENTHAM SCIENCE PUBL LTD

PI SHARJAH

PA EXECUTIVE STE Y-2, PO BOX 7917, SAIF ZONE, 1200 BR SHARJAH, U ARAB

EMIRATES

SN 1574-888X

EI 2212-3946

J9 CURR STEM CELL RES T

JI Curr. Stem Cell Res. Ther.

PD SEP

PY 2011

VL 6

IS 3

BP 208

EP 220

PG 13

WC Cell & Tissue Engineering; Cell Biology

WE Science Citation Index Expanded (SCI-EXPANDED)

SC Cell Biology

GA 910LX

UT WOS:000301639800004

PM 21476977

DA 2023-06-10

ER

PT J

AU Okuma, Y

Wang, FF

Toyoshima, A

Kameda, M

Hishikawa, T

Tokunaga, K

Sugiu, K

Liu, KY

Haruma, J

Nishibori, M

Yasuhara, T

Date, I

AF Okuma, Yu

Wang, Feifei

Toyoshima, Atsuhiko

Kameda, Masahiro

Hishikawa, Tomohito

Tokunaga, Koji

Sugiu, Kenji

Liu, Keyue

Haruma, Jun

Nishibori, Masahiro

Yasuhara, Takao

Date, Isao

TI Mannitol enhances therapeutic effects of intra-arterial transplantation

of mesenchymal stem cells into the brain after traumatic brain injury

SO NEUROSCIENCE LETTERS

LA English

DT Article

DE Traumatic brain injury; Mesenchymal stem cells; Hypertonic solution

ID BONE-MARROW; RATS; PRESSURE; DELIVERY; BARRIER; STROKE

AB Traumatic brain injury (TBI) sustained in a traffic accident or a fall is a major cause of death that affects a broad range of ages. The aim of this study was to investigate the therapeutic effects of intra-arterial transplantation of mesenchymal stem cells (MSCs) combined with hypertonic glycerol (25%) or mannitol (25%) in a TBI model of rats. TBI models were produced with a fluid percussion device. At 24 h after TBI, MSCs (1 x 10(6) cells/100 mu l) with glycerol or mannitol were administered via the right internal carotid artery. Rats were evaluated behaviorally and immunohistochemically, and hyperpermeability of the blood-brain barrier (BBB) induced by hypertonic solutions was explored. Compared to PBS or glycerol, the administration of mannitol resulted in increased BBB disruption. The mannitol-treated rats showed significant improvement in motor function. Intra-arterial transplantation of MSCs caused no thromboembolic ischemia. Immunohistochemically, more MSCs were observed in the injured brain tissues of mannitol-treated rats than in glycerol or PBS-treated rats at 24 h after transplantation. Intra-arterial transplantation of MSCs combined with mannitol is an effective treatment in a TBI model of rats. This technique might be used for patients with diseases of the central nervous system including TBI. (C) 2013 Elsevier Ireland Ltd. All rights reserved.

C1 [Okuma, Yu; Wang, Feifei; Toyoshima, Atsuhiko; Kameda, Masahiro; Hishikawa, Tomohito; Tokunaga, Koji; Sugiu, Kenji; Haruma, Jun; Yasuhara, Takao; Date, Isao] Okayama Univ, Grad Sch Med Dent & Pharmaceut Sci, Dept Neurol Surg, Okayama, Japan.

[Okuma, Yu; Liu, Keyue; Haruma, Jun; Nishibori, Masahiro] Okayama Univ, Grad Sch Med Dent & Pharmaceut Sci, Dept Pharmacol, Okayama, Japan.

[Wang, Feifei] Kochi Univ, Sch Med, Ctr Innovat & Translat Med, Kochi 7838505, Japan.

C3 Okayama University; Okayama University; Kochi University

RP Wang, FF (通讯作者)，Kochi Univ, Sch Med, Ctr Innovat & Translat Med, Nanko Ku, Oko Cho, Kochi 7838505, Japan.

EM f-wang@kochi-u.ac.jp

RI Yasuhara, Takao/AEU-1075-2022

OI Okuma, Yu/0000-0003-4769-8111

FU Ministry of Education, Culture, Sports, Science and Technology, Japan;

Grants-in-Aid for Scientific Research [24390061, 23791602] Funding

Source: KAKEN

FX The authors thank Ms. Masako Arao and Ms. Natsuki Uemori for their

assistance. This work was supported in part by Grants-in-Aid for

Scientific Research and Health Science Research Grants for Research on

Brain Science from the Ministry of Education, Culture, Sports, Science

and Technology, Japan.

CR Armin SS, 2008, ACTA NEUROCHIR SUPPL, V104, P421

Borlongan CV, 2012, CURR PHARM DESIGN, V18, P3670

Borlongan CV, 2004, STROKE, V35, P2385, DOI 10.1161/01.STR.0000141680.49960.d7

COSOLO WC, 1989, AM J PHYSIOL, V256, pR443, DOI 10.1152/ajpregu.1989.256.2.R443

Galindo LT, 2011, NEUROL RES INT, V2011, DOI 10.1155/2011/564089

Gao JL, 2006, EXP NEUROL, V201, P281, DOI 10.1016/j.expneurol.2006.04.039

GHAJAR J, 1995, CRIT CARE MED, V23, P560, DOI 10.1097/00003246-199503000-00023

Kroll RA, 1998, NEUROSURGERY, V42, P1083, DOI 10.1097/00006123-199805000-00082

Lee HS, 2003, STEM CELLS, V21, P190, DOI 10.1634/stemcells.21-2-190

Li Y, 2002, NEUROLOGY, V59, P514, DOI 10.1212/WNL.59.4.514

Mahmood A, 2003, NEUROSURGERY, V53, P697, DOI 10.1227/01.NEU.0000079333.61863.AA

MCGRAW CP, 1983, NEUROSURGERY, V13, P269, DOI 10.1227/00006123-198309000-00009

Okuma Y, 2012, ANN NEUROL, V72, P373, DOI 10.1002/ana.23602

Sakowitz OW, 2007, J TRAUMA, V62, P292, DOI 10.1097/01.ta.0000203560.03937.2d

Scalfani MT, 2012, J CRIT CARE, V27, DOI 10.1016/j.jcrc.2011.10.008

Schierhout G., 2000, COCHRANE DB SYST REV, V2

Seyfried D, 2004, J NEUROSURG, V101, P104, DOI 10.3171/jns.2004.101.1.0104

Seyfried DM, 2008, BRAIN RES, V1224, P12, DOI 10.1016/j.brainres.2008.05.080

Walczak P, 2008, STROKE, V39, P1569, DOI 10.1161/STROKEAHA.107.502047

Wang FF, 2010, BMC NEUROSCI, V11, DOI 10.1186/1471-2202-11-52

WISE BL, 1961, ARCH NEUROL-CHICAGO, V4, P200, DOI 10.1001/archneur.1961.00450080082009

Yasuhara T, 2010, J CELL MOL MED, V14, P914, DOI 10.1111/j.1582-4934.2008.00671.x

NR 22

TC 26

Z9 27

U1 0

U2 7

PU ELSEVIER IRELAND LTD

PI CLARE

PA ELSEVIER HOUSE, BROOKVALE PLAZA, EAST PARK SHANNON, CO, CLARE, 00000,

IRELAND

SN 0304-3940

EI 1872-7972

J9 NEUROSCI LETT

JI Neurosci. Lett.

PD OCT 25

PY 2013

VL 554

BP 156

EP 161

DI 10.1016/j.neulet.2013.08.058

PG 6

WC Neurosciences

WE Science Citation Index Expanded (SCI-EXPANDED)

SC Neurosciences & Neurology

GA 261ON

UT WOS:000327674600030

PM 24016413

DA 2023-06-10

ER

PT J

AU Wennersten, A

Holmin, S

Al Nimer, F

Meijer, X

Wahlberg, LU

Mathiesen, T

AF Wennersten, Andre

Holmin, Staffan

Al Nimer, Faiez

Meijer, Xia

Wahlberg, Lars U.

Mathiesen, Tiit

TI Sustained survival of xenografted human neural stem/progenitor cells in

experimental brain trauma despite discontinuation of immunosuppression

SO EXPERIMENTAL NEUROLOGY

LA English

DT Article

DE fetal stem cell; traumatic brain injury; immunosuppressed; neural

progenitor; immunesuppressed; xenotransplantation; xenogeneic;

cyclosporine

ID CENTRAL-NERVOUS-SYSTEM; EMBRYONIC HUMAN CNS; STEM-CELLS; CYCLOSPORINE-A;

PROGENITOR CELLS; FUNCTIONAL RECOVERY; DOPAMINE NEURONS; ADULT RATS;

INJURY; TRANSPLANTATION

AB Neural stein cells have emerged as a promising therapeutic tool in CNS disease and injuries. In the clinical setting, cultured human neural stem/progenitor cells (hNSC) are an attractive possibility for transplantation to the damaged brain. However, transplantation of hNSC requires toxic immunosuppressive treatment to avoid rejection. The aim of the current study was to evaluate if shortening the duration of immuno suppression by cyclosporin A would affect hNSC survival and differentiation after transplantation to the site of a focal brain injury in the rat. hNSC were xenografted to the hippocampus and the medial limit of an experimentally induced cortical contusion. The animals received immumosuppression for either 6 or 3 weeks or no immunosuppression. The status of the grafted human cells was analysed by immunohistochemistry. No statistically significant differences were observed between the two immunosuppressed groups regarding graft survival, migration or proliferation at 6 weeks post-transplantation. In contrast, the graft survival was extremely poor in the non-immunosuppressed group. Furthermore, the expression of the differentiation markers nestin, neuronal nuclei (NeuN) and glial fibrillary acidic protein (GFAP) in the transplanted cells did not differ significantly between the two immunosuppressed groups. Moreover, a fourth group of eight animals that were immunosuppressed for 3 weeks were allowed to survive for 6 months. Five of these rats demonstrated robust graft survival in the hippocampus and scattered cells in the cortex. This study demonstrates the importance of immunosuppression but also the possibility of shortening immunosuppression without impacting on the phenotype of the grafted hNSC. (c) 2006 Elsevier Inc. All rights reserved.

C1 Sect Clin CNS Res, Dept Clin Neurosci, S-17176 Stockholm, Sweden.

NS Gene AS, Copenhagen, Denmark.

RP Wennersten, A (通讯作者)，Sect Clin CNS Res, Dept Clin Neurosci, S-17176 Stockholm, Sweden.

EM andre.wennersten@ki.se

RI Nimer, Faiez Al/ABB-9465-2020; mathiesen, tiit/ABC-3627-2021

OI Holmin, Staffan/0000-0002-1628-1615; Al Nimer,

Faiez/0000-0003-0937-5995; Mathiesen, Tiit/0000-0001-9463-1919

CR Aboody KS, 2000, P NATL ACAD SCI USA, V97, P12846, DOI 10.1073/pnas.97.23.12846

Akhlaghi F, 2002, CLIN PHARMACOKINET, V41, P615, DOI 10.2165/00003088-200241090-00001

Al Nimer F, 2004, NEUROREPORT, V15, P1871, DOI 10.1097/00001756-200408260-00007

Aleksandrova MA, 2002, DEV BRAIN RES, V134, P143, DOI 10.1016/S0165-3806(02)00273-0

Armstrong RJE, 2001, NEUROSCIENCE, V106, P201, DOI 10.1016/S0306-4522(01)00273-1

Brevig T, 2000, TRENDS NEUROSCI, V23, P337, DOI 10.1016/S0166-2236(00)01605-2

BRUNDIN P, 1989, EXP BRAIN RES, V75, P195

Buki A, 1999, J NEUROTRAUM, V16, P511, DOI 10.1089/neu.1999.16.511

Carpenter MK, 1999, EXP NEUROL, V158, P265, DOI 10.1006/exnr.1999.7098

Castilho RF, 2000, EXP NEUROL, V164, P94, DOI 10.1006/exnr.2000.7405

Chu K, 2003, STROKE, V34, P241

Cicchetti F, 2003, XENOTRANSPLANTATION, V10, P41, DOI 10.1034/j.1399-3089.2003.01130.x

Conti AC, 1998, J NEUROSCI, V18, P5663

Deacon T, 1998, EXP NEUROL, V149, P28, DOI 10.1006/exnr.1997.6674

Duan WM, 2002, NEUROSCIENCE, V115, P495, DOI 10.1016/S0306-4522(02)00382-2

Ehtesham M, 2002, CANCER RES, V62, P5657

Flax JD, 1998, NAT BIOTECHNOL, V16, P1033, DOI 10.1038/3473

GIULIAN D, 1989, J NEUROSCI, V9, P4416

Hagan M, 2003, NEUROSCI LETT, V351, P149, DOI 10.1016/j.neulet.2003.07.021

HOLMIN S, 1995, ACTA NEUROCHIR, V132, P110, DOI 10.1007/BF01404857

Ishibashi S, 2004, J NEUROSCI RES, V78, P215, DOI 10.1002/jnr.20246

Kaminska B, 2004, J CELL MOL MED, V8, P45, DOI 10.1111/j.1582-4934.2004.tb00259.x

Kelly S, 2004, P NATL ACAD SCI USA, V101, P11839, DOI 10.1073/pnas.0404474101

Larsson LC, 2003, TRANSPLANTATION, V75, P1448, DOI 10.1097/01.TP.0000058807.45320.A2

Larsson LC, 2000, SCAND J IMMUNOL, V52, P249

Le Belle JE, 2004, J NEUROSCI RES, V76, P174, DOI 10.1002/jnr.20035

Li PA, 2000, EXP NEUROL, V165, P153, DOI 10.1006/exnr.2000.7459

MATHIESEN T, 1989, CANCER LETT, V44, P151, DOI 10.1016/0304-3835(89)90010-4

Mirza B, 2004, BRAIN RES BULL, V63, P105, DOI 10.1016/j.brainresbull.2004.01.009

Ogawa Y, 2002, J NEUROSCI RES, V69, P925, DOI 10.1002/jnr.10341

Ortega J D, 1992, Cell Transplant, V1, P33

OUREDNIK J, 2002, NAT BIOTECHNOL, V15, P15

PAKZABAN P, 1994, NEUROSCIENCE, V62, P989, DOI 10.1016/0306-4522(94)90338-7

Philips MF, 2001, J NEUROSURG, V94, P765, DOI 10.3171/jns.2001.94.5.0765

Riess P, 2002, NEUROSURGERY, V51, P1043, DOI 10.1097/00006123-200210000-00035

Scheff SW, 1999, J NEUROTRAUM, V16, P783, DOI 10.1089/neu.1999.16.783

Schwartz RH, 2003, ANNU REV IMMUNOL, V21, P305, DOI 10.1146/annurev.immunol.21.120601.141110

SHIBASAKI F, 1995, J CELL BIOL, V131, P735, DOI 10.1083/jcb.131.3.735

Snyder EY, 2004, J NEUROSCI RES, V76, P157, DOI 10.1002/jnr.20033

Sullivan PG, 2000, NEUROSCIENCE, V101, P289, DOI 10.1016/S0306-4522(00)00380-8

Svendsen CN, 1996, EXP NEUROL, V137, P376, DOI 10.1006/exnr.1996.0039

TROJANOWSKI JQ, 1993, EXP NEUROL, V122, P283, DOI 10.1006/exnr.1993.1128

Uchino H, 2002, NEUROBIOL DIS, V10, P219, DOI 10.1006/nbdi.2002.0514

Vescovi AL, 1999, EXP NEUROL, V156, P71, DOI 10.1006/exnr.1998.6998

Vescovi AL, 1999, J NEUROTRAUM, V16, P689, DOI 10.1089/neu.1999.16.689

Wang HG, 1999, SCIENCE, V284, P339, DOI 10.1126/science.284.5412.339

Wennberg L, 2001, TRANSPLANTATION, V71, P1797, DOI 10.1097/00007890-200106270-00016

Wennersten A, 2004, J NEUROSURG, V100, P88, DOI 10.3171/jns.2004.100.1.0088

Wennersten A, 2003, ACTA NEUROPATHOL, V105, P281, DOI 10.1007/s00401-002-0649-y

NR 49

TC 47

Z9 51

U1 0

U2 5

PU ACADEMIC PRESS INC ELSEVIER SCIENCE

PI SAN DIEGO

PA 525 B ST, STE 1900, SAN DIEGO, CA 92101-4495 USA

SN 0014-4886

EI 1090-2430

J9 EXP NEUROL

JI Exp. Neurol.

PD JUN

PY 2006

VL 199

IS 2

BP 339

EP 347

DI 10.1016/j.expneurol.2005.12.035

PG 9

WC Neurosciences

WE Science Citation Index Expanded (SCI-EXPANDED)

SC Neurosciences & Neurology

GA 063DB

UT WOS:000238995200014

PM 16490195

DA 2023-06-10

ER

PT J

AU Itoh, T

Satou, T

Hashimoto, S

Ito, H

AF Itoh, T

Satou, T

Hashimoto, S

Ito, H

TI Isolation of neural stem cells from damaged rat cerebral cortex after

traumatic brain injury

SO NEUROREPORT

LA English

DT Article

DE culture; nestin; neural stem cell; neurosphere; traumatic brain injury

ID CENTRAL-NERVOUS-SYSTEM; CORTICAL IMPACT INJURY; ADULT-RAT;

PROLIFERATION; EXPRESSION; NESTIN; NEUROGENESIS; ASTROCYTES; NEURONS

AB Nestin-positive cells were seen around the damaged area at 24 h, 72 In and 7 days after rat traumatic brain injury. Tissue was isolated from around the damaged area at 72 h after injury and spheres were cultured with basic fibroblast growth factor and epidermal growth factor. These spheres could not be isolated at 24 h and 7 days after injury. Isolated spheres consisted of nestin-positive neural stem cells. Neurospheres differentiated into Tujl-positive, glial fibrillary acidic protein-positive and O4-positive cells after 4 days in culture without basic fibroblast growth factor and epidermal growth factor. These results indicate that isolated and cultured neurospheres can differentiate into neurons and glia. An increase in nestin-positive cells around a cerebral cortical damaged area might contribute to neurogenesis and neuroplasticity.

C1 Kinki Univ, Sch Med, Dept Pathol, Osaka 589, Japan.

Kinki Univ, Sch Med, Div Hosp Pathol, Osaka, Japan.

Kinki Univ, Div Sports Med, Inst Life Sci, Osaka, Japan.

PL Hosp, Div Pathol, Osaka, Japan.

C3 Kindai University (Kinki University); Kindai University (Kinki

University); Kindai University (Kinki University)

RP Itoh, T (通讯作者)，377-2,Ohno Higashi, Osaka 5898511, Japan.

EM tatsuki@med.kindai.ac.jp

OI Itoh, Tatsuki/0000-0003-2015-7788

CR Arvidsson A, 2002, NAT MED, V8, P963, DOI 10.1038/nm747

Chen S, 2003, EXP NEUROL, V182, P87, DOI 10.1016/S0014-4886(03)00002-5

Chirumamilla S, 2002, J NEUROTRAUM, V19, P693, DOI 10.1089/08977150260139084

Douen AG, 2004, BRAIN RES, V1008, P139, DOI 10.1016/j.brainres.2003.08.070

Gage FH, 2000, SCIENCE, V287, P1433, DOI 10.1126/science.287.5457.1433

GOODMAN JC, 1994, J NEUROTRAUM, V11, P587, DOI 10.1089/neu.1994.11.587

Johansson CB, 2002, J NEUROSCI RES, V69, P784, DOI 10.1002/jnr.10376

Kuhn HG, 1996, J NEUROSCI, V16, P2027

LOIS C, 1994, SCIENCE, V264, P1145, DOI 10.1126/science.8178174

Magavi SS, 2000, NATURE, V405, P951, DOI 10.1038/35016083

Moon C, 2004, BRAIN RES, V1028, P238, DOI 10.1016/j.brainres.2004.09.022

Picard-Riera N, 2004, J NEUROSCI RES, V76, P223, DOI 10.1002/jnr.20040

REYNOLDS BA, 1992, SCIENCE, V255, P1707, DOI 10.1126/science.1553558

Seri B, 2001, J NEUROSCI, V21, P7153, DOI 10.1523/JNEUROSCI.21-18-07153.2001

Xiong Y, 1997, J NEUROTRAUM, V14, P23, DOI 10.1089/neu.1997.14.23

Yamamoto S, 2001, EXP NEUROL, V172, P115, DOI 10.1006/exnr.2001.7798

Yanagisawa M, 2000, J NEUROCHEM, V74, P1498, DOI 10.1046/j.1471-4159.2000.0741498.x

NR 17

TC 55

Z9 73

U1 1

U2 2

PU LIPPINCOTT WILLIAMS & WILKINS

PI PHILADELPHIA

PA TWO COMMERCE SQ, 2001 MARKET ST, PHILADELPHIA, PA 19103 USA

SN 0959-4965

EI 1473-558X

J9 NEUROREPORT

JI Neuroreport

PD OCT 17

PY 2005

VL 16

IS 15

BP 1687

EP 1691

DI 10.1097/01.wnr.0000183330.44112.ab

PG 5

WC Neurosciences

WE Science Citation Index Expanded (SCI-EXPANDED)

SC Neurosciences & Neurology

GA 981FW

UT WOS:000233073900015

PM 16189478

DA 2023-06-10

ER

PT J

AU Willing, AE

Das, M

Howell, M

Mohapatra, SS

Mohapatra, S

AF Willing, Alison E.

Das, Mahasweta

Howell, Mark

Mohapatra, Shyam S.

Mohapatra, Subhra

TI Potential of mesenchymal stem cells alone, or in combination, to treat

traumatic brain injury

SO CNS NEUROSCIENCE & THERAPEUTICS

LA English

DT Review

DE clinical trials; combination treatment; stem cells; traumatic brain

injury

ID MARROW STROMAL CELLS; PROGRESSIVE MULTIPLE-SCLEROSIS; IMPROVES

NEUROLOGICAL FUNCTION; RAT MODEL; NEUROTROPHIC FACTORS; FUNCTIONAL

RECOVERY; ISCHEMIC-STROKE; CLINICAL-TRIALS; INFLAMMATORY RESPONSES;

CEREBRAL-ISCHEMIA

AB Traumatic brain injury (TBI) causes death and disability in the United States and around the world. The traumatic insult causes the mechanical injury of the brain and primary cellular death. While a comprehensive pathological mechanism of TBI is still lacking, the focus of the TBI research is concentrated on understanding the pathophysiology and developing suitable therapeutic approaches. Given the complexities in pathophysiology involving interconnected immunologic, inflammatory, and neurological cascades occurring after TBI, the therapies directed to a single mechanism fail in the clinical trials. This has led to the development of the paradigm of a combination therapeutic approach against TBI. While there are no drugs available for the treatment of TBI, stem cell therapy has shown promising results in preclinical studies. But, the success of the therapy depends on the survival of the stem cells, which are limited by several factors including route of administration, health of the administered cells, and inflammatory microenvironment of the injured brain. Reducing the inflammation prior to cell administration may provide a better outcome of cell therapy following TBI. This review is focused on different therapeutic approaches of TBI and the present status of the clinical trials.

C1 [Willing, Alison E.] Univ S Florida, Morsani Coll Med, Dept Neurosurg & Brain Repair, Ctr Excellence Aging & Brain Repair, Tampa, FL 33620 USA.

[Das, Mahasweta; Howell, Mark; Mohapatra, Subhra] Univ S Florida, Morsani Coll Med, Dept Mol Med, Tampa, FL 33620 USA.

[Das, Mahasweta; Howell, Mark; Mohapatra, Shyam S.; Mohapatra, Subhra] James A Haley Vet Hosp, Tampa, FL 33612 USA.

[Mohapatra, Shyam S.] Univ S Florida, Morsani Coll Med, Dept Internal Med, Tampa, FL 33620 USA.

C3 State University System of Florida; University of South Florida; State

University System of Florida; University of South Florida; US Department

of Veterans Affairs; Veterans Health Administration (VHA); James A.

Haley Veterans Hospital; State University System of Florida; University

of South Florida

RP Willing, AE (通讯作者)，Ctr Excellence Aging & Brain Repair, Dept Neurosurg & Brain Repair, 3515 E Fletcher Ave,MDC78, Tampa, FL 33613 USA.

EM awilling@usf.edu

RI Willing, Alison/AAP-7759-2021

OI , Alison/0000-0002-8482-1708

FU BLRD VA [IK6 BX003778, I01 BX002668, IK6 BX004212] Funding Source:

Medline

CR Acosta SA, 2013, PLOS ONE, V8, DOI 10.1371/journal.pone.0053376

Ajmo CT, 2006, CURR NEUROVASC RES, V3, P89, DOI 10.2174/156720206776875849

Alawieh A, 2018, J NEUROSCI, V38, P2519, DOI 10.1523/JNEUROSCI.2197-17.2018

Aloe L, 2012, J TRANSL MED, V10, DOI 10.1186/1479-5876-10-239

Aminmansour Bahram, 2014, Adv Biomed Res, V3, P35, DOI 10.4103/2277-9175.125031

Bang OY, 2005, ANN NEUROL, V57, P874, DOI 10.1002/ana.20501

Barnabe GF, 2009, PLOS ONE, V4, DOI 10.1371/journal.pone.0005222

Beauchamp K, 2008, MOL MED, V14, P731, DOI 10.2119/2008-00050.Beauchamp

Bhang SH, 2007, BIOCHEM BIOPH RES CO, V359, P40, DOI 10.1016/j.bbrc.2007.05.046

Bisicchia E, 2018, MOL NEUROBIOL, V55, P6894, DOI 10.1007/s12035-018-0889-z

Bonab MM, 2012, CURR STEM CELL RES T, V7, P407

Horcajo CB, 2018, CYTOTHERAPY, V20, P314, DOI 10.1016/j.jcyt.2017.11.012

Bramlett HM, 2004, J CEREBR BLOOD F MET, V24, P133, DOI 10.1097/01.WCB.0000111614.19196.04

Cajal SRY, 1991, CAJALS DEGENERATION, DOI 10.1093/acprof:oso/9780195065169.001.0001

Carbonara M, 2018, FRONT NEUROL, V9, DOI 10.3389/fneur.2018.00885

Cekic M, 2011, NEUROBIOL AGING, V32, P864, DOI 10.1016/j.neurobiolaging.2009.04.017

Centers for Disease Control and Prevention, 2014, SURV REP TRAUM BRAIN

Chakraborty S, 2016, CURR NEUROL NEUROSCI, V16, DOI 10.1007/s11910-016-0625-x

Chau MJ, 2018, BMC NEUROSCI, V19, DOI 10.1186/s12868-018-0418-z

Chen JL, 2011, STROKE, V42, P3551, DOI 10.1161/STROKEAHA.111.627174

Chen Q, 2005, J NEUROSCI RES, V80, P611, DOI 10.1002/jnr.20494

Chen SF, 2007, LIFE SCI, V81, P288, DOI 10.1016/j.lfs.2007.05.023

Chen XG, 2002, J NEUROSCI RES, V69, P687, DOI 10.1002/jnr.10334

Connick P, 2012, LANCET NEUROL, V11, P150, DOI 10.1016/S1474-4422(11)70305-2

Cox CS, 2019, TRANSFUSION, V59, P858, DOI 10.1111/trf.14834

Cox CS, 2017, STEM CELLS, V35, P1065, DOI 10.1002/stem.2538

Cox CS, 2011, NEUROSURGERY, V68, P588, DOI 10.1227/NEU.0b013e318207734c

Croft AP, 2006, STEM CELLS, V24, P1841, DOI 10.1634/stemcells.2005-0609

Meirelles LDS, 2006, J CELL SCI, V119, P2204, DOI 10.1242/jcs.02932

Dahbour S, 2017, CNS NEUROSCI THER, V23, P866, DOI 10.1111/cns.12759

Das M, 2019, SCI REP-UK, V9, DOI 10.1038/s41598-019-49428-y

Das M, 2011, J NEUROINFLAMM, V8, DOI 10.1186/1742-2094-8-148

Dekmak A, 2018, BEHAV BRAIN RES, V340, P49, DOI 10.1016/j.bbr.2016.12.039

del Zoppo Gregory J., 2009, V25, P34, DOI 10.1159/000209471

Diez-Tejedor E, 2014, J STROKE CEREBROVASC, V23, P2694, DOI 10.1016/j.jstrokecerebrovasdis.2014.06.011

Donega V, 2015, PEDIATR RES, V78, P520, DOI 10.1038/pr.2015.145

Dong H, 2016, CELL MOL NEUROBIOL, V36, P639, DOI 10.1007/s10571-015-0244-0

Dunkerson J, 2014, RESTOR NEUROL NEUROS, V32, P675, DOI 10.3233/RNN-140408

Faul Mark, 2015, Handb Clin Neurol, V127, P3, DOI 10.1016/B978-0-444-52892-6.00001-5

Feinklestein SP, 1999, STROKE, V30, P2752, DOI 10.1161/01.STR.30.12.2752

Feng Y, 2017, MOL MED REP, V16, P654, DOI 10.3892/mmr.2017.6619

Fernandez O, 2018, PLOS ONE, V13, DOI 10.1371/journal.pone.0195891

Fisher M, 2009, STROKE, V40, P2244, DOI 10.1161/STROKEAHA.108.541128

Furmanski O, 2019, JOVE-J VIS EXP, DOI 10.3791/59561

Garcia-Olmo D, 2005, DIS COLON RECTUM, V48, P1416, DOI 10.1007/s10350-005-0052-6

Goverman J, 2009, NAT REV IMMUNOL, V9, P393, DOI 10.1038/nri2550

Guo SW, 2017, NEUROPSYCH DIS TREAT, V13, P2757, DOI 10.2147/NDT.S141534

Gurkoff G, 2013, PHARMACEUTICALS, V6, P788, DOI 10.3390/ph6070788

Gutierrez-Fernandez M, 2013, STEM CELL RES THER, V4, DOI 10.1186/scrt159

Hall ED, 2010, NEUROTHERAPEUTICS, V7, P51, DOI 10.1016/j.nurt.2009.10.021

Hatton J, 2006, J NEUROSURG, V105, P843, DOI 10.3171/jns.2006.105.6.843

Hawkins KE, 2014, J NEUROCHEM, V129, P130, DOI 10.1111/jnc.12607

Hazeldine J, 2015, FRONT NEUROL, V6, DOI 10.3389/fneur.2015.00235

He J, 2004, RESTOR NEUROL NEUROS, V22, P19

Hess DC, 2014, INT J STROKE, V9, P381, DOI 10.1111/ijs.12065

Hoffer ME, 2013, PLOS ONE, V8, DOI [10.1371/journal.pone.0060061, 10.1371/journal.pone.0054163]

Hong SQ, 2011, NEUROCHEM RES, V36, P2391, DOI 10.1007/s11064-011-0567-2

Hoover DB, 2017, PHARMACOL THERAPEUT, V179, P1, DOI 10.1016/j.pharmthera.2017.05.002

Hosseini SM, 2015, INT J STEM CELLS, V8, P99, DOI 10.15283/ijsc.2015.8.1.99

Hu JN, 2019, STEM CELL RES THER, V10, DOI 10.1186/s13287-019-1210-4

Huang CJ, 2016, BIOMED MATER, V11, DOI 10.1088/1748-6041/11/3/035004

Huat TJ, 2014, BMC NEUROSCI, V15, DOI 10.1186/1471-2202-15-91

Iaccarino C, 2018, J NEUROSURG SCI, V62, P535, DOI 10.23736/S0390-5616.18.04532-0

Isele NB, 2007, NEUROCHEM INT, V50, P243, DOI 10.1016/j.neuint.2006.08.007

Jassam YN, 2017, NEURON, V95, P1246, DOI 10.1016/j.neuron.2017.07.010

Jonhagen ME, 1998, DEMENT GERIATR COGN, V9, P246, DOI 10.1159/000017069

Karussis D, 2010, ARCH NEUROL-CHICAGO, V67, P1187, DOI 10.1001/archneurol.2010.248

Kim DK, 2016, P NATL ACAD SCI USA, V113, P170, DOI 10.1073/pnas.1522297113

Kim Hee Jin, 2015, Alzheimers Dement (N Y), V1, P95, DOI 10.1016/j.trci.2015.06.007

Kim HJ, 2010, J NEUROTRAUM, V27, P131, DOI [10.1089/neu.2008.0818, 10.1089/neu.2008-0818]

Kinder HA, 2019, NEURAL REGEN RES, V14, P413, DOI 10.4103/1673-5374.245334

Konigs M, 2018, ARCH PHYS MED REHAB, V99, P1149, DOI 10.1016/j.apmr.2018.01.013

Kota DJ, 2016, STEM CELL TRANSL MED, V5, P33, DOI 10.5966/sctm.2015-0065

Ladak AA, 2019, WORLD NEUROSURG, V131, P126, DOI 10.1016/j.wneu.2019.07.039

Le Belle JE, 2004, J NEUROSCI RES, V76, P174, DOI 10.1002/jnr.20035

Le Blanc K, 2003, SCAND J IMMUNOL, V57, P11, DOI 10.1046/j.1365-3083.2003.01176.x

Lee JS, 2010, STEM CELLS, V28, P1099, DOI 10.1002/stem.430

Leonardo CC, 2012, TRANSL STROKE RES, V3, P357, DOI 10.1007/s12975-012-0203-8

Lepore AC, 2004, NEURON GLIA BIOL, V1, P113, DOI 10.1017/S1740925X04000213

Li JF, 2014, CELL TRANSPLANT, V23, pS113, DOI 10.3727/096368914X685005

Li L, 2012, J CEREBR BLOOD F MET, V32, P2023, DOI 10.1038/jcbfm.2012.106

Li XS, 1998, MOL BRAIN RES, V57, P92, DOI 10.1016/S0169-328X(98)00075-8

Liao GP, 2015, PEDIATR CRIT CARE ME, V16, P245, DOI 10.1097/PCC.0000000000000324

Lin CH, 2019, J FORMOS MED ASSOC, V118, P1661, DOI 10.1016/j.jfma.2019.01.008

Liu XY, 2020, J NEUROCHEM, V153, P230, DOI 10.1111/jnc.14859

Liu Y, 2014, MOL MED REP, V9, P333, DOI 10.3892/mmr.2013.1803

Liu YWY, 2018, J NEUROINFLAMM, V15, DOI 10.1186/s12974-018-1173-x

Liu Y, 2014, J NEUROINFLAMM, V11, DOI 10.1186/1742-2094-11-135

Livingston DH, 2003, ANN SURG, V238, P748, DOI 10.1097/01.sla.0000094441.38807.09

Lloyd J, 2015, BRAIN INJURY, V29, P539, DOI 10.3109/02699052.2014.1002003

Llufriu S, 2014, PLOS ONE, V9, DOI 10.1371/journal.pone.0113936

Lu D, 2001, NEUROREPORT, V12, P559, DOI 10.1097/00001756-200103050-00025

Lu DY, 2001, J NEUROTRAUM, V18, P813, DOI 10.1089/089771501316919175

Lu P, 2004, J NEUROSCI RES, V77, P174, DOI 10.1002/jnr.20148

Luo CL, 2013, BRAIN RES, V1502, P1, DOI 10.1016/j.brainres.2013.01.037

Ma H, 2019, CELL TRANSPLANT, V28, P874, DOI 10.1177/0963689719855624

Mahmood A, 2006, J NEUROSURG, V104, P272, DOI 10.3171/jns.2006.104.2.272

Mahmood A, 2004, J NEUROTRAUM, V21, P33, DOI 10.1089/089771504772695922

Mahmood A, 2001, NEUROSURGERY, V49, P1196, DOI 10.1097/00006123-200111000-00031

Mahmood A, 2002, J NEUROTRAUM, V19, P1609, DOI 10.1089/089771502762300265

Mahmood A, 2007, NEUROSURGERY, V60, P546, DOI 10.1227/01.NEU.0000255346.25959.99

Mahmood A, 2008, J NEUROTRAUM, V25, P1441, DOI 10.1089/neu.2007.0495

Maitra B, 2004, BONE MARROW TRANSPL, V33, P597, DOI 10.1038/sj.bmt.1704400

Martini AC, 2014, BIOMED RES INT, V2014, DOI 10.1155/2014/316204

Matsuda S, 2000, IMMUNOPHARMACOLOGY, V47, P119, DOI 10.1016/S0162-3109(00)00192-2

Matthay MA, 2017, STEM CELLS, V35, P316, DOI 10.1002/stem.2551

Matthews LR, 2013, INT J CASE REPORTS I, V4, P143, DOI DOI 10.5348/ijcri-2013-03-281-CS-2

Minnich JE, 2010, RESTOR NEUROL NEUROS, V28, P293, DOI 10.3233/RNN-2010-0528

Moretti A, 2015, PHARMACOL THERAPEUT, V146, P23, DOI 10.1016/j.pharmthera.2014.09.003

Morganti JM, 2016, J NEUROINFLAMM, V13, DOI 10.1186/s12974-016-0547-1

Morris GF, 1999, J NEUROSURG, V91, P737, DOI 10.3171/jns.1999.91.5.0737

Muir JK, 1999, J NEUROTRAUM, V16, P403, DOI 10.1089/neu.1999.16.403

Muir KW, 2006, CURR OPIN PHARMACOL, V6, P53, DOI 10.1016/j.coph.2005.12.002

MUIZELAAR JP, 1995, J NEUROSURG, V83, P942

Narayan RK, 2002, J NEUROTRAUM, V19, P503, DOI 10.1089/089771502753754037

National Center for Health S. Health United States, 2017, HLTH US 2016 CHARTB

Ng SY, 2019, FRONT CELL NEUROSCI, V13, DOI 10.3389/fncel.2019.00528

Ni HQ, 2019, FRONT NEUROSCI-SWITZ, V13, DOI 10.3389/fnins.2019.00014

Nizamutdinov D, 2017, BRAIN SCI, V7, DOI 10.3390/brainsci7010011

Oh JS, 2010, NEUROSCI LETT, V472, P215, DOI 10.1016/j.neulet.2010.02.008

Oh KW, 2015, STEM CELL TRANSL MED, V4, P590, DOI 10.5966/sctm.2014-0212

Palella FJ, 1998, NEW ENGL J MED, V338, P853, DOI 10.1056/NEJM199803263381301

Peruzzaro ST, 2019, J NEUROINFLAMM, V16, DOI 10.1186/s12974-018-1383-2

Pischiutta F, 2018, EXP NEUROL, V300, P167, DOI 10.1016/j.expneurol.2017.11.003

Riess P, 2002, NEUROSURGERY, V51, P1043, DOI 10.1097/00006123-200210000-00035

Riess P, 2007, J NEUROTRAUM, V24, P216, DOI 10.1089/neu.2006.0141

Riordan NH, 2018, J TRANSL MED, V16, DOI 10.1186/s12967-018-1433-7

Romeu-Mejia R, 2019, CURR REV MUSCULOSKE, V12, P105, DOI 10.1007/s12178-019-09536-8

Roth S, 2016, SWISS MED WKLY, V146, DOI 10.4414/smw.2016.14329

Satani N, 2019, BRAIN RES, V1720, DOI 10.1016/j.brainres.2019.06.017

Scafidi J, 2014, NATURE, V506, P230, DOI 10.1038/nature12880

Schmidt RH, 1999, J NEUROTRAUM, V16, P1139, DOI 10.1089/neu.1999.16.1139

Schwulst SJ, 2013, J TRAUMA ACUTE CARE, V75, P780, DOI 10.1097/TA.0b013e318299616a

Serhan CN, 2014, NATURE, V510, P92, DOI 10.1038/nature13479

Shahror RA, 2019, J NEUROTRAUM, DOI 10.1089/neu.2019.6422

Shamsara Ali, 2018, BioMolecular Concepts, V9, P155, DOI 10.1515/bmc-2018-0014

Shi W, 2016, ACTA BIOMATER, V45, P247, DOI 10.1016/j.actbio.2016.09.001

Shin DA, 2013, ACTA NEUROCHIR, V155, P1943, DOI 10.1007/s00701-013-1799-5

Shin MS, 2016, INT NEUROUROL J, V20, pS49, DOI 10.5213/inj.1632616.308

Singh R, 2019, CLIN NEUROL NEUROSUR, V186, DOI 10.1016/j.clineuro.2019.105526

Skolnick BE, 2014, NEW ENGL J MED, V371, P2467, DOI 10.1056/NEJMoa1411090

SOARES H, 1991, Journal of Neural Transplantation and Plasticity, V2, P207

Soares HD, 1995, J NEUROTRAUM, V12, P1059, DOI 10.1089/neu.1995.12.1059

Sorby-Adams AJ, 2018, AM J PHYSIOL-REG I, V315, pR165, DOI 10.1152/ajpregu.00163.2017

Steinberg GK, 2016, STROKE, V47, P1817, DOI 10.1161/STROKEAHA.116.012995

Talsky A, 2011, BC MED J, V53, P1

Tang HL, 2013, CELL REPROGRAM, V15, P435, DOI 10.1089/cell.2012.0081

Tang HL, 2015, BRAIN INJURY, V29, P1165, DOI 10.3109/02699052.2015.1035330

Tatebayashi K, 2019, BRAIN RES, V1712, P139, DOI 10.1016/j.brainres.2019.01.037

Thakor Devang K, 2018, Curr Protoc Stem Cell Biol, V47, pe58, DOI 10.1002/cpsc.58

Tian CL, 2013, EXP CLIN TRANSPLANT, V11, P176, DOI 10.6002/ect.2012.0053

van Eijck MM, 2018, BRAIN INJURY, V32, P395, DOI 10.1080/02699052.2018.1429018

VanLandingham JW, 2007, NEUROSCI LETT, V425, P94, DOI 10.1016/j.neulet.2007.08.045

Vaquero J, 2017, CYTOTHERAPY, V19, P349, DOI 10.1016/j.jcyt.2016.12.002

Vaquero J, 2017, CYTOTHERAPY, V19, P88, DOI 10.1016/j.jcyt.2016.10.001

Vazquez-Rosa E, 2019, ACS CHEM NEUROSCI, V10, P1595, DOI 10.1021/acschemneuro.8b00543

Volovici V, 2019, J NEUROTRAUM, V36, P3183, DOI 10.1089/neu.2019.6474

Walker PA, 2009, DIS MODEL MECH, V2, P23, DOI 10.1242/dmm.001198

Walker WC, 2018, J NEUROTRAUM, V35, P1587, DOI 10.1089/neu.2017.5359

Wang S, 2013, BRAIN RES, V1532, P76, DOI 10.1016/j.brainres.2013.08.001

Wang Z, 2015, STEM CELLS, V33, P456, DOI 10.1002/stem.1878

Wang ZM, 2017, NPG ASIA MATER, V9, DOI 10.1038/am.2017.171

Warden DL, 2006, J NEUROTRAUM, V23, P1468, DOI 10.1089/neu.2006.23.1468

Williams AM, 2019, J NEUROTRAUM, V36, P54, DOI 10.1089/neu.2018.5711

Woodbury D, 2002, J NEUROSCI RES, V69, P908, DOI 10.1002/jnr.10365

Wu K, 2019, STEM CELL RES THER, V10, DOI 10.1186/s13287-019-1428-1

Xiong Y, 2017, NEURAL REGEN RES, V12, P19, DOI 10.4103/1673-5374.198966

Xu KY, 2019, HAEMATOLOGICA, V104, P1062, DOI 10.3324/haematol.2018.206581

Xu L, 2019, FRONT CELL NEUROSCI, V12, DOI 10.3389/fncel.2018.00498

Yan T, 2013, PLOS ONE, V8, DOI 10.1371/journal.pone.0081199

Yang YX, 2017, FRONT CELL NEUROSCI, V11, DOI 10.3389/fncel.2017.00055

Yasmin A, 2019, FRONT NEUROSCI-SWITZ, V13, DOI 10.3389/fnins.2019.00863

Zeng X, 2016, J BIOMED MATER RES A, V104, P1902, DOI 10.1002/jbm.a.35720

Zhang AM, 2012, MOL MED REP, V6, P1315, DOI 10.3892/mmr.2012.1069

Zhang R, 2013, J NEUROINFLAMM, V10, DOI 10.1186/1742-2094-10-106

Zhang YL, 2017, NEUROCHEM INT, V111, P69, DOI 10.1016/j.neuint.2016.08.003

NR 176

TC 13

Z9 15

U1 1

U2 8

PU WILEY

PI HOBOKEN

PA 111 RIVER ST, HOBOKEN 07030-5774, NJ USA

SN 1755-5930

EI 1755-5949

J9 CNS NEUROSCI THER

JI CNS Neurosci. Ther.

PD JUN

PY 2020

VL 26

IS 6

SI SI

BP 616

EP 627

DI 10.1111/cns.13300

PG 12

WC Neurosciences; Pharmacology & Pharmacy

WE Science Citation Index Expanded (SCI-EXPANDED)

SC Neurosciences & Neurology; Pharmacology & Pharmacy

GA LQ8CX

UT WOS:000535226500004

PM 32157822

OA Green Published, gold

DA 2023-06-10

ER

PT J

AU Alizada, M

Lin, S

Gao, HZ

AF Alizada, Mujahid

Lin, Shu

Gao, Hongzhi

TI Recent advances in the treatment of traumatic brain injury with

autologous and non-autologous multipotent stem and progenitor cells:

preclinical models and clinical trials

SO FOLIA NEUROPATHOLOGICA

LA English

DT Review

DE traumatic brain injury; stem cells; transplantation; autologous;

non-autologous; clinical trial; preclinical model

ID MARROW STROMAL CELLS; PROMOTES FUNCTIONAL RECOVERY; OLFACTORY

ENSHEATHING CELLS; UMBILICAL-CORD BLOOD; RAT MODEL; NEUROTROPHIC

FACTORS; COGNITIVE DEFICITS; SYNAPTIC PROTEIN; UP-REGULATION; IN-VIVO

AB Traumatic brain injury (TeI) is a global health issue which causes millions of deaths and disabilities every year. The survivors of TeI may suffer from sensorimotor dysfunction, memory and cognitive disturbances, hearing and vision deficits, and various psychological problems. The primary insult may damage neurons, cerebral vessels and the blood-brain barrier, causing reactive astrogliosis and immune response with further damaging consequences. TeI lacks effective therapy. The currently available clinical treatment options include hyperbaric oxygenation, brain stimulation and rehabilitation. In recent years, the research on stem cell treatment of TeI has received extensive attention. Various types of stem cells, such as four types of mesenchymal stem cells, neural stem cells and olfactory ensheathing cells have been tried to treat TeI in clinical trials and preclinical models. This article reviews the research of autologous and non-autologous multipotent stem and progenitor cells for the treatment of TeI in both clinical and preclinical settings.

C1 [Alizada, Mujahid; Lin, Shu] Fujian Med Univ, Affiliated Hosp 2, Ctr Neurol & Metab Res, Quanzhou, Fujian, Peoples R China.

[Alizada, Mujahid; Gao, Hongzhi] Fujian Med Univ, Affiliated Hosp 2, Dept Neurosurg, Quanzhou, Fujian, Peoples R China.

[Lin, Shu] Garvan Inst Med Res, Diabetes & Metab Div, Sydney, NSW, Australia.

C3 Fujian Medical University; Fujian Medical University; Garvan Institute

of Medical Research

RP Lin, S (通讯作者)，Fujian Med Univ, Affiliated Hosp 2, Ctr Neurol & Metab Res, Quanzhou, Fujian, Peoples R China.; Gao, HZ (通讯作者)，Fujian Med Univ, Affiliated Hosp 2, Dept Neurosurg, Quanzhou, Fujian, Peoples R China.

EM shulin1956@126.com; 1564747628@qq.com

FU Science and Technology Bureau of Quanzhou [2020CT003]

FX This work was supported by the Science and Technology Bureau of Quanzhou

(grant number 2020CT003) .

CR Albert-WeiSSenberger Christiane, 2012, Exp Transl Stroke Med, V4, P1, DOI 10.1186/2040-7378-4-1

Anbari F, 2014, NEURAL REGEN RES, V9, P919, DOI 10.4103/1673-5374.133133

Au E, 2003, GLIA, V41, P224, DOI 10.1002/glia.10160

Barraud P, 2010, P NATL ACAD SCI USA, V107, P21040, DOI 10.1073/pnas.1012248107

Bedi SS, 2013, J TRAUMA ACUTE CARE, V75, P410, DOI 10.1097/TA.0b013e31829617c6

Beretta S, 2017, CELL TRANSPLANT, V26, P1247, DOI 10.1177/0963689717714107

Bhang SH, 2007, BIOCHEM BIOPH RES CO, V359, P40, DOI 10.1016/j.bbrc.2007.05.046

Bonilla C, 2012, J TRAUMA ACUTE CARE, V72, P1203, DOI 10.1097/TA.0b013e318248bdcf

Cameron HA, 2001, J COMP NEUROL, V435, P406, DOI 10.1002/cne.1040

Caplan HW, 2020, STEM CELL TRANSL MED, V9, P903, DOI 10.1002/sctm.19-0444

Cernak I, 1996, J TRAUMA, V40, pS100, DOI 10.1097/00005373-199603001-00023

Chen KH, 2020, CELL TRANSPLANT, V29, DOI 10.1177/0963689720929313

Chen T, 2017, NEUROCHEM RES, V42, P3073, DOI 10.1007/s11064-017-2340-7

Chen ZY, 2009, J NEUROTRAUM, V26, P1987, DOI 10.1089/neu.2008.0863

Coronado VG, 2012, J SAFETY RES, V43, P299, DOI 10.1016/j.jsr.2012.08.011

Cox CS, 2018, PEDIATR RES, V83, P325, DOI 10.1038/pr.2017.253

Cox CS, 2017, STEM CELLS, V35, P1065, DOI 10.1002/stem.2538

Cox CS, 2011, NEUROSURGERY, V68, P588, DOI 10.1227/NEU.0b013e318207734c

Dang BQ, 2017, NEURAL PLAST, V2017, DOI 10.1155/2017/1582182

Das M, 2019, REV NEUROSCIENCE, V30, P839, DOI 10.1515/revneuro-2019-0002

Deng QJ, 2018, CELL MOL NEUROBIOL, V38, P467, DOI 10.1007/s10571-017-0490-4

Denny-Brown D E, 1941, Proc R Soc Med, V34, P691

Dong HJ, 2017, J CRANIOFAC SURG, V28, P1615, DOI 10.1097/SCS.0000000000003563

Feng Y, 2017, MOL MED REP, V16, P654, DOI 10.3892/mmr.2017.6619

Fu XM, 2015, CELL TRANSPLANT, V24, P1533, DOI 10.3727/096368914X679345

Fuentealba LC, 2015, CELL, V161, P1644, DOI 10.1016/j.cell.2015.05.041

Gao JL, 2016, CELL TRANSPLANT, V25, P1863, DOI 10.3727/096368916X691150

Gao YH, 2014, APPL BIOCHEM BIOTECH, V174, P682, DOI 10.1007/s12010-014-1100-2

Gincberg G, 2018, J MOL NEUROSCI, V64, P185, DOI 10.1007/s12031-017-1008-8

Gincberg G, 2018, CYTOTHERAPY, V20, P245, DOI 10.1016/j.jcyt.2017.11.008

Gladwin K, 2015, WORLD NEUROSURG, V83, P114, DOI 10.1016/j.wneu.2013.03.010

Guo K, 2020, EXP MOL PATHOL, V114, DOI 10.1016/j.yexmp.2020.104416

Hackenberg K, 2016, NERVENARZT, V87, P203, DOI 10.1007/s00115-015-0051-3

Harmon KG, 2013, CLIN J SPORT MED, V23, P1, DOI 10.1097/JSM.0b013e31827f5f93

Haus DL, 2016, EXP NEUROL, V281, P1, DOI 10.1016/j.expneurol.2016.04.008

Hawley CA, 2003, INJURY, V34, P256, DOI 10.1016/S0020-1383(02)00193-6

Hu HT, 2020, ANN TRANSL MED, V8, DOI 10.21037/atm.2020.03.52

Hu JN, 2019, STEM CELL RES THER, V10, DOI 10.1186/s13287-019-1210-4

Hu W, 2018, FOLIA NEUROPATHOL, V56, P112, DOI 10.5114/fn.2018.76615

Hu Z, 2020, J TRAUMA ACUTE CARE, V88, P477, DOI 10.1097/TA.0000000000002510

Hyder AA, 2007, NEUROREHABILITATION, V22, P341

Imayoshi I, 2008, NAT NEUROSCI, V11, P1153, DOI 10.1038/nn.2185

Jagnoor J, 2014, AUST FAM PHYSICIAN, V43, P758

Jahan-Abad AJ, 2018, MOL NEUROBIOL, V55, P9122, DOI 10.1007/s12035-018-1050-8

Jiang JD, 2012, NEURAL REGEN RES, V7, P46, DOI 10.3969/j.issn.1673-5374.2012.01.008

Jung A, 2018, BRAIN INJURY, V32, P1834, DOI 10.1080/02699052.2018.1510542

Kappy NS, 2018, J TRAUMA ACUTE CARE, V84, P745, DOI 10.1097/TA.0000000000001770

Kochanek PM, 2019, NEUROSURGERY, V84, P1169, DOI 10.1093/neuros/nyz051

Kriegstein A, 2009, ANNU REV NEUROSCI, V32, P149, DOI 10.1146/annurev.neuro.051508.135600

Kumar A, 2012, BRAIN BEHAV IMMUN, V26, P1191, DOI 10.1016/j.bbi.2012.06.008

Lee JY, 2019, THERANOSTICS, V9, P1029, DOI 10.7150/thno.29868

Li L, 2017, BRAIN RES, V1675, P61, DOI 10.1016/j.brainres.2017.09.007

Li LA, 2011, J NEUROTRAUM, V28, P535, DOI 10.1089/neu.2010.1619

Liao GP, 2015, PEDIATR CRIT CARE ME, V16, P245, DOI 10.1097/PCC.0000000000000324

Lin GQ, 2018, NEUROSCI LETT, V674, P11, DOI 10.1016/j.neulet.2018.02.064

Lipton ML, 2012, BRAIN IMAGING BEHAV, V6, P329, DOI 10.1007/s11682-012-9175-2

Liu SJ, 2014, J NEUROINFLAMM, V11, DOI 10.1186/1742-2094-11-66

Liu WP, 2008, NEUROSCI LETT, V434, P160, DOI 10.1016/j.neulet.2007.12.067

Lu DY, 2001, J NEUROTRAUM, V18, P813, DOI 10.1089/089771501316919175

Ma HY, 2011, MOL MED REP, V4, P849, DOI 10.3892/mmr.2011.510

Ma H, 2019, CELL TRANSPLANT, V28, P874, DOI 10.1177/0963689719855624

Maas AIR, 2017, LANCET NEUROL, V16, P987, DOI 10.1016/S1474-4422(17)30371-X

Mahmood A, 2002, J NEUROTRAUM, V19, P1609, DOI 10.1089/089771502762300265

MARMAROU A, 1994, J NEUROSURG, V80, P291, DOI 10.3171/jns.1994.80.2.0291

Mastro-Martinez I, 2015, BRAIN INJURY, V29, P1497, DOI 10.3109/02699052.2015.1053525

Mirzadeh Z, 2008, CELL STEM CELL, V3, P265, DOI 10.1016/j.stem.2008.07.004

Mitra B, 2006, ANZ J SURG, V76, P343, DOI 10.1111/j.1445-2197.2006.03723.x

Moreau F, 2013, CEREBROVASC DIS EXTR, V3, P130, DOI 10.1159/000355024

Mundra V, 2013, MOL PHARMACEUT, V10, P77, DOI 10.1021/mp3005148

Nam HS, 2009, CELL STEM CELL, V5, P515, DOI 10.1016/j.stem.2009.08.017

Nasser M, 2018, FRONT NEUROL, V9, DOI 10.3389/fneur.2018.00895

Osanai T, 2012, NEUROSURGERY, V70, P435, DOI 10.1227/NEU.0b013e318230a795

Pang AL, 2017, CELL TRANSPLANT, V26, P1262, DOI 10.1177/0963689717715168

Peeters W, 2015, ACTA NEUROCHIR, V157, P1683, DOI 10.1007/s00701-015-2512-7

Qi LF, 2018, J CRANIOFAC SURG, V29, P1689, DOI 10.1097/SCS.0000000000005042

Ray SK, 2002, HISTOL HISTOPATHOL, V17, P1137, DOI 10.14670/HH-17.1137

Roet KCD, 2014, EXP NEUROL, V261, P594, DOI 10.1016/j.expneurol.2014.05.007

Ruppert KA, 2020, PLOS ONE, V15, DOI 10.1371/journal.pone.0233263

Sharma A, 2015, SPRINGERPLUS, V4, DOI 10.1186/s40064-015-0794-0

Sharma Alok K, 2020, Cell Regen, V9, P3, DOI 10.1186/s13619-020-00043-7

Sharp DJ, 2014, INT PSYCHOGERIATR, V26, P1591, DOI 10.1017/S1041610214001689

Shen Q, 2016, CELL PHYSIOL BIOCHEM, V38, P748, DOI 10.1159/000443031

Shetty AK, 2014, FRONT CELL NEUROSCI, V8, DOI 10.3389/fncel.2014.00232

Shi XD, 2018, CELL TISSUE RES, V372, P67, DOI 10.1007/s00441-017-2716-7

Shlosberg D, 2010, NAT REV NEUROL, V6, P393, DOI 10.1038/nrneurol.2010.74

Skandsen T, 2010, J NEUROSURG, V113, P556, DOI 10.3171/2009.9.JNS09626

Skardelly M, 2011, J NEUROTRAUM, V28, P401, DOI 10.1089/neu.2010.1526

Spurlock MS, 2017, J NEUROTRAUM, V34, P1981, DOI 10.1089/neu.2016.4602

Srivastava AK, 2019, REGEN MED, V14, P295, DOI 10.2217/rme-2018-0106

Sun D, 2011, J NEUROTRAUM, V28, P961, DOI 10.1089/neu.2010.1697

Taylor CA, 2017, MMWR SURVEILL SUMM, V66, P1, DOI 10.15585/mmwr.ss6609a1

Tian CL, 2013, EXP CLIN TRANSPLANT, V11, P176, DOI 10.6002/ect.2012.0053

Titus DJ, 2013, J NEUROSCI, V33, P5216, DOI 10.1523/JNEUROSCI.5133-12.2013

Ventura RE, 2014, LANCET NEUROL, V13, P1006, DOI 10.1016/S1474-4422(14)70111-5

WALKER AE, 1994, J NEUROSURG, V81, P493, DOI 10.3171/jns.1994.81.3.0493

WANG CC, 1986, ARCH NEUROL-CHICAGO, V43, P570, DOI 10.1001/archneur.1986.00520060034013

Wang YM, 2019, TURK NEUROSURG, V29, P750, DOI 10.5137/1019-5149.JTN.25463-18.2

Wang YC, 2014, CYTOTHERAPY, V16, P1000, DOI 10.1016/j.jcyt.2013.12.009

Wang ZG, 2017, EXP THER MED, V13, P3613, DOI 10.3892/etm.2017.4423

Weston NM, 2018, CURR NEUROL NEUROSCI, V18, DOI 10.1007/s11910-018-0812-z

Wewetzer K, 2005, GLIA, V49, P577, DOI 10.1002/glia.20149

Xiong LL, 2018, MOL NEUROBIOL, V55, P2696, DOI 10.1007/s12035-017-0551-1

Xiong Y, 2008, BRAIN RES, V1230, P247, DOI 10.1016/j.brainres.2008.06.127

Xu L, 2019, FRONT CELL NEUROSCI, V12, DOI 10.3389/fncel.2018.00498

Yan ZJ, 2013, NEUROCHEM RES, V38, P1022, DOI 10.1007/s11064-013-1012-5

Yu B, 2013, ARCH MED SCI, V9, P132, DOI 10.5114/aoms.2012.31438

Yuan XY, 2020, BRAIN BEHAV, V10, DOI 10.1002/brb3.1675

Zhang J, 2015, BIOMED RES INT, V2015, DOI 10.1155/2015/727542

Zhang ZX, 2008, CYTOTHERAPY, V10, P134, DOI 10.1080/14653240701883061

Zhao CM, 2008, CELL, V132, P645, DOI 10.1016/j.cell.2008.01.033

NR 110

TC 2

Z9 2

U1 3

U2 7

PU TERMEDIA PUBLISHING HOUSE LTD

PI POZNAN

PA KLEEBERGA ST 2, POZNAN, 61-615, POLAND

SN 1641-4640

EI 1509-572X

J9 FOLIA NEUROPATHOL

JI Folia Neuropathol.

PY 2021

VL 59

IS 3

BP 298

EP 316

DI 10.5114/fn.2021.108536

PG 19

WC Neurosciences; Pathology

WE Science Citation Index Expanded (SCI-EXPANDED)

SC Neurosciences & Neurology; Pathology

GA WD6YO

UT WOS:000705084900011

PM 34628796

OA gold

DA 2023-06-10

ER

PT J

AU Wei, ZZ

Lee, JH

Zhang, YB

Zhu, YB

Deveau, TC

Gu, XH

Winter, MM

Li, JM

Wei, L

Yu, SP

AF Wei, Zheng Zachory

Lee, Jin Hwan

Zhang, Yongbo

Zhu, Yan Bing

Deveau, Todd C.

Gu, Xiaohuan

Winter, Megan M.

Li, Jimei

Wei, Ling

Yu, Shan Ping

TI Intracranial Transplantation of Hypoxia-Preconditioned iPSC-Derived

Neural Progenitor Cells Alleviates Neuropsychiatric Defects After

Traumatic Brain Injury in Juvenile Rats

SO CELL TRANSPLANTATION

LA English

DT Article

DE Induced pluripotent stem cell-derived neural progenitor cells

(iPSC-NPCs); Hypoxic preconditioning; Regeneration; Juveniles; Traumatic

brain injury (TBI); Posttraumatic disorders

ID PHARMACOLOGICALLY INDUCED HYPOTHERMIA; COMBINING ENRICHED ENVIRONMENT;

EMBRYONIC STEM-CELLS; CLOSED-HEAD-INJURY; NEURONAL DIFFERENTIATION;

INTRANASAL DELIVERY; CEREBRAL-ISCHEMIA; OXYTOCIN RELEASE; STROKE;

VASOPRESSIN

AB Traumatic brain injury (TBI) is a common cause of mortality and long-term morbidity in children and adolescents. Posttraumatic stress disorder (PTSD) frequently develops in these patients, leading to a variety of neuropsychiatric syndromes. Currently, few therapeutic strategies are available to treat juveniles with PTSD and other developmental neuropsychiatric disorders. In the present investigation, postnatal day 14 (P14) Wistar rats were subjected to TBI induced by a controlled cortical impact (CCI) (velocity = 3 m/s, depth = 2.0 mm, contact time = 150 ms). This TBI injury resulted in not only cortical damages, but also posttrauma social behavior deficits. Three days after TBI, rats were treated with intracranial transplantation of either mouse iPSC-derived neural progenitor cells under normal culture conditions (N-iPSC-NPCs) or mouse iPSC-derived neural progenitor cells pretreated with hypoxic preconditioning (HP-iPSC-NPCs). Compared to TBI animals that received N-iPSC-NPCs or vehicle treatment, HP-iPSC-NPC-transplanted animals showed a unique benefit of improved performance in social interaction, social novelty, and social transmission of food preference tests. Western blotting showed that HP-iPSC-NPCs expressed significantly higher levels of the social behavior -related genes oxytocin and the oxytocin receptor. Overall, HP-iPSC-NPC transplantation exhibits a great potential as a regenerative therapy to improve neuropsychiatric outcomes after juvenile TBI.

C1 [Wei, Zheng Zachory; Zhang, Yongbo; Zhu, Yan Bing; Li, Jimei; Wei, Ling] Capital Med Univ, Beijing Friendship Hosp, Expt Res Ctr, Dept Neurol,Labs Stem Cell Biol & Regenerat Med, Beijing, Peoples R China.

[Wei, Zheng Zachory; Zhang, Yongbo; Zhu, Yan Bing; Li, Jimei; Wei, Ling] Capital Med Univ, Beijing Friendship Hosp, Ctr Neurol Dis, Beijing, Peoples R China.

[Wei, Zheng Zachory; Lee, Jin Hwan; Deveau, Todd C.; Gu, Xiaohuan; Winter, Megan M.; Wei, Ling; Yu, Shan Ping] Emory Univ, Sch Med, Dept Anesthesiol, 101 Woodruff Circle WMRB Suite 620, Atlanta, GA 30322 USA.

[Yu, Shan Ping] Atlanta VA Med Ctr, Ctr Visual & Neurocognit Rehabil, Decatur, GA USA.

C3 Capital Medical University; Capital Medical University; Emory

University; US Department of Veterans Affairs; Veterans Health

Administration (VHA); Atlanta VA Health Care System; Atlanta VA Medical

Center

RP Yu, SP (通讯作者)，Emory Univ, Sch Med, Dept Anesthesiol, 101 Woodruff Circle WMRB Suite 620, Atlanta, GA 30322 USA.

EM spyu@emory.edu

RI Wei, Zheng Zachory/I-2109-2019

OI Wei, Zheng Zachory/0000-0001-7682-9286

FU NIH [NS091585, NS062097, NS085568]; AHA [12GRNT12060222]; VA National

Merit grant [RX000666]; AHA Predoctoral/Postdoctoral Fellowships

[15POST25710112, 15POST 25680013, 14PRE18830026]; National Natural

Science Foundation of China [81371355/81500989]; Beijing Natural Science

Foundation [7142045]

FX This work was supported by NIH grants NS091585 (L.W), NS062097 (LW), and

NS085568 (LW.); AHA Grant-in-Aid 12GRNT12060222 (S.P.Y.); VA National

Merit grant RX000666 (S.P.Y.); AHA Predoctoral/Postdoctoral Fellowships

15POST25710112 (Z.Z.W.), 15POST 25680013 (J.H.L.), and 14PRE18830026

(T.C.D.); National Natural Science Foundation of China 81371355/81500989

(Y.Z.); and Beijing Natural Science Foundation 7142045 (Y.Z.). The

authors declare no conflicts of interest.

CR Adriani W, 2006, PSYCHOPHARMACOLOGY, V184, P155, DOI 10.1007/s00213-005-0223-0

All AH, 2015, PLOS ONE, V10, DOI 10.1371/journal.pone.0116933

Anderson VA, 2004, BRAIN, V127, P2608, DOI 10.1093/brain/awh320

Backeljauw B, 2014, PM&R, V6, P814, DOI 10.1016/j.pmrj.2014.04.004

Baharnoori M, 2012, SCHIZOPHRENIA BULL, V38, P444, DOI 10.1093/schbul/sbq098

Blaya MO, 2015, EXP NEUROL, V264, P67, DOI 10.1016/j.expneurol.2014.11.014

Chau MJ, 2014, STEM CELLS, V32, P3075, DOI 10.1002/stem.1802

Choi KE, 2012, FASEB J, V26, P2799, DOI 10.1096/fj.11-201822

Coronado Victor G., 2011, Morbidity and Mortality Weekly Report, V60, P1

Dunkerson J, 2014, RESTOR NEUROL NEUROS, V32, P675, DOI 10.3233/RNN-140408

Eckert A, 2015, STEM CELL TRANSL MED, V4, P841, DOI 10.5966/sctm.2014-0184

Eichenbaum H, 2000, NAT REV NEUROSCI, V1, P41, DOI 10.1038/35036213

FILLEY CM, 1987, ARCH NEUROL-CHICAGO, V44, P194, DOI 10.1001/archneur.1987.00520140058018

Francis KR, 2010, CELL DEATH DIS, V1, DOI 10.1038/cddis.2009.22

GALEF BG, 1987, DEV PSYCHOBIOL, V20, P209, DOI 10.1002/dev.420200209

Gao K, 2013, GLIA, V61, P2063, DOI 10.1002/glia.22577

Garcia D, 2015, J PEDIATR PSYCHOL, V40, P391, DOI 10.1093/jpepsy/jsu093

GomezHernandez R, 1997, ARCH PHYS MED REHAB, V78, P1321, DOI 10.1016/S0003-9993(97)90304-X

Gu XH, 2015, EXP NEUROL, V267, P135, DOI 10.1016/j.expneurol.2015.02.029

Harbert MJ, 2012, STROKE RES TREAT, V2012, DOI 10.1155/2012/914546

Hu XY, 2008, J THORAC CARDIOV SUR, V135, P799, DOI 10.1016/j.jtcvs.2007.07.071

Johnston MV, 2011, LANCET NEUROL, V10, P372, DOI 10.1016/S1474-4422(11)70016-3

Keightley ML, 2014, FRONT HUM NEUROSCI, V8, DOI 10.3389/fnhum.2014.00139

Lam J, 2014, ADV FUNCT MATER, V24, P7053, DOI 10.1002/adfm.201401483

Landgraf R, 2004, FRONT NEUROENDOCRIN, V25, P150, DOI 10.1016/j.yfrne.2004.05.001

Lee JH, 2015, AM J PHYSIOL-CELL PH, V308, pC570, DOI 10.1152/ajpcell.00353.2014

Lee JH, 2014, J NEUROTRAUM, V31, P1417, DOI 10.1089/neu.2013.3251

Lepperhof V, 2014, PLOS ONE, V9, DOI 10.1371/journal.pone.0107363

LEVIN HS, 1995, J NEUROTRAUM, V12, P601, DOI 10.1089/neu.1995.12.601

Mason Christine Narad, 2013, Pediatr Nurs, V39, P267

Max JE, 2014, PSYCHIAT CLIN N AM, V37, P125, DOI 10.1016/j.psc.2013.11.003

McDonald S., 2014, BRAIN BEHAV COGNITIO, V2, DOI 10.4324/9780203557198

Meyer-Lindenberg A, 2011, NAT REV NEUROSCI, V12, P524, DOI 10.1038/nrn3044

Mohamad O, 2013, DIFFERENTIATION, V86, P149, DOI 10.1016/j.diff.2013.12.002

Mohamad O, 2013, PLOS ONE, V8, DOI 10.1371/journal.pone.0064160

Mu SH, 2014, PLOS ONE, V9, DOI 10.1371/journal.pone.0101185

Nudi ET, 2015, J NEUROTRAUM, V32, P1117, DOI 10.1089/neu.2014.3618

Paquin J, 2002, P NATL ACAD SCI USA, V99, P9550, DOI 10.1073/pnas.152302499

Qin I., 2015, PLOS ONE, V10

Romanyuk N, 2015, CELL TRANSPLANT, V24, P1781, DOI 10.3727/096368914X684042

Runyan DK, 2008, AM J PREV MED, V34, pS112, DOI 10.1016/j.amepre.2008.01.011

Sun JM, 2015, EXP NEUROL, V272, P78, DOI 10.1016/j.expneurol.2015.03.011

Szeto A, 2008, AM J PHYSIOL-ENDOC M, V295, pE1495, DOI 10.1152/ajpendo.90718.2008

Temkin NR, 2009, J HEAD TRAUMA REHAB, V24, P460, DOI 10.1097/HTR.0b013e3181c13413

Theus MH, 2008, EXP NEUROL, V210, P656, DOI 10.1016/j.expneurol.2007.12.020

van Handel M, 2010, J PEDIATR PSYCHOL, V35, P286, DOI 10.1093/jpepsy/jsp049

Veenema AH, 2008, PROG BRAIN RES, V170, P261, DOI 10.1016/S0079-6123(08)00422-6

Wang S, 2013, BRAIN RES, V1532, P76, DOI 10.1016/j.brainres.2013.08.001

Wang Z, 2015, ACS NANO, V9, P6683, DOI 10.1021/acsnano.5b00690

Wei L, 2012, NEUROBIOL DIS, V46, P635, DOI 10.1016/j.nbd.2012.03.002

Wei ZZ, 2015, CELL TRANSPLANT, V24, P391, DOI 10.3727/096368915X686887

Wei ZZ, 2014, CELL MOL NEUROBIOL, V34, P881, DOI 10.1007/s10571-014-0067-4

Weitzdoerfer R, 2004, SEMIN PERINATOL, V28, P249, DOI 10.1053/j.semperi.2004.08.001

Wrenn C. C., 2004, CURR PROTOC NEUROSCI, V28

Yu SP, 2013, TRANSL STROKE RES, V4, P76, DOI 10.1007/s12975-012-0251-0

NR 55

TC 26

Z9 26

U1 1

U2 12

PU SAGE PUBLICATIONS INC

PI THOUSAND OAKS

PA 2455 TELLER RD, THOUSAND OAKS, CA 91320 USA

SN 0963-6897

EI 1555-3892

J9 CELL TRANSPLANT

JI Cell Transplant.

PY 2016

VL 25

IS 5

BP 797

EP 809

DI 10.3727/096368916X690403

PG 13

WC Cell & Tissue Engineering; Medicine, Research & Experimental;

Transplantation

WE Science Citation Index Expanded (SCI-EXPANDED)

SC Cell Biology; Research & Experimental Medicine; Transplantation

GA DL0UY

UT WOS:000375349300003

PM 26766038

OA Bronze

DA 2023-06-10

ER

PT J

AU Blaya, MO

Tsoulfas, P

Bramlett, HM

Dietrich, WD

AF Blaya, Meghan O.

Tsoulfas, Pantelis

Bramlett, Helen M.

Dietrich, W. Dalton

TI Neural progenitor cell transplantation promotes neuroprotection,

enhances hippocampal neurogenesis, and improves cognitive outcomes after

traumatic brain injury

SO EXPERIMENTAL NEUROLOGY

LA English

DT Article

DE Traumatic brain injury; Neural progenitor cell transplantation;

Multineurotrophin; Hippocampal neurogenesis; Neuroprotection; Spatial

memory

ID ADULT NEUROGENESIS; DENTATE GYRUS; NEURONS; DIFFERENTIATION;

NEUROTROPHINS; ACTIVATION; RECEPTORS; SURVIVAL; POINT

AB Transplantation of neural progenitor cells (NPCs) may be a potential treatment strategy for traumatic brain injury (TBI) due to their intrinsic advantages, including the secretion of neurotrophins. Neurotrophins are critical for neuronal survival and repair, but their clinical use is limited. In this study, we hypothesized that pericontusional transplantation of NPCs genetically modified to secrete a synthetic, human multineurotrophin (MNTS1) would overcome some of the limitations of traditional neurotrophin therapy. MNTS1 is a multifunctional neurotrophin that binds all three tropomyosin-related kinase (Trk) receptors, recapitulating the prosurvival activity of 3 endogenous mature neurotrophins. NPCs obtained from rat fetuses at E15 were transduced with lentiviral vectors containing MNTS1 and GFP constructs (MNTS1-NPCs) or fluorescent constructs alone (control GFP-NPCs). Adult rats received fluid percussion-induced TBI or sham surgery. Animals were transplanted 1 week later with control GFP-NPCs, MNTS1-NPCs, or injected with saline (vehicle). At five weeks, animals were evaluated for hippocampal-dependent spatial memory. Six weeks post-surgery, we observed significant survival and neuronal differentiation of MNTS1-NPCs and injury-activated tropism toward contused regions. NPCs displayed processes that extended into several remote structures, including the hippocampus and contralateral cortex. Both GFP- and MNTS1-NPCs conferred significant preservation of pericontusional host tissues and enhanced hippocampal neurogenesis. NPC transplantation improved spatial memory capacity on the Morris water maze (MWM) task. Transplant recipients exhibited escape latencies approximately half that of injured vehicle controls. While we observed greater transplant survival and neuronal differentiation of MNTS1-NPCs, our collective findings suggest that MNTS1 may be superfluous in terms of preserving the cytoarchitecture and rescuing behavioral deficits given the lack of significant difference between MNTS1- and GFP-control transplanted groups. Nevertheless, our overall findings support the potential of syngeneic NPC transplantation to enhance endogenous neuroreparative responses and may therefore be an effective treatment for TBI. (C) 2014 Elsevier Inc. All rights reserved.

C1 [Blaya, Meghan O.; Tsoulfas, Pantelis; Bramlett, Helen M.; Dietrich, W. Dalton] Univ Miami, Miller Sch Med, Miami Project Cure Paralysis, Dept Neurol Surg, Miami, FL 33136 USA.

[Bramlett, Helen M.] Bruce W Carter Dept Vet Affairs Med Ctr, Miami, FL 33125 USA.

C3 University of Miami

RP Dietrich, WD (通讯作者)，Univ Miami, Miller Sch Med, Miami Project Cure Paralysis, Dept Neurol Surg, 1095 NW 14th Terrace, Miami, FL 33136 USA.

EM Moconnell2@med.miami.edu; Ptsoulfa@med.miami.edu;

Hbramlett@med.miami.edu; Ddietrich@miami.edu

RI Tsoulfas, Pantelis/GNH-1922-2022

OI Tsoulfas, Pantelis/0000-0003-1974-6366

FU NIH [NS030291, W81XWH-06-1-0187]

FX The studies were supported by NIH NS030291 to WDD and W81XWH-06-1-0187

awarded to WDD. The authors wish to thank Mrs. Ofelia Furones-Alonso,

Mr. Pingping Jia and Mrs. Yunfang Wang for their technical contributions

to this study, and to Dr. Coleen Atkins for proofreading the manuscript.

CR Alonso M, 2004, LEARN MEMORY, V11, P172, DOI 10.1101/lm.67804

Atkins CM, 2010, EUR J NEUROSCI, V32, P1912, DOI 10.1111/j.1460-9568.2010.07467.x

Bakshi A, 2006, EUR J NEUROSCI, V23, P2119, DOI 10.1111/j.1460-9568.2006.04743.x

Barnabe-Heider F, 2003, J NEUROSCI, V23, P5149

Blaiss CA, 2011, J NEUROSCI, V31, P4906, DOI 10.1523/JNEUROSCI.5265-10.2011

Bramlett HM, 2004, J CEREBR BLOOD F MET, V24, P133, DOI 10.1097/01.WCB.0000111614.19196.04

Bregy A, 2012, EXP NEUROL, V233, P821, DOI 10.1016/j.expneurol.2011.12.008

Bullock R, 1995, ANN NY ACAD SCI, V765, P290, DOI 10.1111/j.1749-6632.1995.tb16586.x

Chao MV, 2003, NAT REV NEUROSCI, V4, P299, DOI 10.1038/nrn1078

Cossetti C, 2012, CELL TISSUE RES, V349, P321, DOI 10.1007/s00441-012-1341-8

Dash PK, 2001, J NEUROSCI RES, V63, P313, DOI 10.1002/1097-4547(20010215)63:4<313::AID-JNR1025>3.3.CO;2-W

Deng W, 2010, NAT REV NEUROSCI, V11, P339, DOI 10.1038/nrn2822

Dull T, 1998, J VIROL, V72, P8463, DOI 10.1128/JVI.72.11.8463-8471.1998

Follenzi A, 2002, METHOD ENZYMOL, V346, P454

Gage FH, 2013, NEURON, V80, P588, DOI 10.1016/j.neuron.2013.10.037

Gao X, 2008, J NEUROSCI RES, V86, P2258, DOI 10.1002/jnr.21677

GUNDERSEN HJG, 1988, APMIS, V96, P857, DOI 10.1111/j.1699-0463.1988.tb00954.x

Harting MT, 2008, NEUROSURG FOCUS, V24, DOI 10.3171/FOC/2008/24/3-4/E17

Kumagai G, 2013, EXP NEUROL, V248, P369, DOI 10.1016/j.expneurol.2013.06.028

Lessmann V, 2003, PROG NEUROBIOL, V69, P341, DOI 10.1016/S0301-0082(03)00019-4

Martino G, 2006, NAT REV NEUROSCI, V7, P395, DOI 10.1038/nrn1908

Ming GL, 2011, NEURON, V70, P687, DOI 10.1016/j.neuron.2011.05.001

MorgantiKossman MC, 1997, MOL PSYCHIATR, V2, P133, DOI 10.1038/sj.mp.4000227

Pluchino S, 2005, NATURE, V436, P266, DOI 10.1038/nature03889

Reichardt LF, 2006, PHILOS T R SOC B, V361, P1545, DOI 10.1098/rstb.2006.1894

Rola R, 2006, EXP NEUROL, V202, P189, DOI 10.1016/j.expneurol.2006.05.034

Skold MK, 2005, J NEUROTRAUM, V22, P353, DOI 10.1089/neu.2005.22.353

Snyder JS, 2005, NEUROSCIENCE, V130, P843, DOI 10.1016/j.neuroscience.2004.10.009

URFER R, 1994, EMBO J, V13, P5896, DOI 10.1002/j.1460-2075.1994.tb06935.x

Urrea C, 2007, RESTOR NEUROL NEUROS, V25, P65

Walker PA, 2012, J NEUROINFLAMM, V9, P1742

Wu Y., 2012, TRANSL NEURODEGENER, V1, P2047

Xu Q, 2007, CLIN EXP PHARMACOL P, V34, P624, DOI 10.1111/j.1440-1681.2007.04619.x

Yoshimura S, 2001, P NATL ACAD SCI USA, V98, P5874, DOI 10.1073/pnas.101034998

Yu TS, 2008, J NEUROSCI, V28, P12901, DOI 10.1523/JNEUROSCI.4629-08.2008

Zhao CM, 2008, CELL, V132, P645, DOI 10.1016/j.cell.2008.01.033

Zhou Q, 2004, NEURON, V44, P749, DOI 10.1016/j.neuron.2004.11.011

NR 37

TC 52

Z9 52

U1 0

U2 10

PU ACADEMIC PRESS INC ELSEVIER SCIENCE

PI SAN DIEGO

PA 525 B ST, STE 1900, SAN DIEGO, CA 92101-4495 USA

SN 0014-4886

EI 1090-2430

J9 EXP NEUROL

JI Exp. Neurol.

PD FEB

PY 2015

VL 264

BP 67

EP 81

DI 10.1016/j.expneurol.2014.11.014

PG 15

WC Neurosciences

WE Science Citation Index Expanded (SCI-EXPANDED)

SC Neurosciences & Neurology

GA CB4IC

UT WOS:000349590400008

PM 25483396

OA Green Accepted

DA 2023-06-10

ER

PT J

AU Cozene, B

Sadanandan, N

Farooq, J

Kingsbury, C

Park, YJ

Wang, ZJ

Moscatello, A

Saft, M

Cho, J

Gonzales-Portillo, B

Borlongan, CV

AF Cozene, Blaise

Sadanandan, Nadia

Farooq, Jeffrey

Kingsbury, Chase

Park, You Jeong

Wang, Zhen-Jie

Moscatello, Alexa

Saft, Madeline

Cho, Justin

Gonzales-Portillo, Bella

Borlongan, Cesar, V

TI Mesenchymal Stem Cell-Induced Anti-Neuroinflammation Against Traumatic

Brain Injury

SO CELL TRANSPLANTATION

LA English

DT Review

DE traumatic brain injury; acquired brain injury; bone marrow-derived

mesenchymal stem cells; clinical trials; inflammation; preclinical

studies

ID NEUROLOGICAL DEFICITS; ISCHEMIC-STROKE; CHOROID-PLEXUS; IMMUNE CELLS;

INFLAMMATION; THERAPY; EXPRESSION; RAT; MODERATE; RESPONSES

AB Traumatic brain injury (TBI) is a pervasive and damaging form of acquired brain injury (ABI). Acute, subacute, and chronic cell death processes, as a result of TBI, contribute to the disease progression and exacerbate outcomes. Extended neuroinflammation can worsen secondary degradation of brain function and structure. Mesenchymal stem cell transplantation has surfaced as a viable approach as a TBI therapeutic due to its immunomodulatory and regenerative features. This article examines the role of inflammation and cell death in ABI as well as the effectiveness of bone marrow-derived mesenchymal stem/stromal cell (BM-MSC) transplants as a treatment for TBI. Furthermore, we analyze new studies featuring transplanted BM-MSCs as a neurorestorative and anti-inflammatory therapy for TBI patients. Although clinical trials support BM-MSC transplants as a viable TBI treatment due to their promising regenerative characteristics, further investigation is imperative to uncover innovative brain repair pathways associated with cell-based therapy as stand-alone or as combination treatments.

C1 [Cozene, Blaise] Tulane Univ, New Orleans, LA 70118 USA.

[Sadanandan, Nadia] Georgetown Univ, Washington, DC USA.

[Farooq, Jeffrey; Kingsbury, Chase; Park, You Jeong; Wang, Zhen-Jie; Moscatello, Alexa; Cho, Justin; Borlongan, Cesar, V] Univ S Florida, Dept Neurosurg & Brain Repair, Morsani Coll Med, Tampa, FL 33620 USA.

[Saft, Madeline] Univ Michigan, Ann Arbor, MI 48109 USA.

[Gonzales-Portillo, Bella] Northwestern Univ, Evanston, IL USA.

C3 Tulane University; Georgetown University; State University System of

Florida; University of South Florida; University of Michigan System;

University of Michigan; Northwestern University

RP Borlongan, CV (通讯作者)，Univ S Florida, Dept Neurosurg & Brain Repair, Morsani Coll Med, Tampa, FL 33620 USA.

EM cborlong@usf.edu

RI Park, You Jeong/GXG-7523-2022

FU National Institutes of Health (NIH) [R01NS090962]; NIH [R01NS102395,

R21NS109575]

FX The author(s) disclosed receipt of the following financial support for

the research, authorship, and/or publication of this article: CVB is

funded by National Institutes of Health (NIH) R01NS090962, NIH

R01NS102395, and NIH R21NS109575.

CR A Clinical Trial to Determine the Safety and Efficacy of Hope Biosciences Autologous Mesenchymal Stem Cell Therapy for the Treatment of Traumatic Brain Injury and Hypoxic-Ischemic Encephalopathy Status, CLIN TRIAL DETERMINE

Acosta SA, 2015, STROKE, V46, P2616, DOI 10.1161/STROKEAHA.115.009854

Acosta SA, 2013, PLOS ONE, V8, DOI 10.1371/journal.pone.0053376

Ajmo CT, 2008, J NEUROSCI RES, V86, P2227, DOI 10.1002/jnr.21661

Alvarado-Velez M, 2021, BIOMATERIALS, V266, DOI 10.1016/j.biomaterials.2020.120419

Anbari F, 2014, NEURAL REGEN RES, V9, P919, DOI 10.4103/1673-5374.133133

Andriessen TMJC, 2011, J NEUROTRAUM, V28, P2019, DOI 10.1089/neu.2011.2034

Appel S H, 2011, Acta Myol, V30, P4

Arnoux I, 2015, NEURAL PLAST, V2015, DOI 10.1155/2015/689404

Atkins CM, 2013, NEUROSCI LETT, V532, P1, DOI 10.1016/j.neulet.2012.10.019

Bai LH, 2009, GLIA, V57, P1192, DOI 10.1002/glia.20841

Bazarian JJ, 2009, J HEAD TRAUMA REHAB, V24, P439, DOI 10.1097/HTR.0b013e3181c15600

Beschorner R, 2002, ACTA NEUROPATHOL, V103, P541, DOI 10.1007/s00401-001-0503-7

Bonsack B, 2020, CNS NEUROSCI THER, V26, P603, DOI 10.1111/cns.13378

Borlongan C V, 1999, Lancet, V353 Suppl 1, pSI29

Borlongan CV, 2012, CURR PHARM DESIGN, V18, P3670

Borlongan CV, 2000, NEUROREPORT, V11, P923, DOI 10.1097/00001756-200004070-00005

Borlongan CV, 1996, NEUROL RES, V18, P297

Chen YF, 2020, AGING-US, V12, P18274, DOI 10.18632/aging.103692

Cox CS, 2017, STEM CELLS, V35, P1065, DOI 10.1002/stem.2538

Crane PK, 2016, JAMA NEUROL, V73, P1062, DOI 10.1001/jamaneurol.2016.1948

Dabrowska S, 2019, J NEUROINFLAMM, V16, DOI 10.1186/s12974-019-1571-8

Dailey T, 2013, J CLIN MED, V2, P220, DOI 10.3390/jcm2040220

Dang DD, 2018, CNS NEUROSCI THER, V24, P564, DOI 10.1111/cns.12823

Dantzer R, 2018, PHYSIOL REV, V98, P477, DOI 10.1152/physrev.00039.2016

Das M, 2012, J NEUROINFLAMM, V9, DOI 10.1186/1742-2094-9-236

Das M, 2011, J NEUROINFLAMM, V8, DOI 10.1186/1742-2094-8-148

de Oliveira A., 2016, J STEM CELL RES THER, V2, P59, DOI [10.15406/jsrt.2016.01.00012, DOI 10.15406/JSRT.2016.01.00012]

Dekmak A, 2018, BEHAV BRAIN RES, V340, P49, DOI 10.1016/j.bbr.2016.12.039

Dela Pena I, 2014, J NEUROSURG SCI, V58, P145

Donat CK, 2017, FRONT AGING NEUROSCI, V9, DOI 10.3389/fnagi.2017.00208

Emerich DF, 2006, NEUROBIOL DIS, V23, P471, DOI 10.1016/j.nbd.2006.04.014

Faustino J, 2019, J CEREBR BLOOD F MET, V39, P1919, DOI 10.1177/0271678X18817663

Fukumoto Y, 2019, J CEREBR BLOOD F MET, V39, P2144, DOI 10.1177/0271678X18805317

Garcia-Culebras A, 2018, J CEREBR BLOOD F MET, V38, P2150, DOI 10.1177/0271678X18795789

Giunta B, 2012, J NEUROINFLAMM, V9, DOI 10.1186/1742-2094-9-185

Greig NH, 2020, CNS NEUROSCI THER, V26, P636, DOI 10.1111/cns.13274

Grotenhuis N, 2016, TISSUE ENG PT A, V22, P1098, DOI [10.1089/ten.tea.2016.0162, 10.1089/ten.TEA.2016.0162]

Gu LJ, 2014, CNS NEUROSCI THER, V20, P67, DOI 10.1111/cns.12160

Guan FX, 2019, STEM CELL RES THER, V10, DOI 10.1186/s13287-019-1433-4

Hasan A, 2017, FRONT NEUROL, V8, DOI 10.3389/fneur.2017.00028

Hawthorne AL, 2011, NEUROTHERAPEUTICS, V8, P252, DOI 10.1007/s13311-011-0032-6

Hernandez-Ontiveros DG, 2013, FRONT NEUROL, V4, DOI 10.3389/fneur.2013.00030

Huang P, 2019, SCI REP-UK, V9, DOI 10.1038/s41598-019-42182-1

James SL, 2019, LANCET NEUROL, V18, P56, DOI [10.1016/S1474-4422(18)30499-X, 10.1016/S1474-4422(18)30415-0]

Janova H, 2016, GLIA, V64, P635, DOI 10.1002/glia.22955

Jenne CN, 2013, NAT IMMUNOL, V14, P996, DOI 10.1038/ni.2691

Jin R, 2010, J LEUKOCYTE BIOL, V87, P779, DOI 10.1189/jlb.1109766

Jordan BD, 2000, SEMIN NEUROL, V20, P179, DOI 10.1055/s-2000-9826
[truncated: 3,679,373 more chars]
